# Nanoenabling MbtI Inhibitors for Next-Generation Tuberculosis Therapy

**DOI:** 10.1021/acs.jmedchem.4c02386

**Published:** 2025-03-03

**Authors:** Giulia Cazzaniga, Matteo Mori, Anna Griego, Edoardo Scarpa, Giorgia Moschetti, Stefano Muzzioli, Giovanni Stelitano, Laurent R. Chiarelli, Mario Cocorullo, Emanuele Casali, Alessio Porta, Giuseppe Zanoni, Andrea Tresoldi, Elena Pini, Íris L. Batalha, Giuseppe Battaglia, Tiziano Tuccinardi, Loris Rizzello, Stefania Villa, Fiorella Meneghetti

**Affiliations:** † Department of Pharmaceutical Sciences, University of Milan, Via L. Mangiagalli 25, 20133 Milano, Italy; ‡ Department of Science and High Technology, University of Insubria, via Valleggio 9, 22100 Como, Italy; § National Institute of Molecular Genetic (INGM), Via F. Sforza 35, 20122 Milano, Italy; ∥ Department of Biology and Biotechnology “Lazzaro Spallanzani″, University of Pavia, via A. Ferrata 9, 27100 Pavia, Italy; ⊥ Department of Chemistry, University of Pavia, Viale T. Taramelli 12, 27100 Pavia, Italy; # Department of Life Sciences, University of Bath, Claverton Down, BA2 7AY Bath, U.K.; ∇ Molecular Bionics Group, Institute for Bioengineering of Catalonia (IBEC), C. Baldiri Reixac 10-12, 08028 Barcelona, Spain; ○ Catalan Institution of Research and Advanced Studies, (ICREA), Passeig de Lluís Companys, 23, 08010 Barcelona, Spain; ◆ Department of Pharmacy, University of Pisa, Via Bonanno Pisano 6, 56126 Pisa, Italy

## Abstract

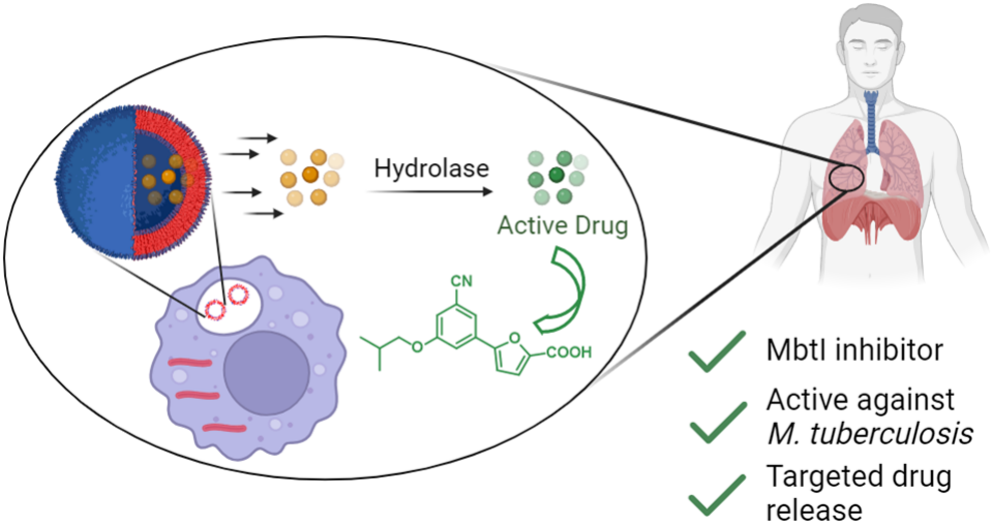

The urgent need for safer and innovative antitubercular agents remains a priority for the scientific community. In pursuit of this goal, we designed and evaluated novel 5-phenylfuran-2-carboxylic acid derivatives targeting *Mycobacterium tuberculosis* (*Mtb*) salicylate synthase (MbtI), a key enzyme, absent in humans, that plays a crucial role in *Mtb* virulence. Several potent MbtI inhibitors demonstrating significant antitubercular activity and a favorable safety profile were identified. Structure-guided optimization yielded 5-(3-cyano-5-isobutoxyphenyl)furan-2-carboxylic acid (**1e**), which exhibited strong MbtI inhibition (IC_50_ = 11.2 μM) and a promising *in vitro* antitubercular activity (MIC_99_ = 32 μM against *M. bovis* BCG). Esters of **1e** were effectively loaded into poly(2-methacryloyloxyethyl phosphorylcholine)-poly(2-(diisopropylamino)ethyl methacrylate) (PMPC–PDPA) polymersomes (POs) and delivered to intracellular mycobacteria, resulting in reduced *Mtb* viability. This study provides a foundation for the use of POs in the development of future MbtI-targeted therapies for tuberculosis.

## Introduction

**Figure 1:**
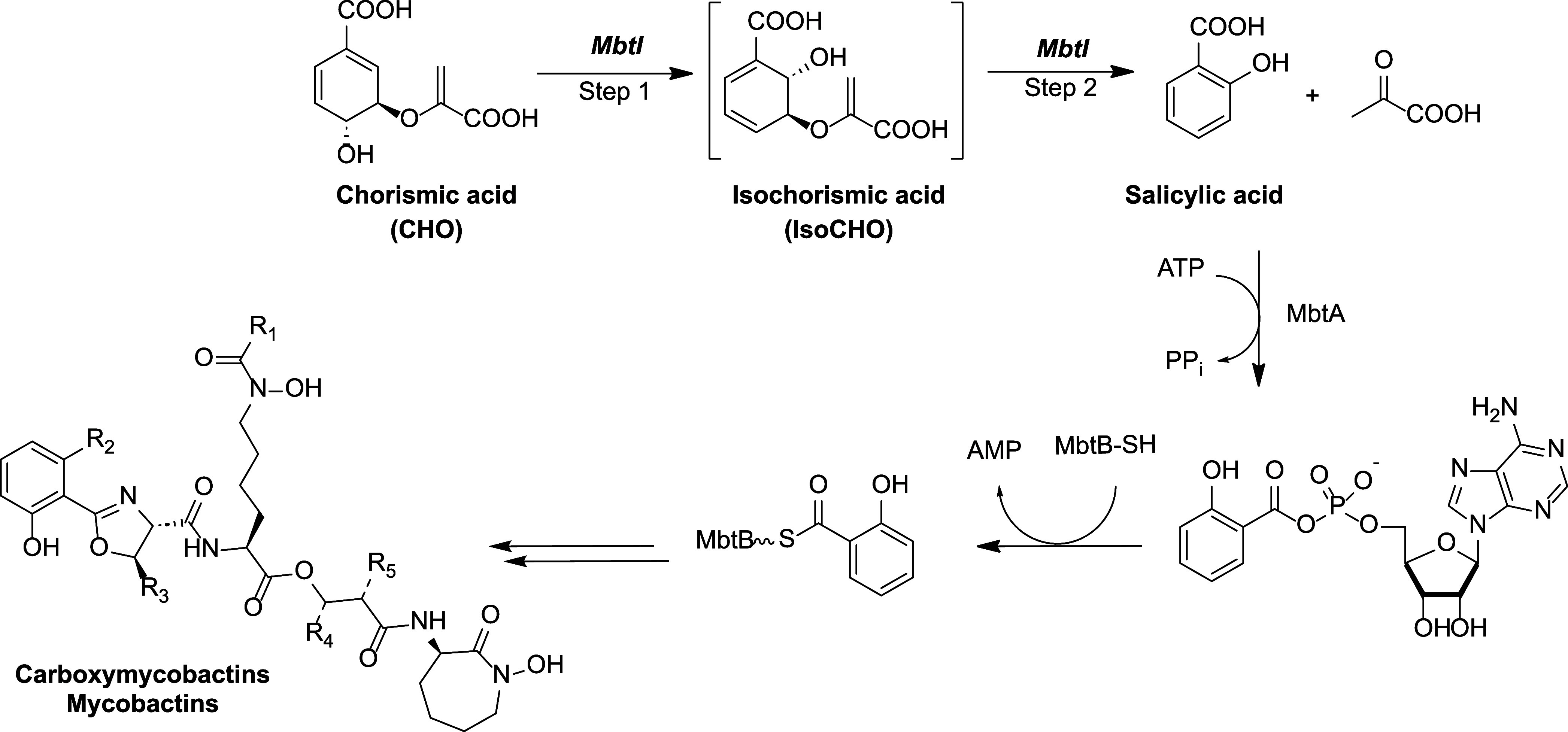
Biosynthetic pathway leading to the production of mycobactins and carboxymycobactins. The R groups are defined as follows: R_1_, R_4_ = CH_3_, CH_2_CH_3_, or long alkyl chain; R_2_, R_3_, R_5_ = H or CH_3_.

Tuberculosis (TB) is an infectious disease caused by *Mycobacterium tuberculosis* (*Mtb*), one of the oldest and most aggressive pathogens known to humanity.^1,2^ Nowadays, TB remains one of the leading causes of death worldwide.^2,3^ To counter the spread of this disease, the World Health Organization (WHO) has emphasized the need to intensify the search for new drugs and develop improved drug regimens that can shorten the current treatment.^3−6^ According to the most recent Global TB Report, published by the WHO in November 2024, the latest treatment guidelines for people with drug-susceptible TB (both pulmonary and extrapulmonary) recommend a 6-month regimen with isoniazid (H), rifampicin (R), ethambutol (E), and pyrazinamide (Z) for the first two months, followed by H and R for the remaining 4 months. The mortality rate is gradually returning to pre-COVID-19 levels, and the notifications have resumed their increase, exceeding the 2019 numbers.^3,7^ However, at the same time, multidrug-resistant (MDR) and extensively drug-resistant (XDR) infections continue to threaten public health, with no signs of decline.^3,8−10^ As a result, the 90% reduction target in TB deaths by 2030, envisioned by the UN Sustainable Development Goals, now seems highly unlikely.^11^ An analysis focusing on annual changes in mortality risk for 120 countries highlighted that this scenario will have major economic and demographic consequences, estimating a loss of 17.5 trillion USD, with the greatest effects expected in sub-Saharan Africa.^11^ In addition, because this analysis considers only mortality and excludes costs associated with TB-related disability, the economic loss may be underestimated.^12^ Therefore, the discovery of new antitubercular agents with novel mechanisms of action remains a priority for the development of effective therapeutic strategies.^13−16^ Compounds targeting pathways involved in pathogenesis, yet nonessential for the bacterial cell outside the host, are emerging as innovative and promising solutions.^17,18^ This approach, known as antivirulence therapy, differs from traditional antibiotic treatment in its purpose: instead of killing pathogens or halting their growth, it focuses on neutralizing their harmful mechanisms, making them less dangerous to the host.^19−22^ This enables the immune system to effectively eliminate the infection without intensifying the evolutionary fight between the host and the pathogen. By not threatening microbial survival or growth, antivirulence therapies reduce the selective pressure for resistance, minimizing the pathogen’s incentive to adapt.^17,18^ Moreover, unlike broad-spectrum antibiotics, these therapies typically spare beneficial microbial communities, further decreasing the likelihood of resistance development. Several examples of such compounds have been reported in recent years, including biofilm formation inhibitors, antiquorum sensing compounds, antiadhesion agents, or molecules that interfere with the acquisition of essential nutrients, critical for maintaining microbial virulence and successful colonization within the host.^19^ An interesting example of this strategy is provided by anti-*Pseudomonas aeruginosa* compounds that interfere with iron assimilation. Most notably, experimental studies have shown that these agents do not lead to resistance phenomena under test conditions.^23^ Such an approach has also been successfully applied for the development of antimycobacterial compounds targeting the biosynthesis of mycobactins and carboxymycobactins, a class of small-molecule siderophores (from the Greek, “iron carriers”) uniquely produced by the *Mycobacterium* genus.^24−27^ Iron acquisition is pivotal during the infection cycle of *Mtb*, as this metal acts as a cofactor in multiple biological pathways crucial for the survival and virulence of the microorganism within the host.^24^ The first enzyme involved in the production of these iron chelators is a salicylate synthase (MbtI in *Mtb*) that converts chorismic acid to salicylic acid (Figure 1).^24,28−30^

**Figure 2:**
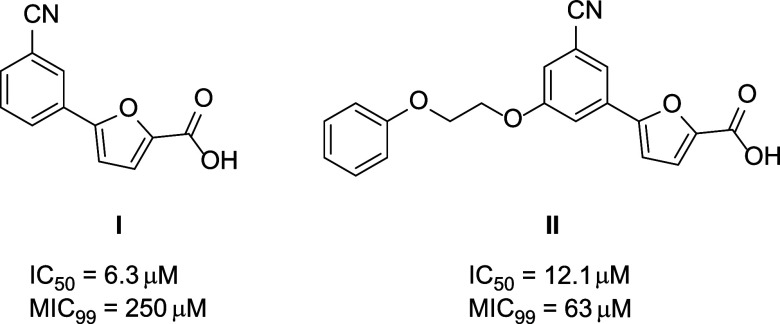
Structures and biological activities of 5-phenylfuran-based MbtI inhibitors **I** and **II**.

Therefore, MbtI inhibitors block the initial step of siderophore biosynthesis.^24^ These compounds can be broadly classified based on their chemical structure as chorismate,^31^ isochorismate,^32^ and transition-state analogs.^33^ High-throughput screenings and computational studies have also led to the identification of additional inhibitory scaffolds, including gallate, diarylsulfone, benzimidazole-2-thione, and furan.^34,35^ Recent advances in the discovery of MbtI inhibitors, along with the relevant biochemical mechanisms, are explored in several current reviews on the topic.^27−30,36^ In our previous studies, we successfully identified new compounds for the inhibition of MbtI, based on the 5-phenylfuran-2-carboxylic acid scaffold.^35,37−39^ Among the most promising derivatives, **I** (Figure 2) stood out for its potency on the enzyme (IC_50_ = 6.3 μM) and moderate antimycobacterial activity against *Mtb* H37Rv and *M. bovis* BCG (MIC_99_ = 250 μM).^38^ This compound, along with many other members of its class, was safe on several human cell lines.^37,40^ To improve its antimycobacterial effect, we performed structural modifications to increase its lipophilicity by adding an aromatic or alkyl-aromatic side chain at position 5 of the phenyl ring.^40^ It has been consistently observed that the antimycobacterial potency of many antitubercular agents increases with the lipophilicity of the molecule.^41^ This phenomenon has been attributed to the unique characteristics of the *Mtb* cell wall: with its thick layer of mycolic acids, this structure restricts the passive diffusion of hydrophilic compounds.^42^ Conversely, hydrophobic molecules are internalized more easily due to their improved liposolubility.^43,44^ Our efforts led to the discovery of **II** (Figure 2), which displayed a higher potency *in vitro* (Figure 2).^40^

Here, we expanded on our previous work to introduce a new series of derivatives of **I** (**1a**-**o**), substituted with a set of alkyl and alkenyl moieties at the same position. Our goal was to determine how the insertion of less sterically hindered lipophilic groups influenced the biological activity of the compounds to enhance their antimycobacterial effect. This approach proved to be successful, leading to the discovery of a new potent MbtI inhibitor, 5-(3-cyano-5-isobutoxyphenyl)furan-2-carboxylic acid (**1e**), exhibiting a significant activity against mycobacterial models *in vitro*. However, when **1e** was tested in *Mtb-*infected murine alveolar-like macrophages (mAMs), a reduction in *Mtb* intracellular burden was detected only at the highest tested concentration (200 μM). We speculated that this low efficacy might be related to difficulties encountered by **1e** in crossing the mAM cell membrane. Hence, to overcome this possible limitation, we successfully utilized synthetic polymeric vesicles, known as polymersomes (POs), loaded with ester derivatives of **1e** to effectively deliver the compound to the target cells. POs are versatile drug delivery systems capable of encapsulating a wide variety of substrates.^45−47^ Moreover, they can be modified to target specific delivery sites, minimizing toxicity and enhancing the efficacy of the drug.^46,48,49^ They are more stable than other carriers (*e.g*., liposomes) and maintain their structural integrity, preventing the release of the payload.^46,50^

We are confident that our investigations will contribute to advancing the antitubercular field, providing new therapeutic agents acting on novel molecular pathways and offering useful insights into advanced delivery systems. Our results will open new avenues for effective treatments and propose strategies to enhance the precision and efficacy of current therapeutic interventions.

## Results and Discussion

### Design Strategy

**Figure 3:**
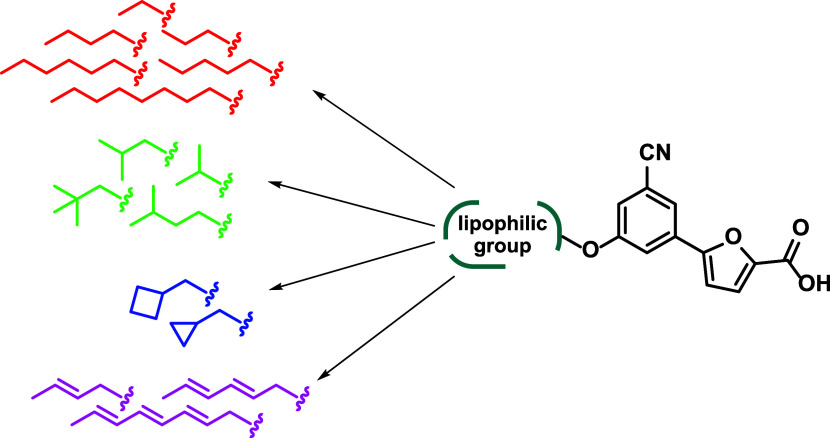
Overview of the lipophilic derivatives synthesized in this work.

It has been consistently observed that the antimycobacterial potency of many antitubercular agents increases with the lipophilicity of the molecule.^41,51,52^ This phenomenon has been attributed to the unique characteristics of the *Mtb* cell wall: with its thick layer of mycolic acids, this structure restricts the passive diffusion of hydrophilic compounds.^42^ Conversely, hydrophobic molecules are internalized more easily due to their improved liposolubility.^43,44^ In our efforts to improve our class of 5-phenylfuran-2-carboxylic acid derivatives, we expanded our previous library to include new analogs bearing alkyl or alkenyl moieties at position 5 of the phenyl ring. The choice of this substitution pattern was guided by analyzing the cocrystal structure of the lead compound **I** with the target MbtI.^38^ This analysis suggested that the substitution at position 5 of the phenyl ring would preserve the crucial interactions within the binding pocket.^38^ Previously, this approach successfully led to the discovery of compound **II** (Figure 2), which demonstrated significantly improved antimycobacterial action. This enhanced efficacy is likely due to the compound’s superior ability to penetrate the cell wall, highlighting the success and potential of our strategy.^40^ Applying the same rationale, we decided to test the effect of less bulky alkyl or alkenyl chains. We deemed this step crucial to expand our understanding of how clogP influences antimycobacterial efficacy, potentially leading to the development of more effective treatments. To this end, we varied the chain length (2–8 carbon atoms), steric hindrance (branched and unbranched), and flexibility (saturated and unsaturated) of the moieties at position 5 of the phenyl ring. We maintained a certain heterogeneity, considering that subtle differences in the chemical structure could result in non-negligible variations of the antimycobacterial activity. With these premises, we selected the chains reported in Figure 3 and synthesized the corresponding compounds.

### Synthesis

**Scheme 1:**
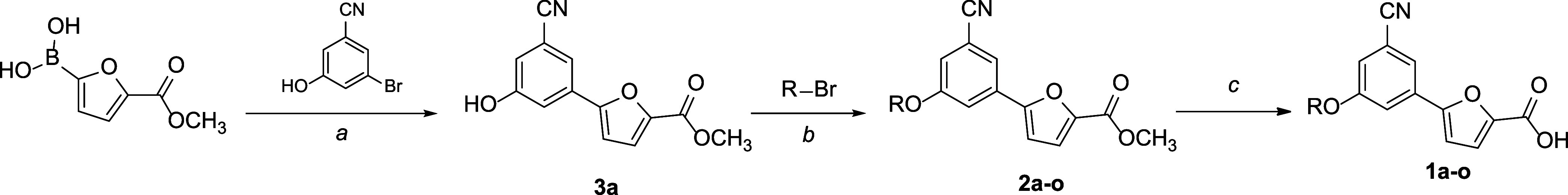
Reagents and Conditions: (a) Pd(PPh_3_)_2_Cl_2_, 2 M Na_2_CO_3_, 1,4-Dioxane, 60 °C, 80 min, MW, N_2_ atm.; (b) K_2_CO_3_, Acetone, Reflux, 30 min–48 h, N_2_ atm.; (c) LiOH·H_2_O, THF/H_2_O 2:1, RT, 2–5 h For the definition of R groups, see Table 1.

**Scheme 2:**
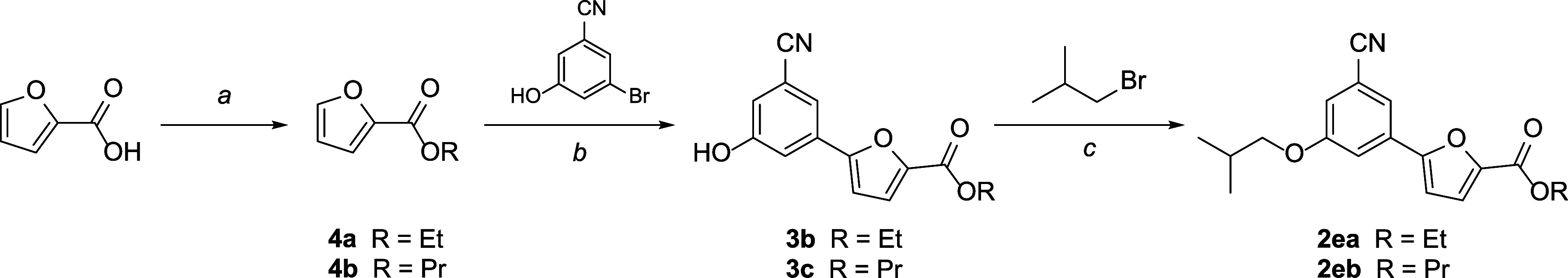
Reagents and Conditions: (a) ROH, conc. H_2_SO_4_, reflux, 24 h; (b) Pd(OAc)_2_, KOAc, DMA, 125 °C, 30 min, MW, Ar atm.; (c) K_2_CO_3_, Acetone, Reflux, 48 h, N_2_ atm

Compounds **1a**–**o** (Table 1) were synthesized following a previously published procedure, as reported in Scheme 1.^40^ Briefly, a Suzuki–Miyaura cross-coupling between (5-(methoxycarbonyl)furan-2-yl)boronic acid^38^ and 3-bromo-5-hydroxybenzonitrile afforded the key intermediate **3a**, which underwent a Williamson ether synthesis with a suitable bromo-derivative to give compounds **2a**–**o**. Then, the ester moiety was cleaved by conventional basic hydrolysis to afford the final acids **1a**–**o**. The ethyl and propyl ester derivatives of **1e** were obtained by a different approach (Scheme 2) due to the cumbersome isolation of the ethyl and propyl ester analogs of the starting boronic acid. The alternative route involved the esterification of 2-furoic acid with the proper alcohol (ethanol or *n*-propanol), followed by a palladium-catalyzed direct arylation with 3-bromo-5-hydroxybenzonitrile, leading to **3b**,**c**. The latter were then reacted with 1-bromo-2-methylpropane to give the ethyl and propyl esters **2ea** and **2eb**, respectively. The main synthetic steps are illustrated below; further synthetic details and the most relevant spectra are reported in the Supporting Information.

**Table 1:**
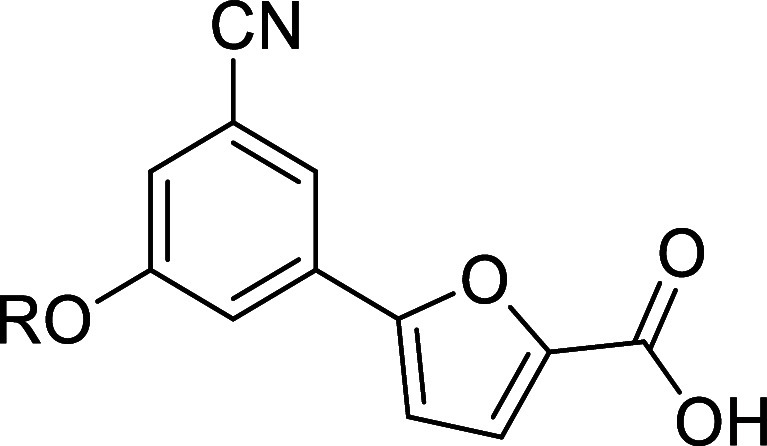

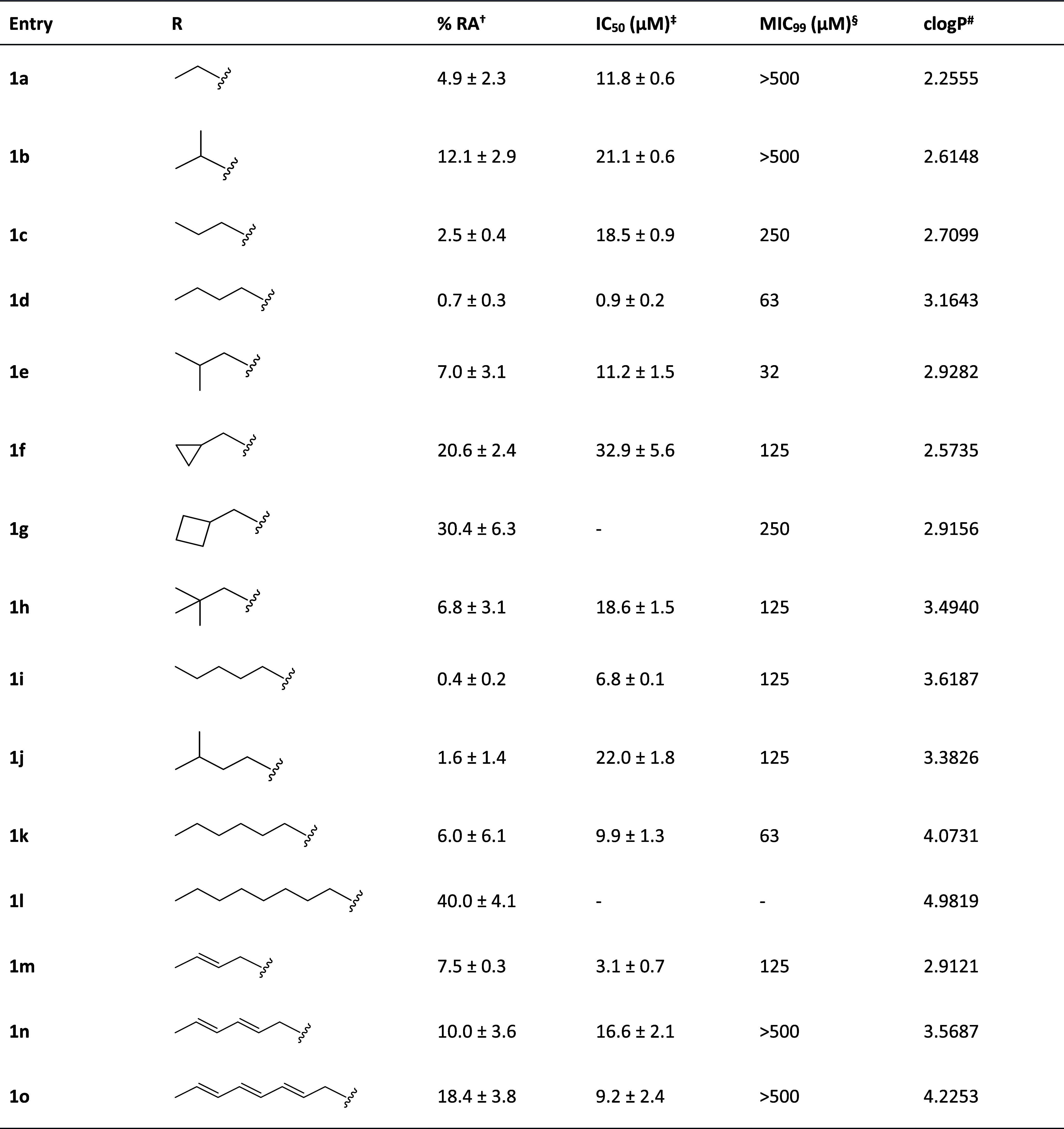
*In Vitro* Activity (%RA, IC_50_, and MIC_99_) of the Candidate MbtI Inhibitors **1a**–**o^a^**

a ^*^For each compound, the calculated partition coefficient (clogP) is also shown. ^†^Percentage of residual enzymatic activity at 100 μM. ^‡^IC_50_ values were calculated for compounds showing a %RA < 20. ^§^MIC_99_ were determined against *M. bovis* BCG. Values were calculated for compounds showing a %RA < 20. ^#^clogP values were computed by using the DataWarrior software.^55^

### Crystallography

**Figure 4:**
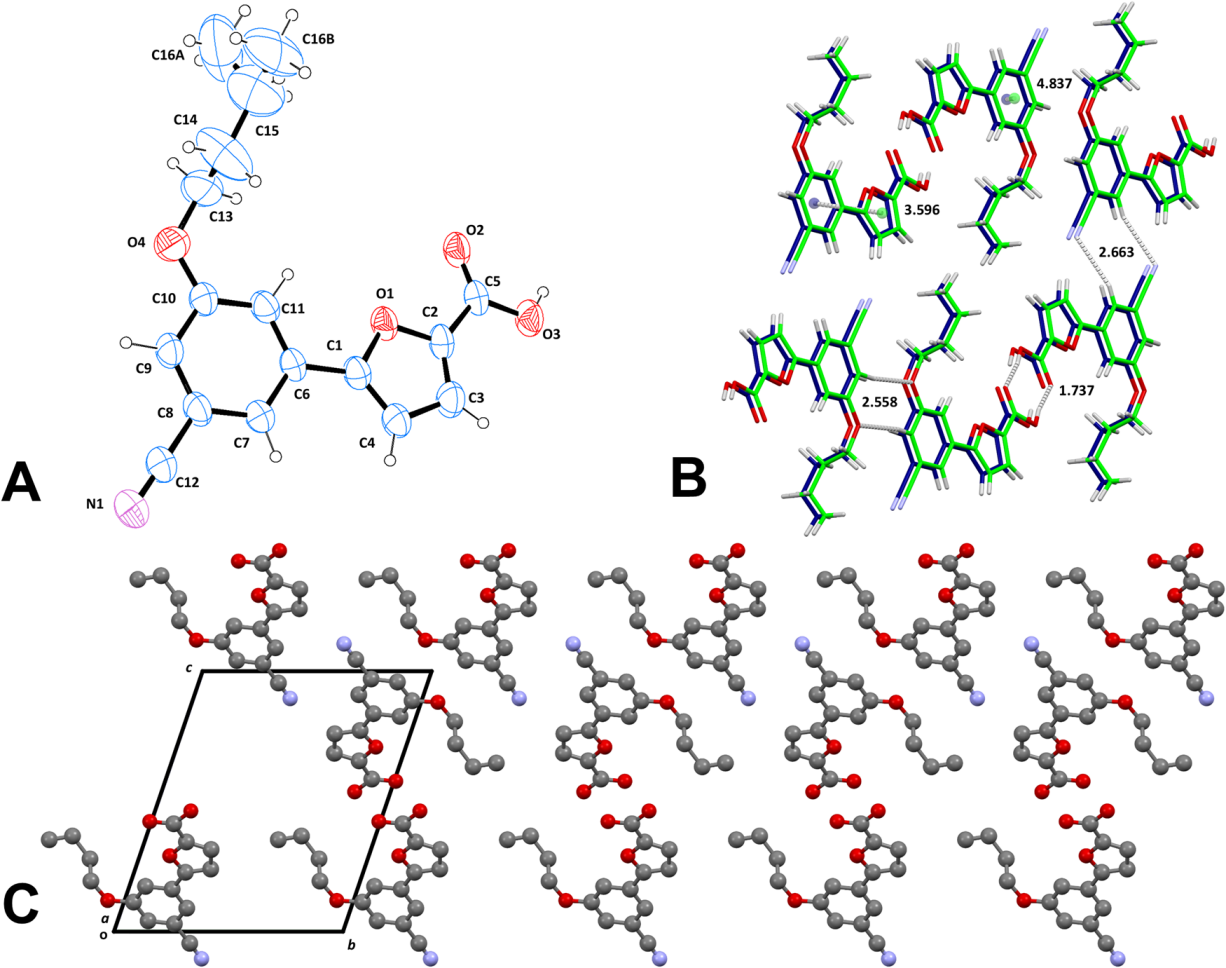
(A) ORTEP diagram of 1d, reporting the atom numbering scheme. Thermal ellipsoids are set at the 30% probability level. (B) Stick model showing the main intermolecular interactions, in an arbitrary orientation. One of the disordered methyl groups is omitted. (C) Crystal packing viewed along the *a* axis. Molecules are represented as ball-and-stick models; hydrogen atoms and one of the disordered methyl groups are omitted for the sake of clarity.

For derivative **1d**, the most potent MbtI inhibitor of the series (see Biological Evaluation), we investigated the molecular structure by single-crystal X-ray diffraction (SC-XRD). This analysis allowed us to determine its identity and examine its three-dimensional features. Intensity data showed that the compound crystallized in the triclinic space group P-1, with one molecule in the asymmetric unit. The ORTEP diagram,^53^ with the arbitrary atom numbering scheme, is depicted in Figure 4A. The molecule has a nearly planar conformation, with the phenyl group inclined at about 9.5° to the plane of the 2-furoic acid moiety. The lateral chain forms a dihedral angle of −93.3(7)° with the aromatic core (C10–O4–C13-C14). Due to its high mobility, the whole alkyl ether portion is disordered, with the terminal methyl group split into two positions C16A/C16B, characterized by a refined occupancy of 65 and 35%, respectively. Bond lengths and angles are within the expected parameters.^54^ The crystal packing is consolidated by intermolecular H-bonds between the carboxylic groups of two neighboring molecules, which form dimers along the *a* axis (D···H/Å = 0.86(4); D···A/Å = 2.65(1); H···A/Å = 1.79(4); D-H···A/° = 170(4)). Different dimeric units are further connected by nontraditional H-bonds between the ether oxygen and the aryl C7–H7 of an adjacent molecule (D···H/Å = 0.97(4); D···A/Å = 3.52(1); H···A/Å = 2.56(4); D-H···A/° = 171(3)). Similar, long-range H-bonds, established between the nitrile group and C4–H4, bridge dimer chains along the *b* axis (D···H/Å = 0.91(4); D···A/Å = 3.51(1); H···A/Å = 2.66(4); D-H···A/° = 154(3)). Heteroaromatic π–π stacking interactions between the phenyl group and the furan (centroid-centroid distance: 3.60 Å) stabilize the structure. The phenyl moieties also form weak offset interactions (centroid-centroid distance: 4.84 Å; offset angle: ∼45.8°), with a marginal influence on the overall assembly. Graphical depictions of the most important contacts and the crystal packing are shown in Figure 4B,C, respectively.

**Figure 5:**
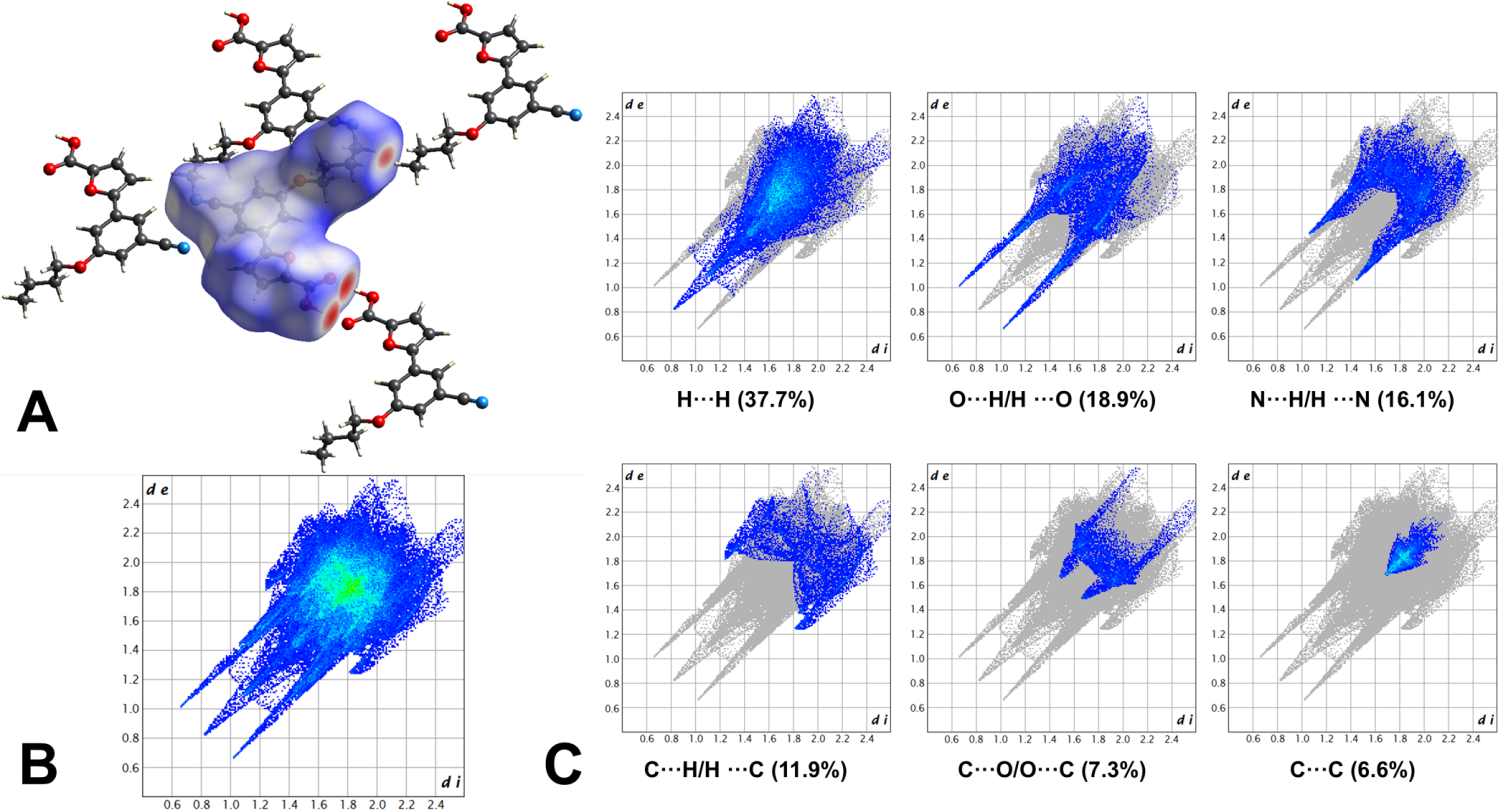
(A) HS of 1d mapped over *d*_norm_ with a fixed color scale in the range −0.7259 au (red) to 1.4013 au (blue). Red, blue, and white indicate intermolecular contacts shorter, longer, and approximately equal to the sum of their van der Waals radii. (B) 2D fingerprint plot of **1d**, providing a visual summary of the frequency of each combination of *d*_*e*_ and *d*_*i*_ across the HS. Points contributing to the Hirshfeld surface are colored from blue (lower contribution) to green (greater contribution), based on the extent of their contribution to the surface area. (C) Main 2D fingerprint plots of **1d**, specifying the contribution of each contact type. The least represented contacts are not shown.

The intermolecular network was also investigated by analyzing the Hirshfeld surface (HS) of **1d** (Figure 5). For this study, the disorder of the alkyl chain was purposely left unrefined to avoid artifacts due to the split position of the methyl group. The HS (*V* = 362.26 Å^3^; A = 343.39 Å^2^; *G* = 0.716; Ω = 0.132) was mapped over the normalized contact distance (*d*_norm_) using a red-white-blue color scheme based on the length of the intermolecular contact relative to the sum of the van der Waals radii of the atoms involved. The visual inspection of the surface revealed two intense red spots corresponding to the short-range H-bond between the carboxylic acids. Other, fainter red areas confirmed the presence of long-range nontraditional H-bonds consolidating the contacts between adjacent dimeric units. Another significant spot appeared on the terminal portion of the lateral chain, indicating a short contact between the alkyl substituents of neighboring molecules. This observation suggested the presence of additional van der Waals forces in the stabilization of the packing. The 2D fingerprint plots, describing the contribution of each contact type and the relative area of the surface corresponding to it, corroborated the importance of O···H/H···O contacts. In detail, the long spikes in the lower left corner of the graph confirmed the presence of short-range H-bonds between the carboxylic acids. In the middle, a third elongated region (H···H) highlighted the rather strong hydrophobic forces between the alkyl chains. The most external, shorter spikes (N···H/H···N) represented the long-range H-bonds between the nitrile and the aryl CH. Finally, a typical arrow-shaped area in the center of the plot, representing C···C contacts, confirmed the presence of π–π interactions. These observations were verified by the calculation of the enrichment ratios, which showed that O···H/H···O, N···H/H···N, O···C/C···O, C···C, and H···H contacts were enriched.

### Biological Evaluation

The biological evaluation of the new derivatives **1a**–**o** began with assessing their inhibitory effect on MbtI (Table 1). The calculation of the residual activity (%RA) at 100 μM revealed that all compounds exhibited a modest-to-excellent activity on the enzyme. Most notably, the *n*-butoxy analog **1d** reduced the enzymatic activity to less than 1%, corresponding to an IC_50_ value of 0.9 μM; hence, this compound emerged as the most potent inhibitor across the entire series. Shorter (**1a,c**) and longer (**1i,k**) linear alkyl chains generally led to higher IC_50_ values compared to **1d** (6.8 ≤ IC_50_ ≤ 18.5 μM). Branched chains (**1b,e,h,j**) also determined a reduction in the inhibitory effect relative to the best member of the series (11.2 ≤ IC_50_ ≤ 22.0 μM), with the most significant decrease observed for the longer isopentyloxy derivative (**1j**). Overall, rigidified analogs showed higher IC_50_ values compared to the more flexible congeners featuring carbon chains of the same length. This effect was modest when unsaturations were introduced (**1m**–**o**), with IC_50_ values of 3.1–16.6 μM. Surprisingly, the 2,4,6-octatrienyloxy derivative (**1o**) was found to be more active than its saturated analog (**1l**), which did not exert a significant inhibitory effect on the enzyme (40% RA). The loss of inhibition was much more prominent with more constrained, cyclic derivatives. Despite the length of the chain remaining substantially unchanged, the cyclopropyl (**1f**) and cyclobutyl (**1g**) moieties led to a drastic increase of the %RA (20 and 30%, respectively), suggesting that the rise in bulkiness and associated loss of structural freedom was detrimental for the activity. Overall, we can conclude that the selected substituents at position 5 of the phenyl ring can enhance the inhibitory effect of the compounds on MbtI, but only if certain steric and mobility requirements are met. In detail, lateral chains with 4–5 carbon atoms are preferred; branching and unsaturation are tolerated only to some extent, ideally with short substituents.

The assessment of the *in vitro* antimycobacterial activity of this new series (**1a**–**o**) was carried out in iron-limiting conditions (chelated Sauton’s medium) against the nonpathogenic *M. bovis* BCG, using the REMA method (Table 1). Measuring the MIC_99_ values of potential antitubercular agents is essential, as substantial differences can occur even within series exhibiting minimal structural variability. This parameter is crucial for identifying potent antimycobacterial agents and optimizing candidate compounds, thereby guiding subsequent development efforts. Furthermore, the Universal CAS assay was employed to ascertain the inhibition of siderophore production. More detailed information is available in the Experimental section. Overall, our design strategy led to the discovery of analogs that significantly lowered the MIC_99_ value compared to the parent compound **I**. Aside from the variability typical of microbiological assays, we observed that the increase in lipophilicity of the molecules was positively linked to a decrease in the MIC_99_. However, excessive steric hindrance was detrimental to the potency (**1g**), probably due to the more difficult penetration of the cell wall. This effect was particularly evident for **1n,o**, in which the rigidification of a long, hindered chain (6–8 carbon atoms) completely abolished the antimycobacterial effect. The most potent candidate was **1e**, with an MIC_99_ of 32 μM; promising results were also obtained with **1d,k**, which matched the potency of **II** (63 μM). Time-killing assays were performed for **1e**, demonstrating the bacteriostatic activity of the compound in the conditions tested (Figure S106). Despite not being the best MbtI inhibitor, **1e** retained a more than satisfactory IC_50_ (11.2 μM), comparable to the previous lead **II**. The CAS assay confirmed that **1e** acted by impairing the production of siderophores, validating its mechanism of action, and corroborating the link between its antimycobacterial effect and the inhibition of MbtI (Figure S106). The same experiment was repeated for other compounds (such as **1d,k**), with similar results, further supporting the use of this scaffold for developing a selective antivirulence therapy.

Efflux pumps play a crucial role in the development of resistance by actively pumping out antimicrobial agents from bacterial cells, thereby reducing the intracellular concentration of the drug and limiting its effectiveness.^56^ Specifically, the *Mtb* genome encodes a diverse array of efflux transporters, with over 30 pumps from five major superfamilies, including the ATP-binding cassette (ABC) and major facilitator superfamily (MFS), implicated in drug resistance.^57^ Therefore, to verify whether our compounds could be substrates of mycobacterial efflux pumps, the MIC_99_ of **1d,e,k** was determined in the presence of the known efflux pump inhibitors reserpine and verapamil, each at a concentration of 1/2 MIC_99_. Most notably, our results indicated that the tested compounds were not substrates of efflux pumps, as their effectiveness remained unaffected by the inhibition of these transporters (Figure S107). This also suggests that our inhibitors could lead to effective treatments with a reduced potential for resistance development.

### Molecular Modeling

Compound **1e**, which proved to be the most active candidate in the assay against *M*. *bovis* BCG, was analyzed by molecular modeling studies using the crystal structure of MbtI in complex with **I** (PDB code: 6ZA4).^38^ This structure was chosen as the starting model for the computational investigations, as compound **I** had previously been identified as a potent inhibitor, making its crystal complex an ideal foundation for further *in silico* exploration.^58^ Despite the availability of high-resolution structural data, the G270-G277 loop region was partially absent from the original crystal structure. Given the potential importance of this region in ligand binding and overall protein dynamics, the missing loop was reconstructed computationally using the homology modeling software Modeler.^59^ The reintroduction of this loop was essential to produce a fully functional model that could accurately represent the conformational behavior of the protein in subsequent simulations. After generating the complete model, the resulting protein–ligand complex underwent energy minimization to refine the system. It was subsequently subjected to an extensive 551 ns molecular dynamics (MD) simulation to ensure conformational stability.

**Figure 6:**
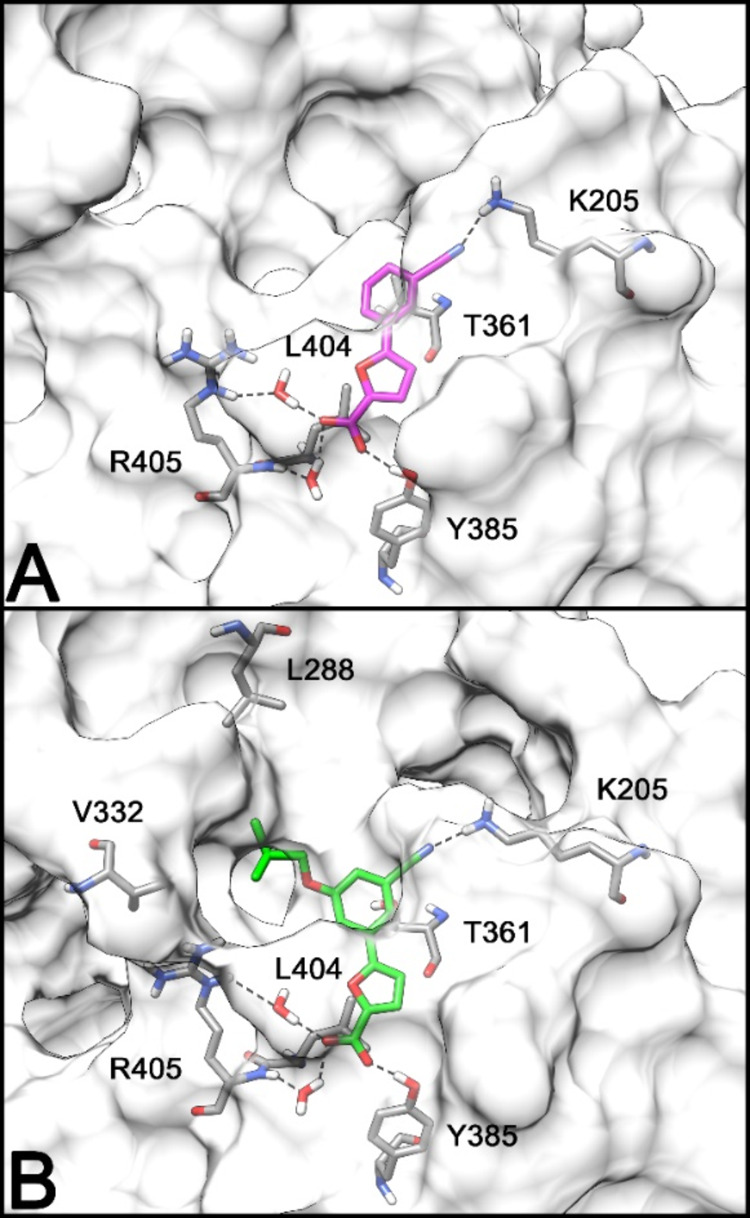
Minimized average structure of **I** (**A**) and **1e** (**B**) within the MbtI binding site.

Upon completion of the MD simulation, the average structure of the protein, representing the predominant conformation over the trajectory, was extracted for use in docking studies with compound **1e**. The docking results produced an initial MbtI-**1e** complex, which was then processed following the same minimization and MD simulation protocol applied to the original complex with **I**. As illustrated in Figure 6A, the carboxyl group of the ligand (**I**) maintains a key hydrogen bonding interaction with Tyr385, along with two water-mediated interactions with Arg405. The phenyl ring engages in hydrophobic contact with Thr361, while the cyano group forms an additional hydrogen bond with Lys205.

The structural modification introduced in compound **1e** involved adding a 5-isobutoxy group to the phenyl ring. The introduction of this group resulted in a noticeable outward movement of the G270-G277 loop region and this outward shift, as shown in Figure S109, is likely driven by the steric hindrance introduced by the isobutoxy group, which creates a physical clash with the loop in its original position. Interestingly, although the isobutyl moiety is partially exposed to the solvent, it forms favorable hydrophobic interactions with nearby residues Leu288 and Val332, as depicted in Figure 6B. These hydrophobic contacts help stabilize the ligand in the binding pocket, compensating for any potential loss of binding affinity due to the steric clash and solvent exposure. This finding is critical, as it supports the hypothesis that the addition of bulky, lipophilic groups can be tolerated in the binding site, leading to comparable biological activity between compound **1e** and the original derivative (**I**).

### Activity of **1e** on an Infected Macrophage Model

Considering that alveolar macrophages (AMs) are the major targets of *Mtb* in the human host,^60^ we tested the new lead compound **1e** on murine-derived Max Planck Institute 2 (MPI-2) cells. MPIs are functionally close to lung AMs and replicate the mycobacterial–host crosstalk accurately.^61^ Unfortunately, **1e** did not exhibit significant activity on infected macrophages, suggesting possible issues in crossing the eukaryotic membrane. Therefore, we hypothesized that utilizing a nanocarrier to facilitate the crossing of the plasma membrane would be an ideal strategy to enhance the intracellular uptake and therapeutic efficacy of **1e**, as observed in other cases.^62,63^ Furthermore, this approach would allow for the selection of an appropriate delivery system and increase the selectivity of payload release to specific cell types, namely AMs. This would further improve the compound’s efficacy by ensuring it reached its target efficiently, minimizing potential side effects.

### Encapsulation Experiments

To promote the accumulation of **1e** within the cytoplasm of our *in vitro* cellular model, we encapsulated the compound in a polymeric-based nanocarrier (*i.e*., polymersomes or POs). In this regard, previous studies have demonstrated that PO formulations based on poly(2-methacryloyloxyethyl phosphorylcholine)-poly(2-(diisopropylamino)ethyl methacrylate) (PMPC–PDPA) can selectively deliver antitubercular agents to macrophages. These POs increase safety and therapeutic efficiency while limiting off-target effects.^48^ Moreover, PMPC–PDPA POs are known to selectively target the scavenger receptor B type I (SR-B1), CD36, and CD81, all highly expressed in monocytes, dendritic cells, and macrophages.^64^ However, our attempts to load **1e** into POs resulted in a very low encapsulation efficiency. This was most likely due to the physicochemical properties of the compound, which were not well-suited for effective internalization into POs. Specifically, the intermediate characteristics of **1e**, falling between those of hydrophilic and hydrophobic molecules, prevented its efficient encapsulation in either the core or the lipid bilayer. Therefore, we prepared the ethyl and propyl ester derivatives of **1e** (**2ea** and **2eb**, respectively) to increase the overall lipophilicity of the molecule and improve the compound’s ability to interact with and be encapsulated within the hydrophobic lipid bilayer of the POs (Table 2). The rationale was that eukaryotic or mycobacterial esterases would convert the esters to their active acid form. Indeed, plenty of literature suggests that the human carboxylesterase 1 (CES1), a ubiquitous serine esterase that processes substrates containing bulky acyl moieties and small alcoholic groups, should efficiently cleave the ester bond in these derivatives. For example, CES1 has been reported to be responsible for the activation of several ester prodrugs, such as clopidogrel, oseltamivir, enalapril, *etc*.^65,66^ Before proceeding with the encapsulation of **2ea** and **2eb**, we evaluated their antimycobacterial activity against *M*. *bovis* BCG. The resulting MIC_99_ values were 64 and 125 μM, respectively (Table 2). Although the esters themselves were not active against MbtI (RAs > 70%, Table 2), these MIC_99_ values suggested that mycobacterial enzymes hydrolyzed the compounds into their active acid form. To further confirm hydrolysis, we lysed the mycobacterial cells and analyzed the supernatant by mass spectrometry. The spectra of the lysed cells sample displayed a peak at *m*/*z* = 284.23, corresponding to the acid form of **1e** (exact mass = 285.10; Figure S108). This outcome indicated that *M*. *bovis* BCG, which was used as a model, expresses enzymes capable of hydrolyzing esters to their corresponding acids, thus confirming previous findings by other research groups.^67,68^ These results demonstrated the potential of **2ea** and **2eb** to be converted into their active forms within mycobacterial cells, thus enhancing their therapeutic efficacy.

**Table 2:**
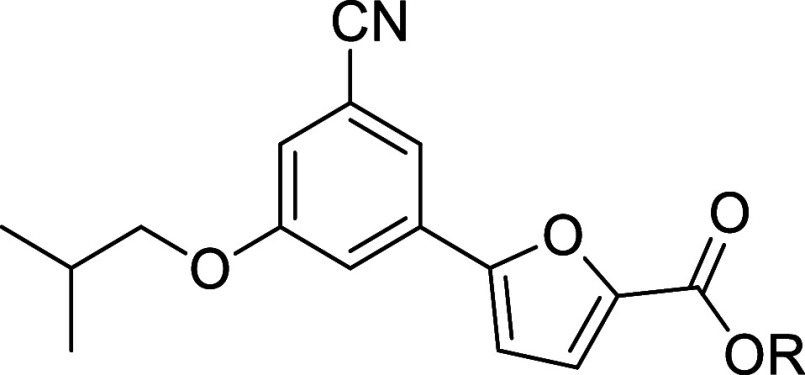

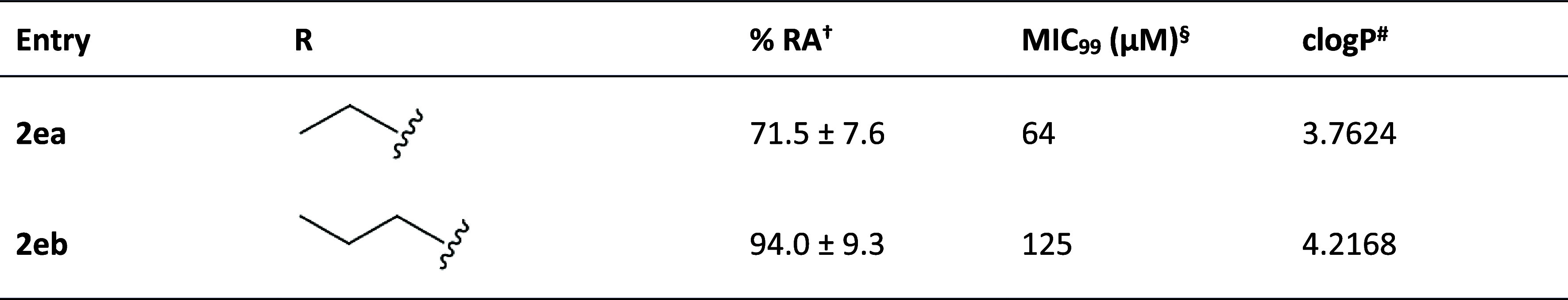
*In Vitro* Activity (%RA and MIC_99_) of **2ea** and **2eb**^a^

a ^†^Percentage of residual enzymatic activity at 100 μM. ^§^MIC_99_ were determined against *M*. *bovis* BCG. ^#^clogP values were computed by using the software DataWarrior.^55^

With the support of these experimental findings, we proceeded with the encapsulation of **2ea** and **2eb** into the selected POs. Notably, our attempt to increase the lipophilicity of the molecules significantly increased the encapsulation efficiency, as revealed by HPLC analysis (34.2 and 60.1% for **2ea** and **2eb**, respectively; Table 3, Figure S110). Dynamic Light Scattering (DLS) and Transmission Electron Microscopy (TEM) analyses confirmed the presence of spherical POs with homogeneous shape distributions and an average size of 44.46 ± 9.58 nm and 45.68 ± 1.86 nm for **2ea**- and **2eb**-loaded POs, respectively (Table 3, Figures S111 and S112). The encapsulation of the compounds did not alter the shape or size distribution (average size of the empty POs calculated from three independent batches: 41.78 ± 5.03 nm) of the POs, indicating that the process was compatible with the structural integrity and consistency of the nanocarriers. Furthermore, TEM analysis of the empty and drug-loaded POs 18 months after the production process confirmed the long-term stability of the formulations, ensuring their effectiveness over extended periods (Figure S112).

**Table 3:** Encapsulation Efficiency, Drug Loading, and Hydrodynamic Diameter of Drug-Containing POs^a^

drug	initial drug conc. (mg/mL)	encapsulated drug conc. (mg/mL)	encapsulation efficiency (%)^b^	drug loading (%)^c^	average size (nm)
**2ea**	2.00	0.20 ± 0.05	34.20 ± 0.21	5.24 ± 1.04	44.46 ± 9.58
**2eb**	2.00	0.40 ± 0.01	60.13 ± 2.14	7.14 ± 1.61	45.68 ± 1.86

a Data are compiled from three independent batches and is expressed as mean ± SD.

b Encapsulation efficiency was calculated as (encapsulated mass of the drug)/(initial mass of the drug) × 100.

c Drug loading was calculated as (encapsulated mass of the drug)/(mass of drug-loaded nanoparticles) × 100.

### Cytotoxicity Assays and Activity on Mtb-Infected Murine Lung Macrophages (mAMs)

**Figure 7:**
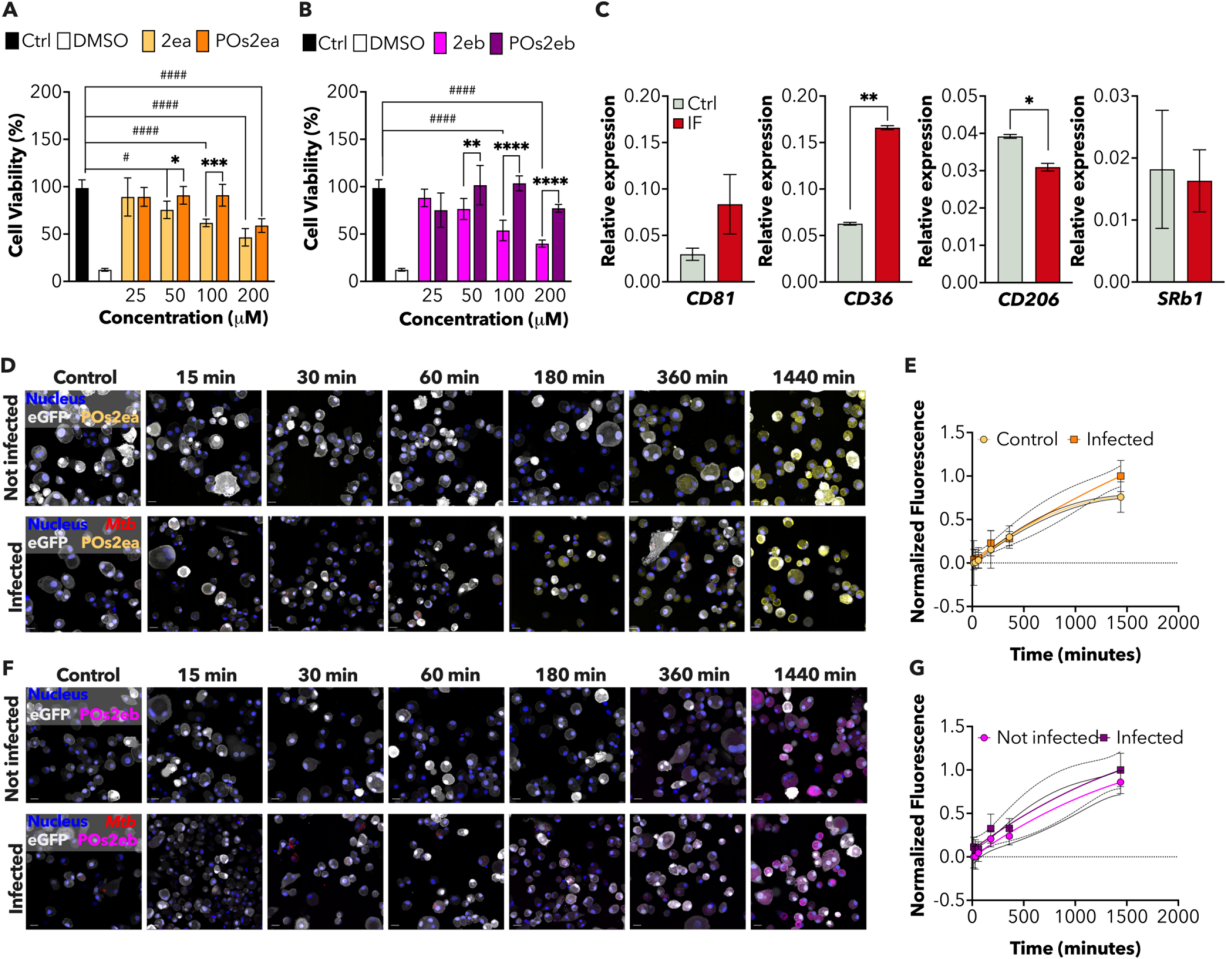
Investigation of the antitubercular activity of **POs2ea** and **POs2eb** on a mAM-like cellular model. (A and B) MTT viability assay was performed on mAM-like cells after a 24-h exposure to 200, 100, 50, and 25 μM of either free or encapsulated (POs) **2ea** (A) or **2eb** (B), as listed in the color-coded legend. 10% DMSO was used as the negative control. Data are shown from four independent experiments. Mean values ± SD are plotted (*n* = 4). Significance by two-way ANOVA followed by Šidàk’s multiple comparisons: #*p* = 0.0177, ###*p* = 0.0004, ####*p* < 0.0001, *p = 0.033, **p = 0.0015, ***p = 0.0002, ****p < 0.0001. (C) qRT-PCR analysis comparing the expression of selected receptors mediating PMPC–PDPA cellular internalization in steady-state conditions (light gray) and after *Mtb* infection (red). Transcripts are normalized to total RNA and GAPDH expression. Data are shown from two independent experiments. Mean values ± SD are plotted (*n* = 2). Significance by paired *t* test: *p = 0.0285, **p = 0.0030. (D and F) Representative snapshots evaluating Cy5–PMPC-PDPA-Et (**POs2ea**) (D) and Cy5 PMPC–PDPA-Pr (**POs2eb**) (F) uptake in either infected (lower panel) or noninfected (upper panel) cells, with fluorescent *Mtb* reporter at different points in time. **POs2ea** (yellow), **POs2eb** (magenta), nuclei (blue), eGFP (gray), and *Mtb* (red) fluorescence are merged and indicated in the legend. The measured time points are indicated in the legend. Scale bar 20 μm. (E and G) Plots showing intracellular Cy5–PMPC-PDPA-Et (**POs2ea**) (E) and Cy5 PMPC–PDPA-Pr (**POs2eb**) (G) in mAMs. Fluorescence intensity is normalized over time in steady-state conditions (yellow and magenta circles) or after *Mtb* infection (orange and purple squares). Yellow circles and orange squares (**E**) indicate Cy5–PMPC-PDPA-Et (**POs2ea**) normalized mean intensity at the measured time points. Dotted and full lines represent SD. Data are from two independent experiments. Magenta circles and purple squares (G) show Cy5-PMPC-PDPA-Pr (**POs2eb**) normalized mean intensity at the measured time points. Light magenta and light purple shadows represent SD. Data are from two independent experiments.

The selection of PMPC–PDPA POs was also supported by their well-established safety profile in clinical applications and a high degree of biocompatibility.^48^ We validated the biosafety of our formulation by conducting cytotoxicity assays on the human leukemia monocytic cell line THP-1, a widely used model for assessing macrophage activity modulation, and on Human Lung Fibroblasts (HLF), an excellent model for studying various aspects of human pulmonary function and pathophysiology. Viability assays confirmed that both the pristine POs and the POs loaded with the selected MbtI inhibitors (**2ea** and **2eb**) did not affect the metabolic activity of THP-1 and HLF cells, even at drug concentrations up to 100 μM and polymer concentrations up to 30 μM (Figure S113). These positive results highlighted the excellent biosafety of our formulations, ensuring their suitability for therapeutic applications. Then, we checked whether PMPC–PDPA POs loaded with either **2ea** or **2eb** showed a similar biocompatibility profile in our *in vitro* cellular model, namely mAMs.^69^ Hence, we treated mAMs with 2-fold decreasing concentrations of both the free compounds (**2ea** and **2eb**) and the encapsulated formulations (**POs2ea** and **POs2eb**), and then we measured mAM viability after 24 h (Figure 7A,B). By measuring cell metabolic activity, we observed that while **2ea** and **2eb** caused an increase in mAM mortality at the two highest tested concentrations (200 and 100 μM), **POs2ea** and **POs2eb** cytotoxic effect was only observed at 200 μM. This result supported previous evidence that encapsulation in POs can enhance drug biosafety, highlighting the positive impact of encapsulation on reducing cytotoxicity at therapeutic concentrations.^70,71^

Macrophages provide an intracellular niche for the proliferation, spread, and survival of *Mtb*. Therefore, intracellular targeting of antibiotics to these cells is a crucial strategy for treating TB in humans. In this context, there is extensive published evidence that POs are taken up rapidly and efficiently by macrophages.^48^ Considering that the uptake of PMPC–PDPA POs is strictly related to the phenotype and density of specific surface receptors (CD81, CD206, CD36, and SRb1),^64^ we investigated whether intracellular pathogens might affect the expression of these surface markers. The rationale was that an infectious state could modulate the expression of the receptors, thereby altering the dynamics of PO uptake. Interestingly, gene expression analysis demonstrated a significant upregulation of all the receptors involved in PMPC–PDPA targeting at 24 h postinfection (Figure 7C). This increase was correlated with enhanced uptake of POs, further demonstrating that infection-induced receptor modulation positively affects the efficiency of PO-mediated drug delivery. In detail, we incubated healthy or *Mtb*-infected mAMs with equal concentrations of fluorescently labeled POs (Cy5) loaded with **2ea** (Cy5-**POs2ea**; Figure 7D,E) or **2eb** (Cy5-**POs2eb**; Figure 7F,G) for up to 24 h. As expected, confocal microscopy quantification of Cy5-**POs2ea** and Cy5-**POs2eb** confirmed that healthy and infected mAMs effectively internalized the POs, regardless of the encapsulated payload (Figure 7E,G). Additionally, PO uptake increased over time without any sign of saturation. Although the upregulation of receptors in infected cells corresponded to enhanced internalization of POs, the increase was not statistically significant. These findings suggest that while infection-induced receptor upregulation generally improves PO uptake, the effect might vary, requiring further investigation to fully elucidate the underlying dynamics (Figure 7E,G).

**Figure 8:**
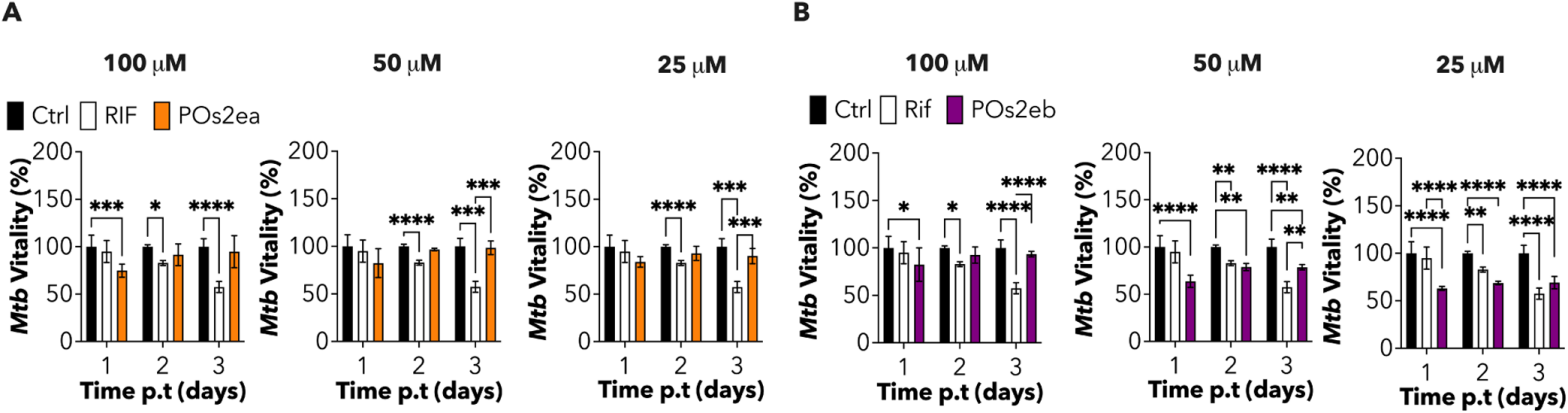
(A and B) Spectrofluorometer (plate reader) measure of *Mtb* fluorescence after infection and treatment with encapsulated **2ea** (**POs2ea**, A) and **2eb** (**POs2eb**, B) at decreasing concentrations over time. Control (Ctrl) indicates infected and untreated cells. Rifampicin (Rif, 1 μg/mL) was used as the positive control. Rif and encapsulated compounds are indicated in the legends, and measured time points are indicated. Black lines represent mean ± SD. Data are from four independent experiments. Significance by two-way ANOVA followed by Tukey’s multiple comparison test: *p = 0.02, **p = 0.0053, ***p = 0.0003, ****p < 0.00001.

Subsequently, we assessed the therapeutic efficacy of **POs2ea** and **POs2eb** in reducing *Mtb* infection. To study their effect, we employed an *Mtb* strain constitutively expressing a fluorescent protein (*i.e*., TdTomato), which can be exploited to quantify *Mtb* intracellular load. We infected mAMs with *Mtb* for 4 h, removed the extracellular bacilli by thorough washes, and then treated the cells with decreasing concentrations of POs, loaded with the two compounds (**POs2ea** and **POs2eb**; Figure 8A,B). The first-line antitubercular drug Rifampicin was used as a positive control (2x MIC, 1 μg/mL). Considering the slow dynamic of *Mtb* infection, we decided to quantify the variation in *Mtb* burden by measuring *Mtb* fluorescence at 1-, 2- or 3-day post-treatment (p.t) (Figure 8A,B). Spectrofluorometer-based quantifications showed that **POs2ea** and **POs2eb** induced a moderate-to-good decrease in *Mtb* fluorescence at all tested concentrations, 24 h (*i.e*., 1 day) post-treatment (Figure 8A,B). These findings highlighted the potential efficacy of the encapsulated drugs in rapidly reducing *Mtb* infection within a short period of time. This effect was in stark contrast to that of Rifampicin, which reached its peak effect only 3-day post-treatment. Moreover, while **POs2ea** approached the maximum effect of Rifampicin (3-day post-treatment) only at the highest tested concentration (100 μM), **POs2eb** consistently reduced *Mtb* load comparably to the reference drug at all tested concentrations. The observed reduction in effect over time (*i.e*., day 2 and 3 post-treatment) may be attributed to the rapid action of **2ea** and **2eb** on mycobacteria. This suggests that tailored interventions to achieve an optimized activity profile could effectively mitigate *Mtb* virulence.

## Conclusions

In this study, we rationally designed new inhibitors of MbtI, the first enzyme involved in siderophore production. We synthesized a library of 5-(3-cyanophenyl)furan-2-carboxylic acid analogs bearing alkyl and alkenyl chains at the 5-position of the phenyl ring. These compounds maintained an excellent inhibitory potency on the target enzyme, promoting iron deprivation while displaying significantly improved antimycobacterial activities. The most promising candidate (**1e**) was selected to explore the use of a specific delivery system based on PMPC–PDPA POs to increase its selective internalization in macrophages, thereby limiting off-target effects and enhancing efficacy. We successfully loaded POs with **2ea** and **2eb**, two ester derivatives of **1e**, designed to increase the parent molecule’s hydrophobicity and improve the nanocarriers’ internalization. The nanoparticles (**POs2ea** and **POs2eb**) crossed the macrophage membrane and delivered the payload to the intracellular mycobacteria, resulting in a moderate-to-good reduction of *Mtb* vitality, especially 1-day post-treatment. Our results provide evidence that optimized MbtI inhibitors may become integral components of the anti-TB therapeutic arsenal. Additionally, our findings confirm that simple-to-produce PO formulations of antimicrobial agents represent a viable approach for clinical development. With further refinement, these approaches hold great promise to make a significant impact in advancing TB treatment strategies.

## Experimental Section

### Chemistry

All starting chemicals and solvents were purchased from commercial suppliers (Sigma-Aldrich, Merck KGaA, Darmstadt, Germany; FluoroChem, Hadfield, UK) and used as received. The course of the reactions was monitored by thin-layer chromatography (TLC) using aluminum-backed Silica Gel 60 plates (0.2 mm; Merck, Darmstadt, Germany). Microwave-assisted reactions were carried out with a Biotage Initiator Classic (Biotage, Uppsala, Sweden). Crude products were purified by flash column chromatography on silica gel 60 (40–63 μM; Merck) using the indicated solvent system. All tested compounds were characterized by mono- and bidimensional NMR techniques (where appropriate), FT-IR, and ESI-MS. ^1^H and ^13^C NMR spectra were acquired at ambient temperature with a Varian Oxford 300 MHz instrument (Varian, Palo Alto, CA) or a Bruker Avance 300 MHz instrument (Bruker, Billerica, MA), operating at 300 MHz for ^1^H and 75 MHz for ^13^C. 2D NMR experiments were performed on a Bruker Avance Neo 400 MHz spectrometer. Chemical shifts are expressed in ppm (δ), and *J*-couplings are given in Hertz. ATR-FT-IR spectra were acquired with a PerkinElmer Spectrum One FT-IR (PerkinElmer, Waltham, MA), equipped with a PerkinElmer Universal ATR sampling accessory consisting of a diamond crystal. Analyses were performed in a spectral region between 4000 and 650 cm^–1^ and analyzed by transmittance technique with 28 scans and 4 cm^–1^ resolution. MS analyses were carried out with a ThermoFisher LCQ Fleet system (ThermoFisher, Waltham, MA), equipped with an ESI electrospray ionization source and an Ion Trap mass analyzer; ionization: ESI positive or ESI negative; capillary temperature: 250 °C; source voltage: 5.50 kV; source current: 4.00 μA; multipole 1 and 2 offset: −5.50 and −7.50 V, respectively; intermultipole lens voltage: −16.00 V; trap DC offset voltage: −10.00 V. The purity (≥95%) of the tested compounds was assessed by RP-HPLC using a Waters system (Waters, Milford, MA), equipped with a Phenomenex Luna 3 μM C18(2) 100 Å, 100 mm × 4.6 mm column (Phenomenex, Torrance, CA) and operating in the following conditions: mobile phase: 30:70 water/methanol (20:80 water/methanol for **2ea** and **2eb**) + 0.05% trifluoracetic acid (TFA) in isocratic mode; flow rate: 1 mL/min; detector λ: 254 nm; time: 30 min; temperature: 23 °C. The main synthetic steps are illustrated below; further synthetic details and the most relevant spectra and chromatograms are reported in the Supporting Information.

#### 5-(3-Cyano-5-ethoxyphenyl)furan-2-carboxylic Acid (**1a**)

General procedure A. LiOH·H_2_O (2.5 mmol) was added to a solution of the suitable ester (**2a**–**o**, 1.0 mmol) in 2:1 THF/H_2_O (6 mL), after cooling on an ice–water bath. The reaction mixture was stirred at RT for a variable time. Then, THF was evaporated under reduced pressure, and the aqueous phase was adjusted to an acidic pH with 3 M HCl in an ice–water bath. The precipitated solid was collected by filtration on a Hirsch funnel and washed with small portions of a 6:4 cyclohexane/EtOAc mixture. The resulting solids were further purified by trituration, if necessary. Starting reagent: methyl 5-(3-cyano-5-ethoxyphenyl)furan-2-carboxylate (**2a**). Reaction time: 5 h. Yield: 78%. Aspect: white solid. TLC (DCM-MeOH 9:1) R_f_: 0.17. ^1^H NMR (300 MHz, DMSO-*d*_6_) δ (ppm): 12.84 (br s exch. D_2_O, 1H, COOH), 7.79 (t, *J* = 1.4 Hz, 1H, H_7_), 7.58 (dd, *J* = 2.3, 1.4 Hz, 1H, H_11_), 7.41 (dd, *J* = 2.3, 1.4 Hz, 1H, H_9_), 7.33 (d, *J* = 3.6 Hz, 1H, H_3_), 7.31 (d, *J* = 3.6 Hz, 1H, H_4_), 4.14 (q, *J* = 7.0 Hz, 2H), 1.34 (t, *J* = 7.0 Hz, 3H). ^13^C NMR (75 MHz, DMSO-*d*_6_) δ (ppm): 159.69 (C_10_), 159.56 (COOH), 154.10 (C_5_), 145.64 (C_2_), 132.13 (C_6_), 120.54 (C_3_), 119.94 (C_7_), 118.60 (CN), 117.94 (C_9_), 115.52 (C_11_), 113.72 (C_8_), 110.44 (C_4_), 64.60 (C_12_), 14.83 (C_13_). FT-IR (ATR): ν = 3516, 3124, 3088, 2983, 2940, 2908, 2233, 1687, 1593, 1519, 1441, 1397, 1368, 1328, 1311, 1287, 1221, 1172, 1051, 1023, 959, 763 cm^–1^. ESI-MS (*m*/*z*) calcd. for C_14_H_11_NO_4_ 257.24, found 256.68 [M-H]^−^, 513.21 [2M-H]^−^.

#### 5-(3-Cyano-5-isopropoxyphenyl)furan-2-carboxylic Acid (**1b**)

The compound was obtained according to general procedure A. Starting reagent: methyl 5-(3-cyano-5-isopropoxyphenyl)furan-2-carboxylate (**2b**). Reaction time: 4 h. Yield: 75%. Aspect: white solid. TLC (DCM-MeOH 9:1) R_f_: 0.18. ^1^H NMR (300 MHz, DMSO-*d*_6_) δ (ppm): 13.09 (br s exch. D_2_O, 1H, COOH), 7.77 (t, *J* = 1.3 Hz, 1H, H_7_), 7.57 (dd, *J* = 2.2, 1.3 Hz, 1H, H_11_), 7.43 (dd, *J* = 2.2, 1.3 Hz, 1H, H_9_), 7.33 (d, *J* = 3.6 Hz, 1H, H_3_), 7.28 (d, *J* = 3.6 Hz, 1H, H_4_), 4.79 (hept, *J* = 6.1 Hz, 1H, H_12_), 1.28 (d, *J* = 6.1 Hz, 6H, H_13_, H_13′_). ^13^C NMR (75 MHz DMSO-*d*_6_) δ (ppm): 159.67 (C_10_), 158.72 (COOH), 153.87 (C_5_), 144.07 (C_2_), 132.35 (C_6_), 120.42 (C_3_), 119.60 (C_7_), 118.74 (CN), 118.65 (C_9_), 116.74 (C_11_), 113.83 (C_8_), 110.39 (C_4_), 70.94 (C_12_), 22.06 (C_13_, C_13′_). FT-IR (ATR): ν = 3120, 3084, 2962, 2923, 2850, 2231, 1680, 1605, 1592, 1583, 1519, 1445, 1432, 1298, 1259, 1216, 1167, 1089, 1016, 957, 794, 760 cm^–1^. ESI-MS (*m*/*z*) calcd. for C_15_H_13_NO_4_ 271.27, found 270.80 [M-H]^−^, 541.87 [2M-H]^−^.

#### 5-(3-Cyano-5-propoxyphenyl)furan-2-carboxylic Acid (**1c**)

The compound was obtained according to general procedure A. Starting reagent: methyl 5-(3-cyano-5-propoxyphenyl)furan-2-carboxylate (**2c**). Reaction time: 1 h. Yield: 75%. Aspect: white solid. TLC (DCM-MeOH 9:1) R_f_: 0.13. ^1^H NMR (300 MHz, DMSO-*d*_6_) δ (ppm): 13.30 (br s exch. D_2_O, 1H, COOH), 7.81 (t, *J* = 1.4 Hz, 1H, H_7_), 7.59 (dd, *J* = 2.4, 1.4 Hz, 1H, H_11_), 7.43 (dd, *J* = 2.4, 1.4 Hz, 1H, H_9_), 7.35 (d, *J* = 3.6 Hz, 1H, H_3_), 7.31 (d, *J* = 3.6 Hz, 1H, H_4_), 4.05 (t, *J* = 6.5 Hz, 2H, H_12_), 1.83–1.64 (m, 2H, H_13_), 0.98 (t, *J* = 7.4 Hz, 3H, H_14_). ^13^C NMR (75 MHz, DMSO-*d*_6_) δ (ppm): 159.85 (C_10_), 159.62 (COOH), 154.04 (C_5_), 145.74 (C_2_), 132.13 (C_6_), 120.55 (C_3_), 119.93 (C_7_), 118.64 (CN), 117.88 (C_9_), 115.52 (C_11_), 113.71 (C_8_), 110.49 (C_4_), 70.30 (C_12_), 22.30 (C_13_), 10.75 (C_14_). FT-IR (ATR): ν = 3496, 3405, 3118, 3084, 2965, 2928, 2881, 2231, 1687, 1594, 1518, 1437, 1365, 1327, 1301, 1284, 1217, 1166, 1049, 1030, 963, 762 cm^–1^. ESI-MS (*m*/*z*) calcd. for C_15_H_13_NO_4_ 271.27, found 270.09 [M-H]^−^, 541.89 [2M-H]^−^.

#### 5-(3-Butoxy-5-cyanophenyl)furan-2-carboxylic Acid (**1d**)

The compound was obtained according to general procedure A. Starting reagent: methyl 5-(3-butoxy-5-cyanophenyl)furan-2-carboxylate (**2d**). Reaction time: 4 h. Yield: 70%. Aspect: off-white solid. TLC (DCM-MeOH 9:1) R_f_: 0.12. ^1^H NMR (300 MHz, DMSO-*d*_6_) δ (ppm): 13.24 (br s exch. D_2_O, 1H, COOH), 7.79 (t, *J* = 1.4 Hz, 1H, H_7_), 7.58 (dd, *J* = 2.4, 1.4 Hz, 1H, H_11_), 7.43 (dd, *J* = 2.4, 1.4 Hz, 1H, H_9_), 7.34 (d, *J* = 3.7 Hz, 1H, H_3_), 7.33 (d, *J* = 3.7 Hz, 1H, H_4_), 4.08 (t, *J* = 6.4 Hz, 2H, H_12_), 1.80–1.61 (m, 2H, H_13_), 1.54–1.36 (m, 2H, H_14_), 0.92 (t, *J* = 7.4 Hz, 3H, H_15_). ^13^C NMR (75 MHz, DMSO-*d*_6_) δ (ppm): 159.88 (C_10_), 159.48 (COOH), 154.23 (C_5_), 145.36 (C_2_), 132.08 (C_6_), 120.57 (C_3_), 120.14 (C_7_), 118.58 (CN), 117.93 (C_9_), 115.64 (C_11_), 113.74 (C_8_), 110.46 (C_4_), 68.60 (C_12_), 30.95 (C_13_), 19.06 (C_14_), 14.05 (C_15_). FT-IR (ATR): ν = 3071, 2964, 2930, 2874, 2700, 2572, 2229, 1714, 1688, 1598, 1517, 1440, 1419, 1306, 1215, 1166, 1027, 862, 807, 761 cm^–1^. ESI-MS (*m*/*z*) calcd. for C_16_H_15_NO_4_ 285.29, found 284.30 [M-H]^−^.

#### 5-(3-Cyano-5-isobutoxyphenyl)furan-2-carboxylic Acid (**1e**)

The compound was obtained according to general procedure A. Starting reagent: methyl 5-(3-cyano-5-isobutoxyphenyl)furan-2-carboxylate (**2e**). Reaction time: 4 h. Yield: 72%. Aspect: white solid. TLC (DCM-MeOH 9:1) R_f_: 0.13. ^1^H NMR (300 MHz, DMSO-*d*_6_) δ (ppm): 13.26 (br s exch. D_2_O, 1H, COOH), 7.80 (t, *J* = 1.4 Hz, 1H, H_7_), 7.59 (dd, *J* = 2.3, 1.4 Hz, 1H, H_11_), 7.43 (dd, *J* = 2.3, 1.4 Hz, 1H, H_9_), 7.35 (d, *J* = 3.6 Hz, 1H, H_3_), 7.33 (d, *J* = 3.6 Hz, 1H, H_4_), 3.87 (d, *J* = 6.5 Hz, 2H, H_12_), 2.27–1.77 (m, 1H, H_13_), 0.98 (d, *J* = 6.7 Hz, 6H, H_14_, H_14′_). ^13^C NMR (75 MHz, DMSO-*d*_6_) δ (ppm): 159.97 (C_10_), 159.48 (COOH), 154.23 (C_5_), 145.34 (C_2_), 132.09 (C_6_), 120.62 (C_3_), 120.16 (C_7_), 118.57 (CN), 117.94 (C_9_), 115.64 (C_11_), 113.74 (C_8_), 110.49 (C_4_), 74.97 (C_12_), 28.08 (C_13_), 19.36 (C_14_, C_14′_). FT-IR (ATR): ν = 3119, 1084, 2955, 2873, 2231, 1682, 1594, 1518, 1435, 1401, 1300, 1218, 1144, 1044, 961, 857, 806, 760 cm^–1^. ESI-MS (*m*/*z*) calcd. for C_16_H_15_NO_4_ 285.29, found 284.71 [M-H]^−^.

#### 5-(3-Cyano-5-(cyclopropylmethoxy)phenyl)furan-2-carboxylic Acid (**1f**)

The compound was obtained according to general procedure A. Starting reagent: methyl 5-(3-cyano-5-(cyclopropylmethoxy)phenyl)furan-2-carboxylate (**2f**). Reaction time: 5 h. Yield: 80%. Aspect: white solid. TLC (DCM-MeOH 7:3) *R*_f_: 0.37. ^1^H NMR (300 MHz, DMSO-*d*_6_) δ (ppm): 13.32 (br s exch. D_2_O, 1H, COOH), 7.78 (t, *J* = 1.4 Hz, 1H, H_7_), 7.60 (dd, *J* = 2.4, 1.4 Hz, 1H, H_11_), 7.41 (dd, *J* = 2.4, 1.4 Hz, 1H, H_9_), 7.33 (d, *J* = 3.6 Hz, 1H, H_3_), 7.29 (d, *J* = 3.6 Hz, 1H, H_4_), 3.94 (d, *J* = 7.1 Hz, 2H, H_12_), 1.32–1.09 (m, 1H, H_13_), 0.68–0.47 (m, 2H, H_14_, H_15_), 0.44–0.27 (m, 2H, H_14′_, H_15′_). ^13^C NMR (75 MHz, DMSO-*d*_6_) δ (ppm): 159.76 (C_10_), 159.67 (COOH), 153.99 (C_5_), 145.88 (C_2_), 132.13 (C_6_), 120.40 (C_3_), 119.82 (C_7_), 118.64 (CN), 118.03 (C_9_), 115.47 (C_11_), 113.64 (C_8_), 110.46 (C_4_), 73.40 (C_12_), 10.35 (C_13_), 3.58 (C_14_, C_15_). FT-IR (ATR): ν = 3406, 3115, 3087, 3001, 3088, 2968, 2892, 2705, 2662, 2230, 1689, 1598, 1517, 1435, 1423, 1368, 1308, 1216, 1167, 1147, 1043, 1028, 963, 865, 806, 758 cm^–1^. ESI-MS (*m*/*z*) calcd. for C_16_H_13_NO_4_ 283.28, found 282.97 [M-H]^−^, 565.94 [2M-H]^−^.

#### 5-(3-Cyano-5-(cyclobutylmethoxy)phenyl)furan-2-carboxylic Acid (**1g**)

The compound was obtained according to general procedure A. Starting reagent: methyl 5-(3-cyano-5-(cyclobutylmethoxy)phenyl)furan-2-carboxylate (**2g**). Reaction time: 5 h. Yield: 72%. Aspect: white solid. TLC (DCM-MeOH 9:1) R_f_: 0.26. ^1^H NMR (300 MHz DMSO-*d*_6_) δ (ppm): 13.36 (br s exch. D_2_O, 1H, COOH), 7.94 (t, *J* = 1.4 Hz, 1H, H_7_), 7.74 (dd, *J* = 2.5, 1.5 Hz, 1H, H_11_), 7.58 (dd, *J* = 2.5, 1.3 Hz, 1H, H_9_), 7.49 (d, *J* = 3.6 Hz, 1H, H_3_), 7.46 (d, *J* = 3.6 Hz, 1H, H_4_), 4.22 (d, *J* = 6.7 Hz, 2H, H_12_), 2.2–2.78 (m, 1H, H_13_), 2.29–1.86 (m, 6H, H_14_, H_15_, H_16_) ppm. ^13^C NMR (75 MHz DMSO) δ: 160.01.77 C_10_, 159.55 COOH, 154.16 C_5_, 145.58 C_2_, 132.13 C_6_, 120.59 C_7_, 120.05 C_3_, 118.61 CN, 117.96 C_11_, 115.70 C_9_, 113.74 C_8_, 110.50 C_4_, 72.71 C_12_, 34.24 C_13_, 24.71 C_14_, C_16_, 18.54 C_15_ ppm. FT-IR (ATR): ν = 3400, 3114, 3087, 3062, 2960, 2921, 2851, 2699, 2230, 1691, 1598, 1518, 1445, 1418, 1367, 1306, 1221, 1166, 1149, 1054, 1026, 962, 865, 806, 760 cm^–1^. ESI-MS (*m*/*z*) calcd for C_17_H_15_NO_4_ 297.10, found = 296.15 [M-H]^−^.

#### 5-(3-Cyano-5-(neopentyloxy)phenyl)furan-2-carboxylic Acid (**1h**)

The compound was obtained according to general procedure A. Starting reagent: methyl 5-(3-cyano-5-(neopentyloxy)phenyl)furan-2-carboxylate (**2h**). Reaction time: 4 h. Purification: trituration in cold hexane. Yield: 70%. Aspect: white solid. TLC (DCM-MeOH 9:1) R_f_: 0.31. ^1^H NMR (300 MHz, DMSO-*d*_6_) δ (ppm): 13.21 (br s exch. D_2_O, 1H, COOH), 7.82 (t, *J* = 1.2 Hz, 1H, H_7_), 7.63–7.56 (m, 1H, H_11_), 7.44 (dd, *J* = 2.1, 1.2 Hz, 1H, H_9_), 7.35 (d, *J* = 3.6 Hz, 1H, H_3_), 7.31 (d, *J* = 3.6 Hz, 1H, H_4_), 3.76 (s, 2H, H_12_), 1.00 (s, 9H, H_14_, H_14′_, H_14″_). ^13^C NMR (75 MHz, DMSO-*d*_6_) δ (ppm): 160.25 (C_10_), 159.58 (COOH), 154.09 (C_5_), 145.68 (C_2_), 132.14 (C_6_), 120.64 (C_3_), 119.92 (C_7_), 118.57 (CN), 117.84 (C_9_), 115.64 (C_11_), 113.73 (C_8_), 110.47 (C_4_), 78.41 (C_12_), 32.07 (C_13_), 26.70(C_14_, C_14′_, C_14″_). FT-IR (ATR): ν = 3087, 2953, 2870, 2701, 2234, 1699, 1682, 1608, 1583, 1574, 1526, 1438, 1425, 1340, 1303, 1262, 1216, 1170, 1047, 1032, 955, 860, 792, 758 cm^–1^. ESI-MS (*m*/*z*) calcd. for C_17_H_17_NO_4_ 299.32, found 298.48 [M-H]^−^, 597.21 [2M-H]^−^.

#### 5-(3-Cyano-5-(pentyloxy)phenyl)furan-2-carboxylic Acid (**1i**)

The compound was obtained according to general procedure A. Starting reagent: methyl 5-(3-cyano-5-(pentyloxy)phenyl)furan-2-carboxylate (**2i**). Reaction time: 4 h. Purification: trituration in cold hexane. Yield: 68%. Aspect: white solid. TLC (DCM-MeOH 9:1) R_f_: 0.23. ^1^H NMR (300 MHz, DMSO-*d*_6_) δ (ppm): 13.35 (br s exch. D_2_O, 1H, COOH), 7.80 (t, *J* = 1.4 Hz, 1H, H_7_), 7.59 (dd, *J* = 2.4, 1.4 Hz, 1H, H_11_), 7.43 (dd, *J* = 2.4, 1.4 Hz, 1H, H_9_), 7.34 (d, *J* = 3.6 Hz, 1H, H_3_), 7.30 (d, *J* = 3.6 Hz, 1H, H_4_), 4.08 (t, *J* = 6.5 Hz, 2H, H_12_), 1.81–1.63 (m, 2H, H_13_), 1.49–1.26 (m, 4H, H_14_, H_15_), 0.88 (t, *J* = 7.1 Hz, 3H, H_16_). ^13^C NMR (75 MHz, DMSO-*d*_6_) δ (ppm): 159.85 (C_10_), 159.65 (COOH), 153.98 (C_5_), 145.86 (C_2_), 132.14 (C_6_), 120.52 (C_3_), 119.82 (C_7_), 118.64 (CN), 117.81 (C_9_), 115.53 (C_11_), 113.69 (C_8_), 110.47 (C_4_), 68.82 (C_12_), 28.58 (C_13_), 28.03 (C_14_), 22.27 (C_15_), 14.34 (C_16_). FT-IR (ATR): ν = 3491, 3396, 3118, 3087, 2957, 2928, 2877, 2232, 1683, 1592, 1518, 1435, 1365, 1330, 1304, 1286, 1217, 1165, 1049, 1030, 962, 762 cm^–1^. ESI-MS (*m*/*z*) calcd. for C_17_H_17_NO_4_ 299.32, found 299.03 [M-H]^+^, 597.83 [2M-H]^−^.

#### 5-(3-Cyano-5-(isopentyloxy)phenyl)furan-2-carboxylic Acid (**1j**)

The compound was obtained according to general procedure A. Starting reagent: methyl 5-(3-cyano-5-(isopentyloxy)phenyl)furan-2-carboxylate (**2j**). Reaction time: 2 h. Purification: trituration in cold cyclohexane-EtOAc 6:4. Yield: 65%. Aspect: white solid. TLC (DCM-MeOH 9:1) R_f_: 0.21. ^1^H NMR (300 MHz, DMSO-*d*_6_) δ (ppm): 13.27 (br s exch. D_2_O, 1H, COOH), 7.80 (t, *J* = 1.4 Hz, 1H, H_7_), 7.59 (dd, *J* = 2.4, 1.4 Hz, 1H, H_11_), 7.46 (dd, *J* = 2.4, 1.4 Hz, 1H, H_9_), 7.35 (d, *J* = 3.6 Hz, 1H, H_3_), 7.32 (d, *J* = 3.6 Hz, 1H, H_4_), 4.11 (t, *J* = 6.6 Hz, 2H, H_12_), 1.87–1.70 (m, 1H, H_14_), 1.62 (q, *J* = 6.6 Hz, 2H, H_13_), 0.93 (d, *J* = 6.6 Hz, 6H, H_15_, H_15′_). ^13^C NMR (75 MHz, DMSO-*d*_6_) δ (ppm): 159.83 (C_10_), 159.57(COOH), 154.15 (C_5_), 145.48 (C_2_), 132.06 (C_6_), 120.55 (C_3_), 120.09 (C_7_), 118.64 (CN), 117.84 (C_9_), 115.64 (C_11_), 113.71 (C_8_), 110.51 (C_4_), 67.35 (C_12_), 37.64 (C_13_), 24.98 (C_14_), 22.83 (C_15_, C_15′_). FT-IR (ATR): ν = 3120, 3082, 2960, 2936, 2900, 2873, 2229, 1677, 1594, 1518, 1440, 1368, 1336, 1309, 1268, 1220, 1168, 1038, 1030, 962, 761 cm^–1^. ESI-MS (*m*/*z*) calcd. for C_17_H_17_NO_4_ 299.32, found 298.96 [M-H]^−^, 597.63 [2M-H]^−^.

#### 5-(3-Cyano-5-(hexyloxy)phenyl)furan-2-carboxylic Acid (**1k**)

The compound was obtained according to general procedure A. Starting reagent: methyl 5-(3-cyano-5-(hexyloxy)phenyl)furan-2-carboxylate (**2k**). Reaction time: 4 h. Yield: 62%. Aspect: off-white solid. TLC (DCM-MeOH 9:1) R_f_: 0.23. ^1^H NMR (300 MHz, DMSO-*d*_6_) δ (ppm): 13.24 (br s exch. D_2_O, 1H, COOH), 7.80 (t, *J* = 1.4 Hz, 1H, H_7_), 7.59 (dd, *J* = 2.3, 1.4 Hz, 1H, H_11_), 7.43 (dd, *J* = 2.3, 1.4 Hz, 1H, H_9_), 7.35 (d, *J* = 3.7 Hz, 1H, H_3_), 7.33 (d, *J* = 3.7 Hz, 1H, H_4_), 4.07 (t, *J* = 6.4 Hz, 2H, H_12_), 1.78–1.64 (m, 2H, H_13_), 1.52–1.10 (m, 6H, H_14_, H_15_, H_16_), 0.86 (t, *J* = 7.0 Hz, 3H, H_17_). ^13^C NMR (75 MHz, DMSO-*d*_6_) δ (ppm): 159.88 (C_10_), 159.49 (COOH), 154.22 (C_5_), 145.41 (C_2_), 132.10 (C_6_), 120.57 (C_3_), 120.11 (C_7_), 118.58 (CN), 117.94 (C_9_), 115.64 (C_11_), 113.74 (C_8_), 110.46 (C_4_), 68.90 (C_12_), 31.35 (C_13_), 28.87 (C_14_), 25.49 (C_15_), 22.47 (C_16_), 14.30 (C_17_). FT-IR (ATR): v = 3090, 2952, 2921, 2855, 2688, 2650, 2566, 1683,1608, 1594, 1582, 1520, 1428, 1331, 1295, 1218, 1158, 1038, 866, 786 cm^–1^. ESI-MS (*m/z)*: calcd. for C_18_H_19_NO_4_ 313.35, found 312.42 [M-H]^−^.

#### 5-(3-Cyano-5-(octyloxy)phenyl)furan-2-carboxylic Acid (**1l**)

The compound was obtained according to general procedure A. Starting reagent: methyl 5-(3-cyano-5-(octyloxy)phenyl)furan-2-carboxylate (**2l**). Reaction time: 4 h. Yield: 60%. Aspect: greyish solid. TLC (DCM-MeOH 9:1) R_f_: 0.25. ^1^H NMR (300 MHz, DMSO-*d*_6_) δ (ppm): 13.27 (br s exch. D_2_O, 1H, COOH), 7.80 (t, *J* = 1.4 Hz, 1H, H_7_), 7.58 (dd, *J* = 2.3, 1.4 Hz, 1H, H_11_), 7.43 (dd, *J* = 2.3, 1.4 Hz, 1H, H_9_), 7.35 (d, *J* = 3.6 Hz, 1H, H_3_), 7.33 (d, *J* = 3.6 Hz, 1H, H_4_), 4.07 (t, *J* = 6.4 Hz, 2H, H_12_), 1.84–1.62 (m, 2H, H_13_), 1.53–1.13 (m, 10H, H_14_, H_15_, H_16_, H_17_, H_18_), 0.84 (t, *J* = 6.7 Hz, 3H, H_19_). ^13^C NMR (75 MHz, DMSO-*d*_6_) δ (ppm): 159.87 (C_10_), 159.49 (COOH), 154.20 (C_5_), 145.43 (C_2_), 132.09 (C_6_), 120.56 (C_7_), 120.09 (C_3_), 118.58 (CN), 117.93 (C_9_), 115.64 (C_11_), 113.74 (C_8_), 110.46 (C_4_), 68.89 (C_12_), 31.65 (C_13_), 29.09 (C_14_), 29.06 (C_15_), 28.89 (C_16_), 25.82 (C_17_), 22.49 (C_18_), 14.35 (C_19_). FT-IR (ATR): ν = 3467, 2950, 2929, 2857, 2635, 2548, 2428, 2228, 1919, 1682, 1599, 1514, 1441, 1362, 1328, 1277, 1220, 1159, 1029, 963, 859, 812 cm^–1^. ESI-MS (*m/z)* calcd. for C_20_H_23_NO_4_ 341.40, found 340.43 [M-H]^−^.

#### 5-(3-(But-2-en-1-yloxy)-5-cyanophenyl)furan-2-carboxylic Acid (**1m**)

The compound was obtained according to general procedure A. Starting reagent: methyl 5-(3-(but-2-en-1-yloxy)-5-cyanophenyl)furan-2-carboxylate (**2m**). Reaction time: 4 h. Yield: 85%. Aspect: white solid. TLC (DCM-MeOH 9:1) R_f_: 0.11. ^1^H NMR (300 MHz, DMSO-*d*_6_): δ (ppm): 13.29 (br s exch. D_2_O, 1H, COOH), 7.82 (t, *J* = 1.3 Hz, 1H, H_7_*cis*), 7.81 (t, *J* = 1.4 Hz, 1H, H_7_*trans*), 7.60 (dd, *J* = 2.5, 1.5 Hz, 1H, H_11_), 7.47 (dd, *J* = 2.4, 1.2 Hz, 1H, H_9_*cis*), 7.42 (dd, *J* = 2.5, 1.3 Hz, 1H, H_9_*trans*), 7.34 (d, *J* = 3.7 Hz, AB spin system, 1H, H_3_), 7.32 (d, *J* = 3.7 Hz, AB spin system, 1H, H_4_), 5.95–5.82 (m, 1H, H_14_), 5.76–5.56 (m, 1H, H_13_), 4.73 (d, *J* = 6.3 Hz, 2H, CH_2_*cis*), 4.60 (d, *J* = 6.1 Hz, 2H, CH_2_*trans*), 1.70 (dd, *J* = 6.4, 1.5 Hz, 3H, CH_3_). ^13^C NMR (75 MHz, DMSO-*d*_6_) δ (ppm): 159.50 (C_10_), 159.48 (COOH), 154.17 (C_5_), 145.49 (C_2_), 132.11 (C_6_), 131.06 (C_14_), 126.01 (C_13_), 120.65 (C_3_), 120.07 (C_7_), 118.57(CN), 118.30 (C_9_), 115.81 (C_11_), 113.70 (C_8_), 110.47 (C_4_), 69.30 (C_12_), 17.97 (C_15_). FT-IR (ATR): ν = 3118, 3086, 2972, 2917, 1857, 2574, 2230, 1688, 1681, 1598, 1517, 1442, 1308, 1214, 1169, 1028, 864, 807, 761 cm^–1^. ESI-MS (*m*/*z*) calcd. for C_16_H_13_O_4_ 283.28, found 282.58 [M-H]^−^.

#### 5-(3-Cyano-5-((2E,4E)-hexa-2,4-dien-1-yloxy)phenyl)furan-2-carboxylic Acid (**1n**)

The compound was obtained according to general procedure A. Starting reagent: methyl 5-(3-cyano-5-((2*E*,4*E*)-hexa-2,4-dien-1-yloxy)phenyl)furan-2-carboxylate (**2n**). Reaction time: 4 h. Yield: 70%. Aspect: white solid. TLC (DCM-MeOH 9:1) R_f_: 0.21. ^1^H NMR (300 MHz, DMSO-*d*_6_) δ (ppm): 13.30 (br s exch. D_2_O, 1H, COOH), 7.81 (t, *J* = 1.4 Hz, 1H, H_7_), 7.61 (dd, *J* = 2.4, 1.4 Hz, 1H, H_11_), 7.44 (dd, *J* = 2.4, 1.4 Hz, 1H, H_9_), 7.37–7.31 (m, 2H, H_3_, H_4_), 6.38 (dd, *J* = 15.0, 10.5 Hz, 1H, H_14_), 6.11 (dd, *J* = 15.0, 10.5 Hz, 1H, H_15_), 5.84–5.69 (m, 2H, H_13_, H_16_), 4.70 (d, *J* = 6.0 Hz, 2H, H_12_), 1.72 (d, *J* = 6.5 Hz, 3H, H_17_). ^13^C NMR (75 MHz, DMSO-*d*_6_) δ (ppm): 159.46 (C_10_), 159.42 (COOH), 154.20 (C_5_), 145.40 (C_2_), 134.46 (C_14_), 132.10 (C_6_), 131.15 (C_15_), 131.05 (C_16_), 124.90 (C_13_), 120.74 (C_3_), 120.14 (C_7_), 118.56 (CN), 118.35 (C_9_), 115.90 (C_11_), 113.71 (C_8_), 110.50 (C_4_), 69.08 (C_12_), 18.31 (C_17_). FT-IR (ATR): ν = 3327, 3086, 3007, 2923, 2853, 2564, 2231, 1682, 1593, 1582, 1520, 1429, 1308, 1217, 1157, 1022, 984, 792, 760 cm^–1^. ESI-MS (*m/z)* calcd. for C_18_H_15_NO_4_ 309.32, found 308.66 [M-H]^−^.

#### 5-(3-Cyano-5-((2E,4E,6E)-octa-2,4,6-trien-1-yloxy)phenyl)furan-2-carboxylic Acid (**1o**)

The compound was obtained according to general procedure A. Starting reagent: methyl 5-(3-cyano-5-((2*E*,4*E*,6*E*)-octa-2,4,6-trien-1-yloxy)phenyl)furan-2-carboxylate (**2o**). Reaction time: 4 h. Yield: 75%. Aspect: off-white solid. TLC (DCM-MeOH 9:1) *R*_f_: 0.26. ^1^H NMR (300 MHz, DMSO-*d*_6_): δ (ppm) 13.24 (br s exch. D_2_O, 1H, COOH), 7.81 (t, *J* = 1.2 Hz, 1H, H_7_), 7.61 (dd, *J* = 2.5, 1.4 Hz, 1H, H_11_), 7.45 (dd, *J* = 2.5, 1.3 Hz, 1H, H_9_), 7.34 (s, 2H, H_3_, H_4_), 6.42 (dd, *J* = 15.5, 10.0 Hz, 1H, H_14_), 6.56–5.98 (m, 3H, H_15_, H_16_, H_17_), 5.96–5.66 (m, 2H, H_13_, H_18_), 4.73 (d, *J* = 6.3 Hz, 2H, CH_2_), 1.73 (dd, *J* = 1.9, 0.9 Hz, 3H, CH_3_). ^13^C NMR (75 MHz, DMSO-*d*_6_) δ (ppm): 159.47 (C_10_), 159.38 (COOH), 154.20 (C_5_), 145.38 (C_2_), 134.54 (C_14_), 134.40 (C_16_), 132.09 (C_6_), 131.91 (C_15_), 131.18 (C_18_), 129.54 (C_17_), 126.78 (C_13_), 120.77 (C_3_), 120.17 (C_7_), 118.57 (CN), 118.33 (C_9_), 115.88 (C_11_), 113.71 (C_8_), 110.51 (C_4_), 69.06 (C_12_), 18.51 (C_19_). FT-IR (ATR): ν = 3124, 2943, 2921, 2870, 2849, 2231, 1694, 1578, 1529, 1434, 1309, 1215, 1138, 1040, 983, 828, 760 cm^–1^. ESI-MS (*m/z)* calcd. for C_20_H_17_NO_4_ 335.35, found 335.03 [M-H]^−^.

#### Methyl 5-(3-Cyano-5-ethoxyphenyl)furan-2-carboxylate (**2a**)

General procedure B. The appropriate 5-(3-cyano-5-hydroxyphenyl)furan-2-carboxylate ester (**3a**–**c**, 1 mmol) was suspended in dry acetone (4.8 mL) under a nitrogen atmosphere. Oven-dried K_2_CO_3_ (4 mmol) was added, stirring the mixture for 10 min. Then, a solution of the suitable bromo-derivative (1.8 mmol) in dry acetone (1.2 mL) was added, and the reaction mixture was heated at reflux for a variable time. The suspension was filtered *in vacuo* to remove K_2_CO_3_, and acetone was evaporated under reduced pressure. The residue was diluted with EtOAc or Et_2_O, washed with water and brine, and then concentrated *in vacuo*. The crude product was purified by flash column chromatography to afford the desired product. Starting reagent: bromoethane. Reaction time: 5 h. Purification: flash column chromatography (cyclohexane-EtOAc 75:25). Yield: 45%. Aspect: white solid. TLC (cyclohexane-EtOAc 8:2) R_f_: 0.35. ^1^H NMR (300 MHz, CDCl_3_) δ (ppm): ^1^H NMR (300 MHz, CDCl_3_) δ (ppm): 7.61 (t, *J* = 1.4 Hz, 1H, H_Ar_), 7.50 (dd, *J* = 2.4, 1.4 Hz, 1H, H_Ar_), 7.28–7.23 (m, partially overlapped with solvent peak, 1H, H_Ar_), 7.09 (dd, *J* = 2.4, 1.4 Hz, 1H, H_Ar_), 6.79 (d, *J* = 3.6 Hz, 1H, H_Ar_), 4.11 (q, *J* = 7.0 Hz, 2H, CH_2_), 3.93 (s, 3H, COOCH_3_), 1.46 (t, *J* = 7.0 Hz, 3H, CH_3_).

#### Methyl 5-(3-Cyano-5-isopropoxyphenyl)furan-2-carboxylate (**2b**)

The compound was obtained according to general procedure B. Starting reagents: methyl 5-(3-cyano-5-hydroxyphenyl)furan-2-carboxylate (**3a**) and 2-bromopropane. Reaction time: 30 h. Purification: flash column chromatography (cyclohexane-EtOAc 7:3). Yield: 40%. Aspect: off-white solid. TLC (cyclohexane-EtOAc 8:2) R_f_: 0.35. ^1^H NMR (300 MHz, CDCl_3_) δ (ppm): 7.59 (t, *J* = 1.3 Hz, 1H, H_Ar_), 7.52–7.43 (m, 1H, H_Ar_), 7.29–7.19 (m, partially overlapped with solvent peak, 1H, H_Ar_), 7.08 (dd, *J* = 2.3, 1.3 Hz, 1H, H_Ar_), 6.78 (d, *J* = 3.6 Hz, 1H, H_Ar_), 4.62 (hept, *J* = 6.0 Hz, 1H, CH), 3.92 (s, 3H, COOCH_3_), 1.37 (d, *J* = 6.1 Hz, 6H, CH_3_).

#### Methyl 5-(3-Cyano-5-propoxyphenyl)furan-2-carboxylate (**2c**)

The compound was obtained according to general procedure B. Starting reagents: methyl 5-(3-cyano-5-hydroxyphenyl)furan-2-carboxylate (**3a**) and 1-bromopropane. Reaction time: 24 h. Purification: flash column chromatography (cyclohexane-EtOAc 8:2). Yield: 32%. Aspect: pearl white solid. TLC (cyclohexane-EtOAc 8:2) R_f_: 0.47. ^1^H NMR (300 MHz, CDCl_3_) δ (ppm): 7.61 (t, *J* = 1.4 Hz, 1H, H_Ar_), 7.51 (dd, *J* = 2.4, 1.4 Hz, 1H, H_Ar_), 7.31–7.20 (m, partially overlapped with solvent peak, 1H, H_Ar_), 7.10 (dd, *J* = 2.4, 1.4 Hz, 1H, H_Ar_), 6.80 (d, *J* = 3.6 Hz, 1H, H_Ar_), 4.00 (t, *J* = 6.5 Hz, 2H, OCH_2_), 3.93 (s, 3H, COOCH_3_), 1.92–1.77 (m, 2H, CH_2_), 1.07 (t, *J* = 7.4 Hz, 3H, CH_3_).

#### Methyl 5-(3-Butoxy-5-cyanophenyl)furan-2-carboxylate (**2d**)

The compound was obtained according to general procedure B. Starting reagents: methyl 5-(3-cyano-5-hydroxyphenyl)furan-2-carboxylate (**3a**) and 1-bromobutane. Reaction time: overnight. Purification: flash column chromatography (cyclohexane-EtOAc 9:1). Yield: 89%. Aspect: off-white solid. TLC (cyclohexane-EtOAc 8:2) R_f_: 0.43. ^1^H NMR (300 MHz, DMSO-*d*_6_) δ (ppm): 7.81 (t, *J* = 1.4 Hz, 1H, H_Ar_), 7.60 (dd, *J* = 2.4, 1.5 Hz, 1H, H_Ar_), 7.45 (dd, *J* = 2.4, 1.3 Hz, 1H, H_Ar_), 7.44 (d, *J* = 3.7 Hz, 1H, H_Ar_), 7.39 (d, *J* = 3.7 Hz, 1H, H_Ar_), 4.08 (t, *J* = 6.4 Hz, 2H, OCH_2_), 3.83 (s, 3H, COOCH_3_), 1.71 (quint, *J* = 6.6 Hz, 2H, CH_2_), 1.44 (sext, *J* = 6.6 Hz, 2H, CH_2_), 0.93 (t, *J* = 7.4 Hz, 3H, CH_3_).

#### Methyl 5-(3-Cyano-5-isobutoxyphenyl)furan-2-carboxylate (**2e**)

The compound was obtained according to general procedure B. Starting reagents: methyl 5-(3-cyano-5-hydroxyphenyl)furan-2-carboxylate (**3a**) and 1-bromo-2-methylpropane. Reaction time: 48 h. Purification: flash column chromatography (cyclohexane-EtOAc 9:1). Yield: 54%. Aspect: off-white solid. TLC (cyclohexane-EtOAc 8:2) R_f_: 0.40. ^1^H NMR (300 MHz, CDCl_3_) δ (ppm): 7.60 (t, *J* = 1.4 Hz, 1H, H_Ar_), 7.50 (dd, *J* = 2.4, 1.4 Hz, 1H, H_Ar_), 7.29–7.23 (m, partially overlapped with solvent peak, 1H, H_Ar_), 7.10 (dd, *J* = 2.4, 1.4 Hz, 1H, H_Ar_), 6.80 (d, *J* = 3.6 Hz, 1H, H_Ar_), 3.93 (s, 3H, COOCH_3_), 3.79 (d, *J* = 6.5 Hz, 2H, OCH_2_), 2.20–2.04 (m, 1H, CH), 1.05 (d, *J* = 6.7 Hz, 6H, CH_3_).

#### Methyl 5-(3-Cyano-5-(cyclopropylmethoxy)phenyl)furan-2-carboxylate (**2f**)

The compound was obtained according to general procedure B. Starting reagents: methyl 5-(3-cyano-5-hydroxyphenyl)furan-2-carboxylate (**3a**) and (bromomethyl)cyclopropane. Reaction time: 30 h. Purification: flash column chromatography (cyclohexane-EtOAc 9:1). Yield: 50%. Aspect: white solid. TLC (cyclohexane-EtOAc 8:2) R_f_: 0.37. ^1^H NMR (300 MHz, CDCl_3_) δ (ppm): 7.61 (t, *J* = 1.4 Hz, 1H, H_Ar_), 7.52 (dd, *J* = 2.4, 1.4 Hz, 1H, H_Ar_), 7.29–7.22 (m, partially overlapped with solvent peak, 1H, H_Ar_), 7.10 (dd, *J* = 2.4, 1.4 Hz, 1H, H_Ar_), 6.79 (d, *J* = 3.6 Hz, 1H, H_Ar_), 3.93 (s, 3H, COOCH_3_), 3.88 (d, *J* = 7.0 Hz, 2H, OCH_2_), 1.37–1.21 (m, 1H, CH), 0.75–0.63 (m, 2H, CH_2_), 0.44–0.34 (m, 2H, CH_2_).

#### Methyl 5-(3-Cyano-5-(cyclobutylmethoxy)phenyl)furan-2-carboxylate (**2g**)

The compound was obtained according to general procedure B. Starting reagents: methyl 5-(3-cyano-5-hydroxyphenyl)furan-2-carboxylate (**3a**) and (bromomethyl)cyclobutane. Reaction time: 48 h. Purification: flash column chromatography (cyclohexane-EtOAc 8:2). Yield: 30%. Aspect: white solid. TLC (cyclohexane-EtOAc 8:2) R_f_: 0.43. ^1^H NMR (300 MHz, CDCl_3_) δ (ppm): 7.63–7.57 (m, 1H, H_Ar_), 7.51 (dd, *J* = 2.5, 1.6 Hz, 1H, H_Ar_), 7.25 (d, *J* = 3.6 Hz, 1H, H_Ar_), 7.10 (dd, *J* = 2.5, 1.6 Hz, 1H, H_Ar_), 6.80 (d, *J* = 3.6 Hz, 1H, H_Ar_), 4.00 (d, *J* = 6.6 Hz, 2H, OCH_2_), 3.93 (s, 3H, COOCH_3_), 2.91–2.64 (m, 1H, CH), 2.24–2.09 (m, 2H, CH_2_), 2.05–1.82 (m, 4H, CH_2_).

#### Methyl 5-(3-Cyano-5-(neopentyloxy)phenyl)furan-2-carboxylate (**2h**)

The compound was obtained according to general procedure B, using DMF (instead of acetone) at 120 °C. Starting reagents: methyl 5-(3-cyano-5-hydroxyphenyl)furan-2-carboxylate (**3a**) and 1-bromo-2,2-dimethylpropane. Reaction time: 4 h. Purification: flash column chromatography (cyclohexane-EtOAc 8:2). Yield: 25%. Aspect: white solid. TLC (cyclohexane-EtOAc 7:3) R_f_: 0.68. ^1^H NMR (300 MHz, CDCl_3_) δ (ppm): 7.60 (t, *J* = 1.3 Hz, 1H, H_Ar_), 7.56–7.47 (m, 1H, H_Ar_), 7.30–7.23 (m, partially overlapped with solvent peak, 1H, H_Ar_), 7.14–7.09 (m, 1H, H_Ar_), 6.81 (d, *J* = 3.7 Hz, 1H, H_Ar_), 3.93 (s, 3H, COOCH_3_), 3.66 (s, 2H, OCH_2_), 1.06 (s, 9H, CH_3_).

#### Methyl 5-(3-Cyano-5-(pentyloxy)phenyl)furan-2-carboxylate (**2i**)

The compound was obtained according to general procedure B. Starting reagents: methyl 5-(3-cyano-5-hydroxyphenyl)furan-2-carboxylate (**3a**) and 1-bromopentane. Reaction time: overnight. Purification: flash column chromatography (cyclohexane-EtOAc 8:2) and titration in cold hexane. Yield: 48%. Aspect: white solid. TLC (cyclohexane-EtOAc 8:2) R_f_: 0.53. ^1^H NMR (300 MHz, CDCl_3_) δ (ppm): 7.60 (t, *J* = 1.4 Hz, 1H, H_Ar_), 7.50 (dd, *J* = 2.4, 1.4 Hz, 1H, H_Ar_), 7.30–7.20 (m, partially overlapped with solvent peak, 1H, H_Ar_), 7.10 (dd, *J* = 2.4, 1.4 Hz, 1H, H_Ar_), 6.80 (d, *J* = 3.7 Hz, 1H, H_Ar_), 4.03 (t, *J* = 6.5 Hz, 2H, OCH_2_), 3.93 (s, 3H, COOCH_3_), 1.89–1.75 (m, 2H, CH_2_), 1.53–1.31 (m, 4H, CH_2_), 0.95 (t, *J* = 7.1 Hz, 3H, CH_3_).

#### Methyl 5-(3-Cyano-5-(isopentyloxy)phenyl)furan-2-carboxylate (**2j**)

The compound was obtained according to general procedure B. Starting reagents: methyl 5-(3-cyano-5-hydroxyphenyl)furan-2-carboxylate (**3a**) and 1-bromo-3-methylbutane. Reaction time: 5 h. Purification: flash column chromatography (cyclohexane-EtOAc 8:2). Yield: 30%. Aspect: white solid. TLC (cyclohexane-EtOAc 8:2) R_f_: 0.42. ^1^H NMR (300 MHz, CDCl_3_) δ (ppm): 7.60 (t, *J* = 1.4 Hz, 1H, H_Ar_), 7.50 (dd, *J* = 2.4, 1.4 Hz, 1H, H_Ar_), 7.28–7.22 (m, partially overlapped with solvent peak, 1H, H_Ar_), 7.10 (dd, *J* = 2.4, 1.4 Hz, 1H, H_Ar_), 6.80 (d, *J* = 3.6 Hz, 1H, H_Ar_), 4.06 (t, *J* = 6.5 Hz, 2H, OCH_2_), 3.93 (s, 3H, COOCH_3_), 1.93–1.78 (m, 1H, CH), 1.71 (q, *J* = 6.5 Hz, 2H, CH_2_), 0.98 (d, *J* = 6.5 Hz, 6H, CH_3_).

#### Methyl 5-[3-Cyano-5-(hexyloxy)phenyl]furan-2-carboxylate (**2k**)

The compound was obtained according to general procedure B. Starting reagents: methyl 5-(3-cyano-5-hydroxyphenyl)furan-2-carboxylate (**3a**) and 1-bromohexane. Reaction time: overnight. Purification: flash column chromatography (cyclohexane-EtOAc 8:2). Yield: 80%. Aspect: white solid. TLC (cyclohexane-EtOAc 8:2) R_f_: 0.38. ^1^H NMR (300 MHz, CDCl_3_) δ (ppm): 7.60 (t, *J* = 1.4 Hz, 1H, H_Ar_), 7.50 (dd, *J* = 2.4, 1.4 Hz, 1H, H_Ar_), 7.29–7.22 (m, partially overlapped with solvent peak, 1H, H_Ar_), 7.10 (dd, *J* = 2.4, 1.4 Hz, 1H, H_Ar_), 6.80 (d, *J* = 3.7 Hz, 1H, H_Ar_), 4.02 (t, *J* = 6.5 Hz, 2H, OCH_2_), 3.93 (s, 3H, COOCH_3_), 1.87–1.75 (m, 2H, CH_2_), 1.54–1.42 (m, 2H, CH_2_), 1.40–1.31 (m, 4H, CH_2_), 0.92 (t, *J* = 7.0 Hz, 3H, CH_3_).

#### Methyl 5-[3-Cyano-5-(octyloxy)phenyl]furan-2-carboxylate (**2l**)

The compound was obtained according to general procedure B. Starting reagents: methyl 5-(3-cyano-5-hydroxyphenyl)furan-2-carboxylate (**3a**) and 1-bromooctane. Reaction time: overnight. Purification: recrystallization from hexane. Yield: 42%. Aspect: greyish solid. TLC (cyclohexane-EtOAc 8:2) R_f_: 0.52. ^1^H NMR (300 MHz, DMSO-*d*_6_) δ (ppm): 7.81 (m, 1H, H_Ar_), 7.59 (m, 1H, H_Ar_), 7.45 (m, 1H, H_Ar_), 7.44 (d, *J* = 3.8 Hz, 1H, H_Ar_), 7.39 (d, *J* = 3.8 Hz, 1H, H_Ar_), 4.08 (t, *J* = 6.4 Hz, 2H, OCH_2_), 3.83 (s, 3H, COOCH_3_), 3.50 (t, *J* = 6.7 Hz, 2H, CH_2_), 1.97–1.61 (m, 4H, CH_2_), 1.50–1.31 (m, 4H, CH_2_), 0.84 (t, *J* = 6.0 Hz, 3H, CH_3_).

### Methyl 5-[(3-But-2-en-1-yloxy)-5-cyanophenyl]furan-2-carboxylate (**2m**)

The compound was obtained according to general procedure B. Starting reagents: methyl 5-(3-cyano-5-hydroxyphenyl)furan-2-carboxylate (**3a**) and 1-bromobut-2-ene predominantly *trans* (80:20). Reaction time: 3 h. Purification: flash column chromatography (cyclohexane-EtOAc 8:2). Yield: 35%. Aspect: white solid. TLC (cyclohexane-EtOAc 8:2) R_f_: 0.35. ^1^H NMR (300 MHz, DMSO-*d*_6_) δ (ppm): 7.83 (t, *J* = 1.4 Hz, 1H, *cis* H_Ar_), 7.81 (t, *J* = 1.4 Hz, 1H, *trans* H_Ar_), 7.61 (dd, *J* = 2.5, 1.5 Hz, 1H, H_Ar_), 7.48 (dd, *J* = 2.5, 1.3 Hz, 1H, *cis* H_Ar_), 7.46 (dd, *J* = 2.5, 1.3 Hz, 1H, *trans* H_Ar_), 7.44 (d, *J* = 3.7 Hz, 1H, H_Ar_), 7.38 (d, *J* = 3.7 Hz, 1H, H_Ar_), 5.99–5.80 (m, 1H, CH), 5.78–5.70 (m, 1H, CH), 4.74 (d, *J* = 6.3 Hz, 2H, *cis* CH_2_), 4.60 (d, *J* = 6.1 Hz, 2H, *trans* CH_2_), 3.83 (s, 3H, COOCH_3_), 1.74 (dd, *J* = 7.1, 1.5 Hz, 3H, *cis* CH_3_), 1.70 (dd, *J* = 6.4, 1.3 Hz, 3H, *trans* CH_3_).

#### Methyl 5-[3-Cyano-5-(2E,4E-hexa-2,4- dien-1-yloxy)phenyl]furan-2-carboxylate (**2n**)

The compound was obtained according to general procedure B. Starting reagents: methyl 5-(3-cyano-5-hydroxyphenyl)furan-2-carboxylate (**3a**) and (2*E*,4*E*)-1-bromohexa-2,4-diene (for further synthetic information, see Supporting Information).^72^ Reaction time: 1 h. Purification: recrystallization from Et_2_O/hexane. Yield: 70%. Aspect: white solid. TLC (cyclohexane-EtOAc 8:2) R_f_: 0.22. ^1^H NMR (300 MHz, DMSO-*d*_6_) δ (ppm) 7.83 (t, *J* = 1.4 Hz, 1H, H_Ar_), 7.62 (dd, *J* = 2.5, 1.5 Hz, 1H, H_Ar_), 7.48 (dd, *J* = 2.5, 1.3 Hz, 1H, H_Ar_), 7.45 (d, *J* = 3.7 Hz, 1H, H_Ar_), 7.39 (d, *J* = 3.7 Hz, 1H, H_Ar_), 6.38 (dd, *J* = 15.1, 10.7 Hz, 1H, CH), 6.06–6.15 (m, 1H, CH), 5.71–5.80 (m, 2H, CH), 4.7 (d, *J* = 5.9 Hz, 2H, OCH_2_), 1.72 (d, *J* = 6.6 Hz, 3H, CH_3_).

#### Methyl 5-[3-Cyano-5-(2E,4E,6E-octa-2,4,6-trien-1-yloxy)phenyl]furan-2-carboxylate (**2o**)

The compound was obtained according to general procedure B. Starting reagents: methyl 5-(3-cyano-5-hydroxyphenyl)furan-2-carboxylate (**3a**) and (2*E*,4*E*,6*E*)-1-bromoocta-2,4,6-triene (for further synthetic information, see Supporting Information).^72^ Reaction time: 30 min. Purification: recrystallization from Et_2_O/hexane. Yield: 23%. Aspect: light brown solid. TLC (cyclohexane-EtOAc 8:2) R_f_: 0.32. ^1^H NMR (300 MHz, DMSO-*d*_6_) δ (ppm) 7.86–7.81 (m, 1H, H_Ar_), 7.66–7.59 (m, 1H, H_Ar_), 7.48 (dd, *J* = 2.4, 1.3 Hz, 1H, H_Ar_), 7.45 (d, *J* = 3.7 Hz, 1H, H_Ar_), 7.39 (d, *J* = 3.7 Hz, 1H, H_Ar_), 6.43 (dd, *J* = 15.3, 10.1 Hz, 1H, CH) 6.55–6.02 (m, 3H, CH), 5.94–5.68 (m, 2H, CH), 4.74 (d, *J* = 6.2 Hz, 2H, OCH_2_), 3.83 (s, 3H, COOCH_3_), 1.73 (d, *J* = 6.9 Hz, 2H, CH_3_).

#### Ethyl 5-(3-Cyano-5-isobutoxyphenyl)furan-2-carboxylate (**2ea**)

The compound was obtained according to general procedure B. Starting reagents: ethyl 5-(3-cyano-5-hydroxyphenyl)furan-2-carboxylate (**3b**) and 1-bromo-2-methylpropane. Reaction time: 48 h. Purification: flash column chromatography (cyclohexane-EtOAc 85:15). Yield: 30%. Aspect: off-white solid. TLC (cyclohexane-EtOAc 8:2) R_f_: 0.42. ^1^H NMR (300 MHz, CDCl_3_) δ (ppm): 7.61 (t, *J* = 1.4 Hz, 1H, H_7_), 7.51 (dd, *J* = 2.4, 1.4 Hz, 1H, H_9_), 7.24 (d, *J* = 3.6 Hz, 1H, H_3_), 7.10 (dd, *J* = 2.4, 1.4 Hz, 1H, H_11_), 6.80 (d, *J* = 3.6 Hz, 1H, H_4_), 4.40 (q, *J* = 7.1 Hz, 2H, H_15_), 3.79 (d, *J* = 6.5 Hz, 2H, H_12_), 2.20–2.02 (m, 1H, H_13_), 1.41 (t, *J* = 7.1 Hz, 3H, H_16_), 1.05 (d, *J* = 6.7 Hz, 6H, H_14_, H_14′_). ^13^C NMR (75 MHz, CDCl_3_) δ (ppm): 159.78 (C_10_), 158.54 (COO), 154.75 (C_5_), 144.73 (C_2_), 131.88 (C_6_), 120.50 (C_3_), 119.57 (C_7_), 118.25 (CN), 117.35 (C_9_), 115.41 (C_11_), 113.86 (C_8_), 108.49 (C_4_), 74.98 (C_12_), 61.18 (C_15_), 28.20 (C_16_), 19.14 (C_14_, C_14′_). FT-IR (ATR): ν = 3114, 3083, 2962, 2919, 2892, 2877, 2229, 1701, 1611, 1579, 1527, 1472, 1445, 1433, 1367, 1345, 1295, 1262, 1217, 1147, 1043, 1021, 954, 879, 868, 848, 836, 810, 802, 757 cm^–1^. ESI-MS (*m*/*z*) calcd. for C_18_H_19_NO_4_ 313.35, found 336.15 [M + Na]^+^, 353.37 [M+K]^+^, 648.81 [2M+Na]^+^.

#### Propyl 5-(3-Cyano-5-isobutoxyphenyl)furan-2-carboxylate (**2eb**)

The compound was obtained according to general procedure B. Starting reagents: propyl 5-(3-cyano-5-hydroxyphenyl)furan-2-carboxylate (**3c**) and 1-bromo-2-methylpropane. Reaction time: 48 h. Purification: flash column chromatography (cyclohexane-EtOAc 85:15). Yield: 25%. Aspect: off-white solid. TLC (cyclohexane-EtOAc 8:2) R_f_: 0.51. ^1^H NMR (300 MHz, CDCl_3_) δ (ppm): 7.61 (t, *J* = 1.4 Hz, 1H, H_7_), 7.52 (dd, *J* = 2.4, 1.4 Hz, 1H, H_11_), 7.25 (d, *J* = 3.6 Hz, 1H, H_3_), 7.11 (dd, *J* = 2.4, 1.4 Hz, 1H, H_9_), 6.81 (d, *J* = 3.6 Hz, 1H, H_4_), 4.31 (t, *J* = 6.7 Hz, 2H, H_15_), 3.80 (d, *J* = 6.5 Hz, 2H, H_12_), 2.30–2.02 (m, 1H, H_13_), 1.91–1.72 (m, 2H, H_16_), 1.06 (d, *J* = 6.7 Hz, 6H, H_14_, H_14′_), 1.04 (t, *J* = 7.4 Hz, 3H, H_17_). ^13^C NMR (75 MHz, CDCl_3_) δ (ppm): 159.80 (C_10_), 158.64 (COO), 154.78 (C_5_), 144.73 (C_2_), 131.91 (C_6_), 120.50 (C_3_), 119.53 (C_7_), 118.27 (CN), 117.40 (C_9_), 115.40 (C_11_), 113.89 (C_8_), 108.49 (C_4_), 75.00 (C_12_), 66.67 (C_15_), 28.20 (C_13_), 22.09 (C_16_), 19.14 (C_14_, C_14′_), 10.39 (C_17_). FT-IR (ATR): ν 3118, 3077, 2965, 2940, 2880, 2228, 1708, 1610, 1581, 1526, 1445, 1432, 1347, 1340, 1297, 1265, 1216, 1144, 1042, 1025, 971, 809, 757 cm^–1^. ESI-MS (*m*/*z*) calcd. for C_19_H_21_NO_4_ 327.37, found 350.31 [M + Na]^+^, 676.79 [2M+Na]^+^.

#### Methyl 5-(3-Cyano-5-hydroxyphenyl)furan-2-carboxylate (**3a**)

3-Bromo-5-hydroxybenzonitrile (200 mg, 1.01 mmol), (5-(methoxycarbonyl)furan-2-yl)boronic acid (224 mg, 1.31 mmol), and bis(triphenylphosphine)palladium dichloride (35 mg, 0.05 mmol) were dissolved in dry 1,4-dioxane (5 mL) under N_2_ atmosphere. A 2 M Na_2_CO_3_ solution (1 mL, 2.00 mmol) was added, and the resulting mixture was stirred in a microwave synthesizer at 60 °C for 80 min. The reaction was filtered on a Celite pad, diluted with H_2_O (10 mL) and extracted with EtOAc (3 × 10 mL). The organic layers were dried over anhydrous Na_2_SO_4_, filtered, and concentrated under reduced pressure. The crude was washed with a 7:3 cyclohexane/EtOAc mixture, and the resulting greenish solid was collected by filtration. Yield: 90%. TLC (cyclohexane-EtOAc 8:2) R_f_: 0.16. ^1^H NMR (300 MHz, DMSO-*d*_6_) δ (ppm): 10.51 (br s exch. D_2_O, 1H, OH), 7.74 (t, *J* = 1.3 Hz, 1H, H_Ar_), 7.52–7.46 (m, 1H, H_Ar_), 7.43 (d, *J* = 3.7 Hz, 1H, H_Ar_), 7.31 (d, *J* = 3.7 Hz, 1H, H_Ar_), 7.15 (dd, *J* = 2.2, 1.3 Hz, 1H, H_Ar_), 3.83 (s, 3H, COOCH_3_).

#### Ethyl 5-(3-Cyano-5-hydroxyphenyl)furan-2-carboxylate (**3b**)

General procedure C. 3-Bromo-5-hydroxybenzonitrile (100 mg, 0.50 mmol), the appropriate furan-2-carboxylate (425 mg, 3.00 mmol), Pd(OAc)_2_ (17 mg, 0.075 mmol), and KOAc (294 mg, 3.00 mmol) were charged in a microwave tube and dissolved in dry DMA (3.8 mL) under Ar atmosphere. The resulting mixture was heated at 125 °C for 30 min in a microwave synthesizer. Then, water was added, and the reaction was extracted in EtOAc (3 × 5 mL); the collected organic layers were dried over anhydrous Na_2_SO_4_ and evaporated under reduced pressure. The crude was purified by flash column chromatography (cyclohexane-EtOAc 75:25) to afford the desired product. Starting reagent: ethyl furan-2-carboxylate (**4a**). Yield: 30%. Aspect: white solid. TLC (cyclohexane-EtOAc 7:3) R_f_: 0.35. ^1^H NMR (300 MHz, DMSO-*d*_6_) δ (ppm): 10.57 (br s exch. D_2_O, OH), 7.72 (t, *J* = 1.4 Hz, 1H, H_Ar_), 7.49 (dd, *J* = 2.3, 1.4 Hz, 1H, H_Ar_), 7.40 (d, *J* = 3.7 Hz, 1H, H_Ar_), 7.29 (d, *J* = 3.7 Hz, 1H, H_Ar_), 7.14 (dd, *J* = 2.3, 1.4 Hz, 1H, H_Ar_), 4.30 (q, *J* = 7.1 Hz, 2H, OCH_2_), 1.30 (t, *J* = 7.1 Hz, 3H, CH_3_).

#### Propyl 5-(3-Cyano-5-hydroxyphenyl)furan-2-carboxylate (**3c**)

The compound was obtained according to general procedure C. Starting reagent: ethyl furan-2-carboxylate (**4b**). Yield: 28%. Aspect: white solid. TLC (cyclohexane-EtOAc 7:3) R_f_: 0.37. ^1^H NMR (300 MHz, CDCl_3_) δ (ppm) 7.75–7.43 (m, 2H, H_Ar_), 7.30–7.20 (m, 1H, partially overlapped with solvent peak, H_Ar_), 7.16 (dd, *J* = 2.4, 1.4 Hz, 1H, H_Ar_), 6.80 (d, *J* = 3.6 Hz, 1H, H_Ar_), 6.44 (br s exch. D_2_O, 1H, OH), 4.31 (t, *J* = 6.7 Hz, 2H, OCH_2_), 1.81 (h, *J* = 7.3 Hz, 2H, CH_2_), 1.04 (t, J = 7.4 Hz, 3H, CH_3_).

#### Ethyl Furan-2-carboxylate (**4a**)

General procedure D. Concentrated H_2_SO_4_ (1.5 mL) was added dropwise to a solution of furan-2-carboxylic acid (2000 mg, 17.84 mmol) in the appropriate alcohol (70 mL). The mixture was stirred at reflux for 24 h. Then, the solvent was removed under reduced pressure, water was added in an ice bath, and the aqueous residue was brought to basic pH (pH ≈ 8) with NaHCO_3_ before being extracted with EtOAc (3 × 15 mL). The collected organic fractions were dried over anhydrous Na_2_SO_4_, filtered, and evaporated *in vacuo* to afford the desired product. Starting reagent: ethanol. Yield: 97%. Aspect: white solid. TLC (cyclohexane-EtOAc 8:2) R_f_: 0.55. ^1^H NMR (300 MHz, CDCl_3_) δ (ppm): 7.60–7.49 (m, 1H, H_Ar_), 7.17 (d, *J* = 3.6 Hz, 1H, H_Ar_), 6.50 (dd, *J* = 3.6, 1.7 Hz, 1H, H_Ar_), 4.36 (q, *J* = 7.1 Hz, 2H, OCH_2_), 1.38 (t, *J* = 7.1 Hz, 3H, CH_3_).

#### Propyl Furan-2-carboxylate (**4b**)

The compound was obtained according to general procedure D. Starting reagent: *n*-propanol. Yield: 95%. Aspect: colorless oil. TLC (cyclohexane-EtOAc 8:2) R_f_: 0.60. ^1^H NMR (300 MHz, CDCl_3_) δ (ppm): 7.57 (dd, *J* = 1.6, 0.7 Hz, 1H, H_Ar_), 7.17 (dd, *J* = 3.5, 0.7 Hz, 1H, H_Ar_), 6.50 (dd, *J* = 3.5, 1.6 Hz, 1H, H_Ar_), 4.27 (t, *J* = 6.7 Hz, 2H, OCH_2_), 1.86–1.69 (m, 2H, CH_2_), 1.00 (t, *J* = 7.4 Hz, 3H, CH_3_).

### Crystallography

Platy crystals of **1d** were obtained by the slow evaporation of an ethanol–water solution at room temperature. X-ray diffraction data were collected at 293 K with a Rigaku XtaLAB Synergy-S diffractometer (Rigaku, Tokyo, Japan), equipped with a microfocus Mo–Kα source and a HyPix-6000HE detector, and operated at 50 kV and 1 mA. Data collection was performed with omega scans with a step size of 1° and an exposure time of 30 s/frame. A total number of 23,925 Bragg reflections were collected, giving a triclinic unit, space group P-1. Data integration, Lorentz-polarization and symmetry-related absorption corrections were carried out using CrysAlisPRO 1.171.41.105a (CrysAlisPro Software System, Rigaku Oxford Diffraction/Agilent Technologies UK Ltd., Yarnton, UK). The structure was solved by direct methods using SIR2014 and completed by iterative cycles of full-matrix least-squares refinement on *F*_*o*_^2^ and Δ*F* synthesis using SHELXL-2019/3 within the WinGX suite (WinGX v.2021.3).^53,73,74^ Hydrogen atoms bound to the alkyl chain were introduced at calculated positions in their described geometries and allowed to ride on the attached atom with fixed isotropic thermal parameters (1.2 Ueq and 1.5 Ueq of the parent atom for methylene and methyl groups, respectively). Aromatic and oxygen-bound hydrogens were located in a difference-Fourier map and refined freely. The structure was analyzed with PARST and Mercury 2022.3.0;^75,76^ graphical representations were generated with ORTEP-3 (v.2020.1) and Mercury.^53,76^ Hirshfeld surface (HS) analysis was performed with CrystalExplorer 21 (v.21.5).^77^

#### Crystal Data for **1d**

Formula: C_16_H_15_NO_4_; MW = 285.29 g/mol; Bravais lattice: triclinic; Space group: P-1; Cell dimensions: *a* = 4.8371(4) Å, *b* = 11.7691(8) Å, *c* = 13.9543(7) Å, α = 70.803(5)°, β = 86.010(6)°, γ = 80.261(7)°; *V* = 739.33(9) Å^3^; *Z* = 2; *D*_calc_ = 1.282 Mg/m^3^; 2θ_min_ = 1.545°; 2θ_max_ = 24.712°; Limiting indices = −5 ≤ *h* ≤ 5, – 13 ≤ *k* ≤ 13, – 16 ≤ *l* ≤ 16; Crystal size: 0.22 × 0.09 × 0.02 mm; *T* = 293(2) K; *F*(000) = 300; *R*_int_ = 0.0942; Data/restraints/parameters: 2523/1/223; *R* = 0.0678 for 1238 reflections with *F*_0_ > 4sig(*F*_0_) (*R* = 0.1381 for all 2523 unique/23925 collected reflections), wR2 = 0.1703 for reflections with *F*_0_ > 4sig(*F*_0_) (wR2 = 0.2115 for all unique reflections); GOOF = 1.025; Residual positive and negative electron densities in the final map: 0.374 and −0.182 eÅ^–3^. CCDC accession number: 2282105.

### Enzymatic Assays, MIC Determination, Time-Killing Assay, Siderophore Quantification, and Efflux Pump Experiments

MbtI from *Mtb* was produced in recombinant form, and enzyme activity was determined using a fluorimetric assay, as previously reported.^38^ For the inhibition studies, the activity was determined in the presence of 100 μM of the test compound and 50 μM of chorismic acid. For compounds that inhibited more than 80% of the initial activity, IC_50_ values were calculated using eq 1 and GraphPad Prism 8.0 software
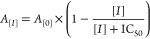
1where *A*_[*I*]_ is the activity at inhibitor concentration [*I*] and *A*_[0]_ is the activity in the absence of the inhibitor.

For the most active compounds, the minimal inhibitory concentration (MIC_99_) was measured against *M. bovis* BCG in low-iron Chelated Sauton’s medium using the resazurin reduction assay (REMA). Siderophore activity was determined by the Universal CAS liquid assay, as previously reported.^35^ For time-kill assays 1 × MIC and 10 × MIC concentrations of compound **1e** in low-iron Chelated Sauton’s medium were prepared in a 24-well plate. Then, *M. bovis* BCG cultures were added to a final density of 5 × 10^5^ cells/mL, and plates were incubated at 37 °C. At each time point, 20 μL of samples were added to 180 μL of phosphate-buffered saline solution containing 0.1% tyloxapol, and 2.5 μL of dilution plated on agar plates containing 0.4% of activated charcoal; agar plates were incubated for 7 days at 37 °C and CFU counted. To evaluate the impact of drug efflux transporters on the susceptibility of *M. bovis* BCG to the compounds, the MIC_99_ was measured (as reported above) in the presence of 32 μg/mL of reserpine or 125 μg/mL of verapamil, two validated efflux inhibitors. The concentration of the efflux inhibitors was 1/2 times their MIC.^78^

### Ester Hydrolysis Experiments

200 mL of an *M. bovis* BCG culture (OD_600_ = 0.8) was treated with 4.5 mg of **2ea** (dissolved in 2 mL of DMSO) and incubated for 24 h at 37 °C. The culture was centrifuged to separate the cells from the broth. The latter was extracted with EtOAc (3 × 50 mL), and the unified organic phases were combined and dried under vacuum. Cells were washed 2 times with PBS, then resuspended in 100 mL PBS and lysed by sonication. The cell lysate was extracted with EtOAc (3 × 50 mL), and the organic layers were combined and dried under *vacuum*. The samples were analyzed in UHPLC/MS, with a JASCO X-LC system (JASCO Europe S.r.l., Cremella, Italy), coupled with a ThermoFisher LTQ XL HESI-MS/MS system (ThermoFisher Scientific, Waltham, MA). Chromatography was performed on a Waters Acquity column (Waters; 3 μm particle size), maintained at 35 °C with an “on-line” oven equipment. Gradient elution was performed with acetonitrile/water–0.2% HCOOH (from 10:90 to 100:0 in 10 min) as a mobile phase; the flow rate was 0.3 mL min^–1^. The analyses were performed in full-scan mode from 150 and 2000 u.m.a. in negative mode (HESI Probe: Gas = N_2_, *T* = 95 °C, Voltage = 3.2 kV; Capillary T = 275 °C, Voltage = 48 V, Tube Lens = 72 V. Tune Settings: Multipole 00 Offset = 2.4 V, Lens 0 = −4.26 V, Multipole 0 Offset = −5.18 V, Lens 1 = −8.95 V, Gate Lens = −65.1 V, Multipole 1 Offset = −6.3 V, Multipole RF Amplitude (p-p)= 400 V, Front Lens = −6.1 V. Settings for MS-MS and MS^3^: detection by CID (Collision Induced Dissociation); Isolation Width: ± 2 d; Activation Q: 0.250; Activation Time 30.0 ms. Isolation width for quantitation ±2.5 d). The runs were also monitored by recording the absorbance at 220 nm. ESI-MS (*m*/*z*) calcd. for C_18_H_19_NO_4_ 313.12, found 312.25 [M-H]^−^. Mass analysis of lysed cell = ESI-MS (*m*/*z*) calcd. for C_16_H_15_NO_4_ 285.10, found 284.23 [M-H]^−^.

### Molecular Docking Studies

The X-ray structure of MbtI in complex with **I** (PDB code: 6ZA4) was downloaded from the Protein Data Bank and used for docking studies.^79^ Prior to docking, the protein loop corresponding to residues 270–277, which was unresolved in the X-ray structure, was automatically reconstructed using Modeler software. Docking calculations were performed with GOLD software using the ChemScore fitness function.^80^ The region of interest for the docking calculations included all residues within 10 Å from the bound ligand in the reference X-ray structures. Compound **1e** was subjected to 100 genetic algorithm runs, in which the “allow early termination” option was deactivated, while the possibility for the ligand to flip ring corners was activated. All other settings were left as their defaults. The RMSD threshold for pose clustering was set to 2.0 Å. The best-docked conformation belonging to the best cluster of solutions was considered.

### Molecular Dynamics Simulations

All MD simulations were carried out with AMBER22,^81^ using ff14SB force field for the protein. In contrast, GAFF (General Amber force field) was used for the ligands, whose partial charges were calculated using the AM1-BCC method through the antechamber suite of AMBER22. The ligand–protein complexes generated by docking, as well as the reference MbtI in complex with **I**, were included in a parallelepiped box and solvated with a 15 Å water cap using the TIP3P explicit solvent model. Sodium ions were added for the neutralization of the systems. Before the MD simulations, a two-stage minimization protocol was used for each complex. A 5000-step minimization, including 2000 steps of steepest descent (SD), followed by 3000 steps of conjugate gradient (CG), was initially performed. In this stage, a position restraint of 100 kcal/mol·Å^2^ was applied on all residues and ligand-heavy atoms to uniquely minimize the positions of the water molecules and the orientation of rotatable polar hydrogens. A second minimization stage, including 5000 total steps of SD/CG algorithms, was then performed, applying a harmonic potential of 10 kcal/mol·Å^2^ only to the protein α-carbons, thus energy-minimizing the entire system. The energy-minimized systems were used as inputs for an MD simulation protocol in which particle mesh Ewald (PME) electrostatics and periodic boundary conditions were used, with a cutoff of 10 Å for the nonbonded interactions. A time step of 2.0 fs was employed in the simulations since all bonds involving hydrogen atoms were kept fixed using the SHAKE algorithm. For each complex, a constant volume MD simulation was carried out for the first 1.0 ns, during which the temperature of the system was increased from 0 to 300 K. The system was then equilibrated through 50 ns of constant-pressure simulation, which was carried out keeping the temperature at the constant value of 300 K by using a Langevin thermostat. Finally, an additional constant-pressure MD simulation stage of 500 ns was performed for each system for a total MD simulation time of 551 ns. In all MD stages, a 10 kcal/(mol·Å^2^) harmonic potential was applied to the protein α-carbons, excluding the 268–279 loop region. All the obtained MD trajectories were analyzed using the Cpptraj program implemented in AMBER22.

### PMPC–PDPA Copolymer Synthesis

The PMPC–PDPA polymer was synthesized by a typical Atom Transfer Radical Polymerization (ATRP) procedure. In detail, a solution of 2-methacryloyloxyethyl phosphorylcholine (MPC, 16.9 mmol), and 2-(4-morpholino)ethyl 2-bromoisobutyrate (ME-Br) initiator (0.7 mmol) in 6 mL of ethanol was deoxygenated by purging with nitrogen for 1 h under stirring at room temperature. Then, 2,2′-bipyridine (bpy) ligand (1.4 mmol) and CuBr (0.7 mmol) were added, and the reaction was carried out under a nitrogen atmosphere at 30 °C. After 90 min, a solution of 2(diisopropylamino)ethyl methacrylate (DPA, 57.6 mmol) in ethanol (15 mL), previously deoxygenated by purging with N_2_ for 1 h at room temperature, was injected into the flask. After 48 h, the reaction solution was opened to the air, diluted by the addition of ethanol (∼200 mL), and left stirring for 1 h. The copper catalyst was removed by eluting the reaction mixture through a silica column. The filtrate was concentrated by evaporation *in vacuo* and dialyzed using a 1 kDa MWCO dialysis membrane (Spectrum Laboratories, Breda, The Netherlands) against chloroform/methanol 2:1 (*v*/*v*) (2 × 500 mL), methanol (2 × 500 mL), and double-distilled water (4 × 2 L). At least 8 h passed between changes. After dialysis, the copolymer was isolated by freeze-drying and characterized by ^1^H NMR spectroscopy, performed on a Bruker Avance III 600 spectrometer (Bruker), and gel permeation chromatography (GPC). The GPC analysis was carried out on a Malvern Viscotek GPCmax equipped with an RI detector (Malvern Panalytical, Malvern, UK), using acidic water (0.25 vol % TFA in water) as the solvent and a NOVEMA Max column (including a guard column) from PSS Polymers (PSS Polymer Standards Service GmbH, Mainz, Germany).

### Polymersome Production and Characterization

PMPC_25_–PDPA_74_ self-assembly in polymersomes and compound encapsulation were carried out using the solvent-switch technique in sterile conditions. In detail, the polymers and the desired payload were first dissolved in 1 mL of a THF/methanol solution (1:3), at a concentration of 20 and 2 mg/mL, respectively. 2.3 mL of Milli-Q water was injected using a NE-1000 One Channel Programmable Syringe Pump (New Era Pump Systems Inc., Farmingdale, NY) into the polymer solution under a constant stirring (500 rpm) and at 40 °C. The water was injected with a flow rate of 2 μL/min. The resulting milky liquid was purified by dialysis using a 3.5 kDa Spectra/Por 6 bag (Spectrum Laboratories Inc., Piscataway, NJ) against Milli-Q water for 1 h, and then PBS over 2 days, changing the bath solution of PBS every 8 h. The polymersomes were then centrifuged at 1000 Rotational Centrifugal Force (RCF) for 10 min, the supernatant was collected, and the resulting pellet was discarded. The samples were then further purified by filtration using a 0.22 μM sterile filter.

The procedure reported above was also applied to produce and characterize the polymersomes functionalized with Cy5, using 18 mg/mL of PMPC–PDPA, 2 mg/mL of PMPC–PDPA Cy5, and 2 mg/mL of the desired compound.

The purified solutions were then analyzed by Transmission Electron Microscopy (TEM) on a Jeol JEM 1010 100 kV microscope, equipped with a Megaview 1kx1k CCD (Jeol Ltd., Tokyo, Japan). Copper grids were glow-discharged, and the sample was adsorbed onto the grid. The sample was stained with 1 wt % phosphotungstic acid (PTA) adjusted to pH 7.4 with NaOH. All the TEM analyses were performed with dried samples. The nanoparticle size distribution was characterized by DLS analyses (DLS PN3704 Zetasizer, Malvern Panalytical) using a copolymer concentration of 0.20 mg/mL. For each analysis, 3 measurements were carried out, based on 11 runs. Samples were analyzed at 25 °C with a scattering angle of 173° and a 633 nm HeNe laser, based on a material refractive index (RI) of 1.59, a dispersant refractive index of 1.330, and a viscosity of 0.89. Drug encapsulation was measured by RP-HPLC (Dionex UltiMate 3000, ThermoFisher Scientific). A gradient of water + TFA 0.05% (A) and MeOH + TFA 0.05% (B) from 0 min (30% B) to 30 min (100% B) was used to run the samples through a Jupiter 5 μm C18 300 Å, 150 × 4.6 mm column (Phenomenex, Torrance, CA). The peak area was integrated by using Chromeleon version 6.8 (ThermoFisher Scientific).

### *In Vitro* Cellular Cytotoxicity Assays

Cell viability was evaluated on human monocytes (THP-1 cell line), human lung fibroblasts (HLF cell line), and mouse alveolar-like macrophages (MPI-2 cell line) using the thiazolyl blue tetrazolium bromide method (MTT; chemicals acquired from Sigma-Aldrich, Merck KGaA).

First, THP-1 cells were seeded at a concentration of 5 × 10^4^ cells/well in a 96-well plate and differentiated into macrophages by incubation with 8 ng/mL of phorbol 12-myristate 13-acetate (PMA, Sigma-Aldrich, Merck KGaA) for 72 h. Instead, HLF cells were seeded at 10^5^ cells/well in a 96-well plate and incubated for 24 h. Then, the cell culture media was removed, and the cells were incubated with increasing amounts of the test solutions (**1e**, **2ea**, **2eb**, **POs2ea**, **POs2eb**) diluted in the growth media for 24 or 48 h. After incubation, the medium was aspirated, and 100 μL of MTT solution (0.5 mg/mL in PBS) was added to each well and left for 3 h. Intracellular metabolic activity reduced MTT to a purple formazan salt. Subsequently, the solution was aspirated, and the insoluble formazan product was solubilized by adding DMSO. The solubilized blue crystals were measured colorimetrically at 570 nm using a Tecan Spark microplate reader (Tecan Group Ltd., Männedorf, Switzerland).

### *Mtb* Cultures

*Mtb* cells were cultured at 37 °C in Middlebrook 7H9 media (Sigma-Aldrich, Merck KGaA) supplemented with 10% OADC (ThermoFisher Scientific), 0.5% glycerol and 0.05% Tween-80. Exponentially growing cultures were started from a single colony, aliquots were enriched with 15% glycerol, stored at −80 °C, and used once to start primary cultures. Before the infection assay, *Mtb* H37Rv was grown in Middlebrook 7H9 complete media at 37 °C in shaking conditions to mid log phase (OD_600_ = 0.6–0.8). A selective antibiotic was only added for the strains carrying chromosomal integrative vectors in the primary culture and removed in the culture used for the final experiments. The constitutive reporter strain used in the following study was generated using the integrative vector pTdTomato-L5 (Addgene #140994). *Mtb* was transformed with the construct by electroporation (2500 V, 25 μF, 1000 Ω, 2 mm path), and plated on Middlebrook 7H10 agar containing Streptomycin (Sigma-Aldrich, Merck KGaA) at a concentration of 30 μg/mL.

### Cell Cultures

Cell lines were incubated at 37 °C in a humidified atmosphere containing 5% CO_2_. All media were supplemented with 10% fetal bovine serum (ThermoFisher Scientific), 100 U/mL of penicillin (ThermoFisher Scientific), 0.1 mg/mL streptomycin, and 0.25 μg/mL amphotericin B (ThermoFisher Scientific). Max Planck Institute cell-2 (MPI-2, kindly provided by Marina Freudenberg, Max Planck Institute of Immunology and Epigenetics, Freiburg, Germany) were cultured in Roswell Park Memorial Institute (RPMI) 1640 medium (ThermoFisher Scientific) supplemented with 30 ng/mL of murine granulocyte-macrophage colony-stimulating factor (GM-CSF; Preprotech, Cranbury, NJ). MPI-2 cells were passaged twice weekly at a concentration of 2 × 10^5^ cells/mL. Cells from passages 8 (p8) to 70 (p70) were used. Lastly, MPI-2 cells were routinely tested for contamination by mycoplasma.

### Infection Assay with *Mtb*

MPI-2 cells were seeded in black clear-bottom 96-well plates at a density of 2 × 10^4^ cells per well. After 24 h, they were infected with an *Mtb* suspension (MOI = 1:5) for 4 h. Then, the cells were extensively washed with PBS (ThermoFisher Scientific) and resuspended in 100 μL of RPMI 1640 supplemented with 30 ng/mL of murine GM-CSF. The infected cells were treated with decreasing concentrations of **2ea**, **2eb**, **POs2ea**, or **POs2eb** ranging from 100 to 25 μM. 1, 2, and 3 days postinfection *Mtb* fluorescence was measured using a Spark microplate reader (Tecan).

### Cy5–PMPC-PDPA Uptake

MPI-2 cells were seeded in an 8-well Ibidi plate at a density of 4 × 10^4^ cells per well. After 24 h, the cells were incubated with Cy5-**POs2ea** and Cy5-**POs2eb** at a final compound concentration of 100 μM at diverse time points. At each time, cells were fixed with 100 μL of 4% PFA (Santa Cruz Biotechnology, Dallas, TX) for 10 min. Fixed cells were then washed once with PBS. MPI-2 membrane was stained using CellMask Deep Red dye (1:1000) (ThermoFisher Scientific) for 10 min. Cells were quickly washed and finally resuspended in 100 μL of PBS.

Cy5-**POs2ea** and Cy5-**POs2eb** uptake in MPI-2 cells infected with *Mtb* was analyzed by seeding MPI-2 cells in an 8-well Ibidi plate at a density of 4 × 10^4^ cells per well. After 24 h, MPI-2 cells were infected with *Mtb* (MOI 1:5) for 4 h; then, extracellular bacteria were removed by extensively washing the cells. Finally, infected MPI-2 cells were incubated with Cy5-**POs2ea** and Cy5-**POs2eb** at a final compound concentration of 100 μM at diverse time points. At each time, cells were fixed with 100 μL of 4% PFA (Santa Cruz Biotechnology) for 20 min. Fixed cells were then washed once with PBS. MPI-2 membrane was stained using CellMask Deep Red dye (1:1000) (ThermoFisher Scientific) for 10 min. Cells were quickly washed and finally resuspended in 100 μL of PBS.

For the microscopy analysis, cells were imaged with a Stellaris 8 Confocal Microscope (Leica Microsystems, Wetzlar, Germany). The image analysis and segmentation were performed using Nis-Element v 5.41 (Nikon, Tokyo, Japan), creating a binary mask on the CellMask channel. Then, a threshold adjustment was applied. Next, a CellMask mask was used to segment the cells, and Cy5 mean fluorescence was measured.

### Real-Time Quantitative PCR

Four ×10^5^ MPI-2 were seeded in a 32 mm dish and incubated at 37 °C with 5% CO_2_ for 24 h. Then, samples were either infected or not with *Mtb* for 4 h. The cells were abundantly washed with PBS, and RNA extraction was performed using Monarch Total RNA Miniprep Kit (New England Biolabs, Ipswich, MA), following the manufacturer’s instructions. Total RNA was eluted in 30 μL, quantified using a NanoDrop One/OneC Microvolume UV–vis Spectrophotometer (ThermoFisher Scientific), and stored at −80 °C. cDNA was generated starting from 150 ng of RNA using ProtoScript II First Strand cDNA Synthesis Kit (New England Biolabs), using random hexamers according to the manufacturer’s instructions. The murine primers were the following: *cd36* (f: GGAATCCACTATCCATACCCAGG; r: CTCTTCACCAGACAGCAGGAGA), *srb1* (f: ACACCCGAATCCTCGCTGGAAT; rCCGTTGGCAAACAGAGTATCGG:), *cd206* (f: GTTCACCTGGAGTGATGGTTCTC; r: AGGACATGCCAGGGTCACCTTT), *cd81* (f: CCACCATACTGAGGAACAGCCT; r: GCTACCACAATGGCTGCAATTCC), and gapdh (f: CATCACTGCCACCCAGAAGACTG; r: ATGCCAGTGAGCTTCCCGTTCAG), were used. qRT-PCRs were carried out using a Luna Universal One-Step RT-qPCR Kit (New England Biolabs), 0.3 μM primers, and 1 μL of cDNA diluted 1:4. Gapdh-relative quantification was run on Quant Studio Real-Time PCR System (ThermoFisher Scientific). Gene expression was measured using the 2^–ΔCt^ method for each technical triplicate.

### Statistics

Plots and statistical analysis were generated using Prism 10 (GraphPad Software). Two-way ANOVA, followed by correction for multiple comparisons, was performed to analyze the statistical significance between multiple groups. The plots aggregate data deriving from at least two independent replicates. Significant *p*-values, sample size, and statistical tests are reported in the legends.

## Supporting Information Available

Additional synthetic information, analytical data, biological evaluation, molecular modeling studies, HPLC chromatograms of polymersomes, DLS analysis of polymersomes, TEM images of polymersomes, and cytotoxicity assays of the free and encapsulated compounds (PDF)
Molecular formula strings (CSV)
Crystallographic information file (CIF)

## Supplementary Material

jm4c02386_si_002.csv

jm4c02386_si_003.cif

## Abbreviations Used

AM: alveolar macrophage
ATR: attenuated total reflectance
ATRP: atom transfer radical polymerization
BCG: Bacillus Calmette–Guérin
bpy: 2,2′-bipyridine
CAS: chrome azurol S
CD206: cluster of differentiation 206
CD36: cluster of differentiation 36
CD87: cluster of differentiation 87
CES1: carboxylesterase 1
CG: conjugate gradient
COVID-19: Coronavirus disease 2019
Ctrl: control
Cy5: Cyanine5
DLS: dynamic light scattering
DPA: 2(diisopropylamino)ethyl methacrylate
EtOAc: ethyl acetate
FT-IR: Fourier transform infrared spectroscopy
GAFF: general Amber force field
GAPDH: glyceraldehyde-3-phosphate dehydrogenase
GPC: gel permeation chromatography
HLF: human lung fibroblast
HS: Hirshfeld surface
mAM: murine alveolar macrophage
ME-Br: 2-(4-morpholino)ethyl 2-bromoisobutyrate
MeCN: acetonitrile
MeOH: methanol
MOI: multiplicity of infection
MPC: 2-methacryloyloxyethyl phosphorylcholine
MPI-2: Max Planck Institute 2
*Mtb*: *Mycobacterium tuberculosis* (also *M*. *tuberculosis*)
MWCO: molecular weight cutoff
OADC: oleic albumin dextrose catalase
PDPA: poly(2-(diisopropylamino)ethyl methacrylate)
PFA: paraformaldehyde
PME: particle mesh Ewald
PMPC: poly(2-methacryloyloxyethyl phosphorylcholine)
PO: polymersome
PTA: phosphotungstic acid
qRT-PCR: quantitative reverse transcription polymerase chain reaction
RA: residual activity
REMA: resazurin microtiter assay
RI: refractive index
Rif: rifampicin
RP-HPLC: reversed-phase high-performance liquid chromatography
RPMI: Roswell Park Memorial Institute
SC-XRD: single-crystal X-ray diffraction
SD: steepest descent
SR-B1: scavenger receptor class B type 1
TEM: transmission electron microscopy
UHPLC: ultrahigh-performance liquid chromatography
WHO: World Health Organization
