# Supplementary material for: Nanoenabling MbtI Inhibitors for Next-Generation Tuberculosis Therapy
Source: J Med Chem. 2025 Mar 3;68(5):5312–32. doi: 10.1021/acs.jmedchem.4c02386 (PMC11912484; doi:10.1021/acs.jmedchem.4c02386)
Supplement: Supplementary file 1 — jm4c02386_si_001.pdf [file jm4c02386_si_001.pdf]

Supporting Information

Nano-enabling MtbI inhibitors for next-generation tuberculosis therapy

Giulia Cazzaniga[1,2,†], Matteo Mori[1,†], Anna Griego[1,3], Edoardo Scarpa[1,3], Giorgia Moschetti[1,3], Stefano Muzzioli[1,3], Giovanni Stelitano[4], Laurent R. Chiarelli[4], Mario Cocorullo[4], Emanuele Casali[5], Alessio Porta[5], Giuseppe Zanoni[5], Andrea Tresoldi[1], Elena Pini[1], Íris L. Batalha[6], Giuseppe Battaglia[7,8], Tiziano Tuccinardi[9], Loris Rizzello[1,3], Stefania Villa[1,*], Fiorella Meneghetti[1]

[1] Department of Pharmaceutical Sciences, University of Milan, Via L. Mangiagalli 25, 20133 Milano, Italy
[2] Department of Science and High Technology, University of Insubria, via Valleggio 9, 22100, Como, Italy
[3] National Institute of Molecular Genetic (INGM), Via F. Sforza 35, 20122 Milano, Italy
[4] Department of Biology and Biotechnology "Lazzaro Spallanzani", University of Pavia, via A. Ferrata 9, 27100 Pavia, Italy
[5] Department of Chemistry, University of Pavia, Viale T. Taramelli 12, 27100 Pavia, Italy
[6] Department of Life Sciences, University of Bath, Claverton Down, BA2 7AY, Bath, UK
[7] Molecular Bionics Group, Institute for Bioengineering of Catalonia (IBEC), C. Baldiri Reixac 10-12, 08028 Barcelona, Spain
[8] Catalan Institution of Research and Advanced Studies, (ICREA), Passeig de Lluís Companys, 23, 08010 Barcelona, Spain
[9] Department of Pharmacy, University of Pisa, Via Bonanno Pisano 6, 56126 Pisa, Italy

†These authors contributed equally.
*Corresponding author: stefania.villa@unimi.it

Table of contents

1. Additional synthetic information	S2
2. Analytical data	S3
3. Biological evaluation	S64
4. Molecular modeling studies	S66
5. HPLC chromatograms of polymersomes	S67
6. DLS analysis of polymersomes	S68
7. TEM images of polymersomes	S69
8. Cytotoxicity assays on the free and encapsulated compounds	S70

1. Additional synthetic information

- *Synthesis of (2E,4E)-1-bromohexa-2,4-diene*

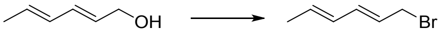

Scheme S1. Reagents and conditions: PBr_3, DCM, -10 °C, 3 h, N_2 atm.

(2*E*,4*E*)-Hexa-2,4-dien-1-ol (500 mg, 5.09 mmol) was dissolved in 1 mL of anhydrous DCM under nitrogen atmosphere, and the solution was cooled to −10 °C. Then, a solution of PBr_3 (468 mg, 1.73 mmol) in anhydrous DCM (1 mL) was slowly added dropwise, keeping the temperature at −10 °C. After 3 h, the reaction was quenched with a saturated solution of $NaHCO_3$ (10 mL). The aqueous layer was extracted with Et_2O (3 x 10 mL), and the combined organic layers were dried over anhydrous Na_2SO_4, filtered, and concentered *in vacuo* to afford the product as a light brown oil, which was directly used in the next synthetic step without further purification (poor stability). TLC (EtOAc/hexane) R_f: 0.71.

- *Synthesis of (2E,4E,6E)-1-bromoocta-2,4,6-triene*

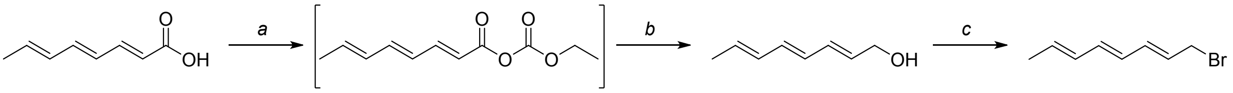

Scheme S2. Reagents and conditions: *a)* Ethyl chloroformate, TEA, DMA, THF, 0 °C, 1 h, N_2 atm.; *b)* $NaBH_4$, H_2O, THF, −15/−20 °C → RT, overnight; c) PBr_3, CCl_4, 0 °C, 30 min, N_2 atm.

<u>(2*E*,4*E*,6*E*)-Octa-2,4,6-trien-1-ol</u>

The procedure was slightly modified from a patent by Giuliani G., Benedusi A., and Milanese A. (WO2010119117). In a round-bottom flask purged with nitrogen, all-*trans* octatrienoic acid (1 g, 7.24 mmol) was dissolved in 15 mL of anhydrous THF and 7.5 mL of DMA to give a clear yellow solution. Then, 1 mL of TEA was added dropwise. The reaction mixture was cooled to 0 °C on an ice-water bath, and ethyl chloroformate (0.69 mL, 7.24 mmol) was slowly added (the reaction is exothermic). After stirring for 1 h at 0 °C, the formed precipitate of TEA·HCl was filtered on a Büchner funnel under a nitrogen flow and washed with THF. The filtrate was directly charged in a round-bottom flask and cooled to −15/−20 °C with dry ice in acetone (do not cool below −20 °C to prevent the freezing of the $NaBH_4$ solution). Then, 1 mL of a $NaBH_4$ (410 mg, 10.83 mmol) solution in basic water (obtained by solubilizing 0.1 g NaOH in 25 mL of H_2O) was slowly added dropwise (the reaction is extremely exothermic). The mixture was gradually warmed to room temperature and stirred overnight. After completion, the reaction was quenched with a saturated solution of $NaHCO_3$, and THF was concentrated under reduced pressure. The aqueous phase was extracted with diethyl ether (3 x 30 mL), and the combined organic layers were dried over Na_2SO_4, filtered, and concentrated under vacuum. The crude was purified by recrystallization using petroleum ether to afford the desired product as a white solid. Yield: 58%. TLC (hexane/EtOAc 7:3) R_f: 0.39. ^1H NMR (300 MHz, $CDCl_3$): δ (ppm) 6.30-6.04 (m, 4H, CH), 5.85-5.68 (m, 2H, CH), 4.19 (d, J = 6.4 Hz, 2H, CH_2), 1.80 (d, J = 6.4 Hz, 3H, CH_3), 1.38 (s exch D_2O, 1H, OH).

<u>(2*E*,4*E*,6*E*)-1-Bromoocta-2,4,6-triene</u>

To a stirred solution of (2*E*,4*E*,6*E*)-octa-2,4,6-trien-1-ol (100 mg, 0.81 mmol) in 12 mL of anhydrous CCl_4, a solution of PBr_3 (72 mg, 0.265 mmol, 0.025 mL) in 1 mL of anhydrous CCl_4 was added dropwise at 0 °C under a nitrogen flow. After 30 min, a saturated aqueous solution of $NaHCO_3$ was added; the aqueous layer was extracted with diethyl ether (3 x 10mL), and the combined organic layers were dried over Na_2SO_4, filtered, and evaporated under vacuum to afford the product as a yellow oil, which was directly used for the next synthetic step without further purification (poor stability). TLC (EtOAc/hexane) R_f: 0.50.

2. Analytical data

- *5-(3-Cyano-5-ethoxyphenyl)furan-2-carboxylic acid (**1a**)*

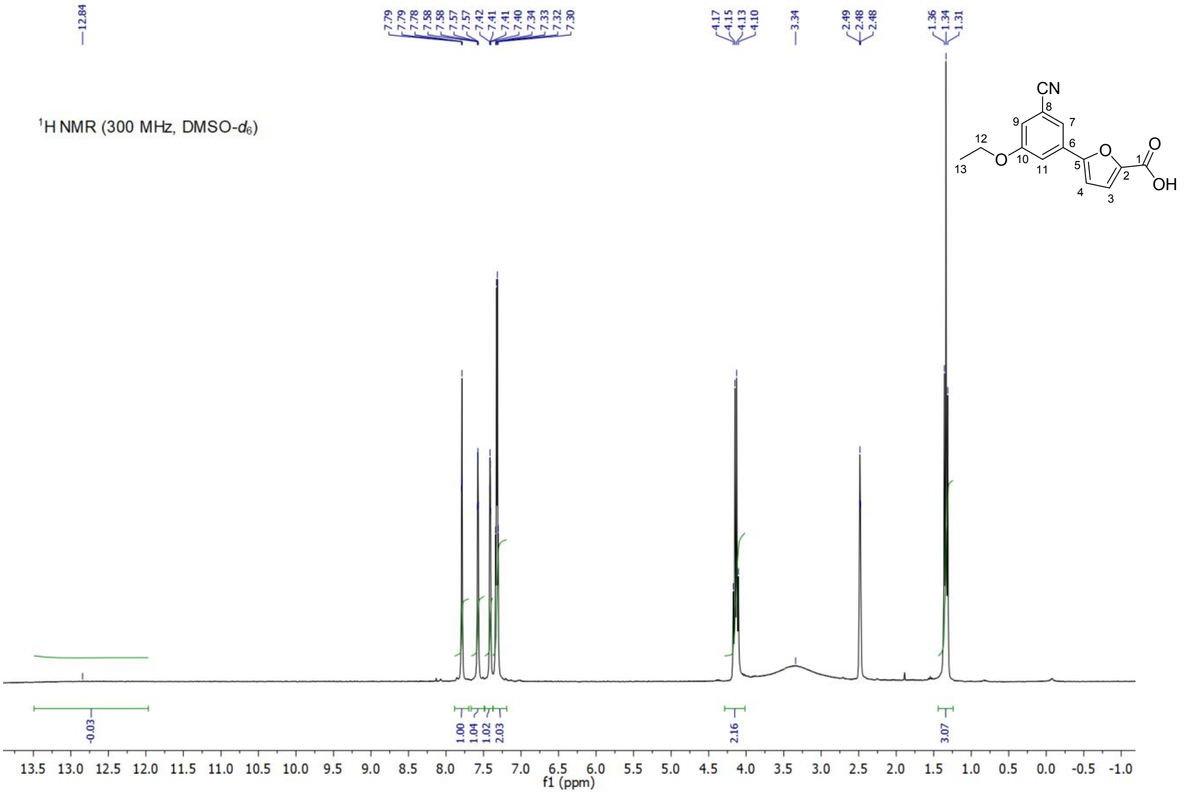

Figure S1. ^{1}H NMR spectrum of **1a**.

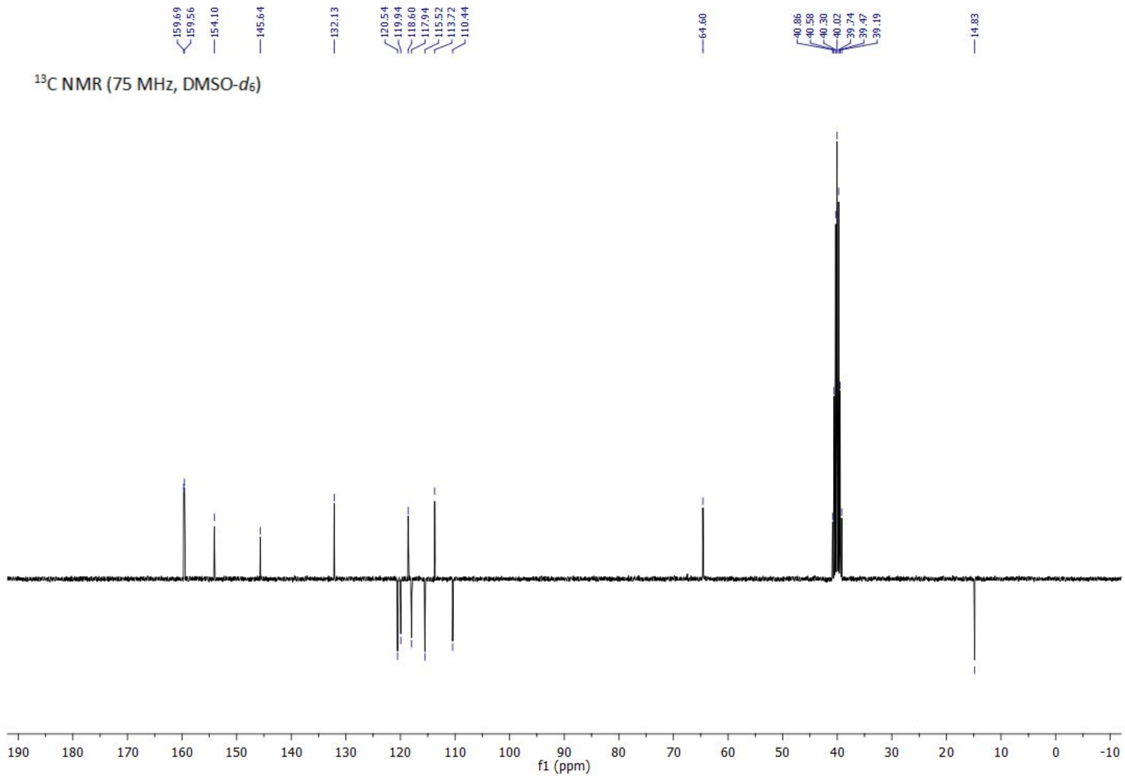

Figure S2. ^{13}C NMR spectrum of **1a**.

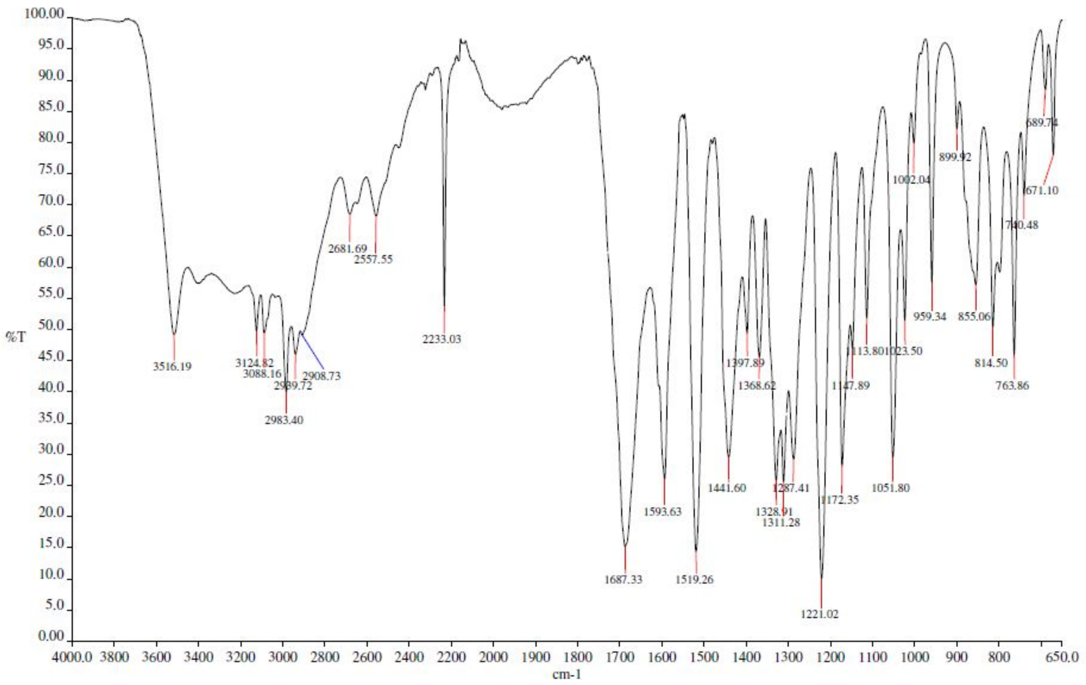

Figure S3. FT-IR spectrum of **1a**.

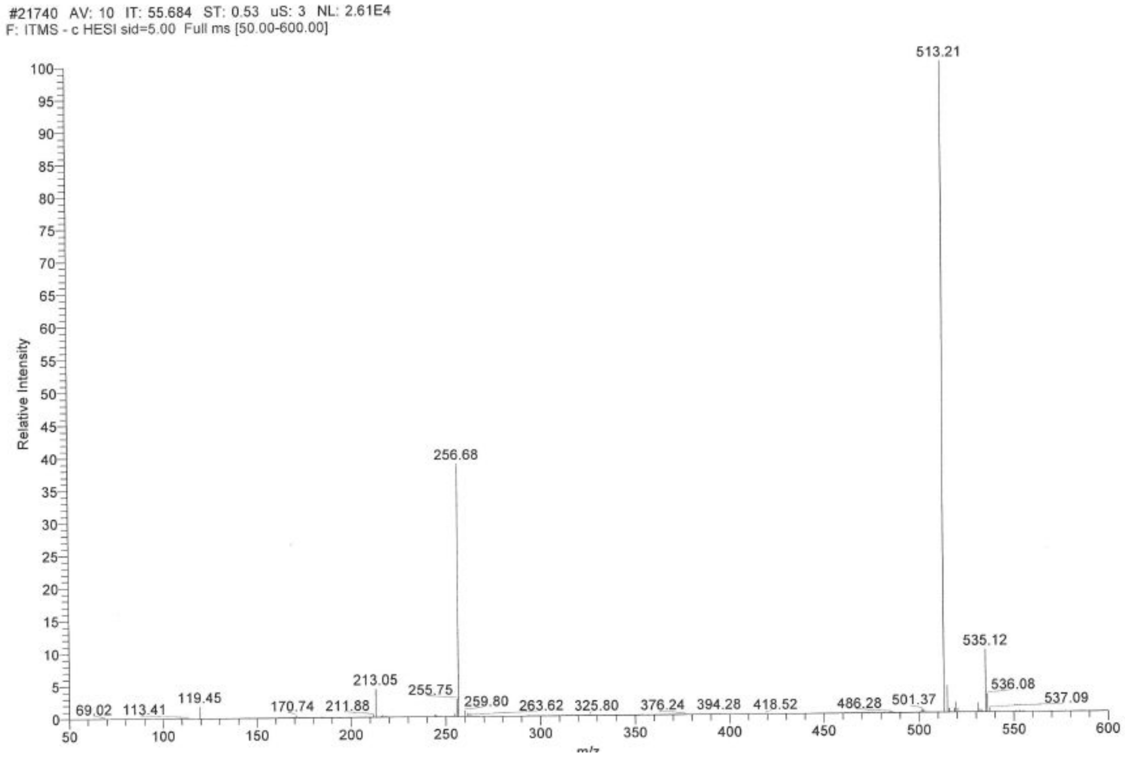

Figure S4. ESI-MS spectrum of **1a**.

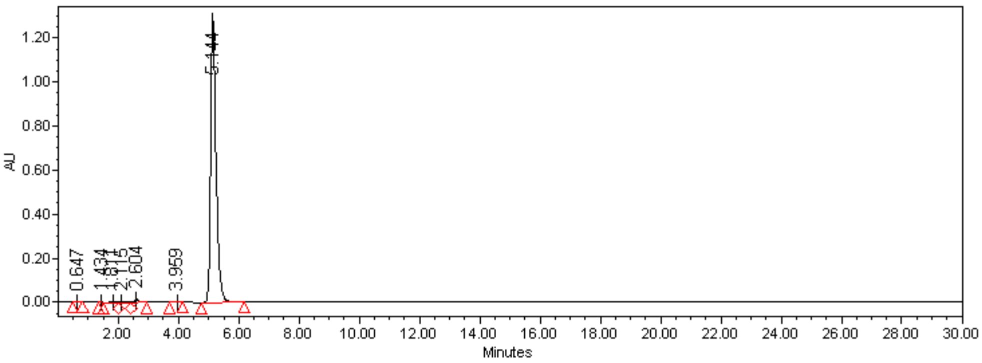

	Name	Retention Time (min)	Area (µV*sec)	% Area	Height (µV)	Int Type	Amount	Units	Peak Type	Peak Codes
1		0.647	10562	0.06	1081	BB			Unknown	
2		1.434	6281	0.04	1205	BB			Unknown	
3		1.811	51293	0.32	3709	BV			Unknown	
4		2.115	37171	0.23	1530	VV			Unknown	
5		2.604	122340	0.75	12697	VB			Unknown	
6		3.959	9480	0.06	759	BB			Unknown	
7		5.144	16042898	98.54	1282390	BB			Unknown	

Figure S5. HPLC chromatogram of **1a**. Operative conditions: sample amount: 10 µg; column: Phenomenex Luna® 3 µM C18(2) 100 Å, 4.6x100 mm; eluent system: water/MeOH + 0.05% TFA 30:70; flow rate: 1 mL/min; detector λ: 254 nm; time: 30 min; temperature: 23 °C.

- *5-(3-Cyano-5-isopropoxyphenyl)furan-2-carboxylic acid (**1b**)*

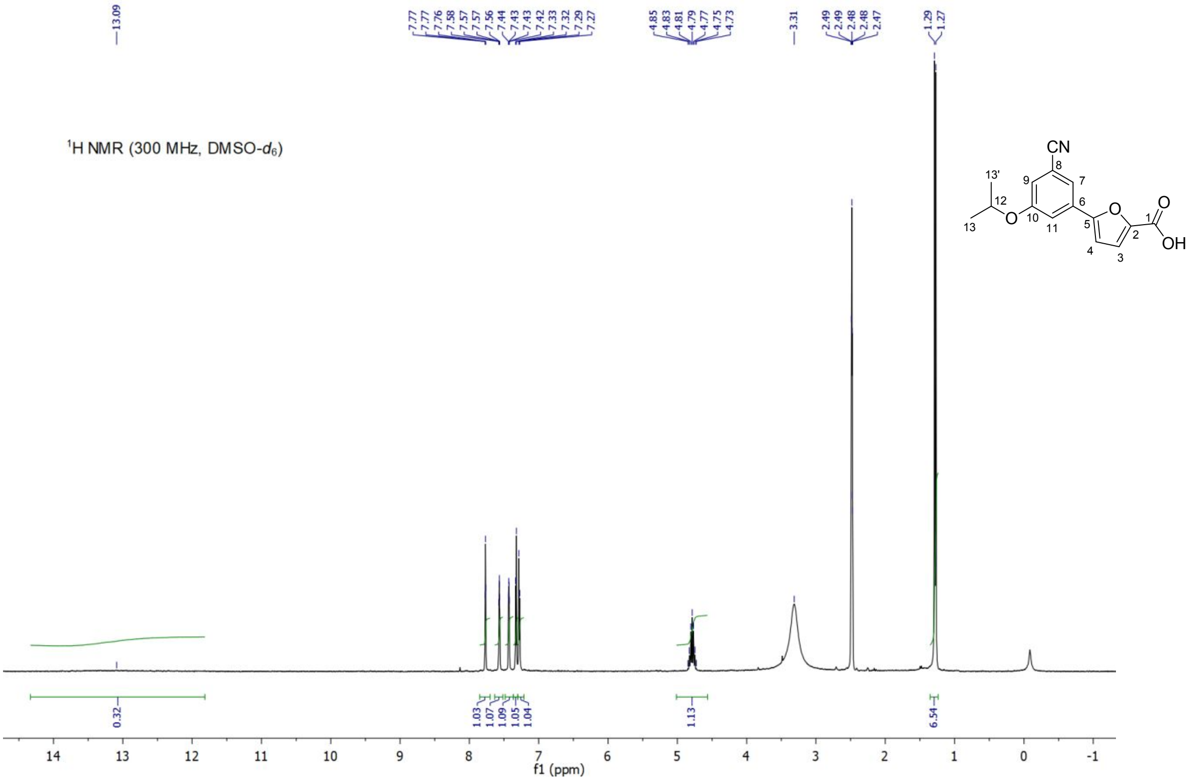

Figure S6. ^{1}H NMR spectrum of **1b**.

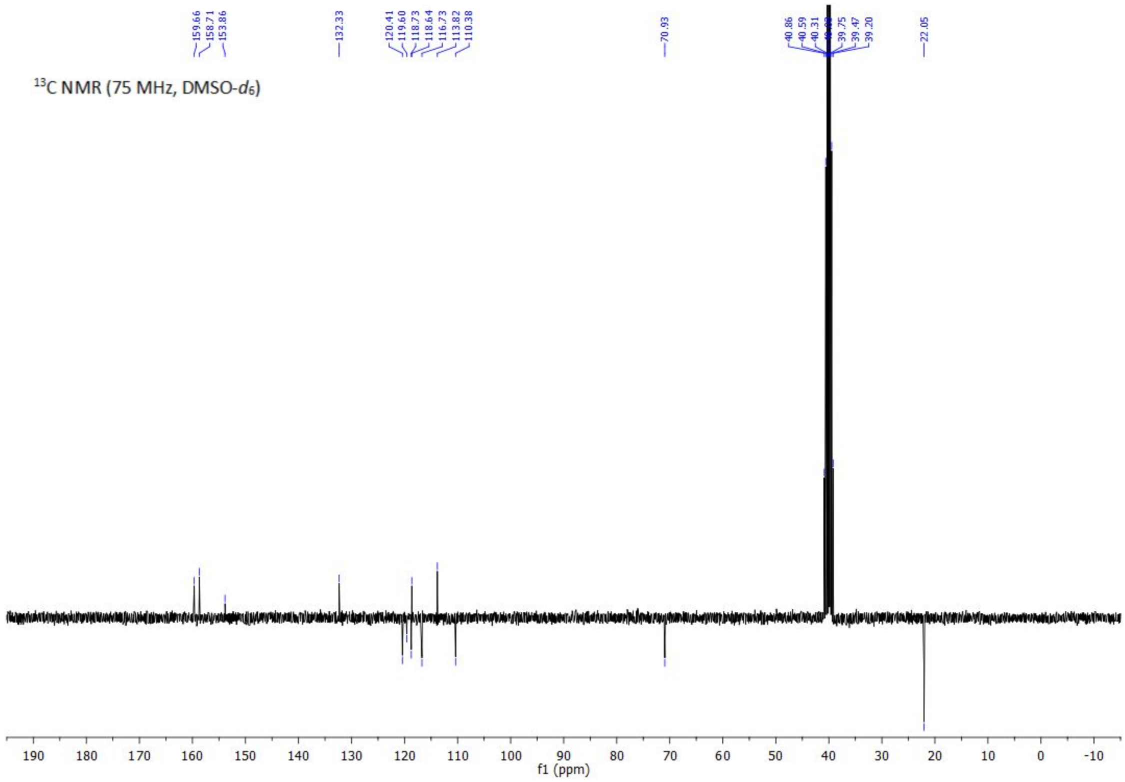

Figure S7. ^{13}C NMR spectrum of **1b**.

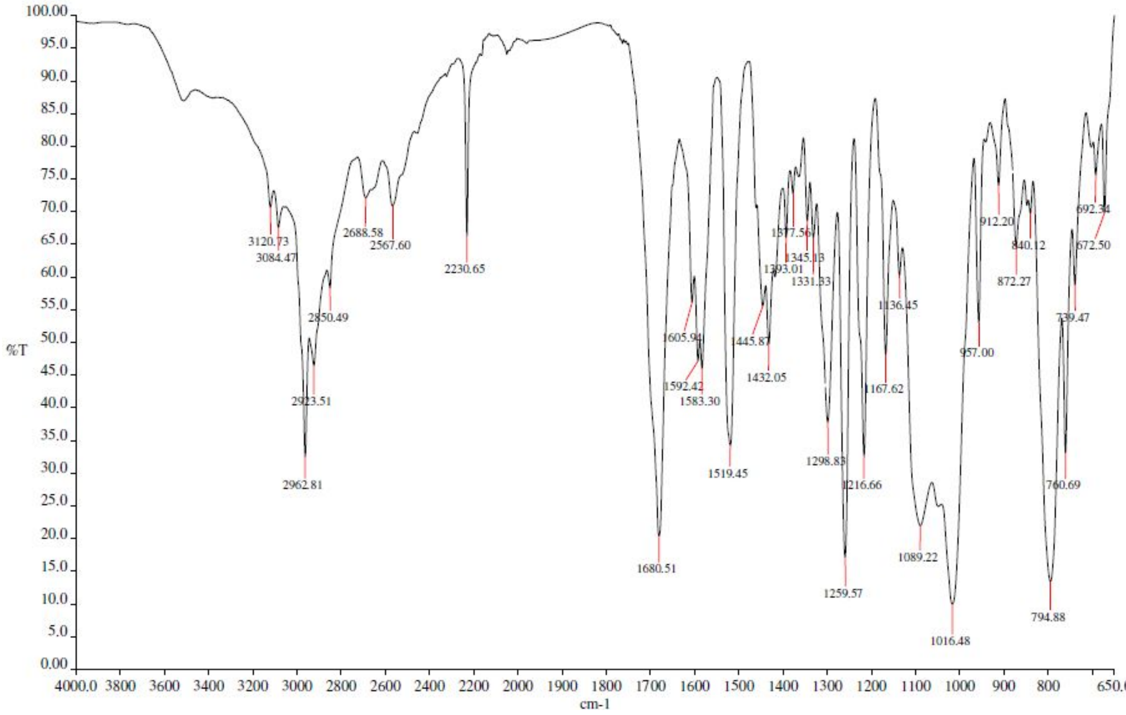

Figure S8. FT-IR spectrum of **1b**.

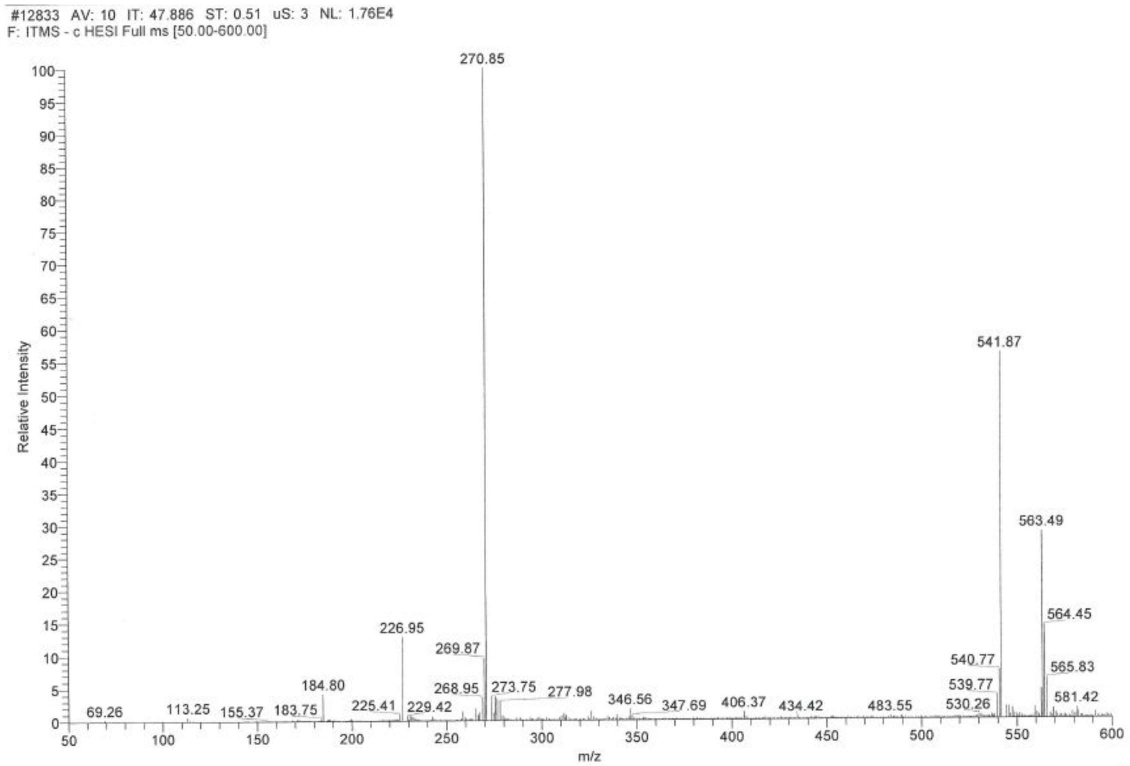

Figure S9. ESI-MS spectrum of **1b**.

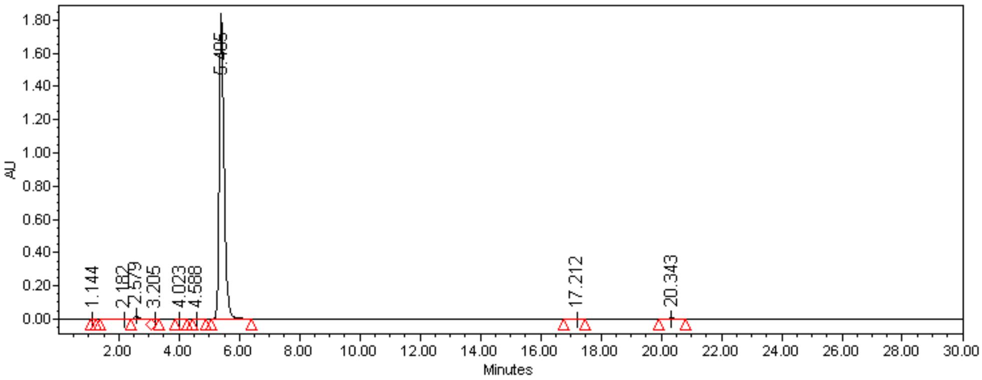

	Name	Retention Time (min)	Area (µV*sec)	% Area	Height (µV)	Int Type	Amount	Units	Peak Type	Peak Codes
1		1.144	5744	0.03	880	BB			Unknown	
2		2.182	118240	0.59	2438	BB			Unknown	
3		2.579	216278	1.07	23208	BV			Unknown	
4		3.205	6500	0.03	681	VB			Unknown	
5		4.023	10954	0.05	1268	BB			Unknown	
6		4.588	7049	0.04	558	BB			Unknown	
7		5.405	19674737	97.76	1801588	BB			Unknown	
8		17.212	22749	0.11	972	BB			Unknown	
9		20.343	63793	0.32	2657	BB			Unknown	

Figure S10. HPLC chromatogram of **1b**. Operative conditions: sample amount: 10 µg; column: Phenomenex Luna® 3 µM C18(2) 100 Å, 4.6x100 mm; eluent system: water/MeOH + 0.05% TFA 30:70; flow rate: 1 mL/min; detector λ: 254 nm; time: 30 min; temperature: 23 °C.

- *5-(3-Cyano-5-propoxyphenyl)furan-2-carboxylic acid (**1c**)*

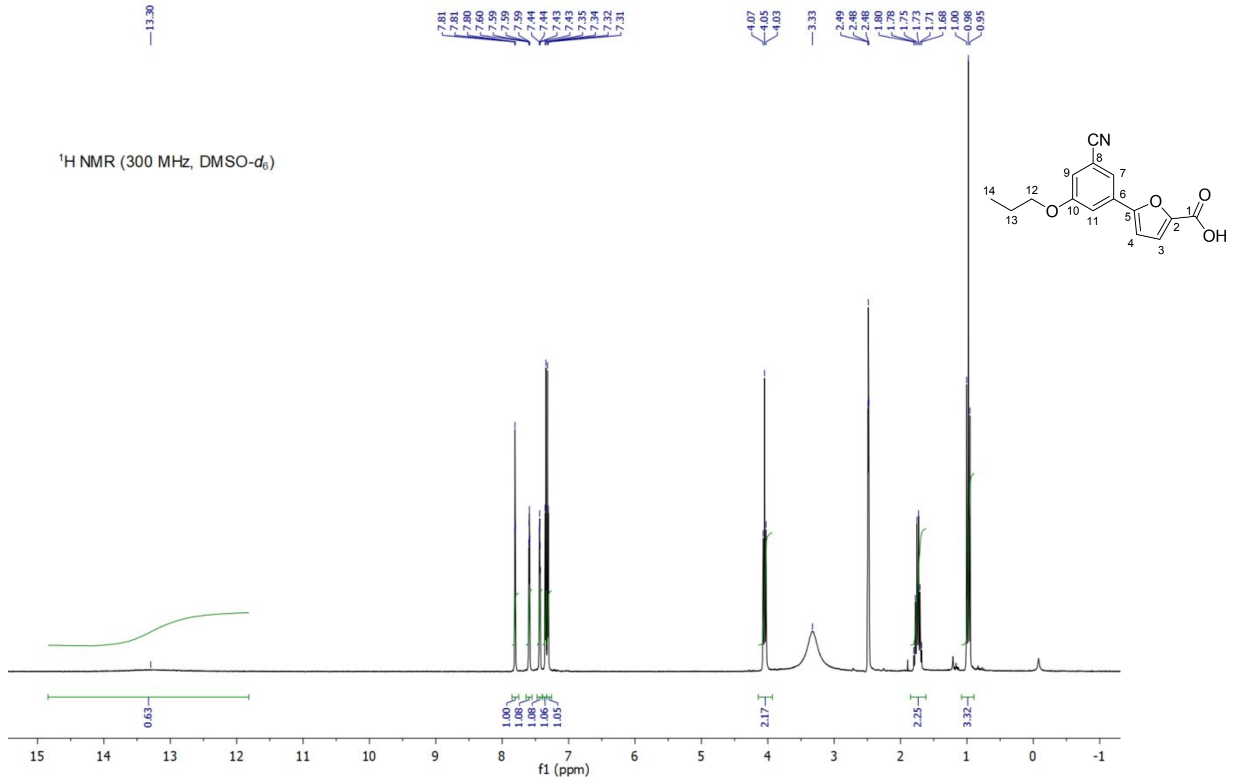

Figure S11. ^{1}H NMR spectrum of **1c**.

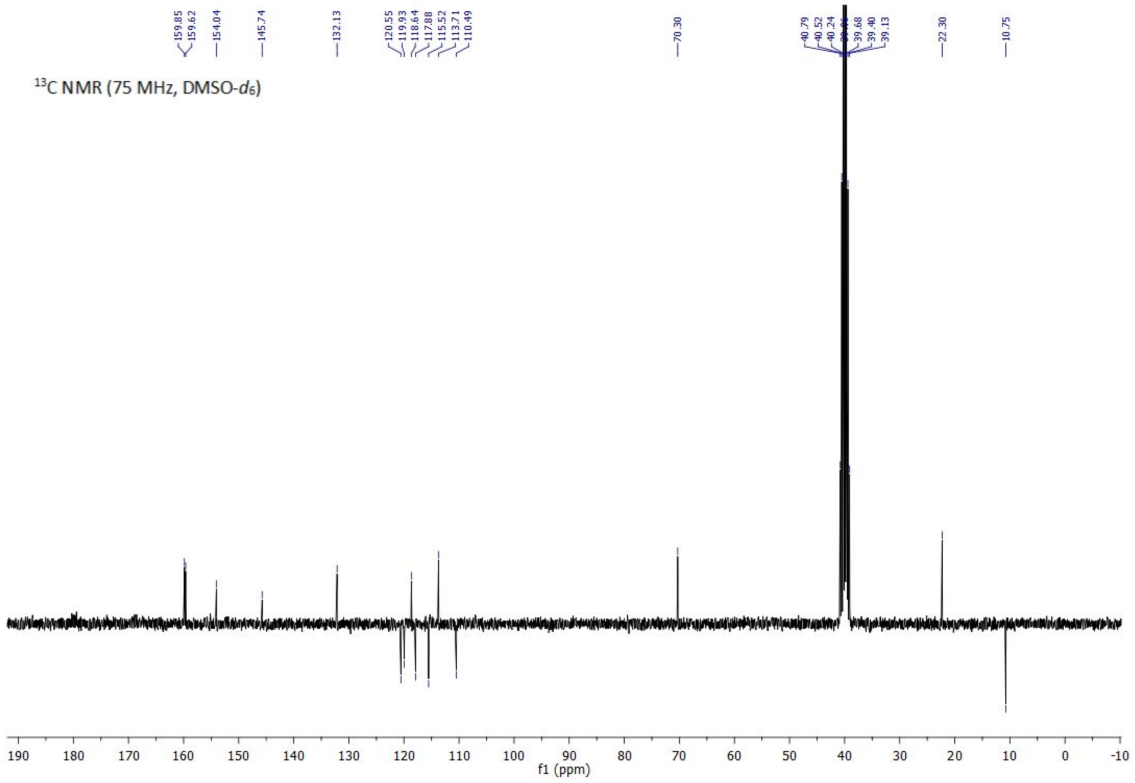

Figure S12. ^{13}C NMR spectrum of **1c**.

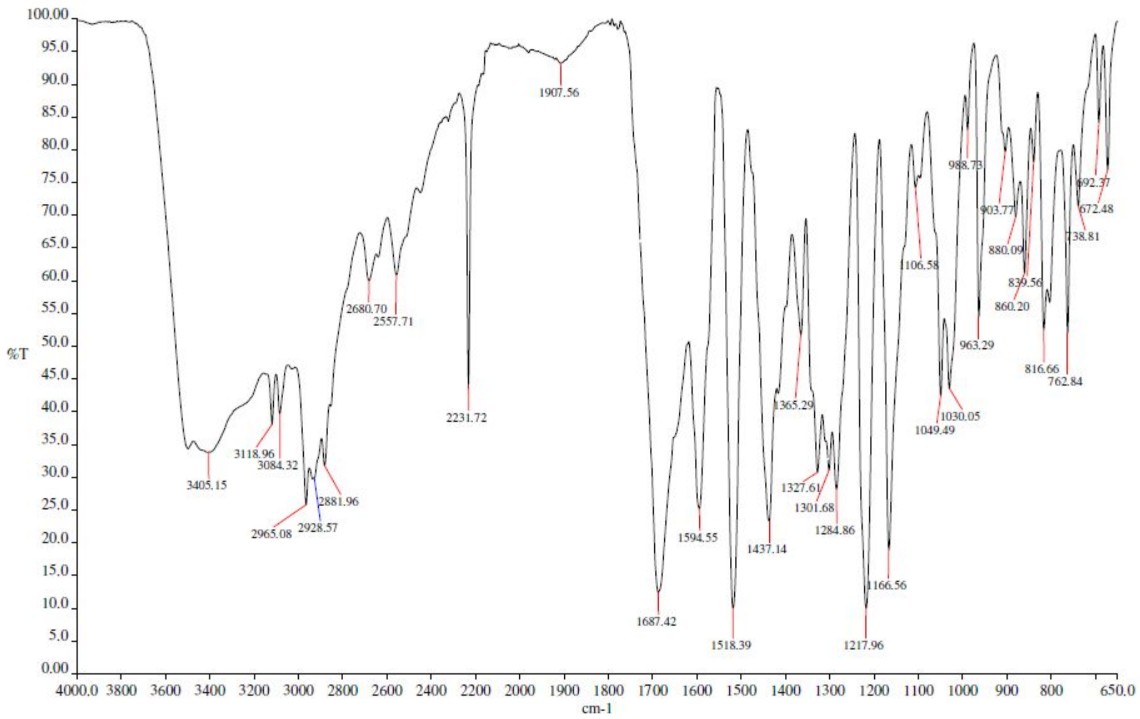

Figure S13. FT-IR spectrum of **1c**.

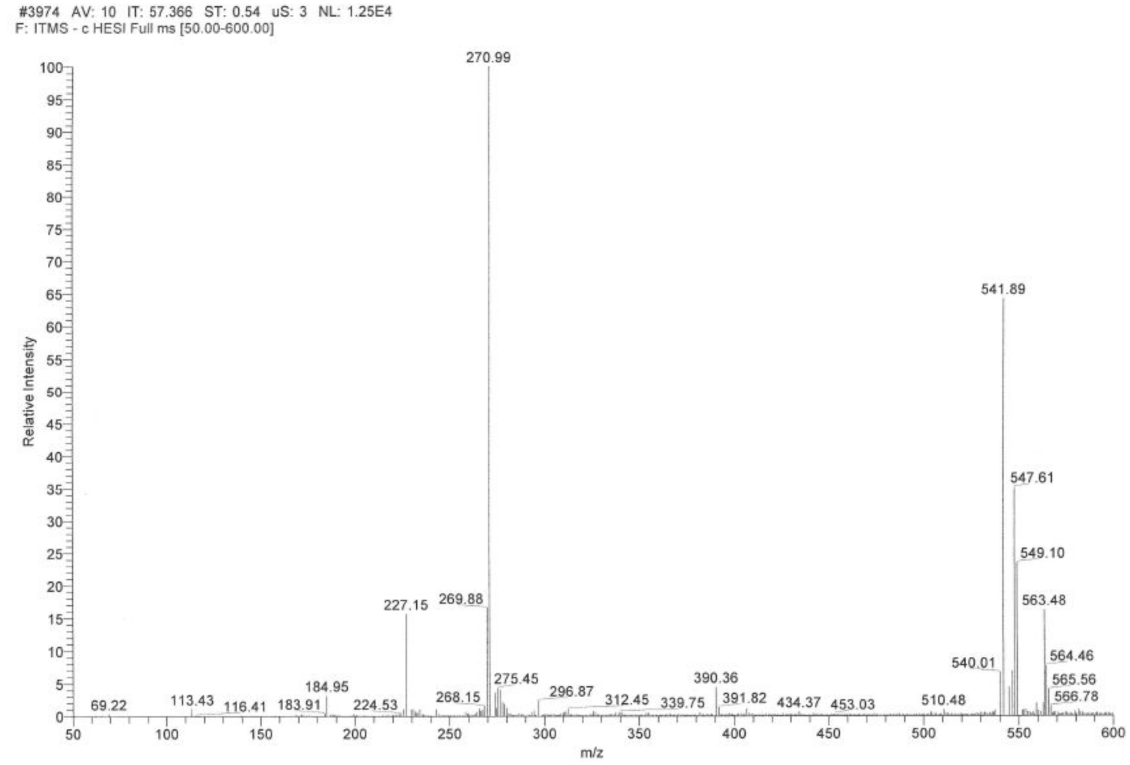

Figure S14. ESI-MS spectrum of **1c**.

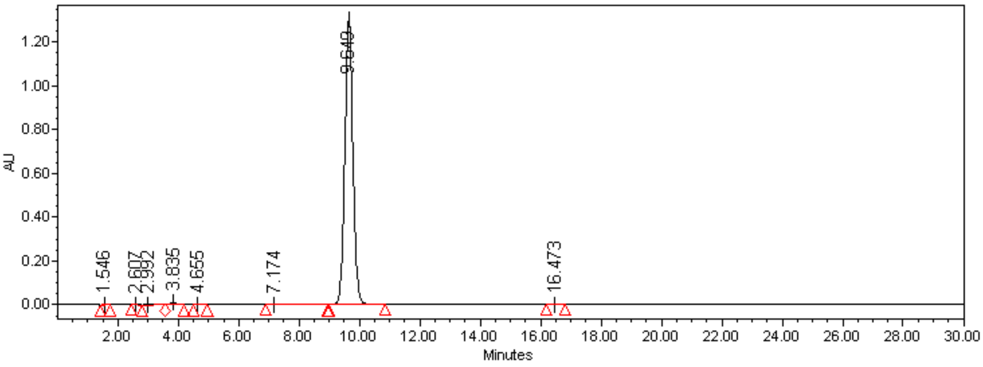

	Name	Retention Time (min)	Area (µV*sec)	% Area	Height (µV)	Int Type	Amount	Units	Peak Type	Peak Codes
1		1.546	12162	0.05	1639	BB			Unknown	
2		2.607	43040	0.17	4274	BB			Unknown	
3		2.992	62570	0.25	1856	BV			Unknown	
4		3.835	142146	0.58	12842	VB			Unknown	
5		4.655	10537	0.04	844	BB			Unknown	
6		7.174	45866	0.19	840	BB			Unknown	
7		9.649	24301399	98.61	1303034	BB			Unknown	
8		16.473	25408	0.10	1432	BB			Unknown	

Figure S15. HPLC chromatogram of **1c**. Operative conditions: sample amount: 10 µg; column: Phenomenex Luna® 3 µM C18(2) 100 Å, 4.6x100 mm; eluent system: water/MeOH + 0.05% TFA 30:70; flow rate: 1 mL/min; detector λ: 254 nm; time: 30 min; temperature: 23 °C.

- *5-(3-Butoxy-5-cyanophenyl)furan-2-carboxylic acid (**1d**)*

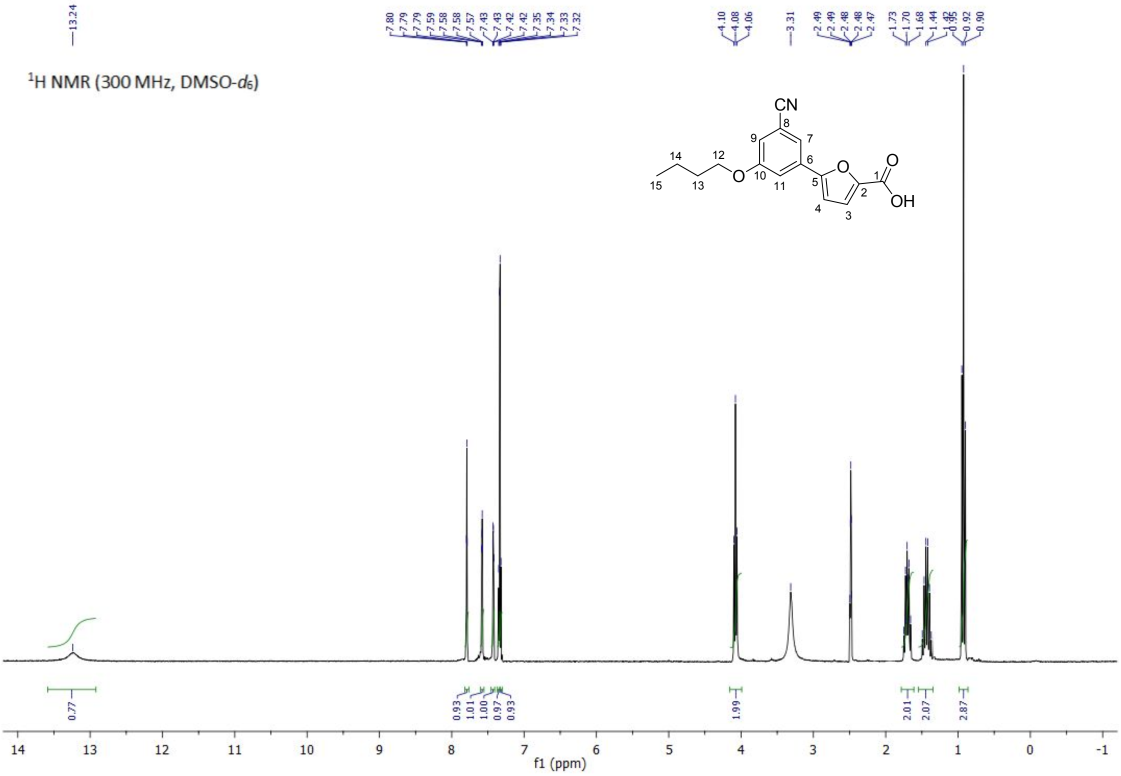

Figure S16. ^{1}H NMR spectrum of **1d**.

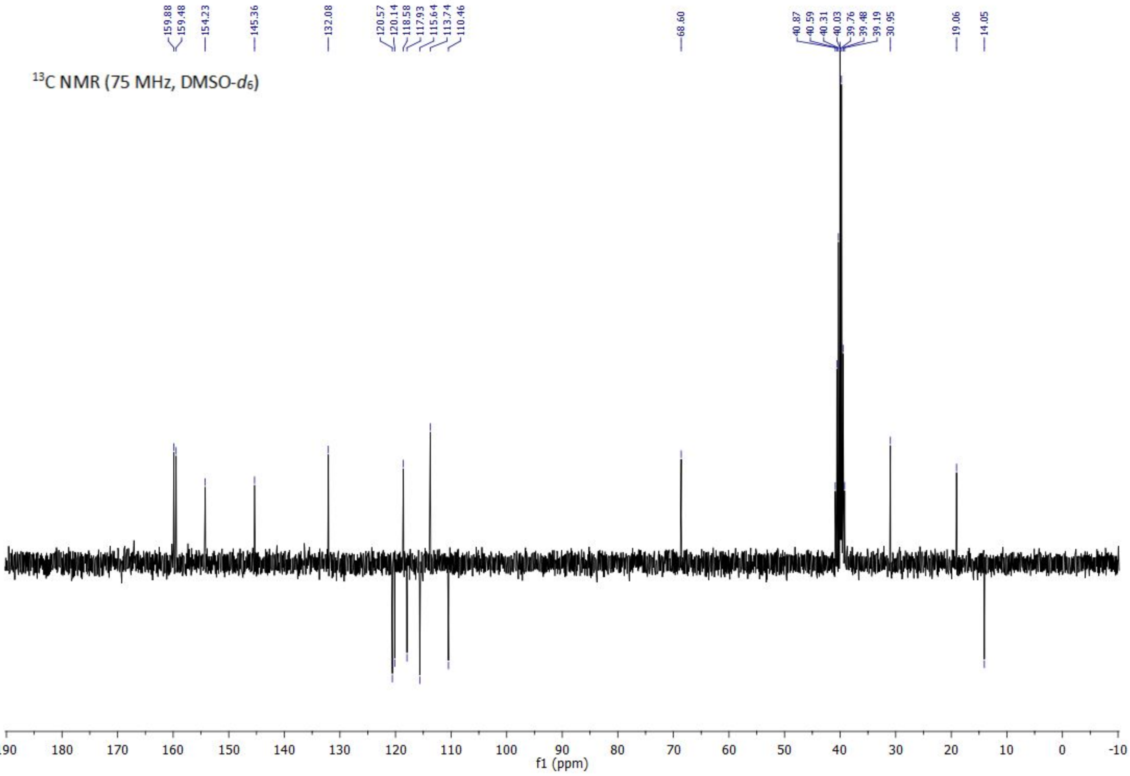

Figure S17. ^{13}C NMR spectrum of **1d**.

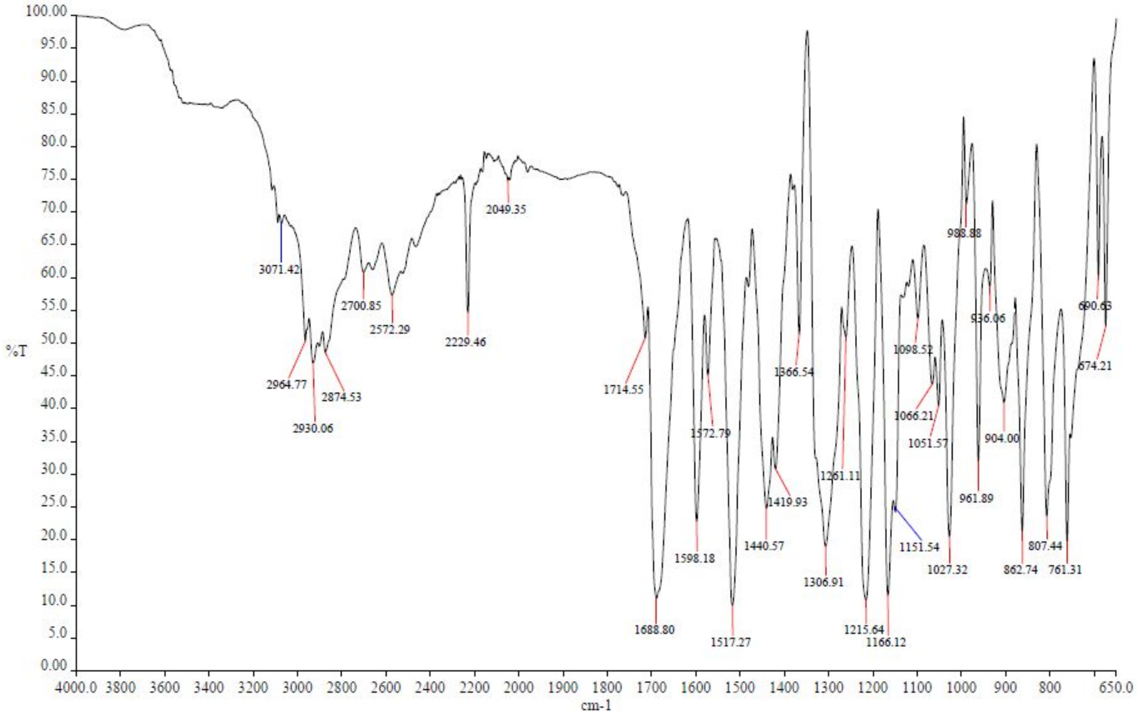

Figure S18. FT-IR spectrum of **1d**.

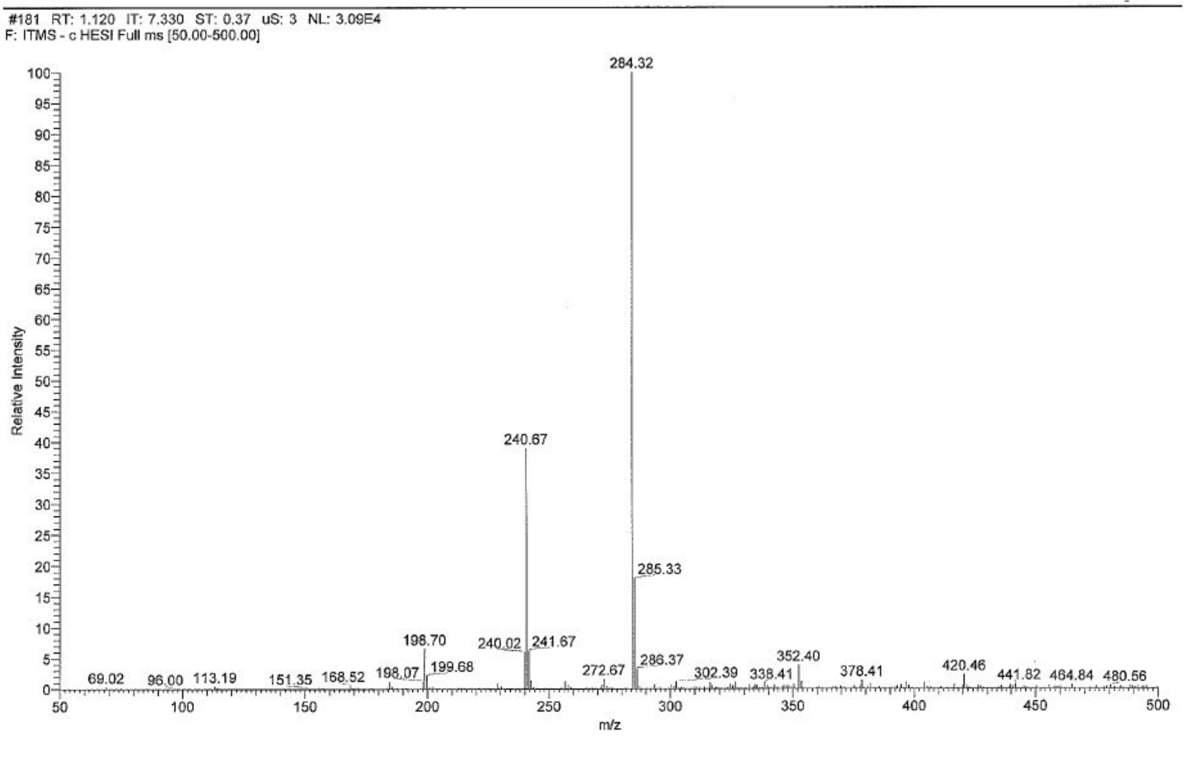

Figure S19. ESI-MS spectrum of **1d**.

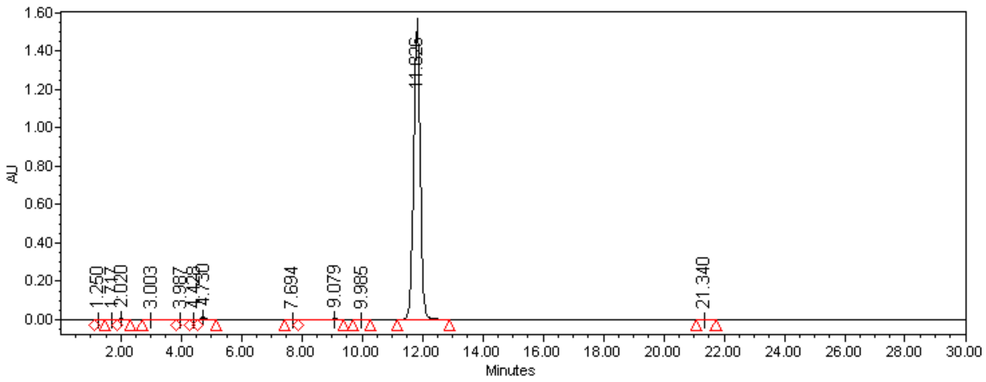

	Name	Retention Time (min)	Area (µV*sec)	% Area	Height (µV)	Int Type	Amount	Units	Peak Type	Peak Codes
1		1.250	28263	0.12	2794	VB			Unknown	
2		1.717	26894	0.11	1583	BV			Unknown	I08
3		2.020	52553	0.22	6155	VB			Unknown	
4		3.003	95783	0.40	1945	BV			Unknown	
5		3.987	27396	0.11	1435	VV			Unknown	
6		4.428	15064	0.06	1404	VV			Unknown	
7		4.730	163310	0.68	15218	VB			Unknown	
8		7.694	10206	0.04	670	BV			Unknown	
9		9.079	83431	0.35	3549	VB			Unknown	
10		9.985	21728	0.09	1676	BB			Unknown	
11		11.826	23424392	97.74	1531181	BB			Unknown	
12		21.340	17880	0.07	826	BB			Unknown	

Figure S20. HPLC chromatogram of **1d**. Operative conditions: sample amount: 10 µg; column: Phenomenex Luna® 3 µM C18(2) 100 Å, 4.6x100 mm; eluent system: water/MeOH + 0.05% TFA 30:70; flow rate: 1 mL/min; detector λ: 254 nm; time: 30 min; temperature: 23 °C.

- *5-(3-Cyano-5-isobutoxyphenyl)furan-2-carboxylic acid (**1e**)*

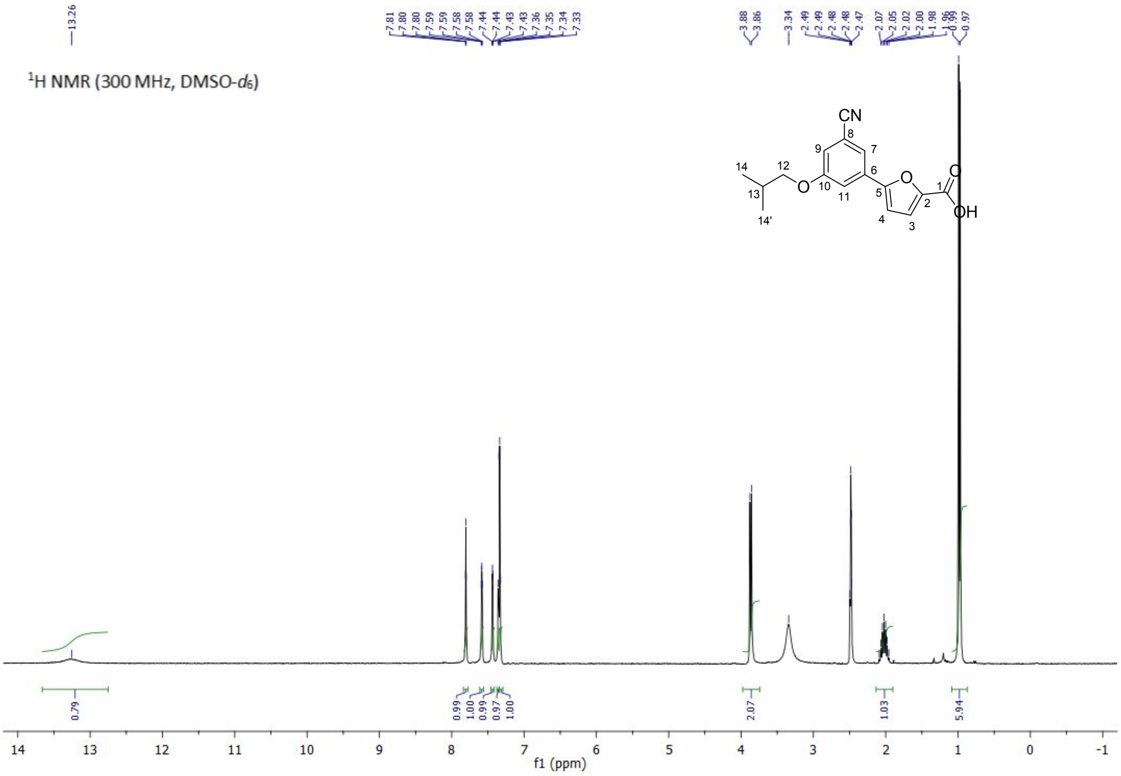

Figure S21. ^{1}H NMR spectrum of **1e**.

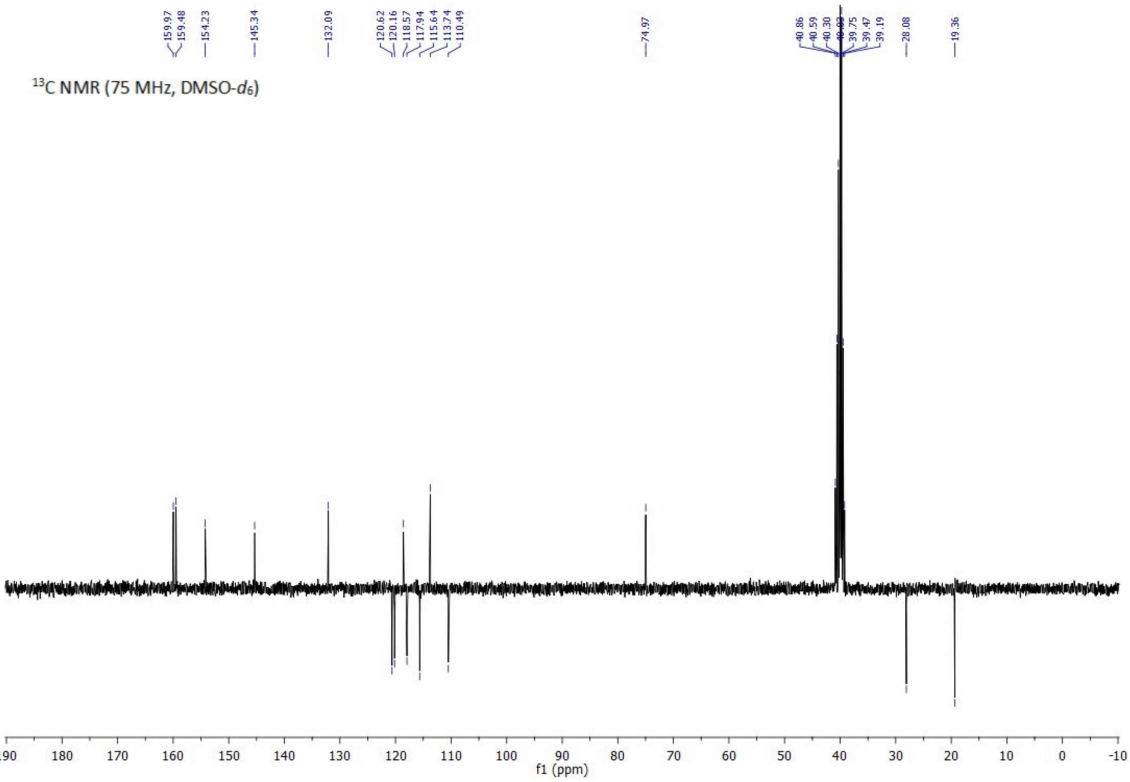

Figure S22. ^{13}C NMR spectrum of **1e**.

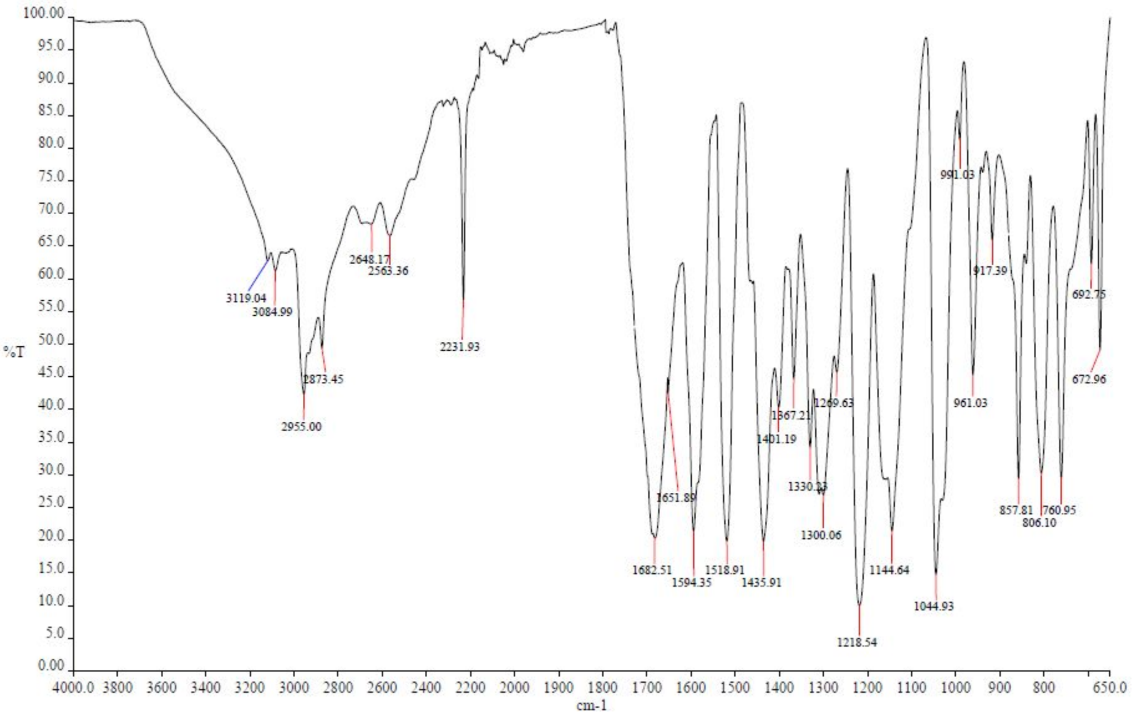

Figure S23. FT-IR spectrum of **1e**.

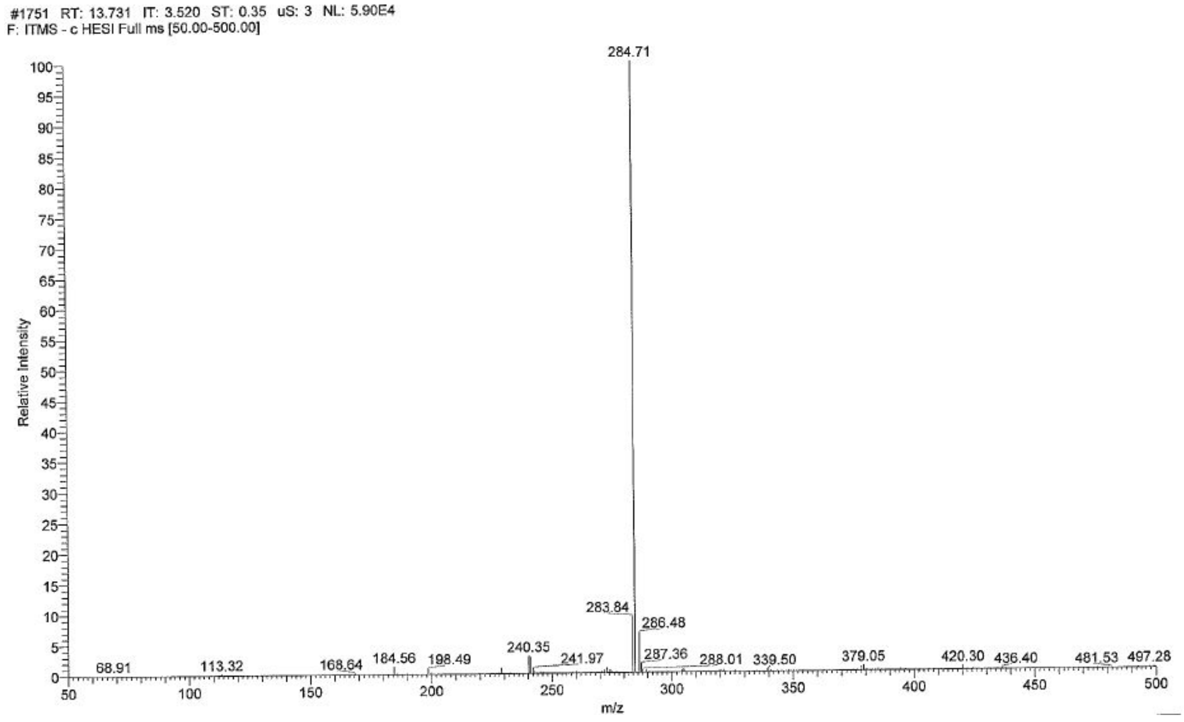

Figure S24. ESI-MS spectrum of **1e**.

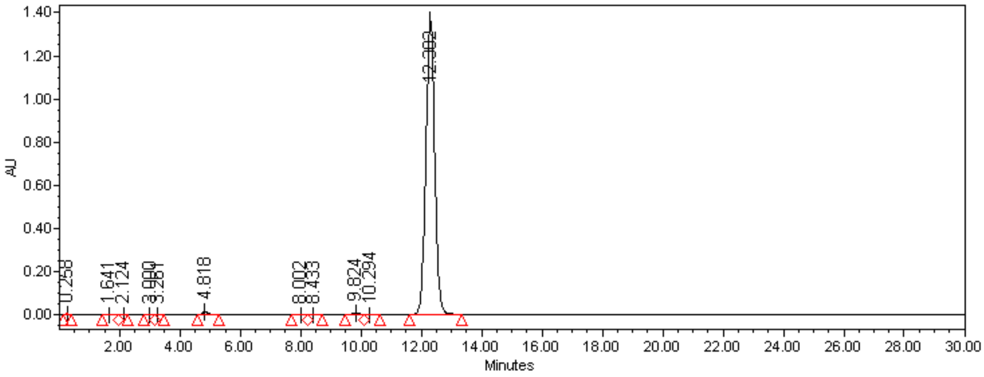

	Name	Retention Time (min)	Area (µV*sec)	% Area	Height (µV)	Int Type	Amount	Units	Peak Type	Peak Codes
1		0.258	19337	0.07	3012	BB			Unknown	
2		1.641	23630	0.08	1153	BV			Unknown	
3		2.124	6850	0.02	774	VB			Unknown	
4		3.000	24698	0.09	1630	BV			Unknown	I08
5		3.261	16598	0.06	1593	VB			Unknown	
6		4.818	188550	0.66	15801	BB			Unknown	
7		8.002	27572	0.10	1731	BV			Unknown	
8		8.433	25928	0.09	1675	VB			Unknown	
9		9.824	116134	0.41	8056	BV			Unknown	
10		10.294	25592	0.09	1689	VB			Unknown	
11		12.302	27898915	98.33	1365216	BB			Unknown	

Figure S25. HPLC chromatogram of **1e**. Operative conditions: sample amount: 10 µg; column: Phenomenex Luna® 3 µM C18(2) 100 Å, 4.6x100 mm; eluent system: water/MeOH + 0.05% TFA 30:70; flow rate: 1 mL/min; detector λ: 254 nm; time: 30 min; temperature: 23 °C.

- *5-(3-Cyano-5-(cyclopropylmethoxy)phenyl)furan-2-carboxylic acid (**1f**)*

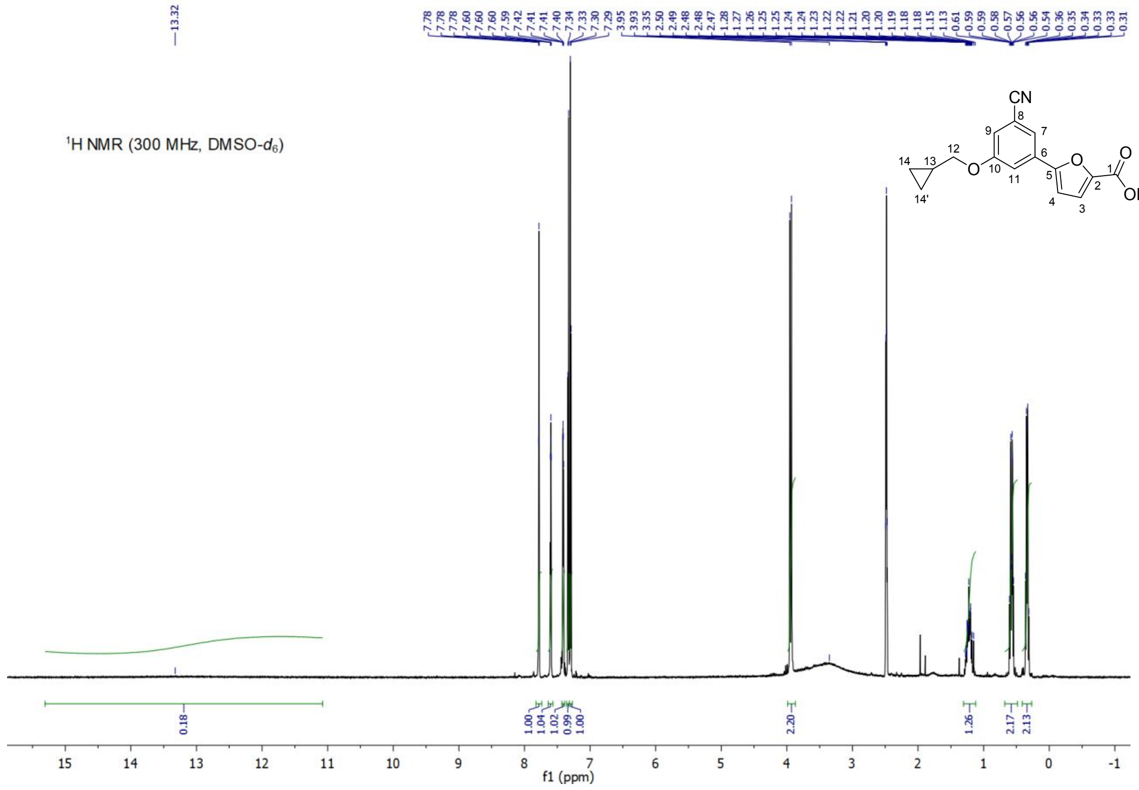

Figure S26. ^{1}H NMR spectrum of **1f**.

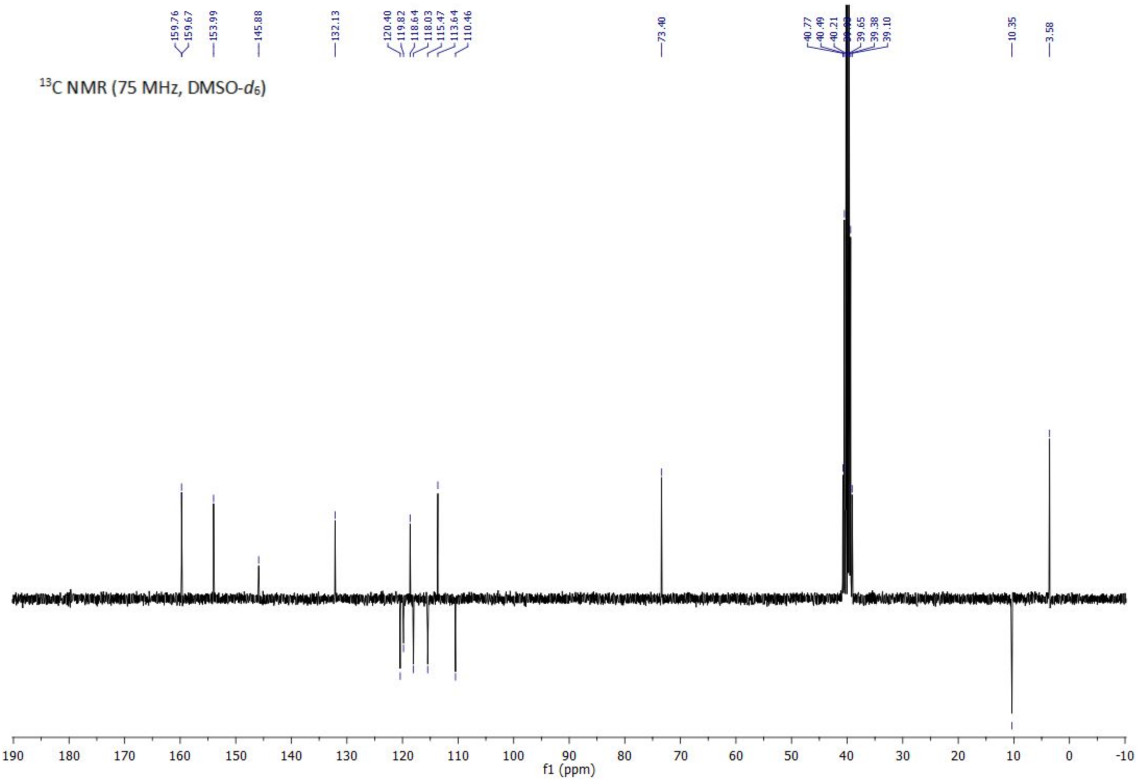

Figure S27. ^{13}C NMR spectrum of **1f**.

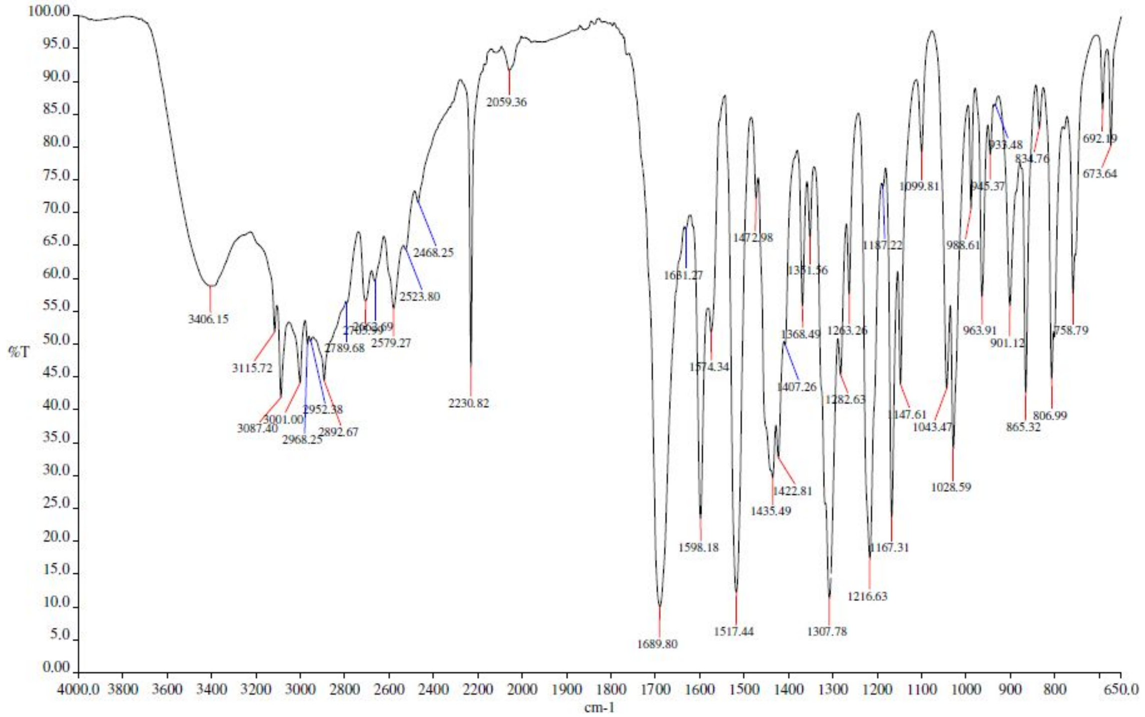

Figure S28. FT-IR spectrum of **1f**.

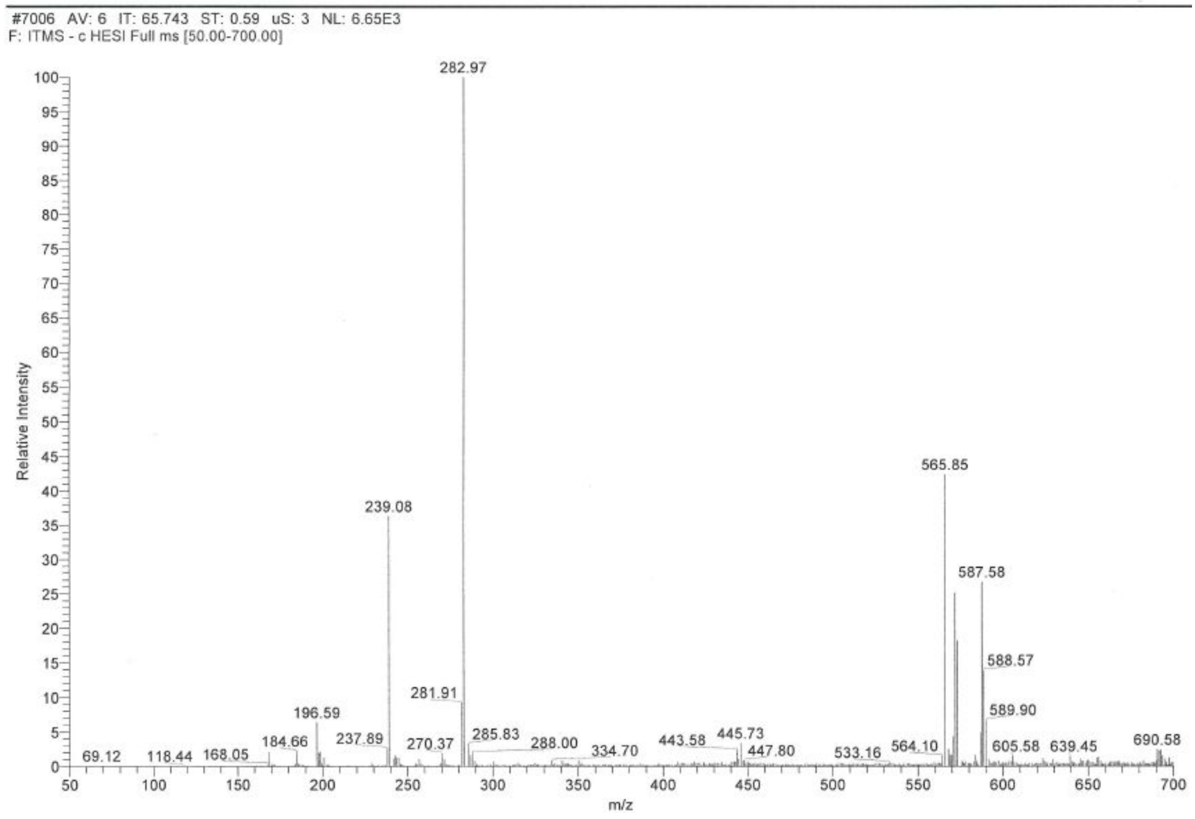

Figure S29. ESI-MS spectrum of **1f**.

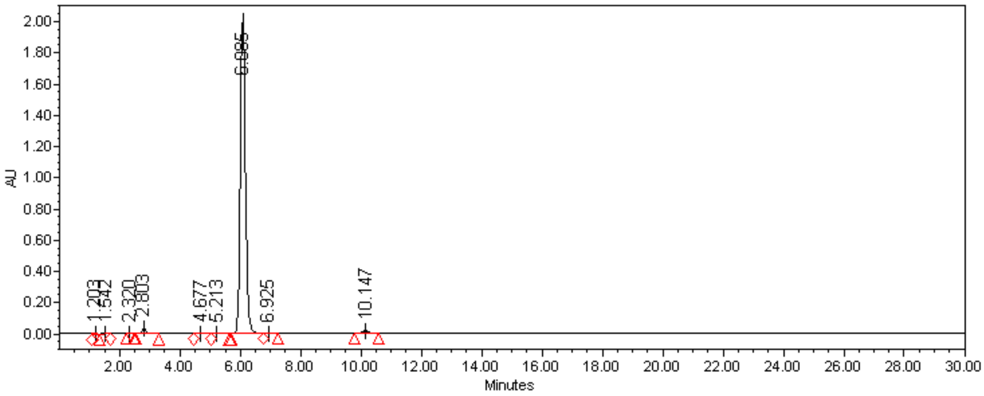

	Name	Retention Time (min)	Area (µV*sec)	% Area	Height (µV)	Int Type	Amount	Units	Peak Type	Peak Codes
1		1.203	20732	0.08	2392	VB			Unknown	
2		1.542	52386	0.21	4224	BV			Unknown	
3		2.320	30692	0.12	5169	BB			Unknown	
4		2.803	309154	1.24	34903	BB			Unknown	
5		4.677	48553	0.20	3028	VV			Unknown	
6		5.213	26345	0.11	2053	VB			Unknown	
7		6.085	24097017	96.79	2008242	BV			Unknown	
8		6.925	59596	0.24	3972	VB			Unknown	
9		10.147	252525	1.01	17179	BB			Unknown	

Figure S30. HPLC chromatogram of **1f**. Operative conditions: sample amount: 10 µg; column: Phenomenex Luna® 3 µM C18(2) 100 Å, 4.6x100 mm; eluent system: water/MeOH + 0.05% TFA 30:70; flow rate: 1 mL/min; detector λ: 254 nm; time: 30 min; temperature: 23 °C.

- *5-(3-Cyano-5-(cyclobutylmethoxy)phenyl)furan-2-carboxylic acid (**1g**)*

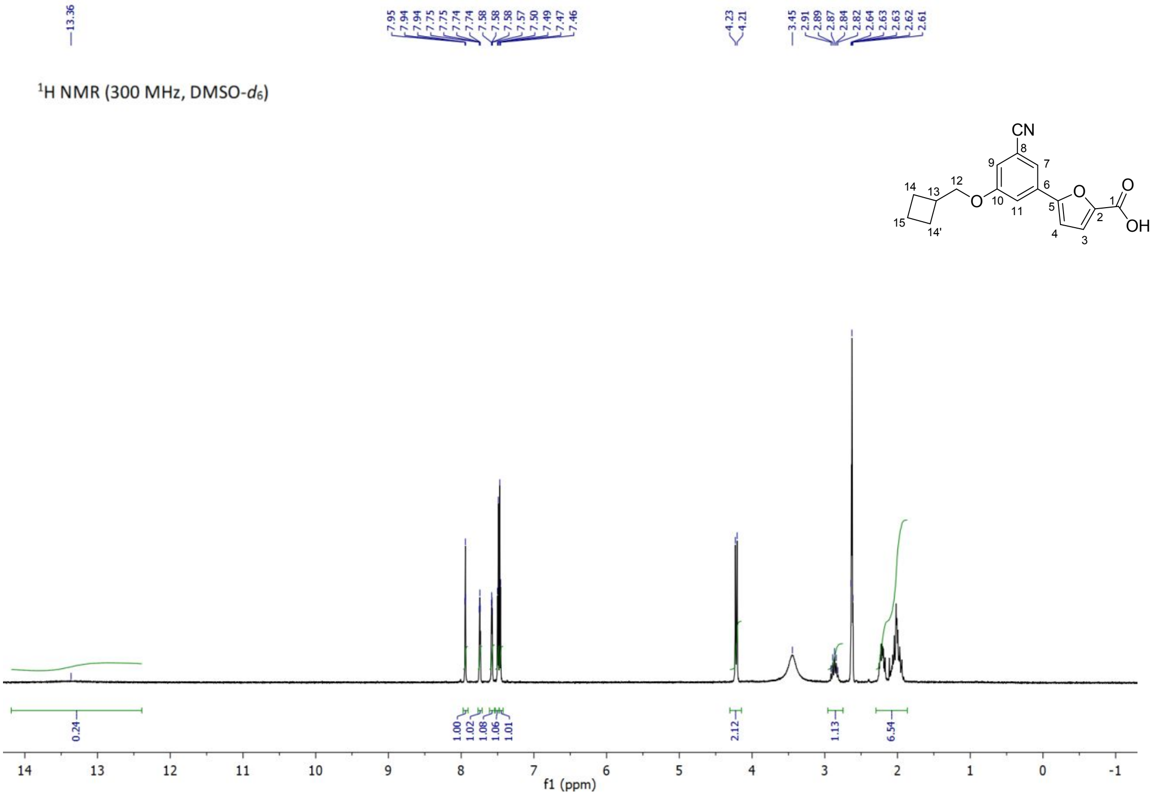

Figure S31. ^{1}H NMR spectrum of **1g**.

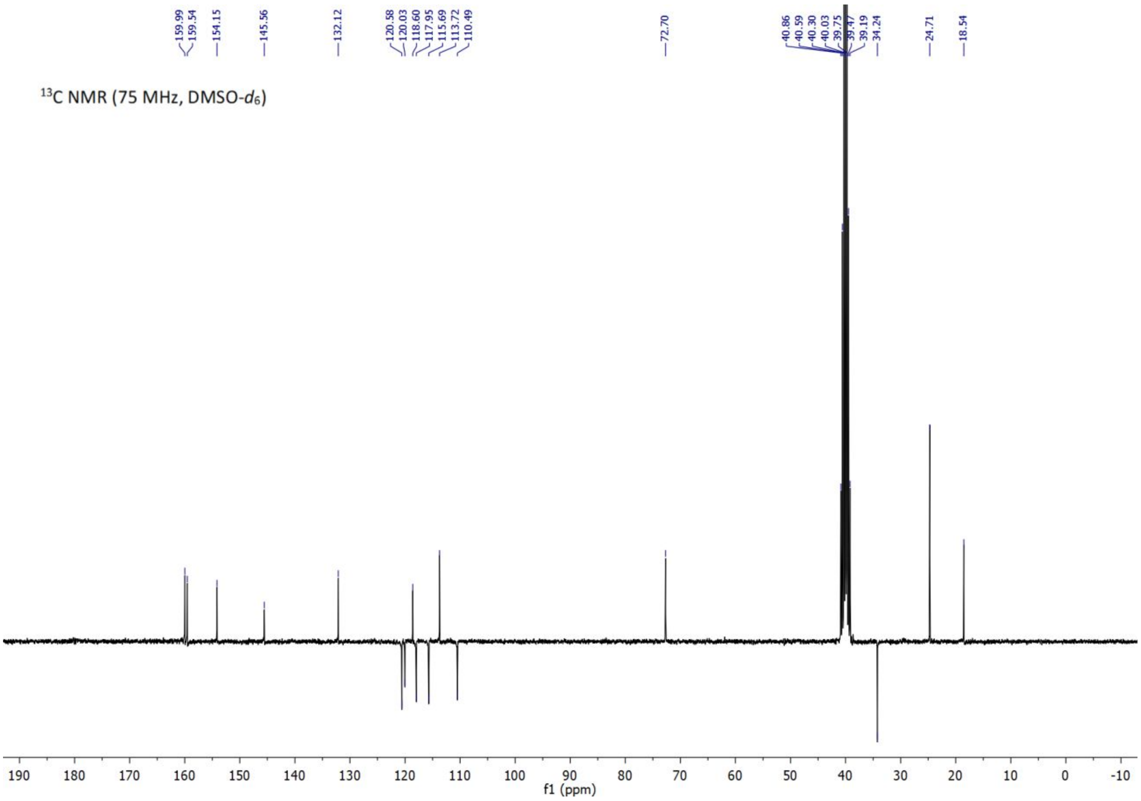

Figure S32. ^{13}C NMR spectrum of **1g**.

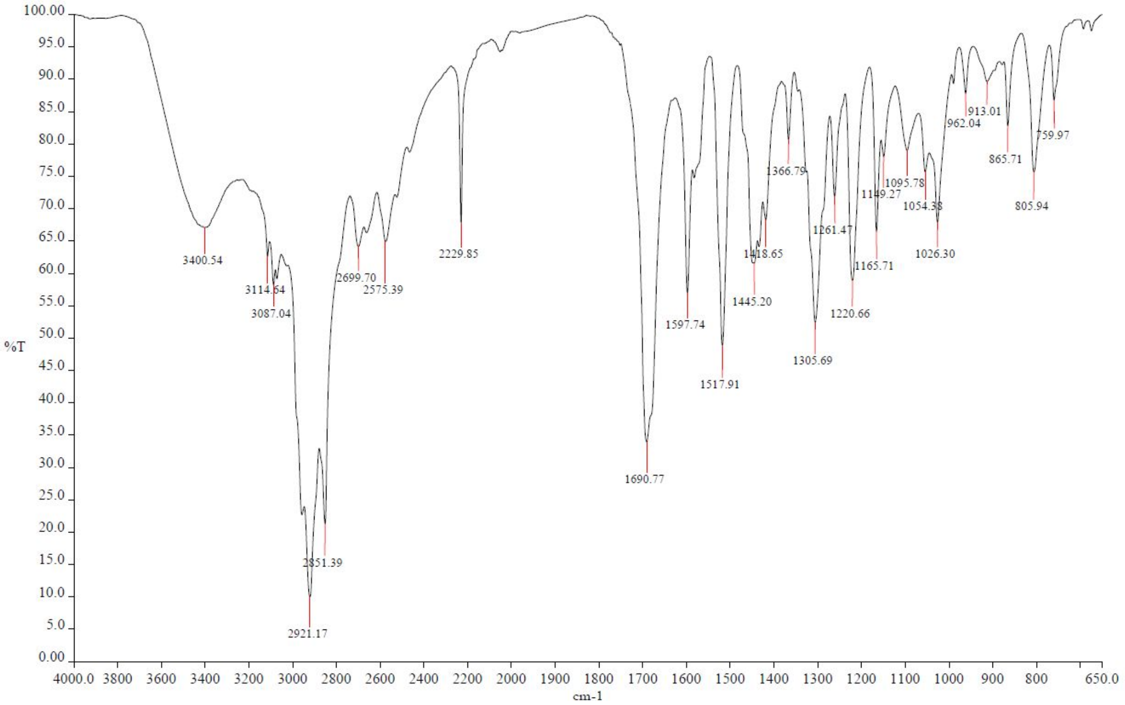

Figure S33. FT-IR spectrum of **1g**.

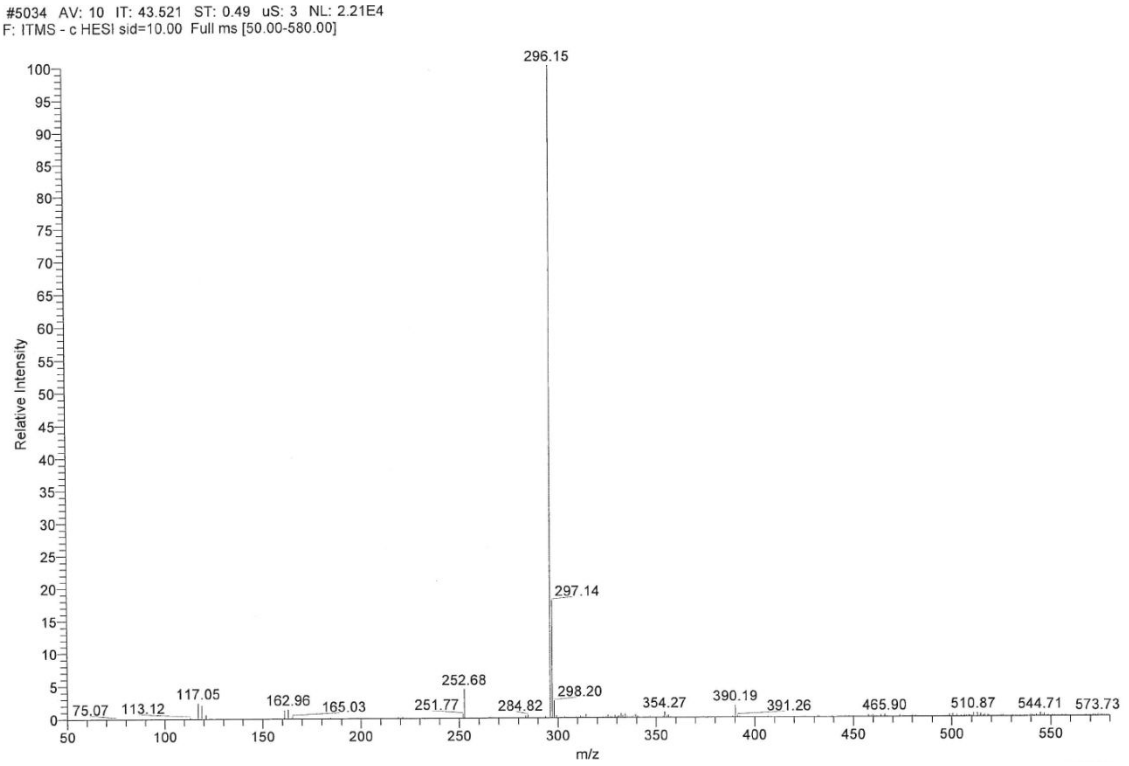

Figure S34. ESI-MS spectrum of **1g**.

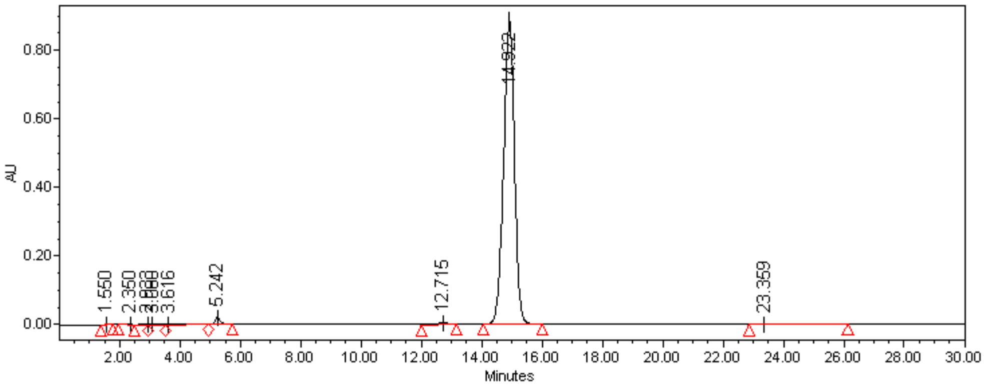

	Name	Retention Time (min)	Area (µV*sec)	% Area	Height (µV)	Int Type	Amount	Units	Peak Type	Peak Codes
1		1.550	18678	0.08	1276	BB			Unknown	I08
2		2.350	39187	0.17	2115	BB			Unknown	I08
3		2.933	45801	0.20	2494	BV			Unknown	I06
4		3.080	93762	0.41	3213	VV			Unknown	
5		3.616	109203	0.48	2259	VV			Unknown	
6		5.242	249347	1.09	20049	VB			Unknown	
7		12.715	135934	0.59	6051	BB			Unknown	
8		14.922	22115410	96.53	885876	BB			Unknown	
9		23.359	103627	0.45	1094	BB			Unknown	

Figure S35. HPLC chromatogram of **1g**. Operative conditions: sample amount: 10 µg; column: Phenomenex Luna® 3 µM C18(2) 100 Å, 4.6x100 mm; eluent system: water/MeOH + 0.05% TFA 30:70; flow rate: 1 mL/min; detector λ: 254 nm; time: 30 min; temperature: 23 °C.

- *5-(3-Cyano-5-(neopentyloxy)phenyl)furan-2-carboxylic acid (**1h**)*

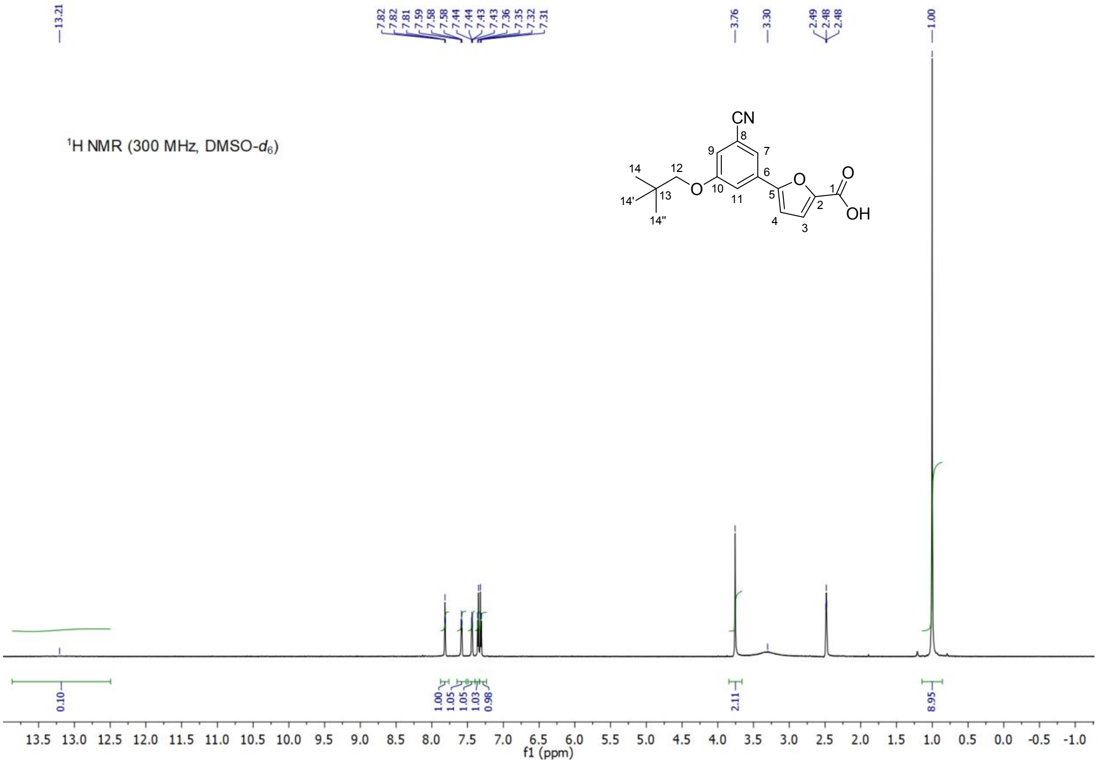

Figure S36. ^{1}H NMR spectrum of **1h**.

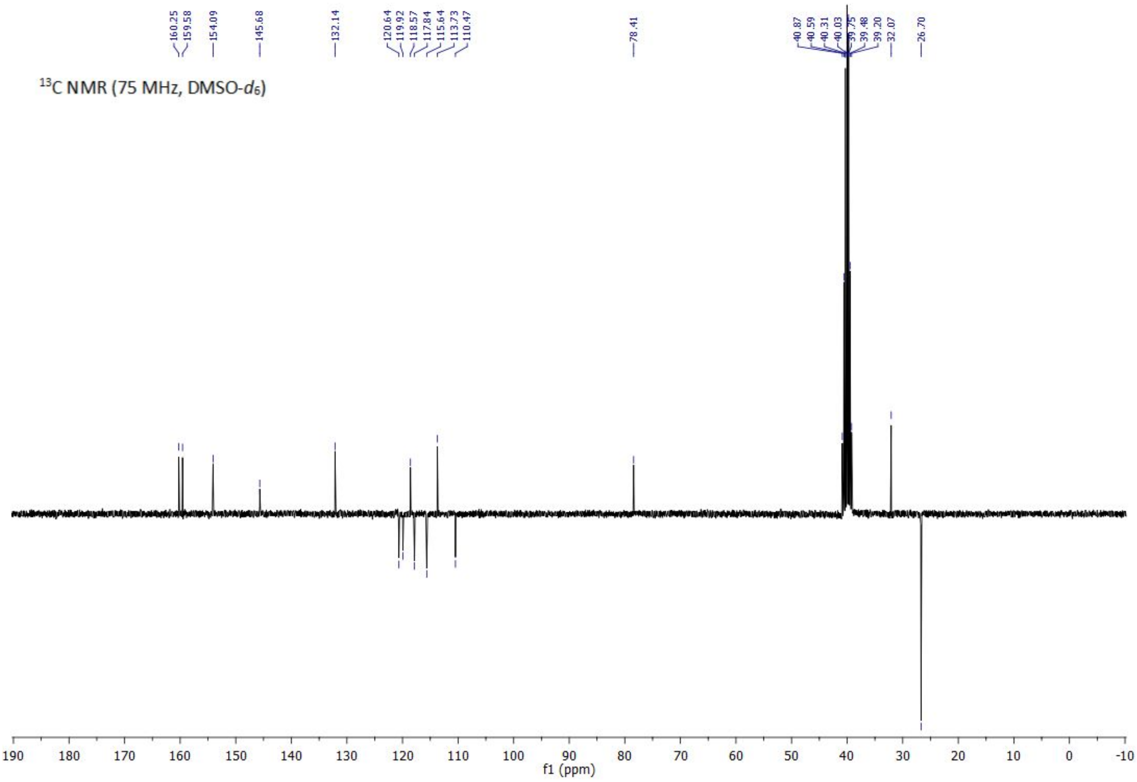

Figure S37. ^{13}C NMR spectrum of **1h**.

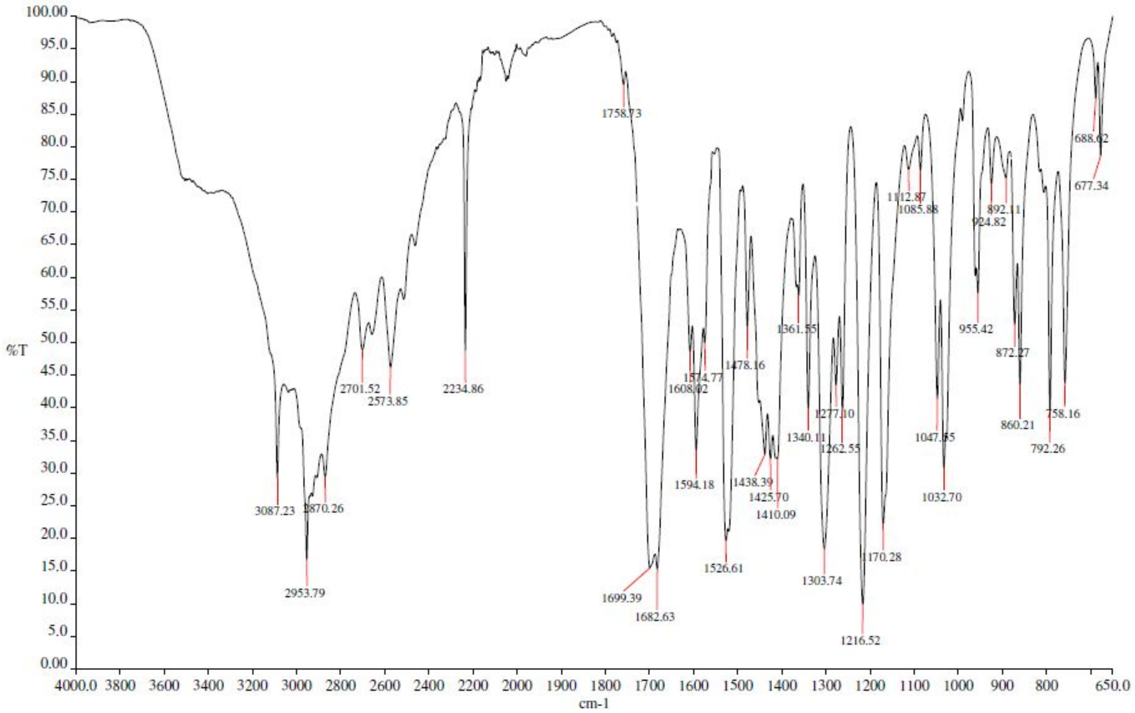

Figure S38. FT-IR spectrum of **1h**.

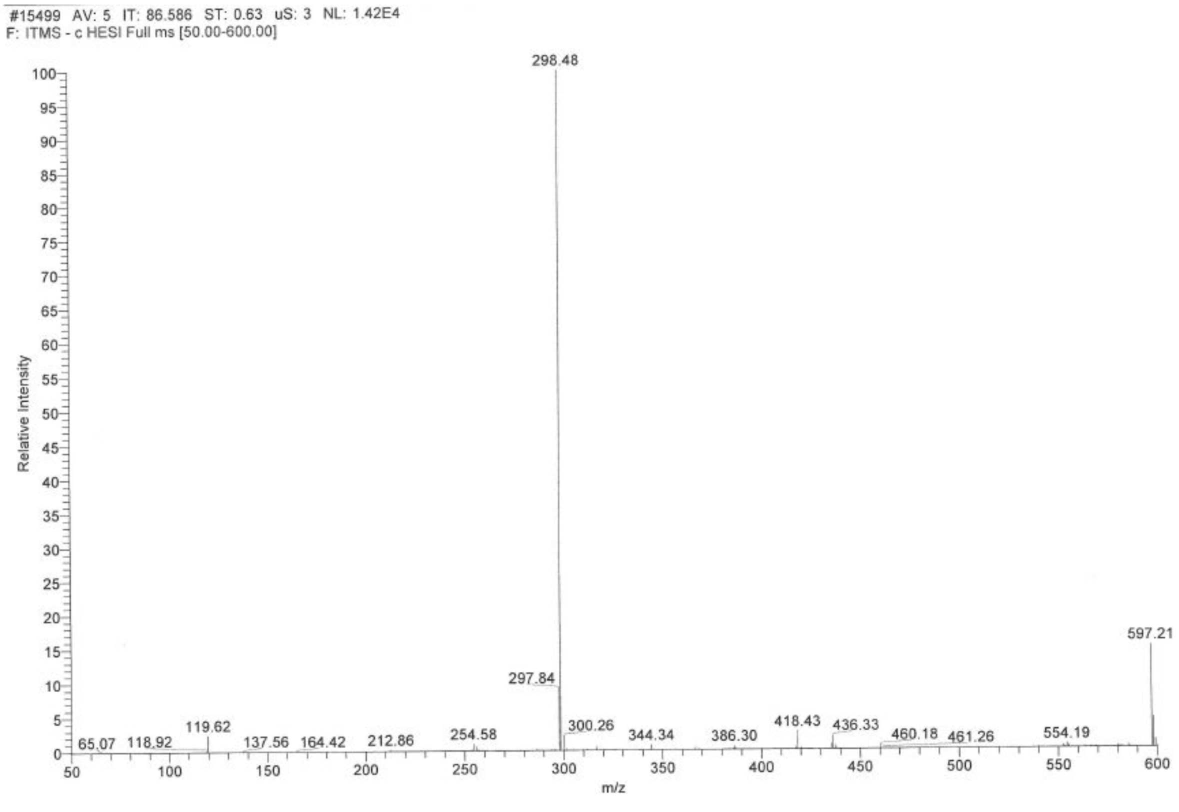

Figure S39. ESI-MS spectrum of **1h**.

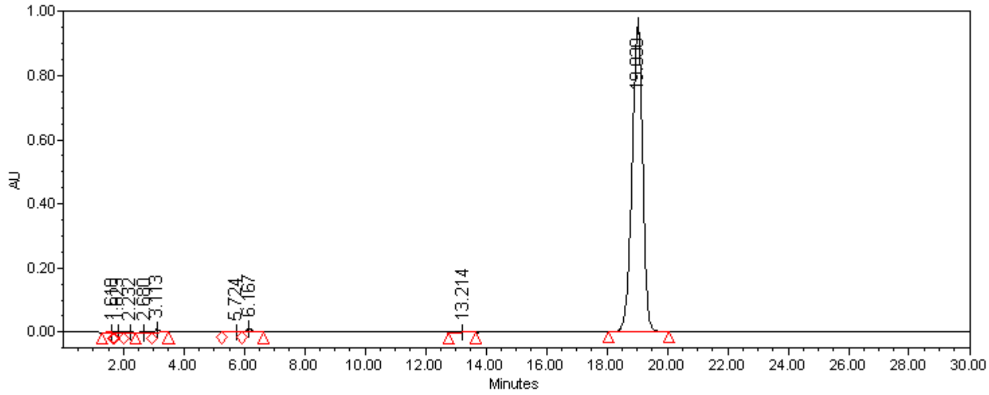

	Name	Retention Time (min)	Area (µV*sec)	% Area	Height (µV)	Int Type	Amount	Units	Peak Type	Peak Codes
1		1.610	18493	0.08	1360	BV			Unknown	
2		1.823	34378	0.14	2187	VV			Unknown	
3		2.232	57313	0.24	4823	VB			Unknown	
4		2.680	33297	0.14	1427	BV			Unknown	
5		3.113	106407	0.44	10730	VB			Unknown	
6		5.724	31320	0.13	2023	VV			Unknown	
7		6.167	161772	0.67	12464	VB			Unknown	
8		13.214	70729	0.29	3120	BB			Unknown	
9		19.030	23487050	97.86	952895	BB			Unknown	

Figure S40. HPLC chromatogram of **1h**. Operative conditions: sample amount: 10 µg; column: Phenomenex Luna® 3 µM C18(2) 100 Å, 4.6x100 mm; eluent system: water/MeOH + 0.05% TFA 30:70; flow rate: 1 mL/min; detector λ: 254 nm; time: 30 min; temperature: 23 °C.

- *5-(3-Cyano-5-(pentyloxy)phenyl)furan-2-carboxylic acid (**1i**)*

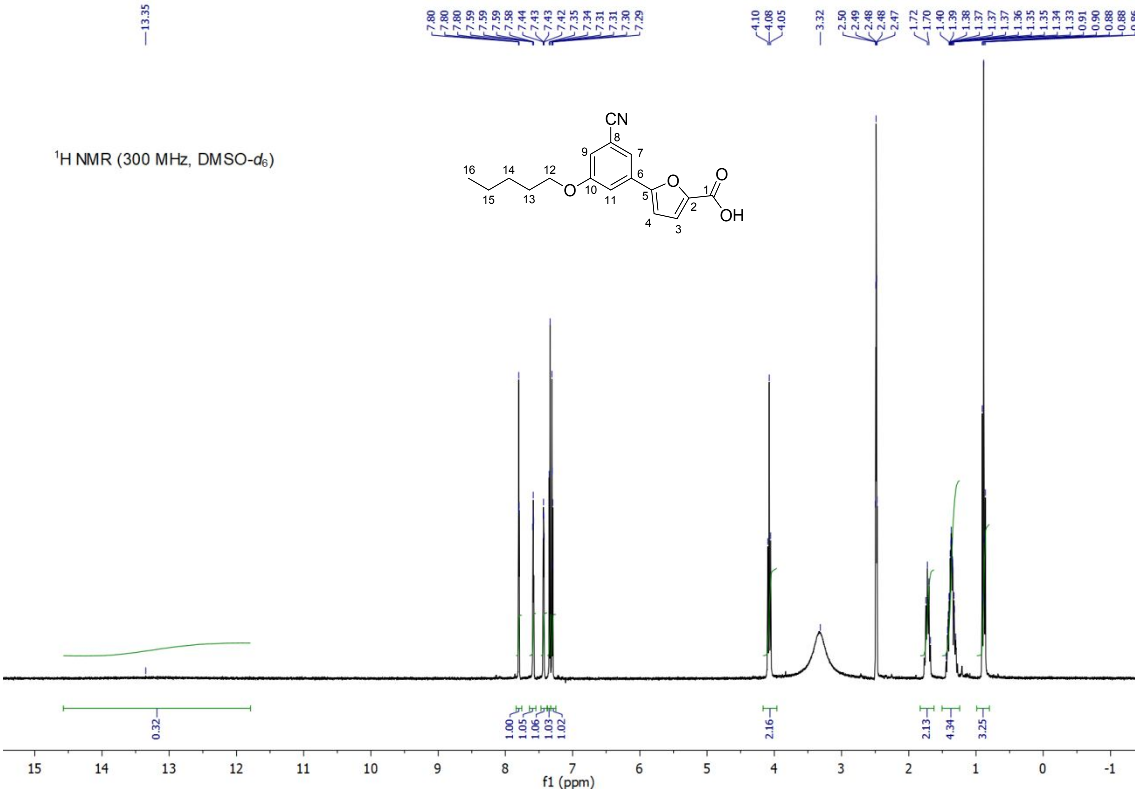

Figure S41. ^{1}H NMR spectrum of **1i**.

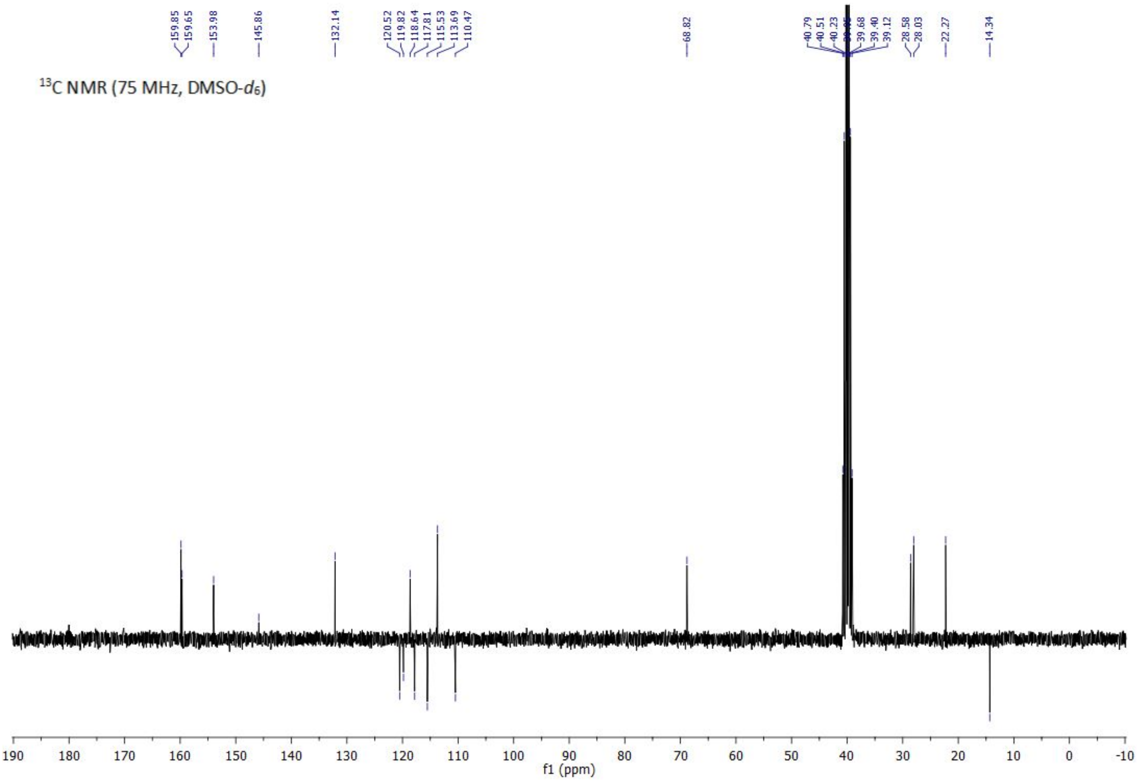

Figure S42. ^{13}C NMR spectrum of **1i**.

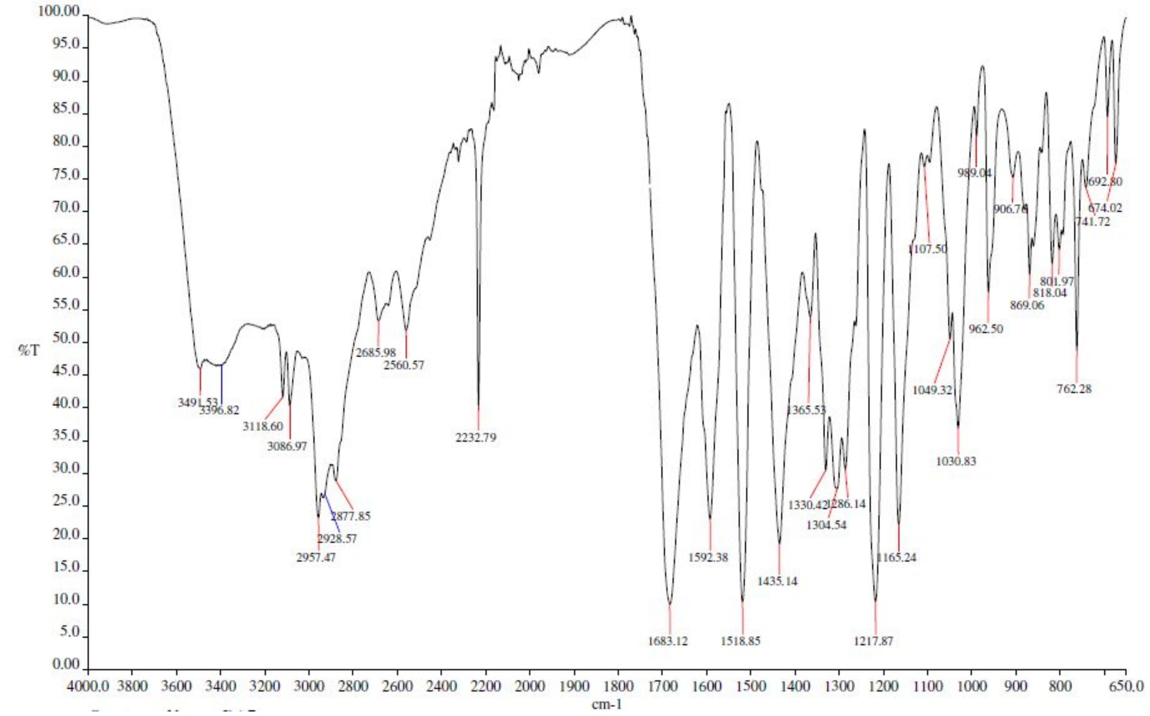

Figure S43. FT-IR spectrum of **1i**.

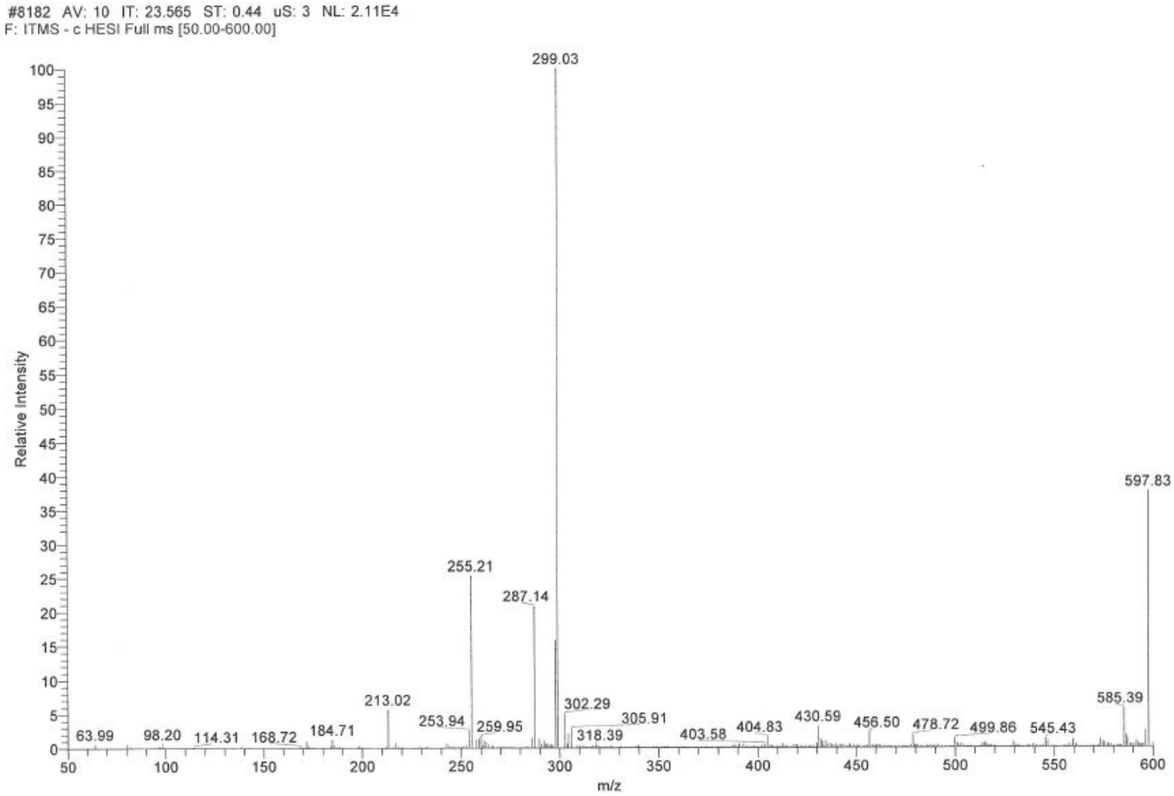

Figure S44. ESI-MS spectrum of **1i**.

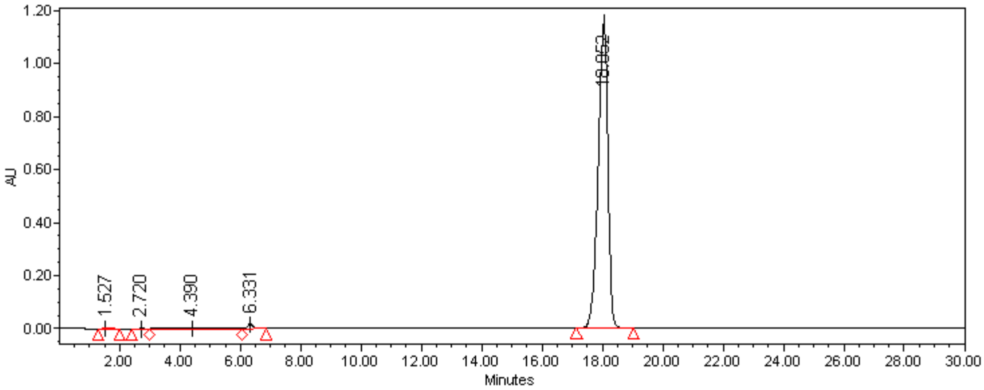

	Name	Retention Time (min)	Area (µV*sec)	% Area	Height (µV)	Int Type	Amount	Units	Peak Type	Peak Codes
1		1.527	60166	0.22	3926	BB			Unknown	
2		2.720	39230	0.15	1640	BV			Unknown	
3		4.390	267065	1.00	2006	VV			Unknown	
4		6.331	237227	0.89	17546	VB			Unknown	
5		18.052	26197360	97.75	1150063	BB			Unknown	

Figure S45. HPLC chromatogram of **1i**. Operative conditions: sample amount: 10 µg; column: Phenomenex Luna® 3 µM C18(2) 100 Å, 4.6x100 mm; eluent system: water/MeOH + 0.05% TFA 30:70; flow rate: 1 mL/min; detector λ: 254 nm; time: 30 min; temperature: 23 °C.

- *5-(3-Cyano-5-(isopentyloxy)phenyl)furan-2-carboxylic acid (**1j**)*

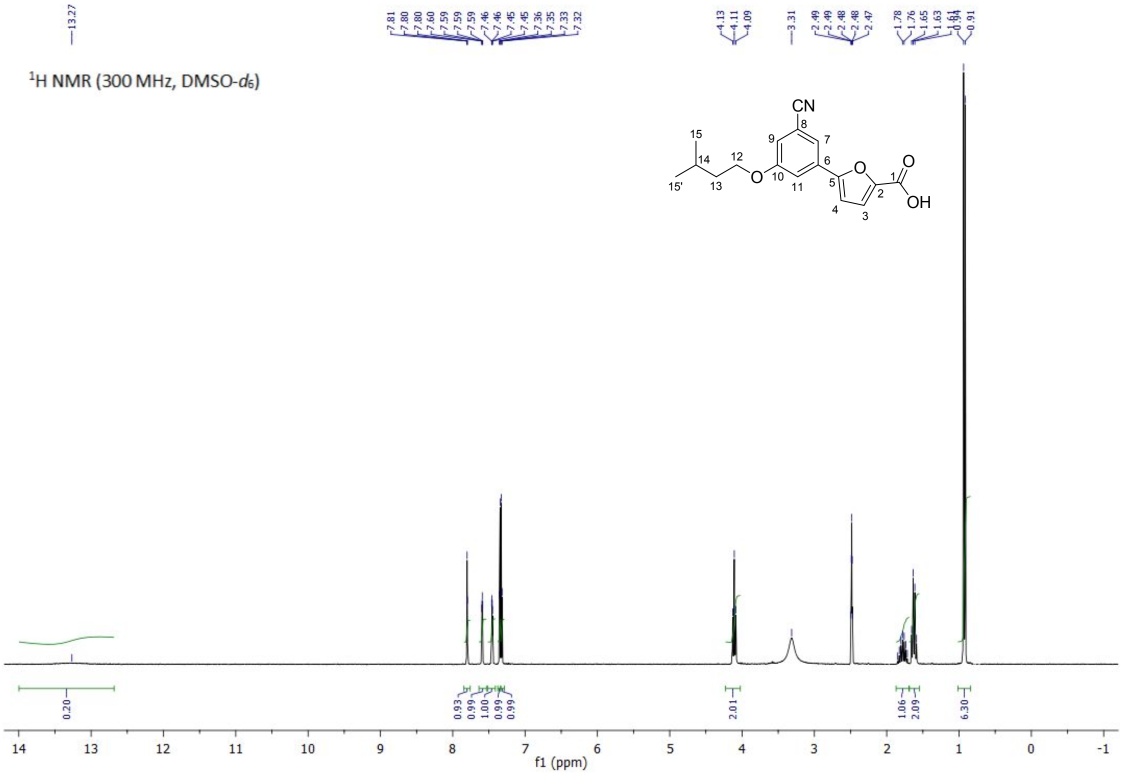

Figure S46. ^{1}H NMR spectrum of **1j**.

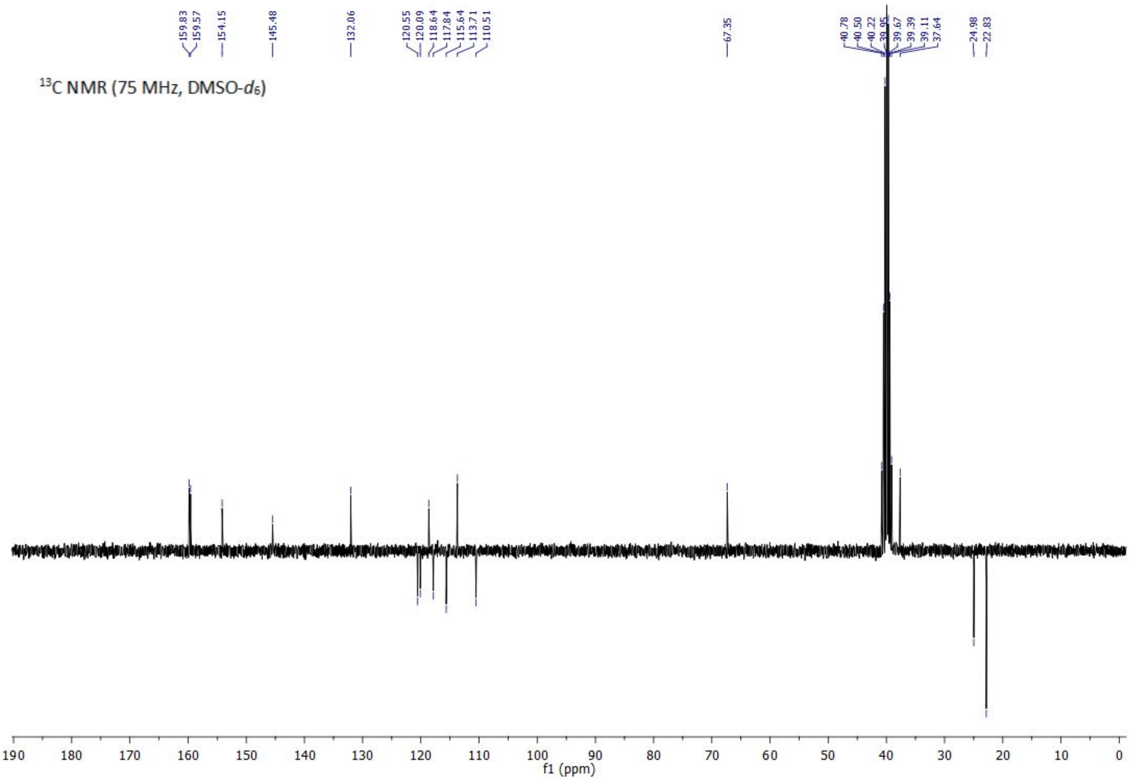

Figure S47. ^{13}C NMR spectrum of **1j**.

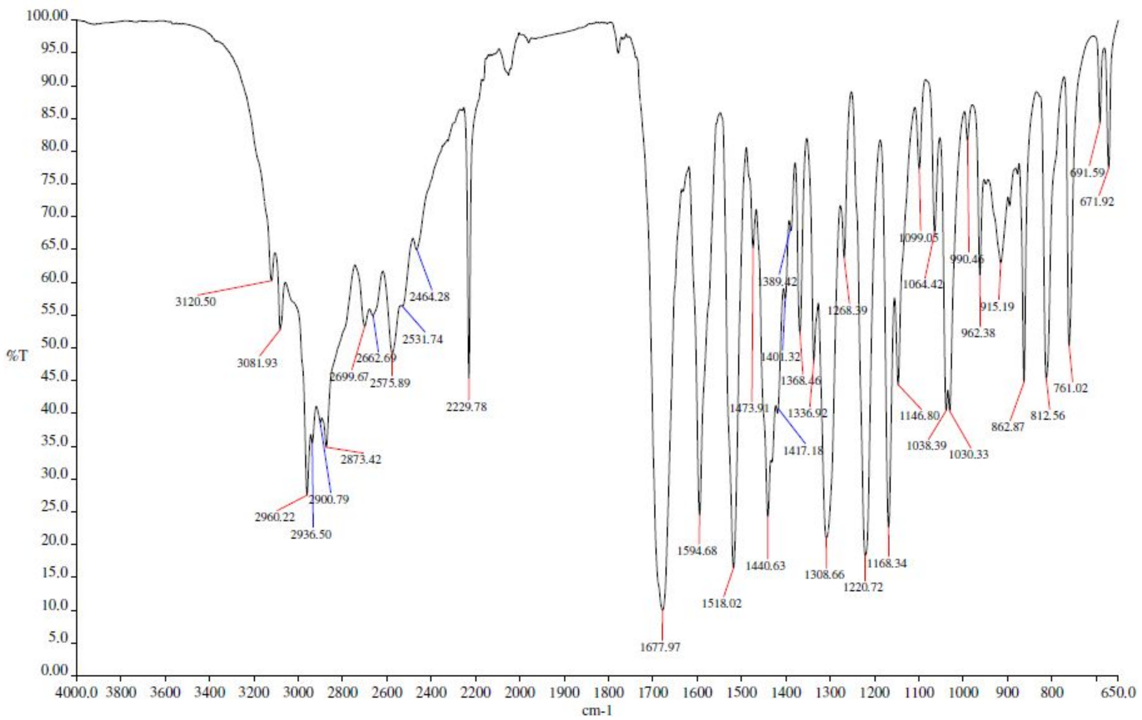

Figure S48. FT-IR spectrum of **1j**.

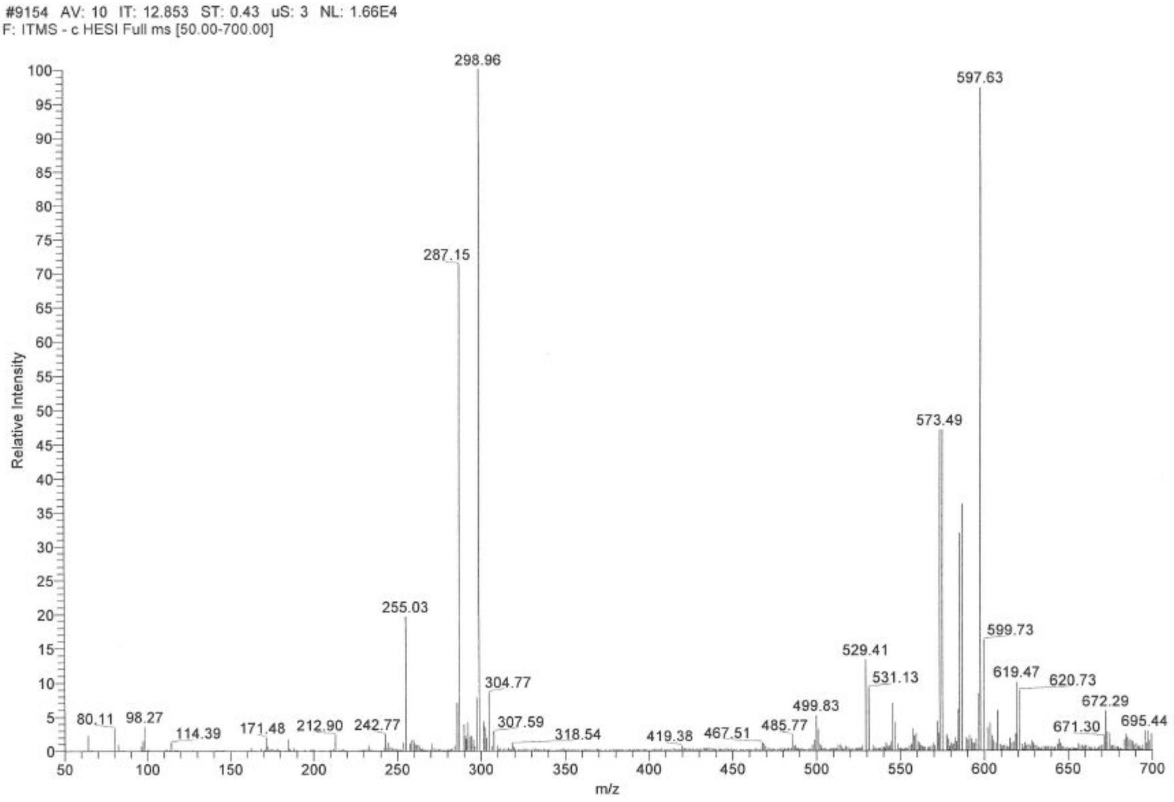

Figure S49. ESI-MS spectrum of **1j**.

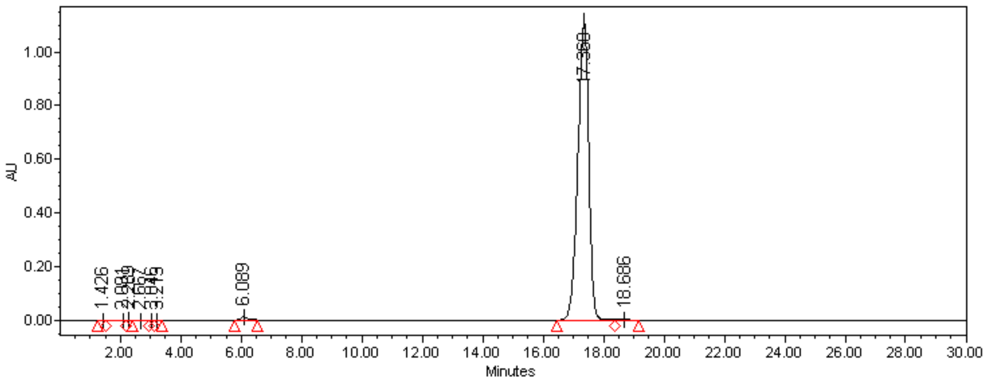

	Name	Retention Time (min)	Area (µV*sec)	% Area	Height (µV)	Int Type	Amount	Units	Peak Type	Peak Codes
1		1.426	23361	0.08	1898	BV			Unknown	
2		2.091	94310	0.33	2656	VV			Unknown	
3		2.289	27351	0.10	3289	VB			Unknown	
4		2.667	37598	0.13	1621	BV			Unknown	I08
5		3.046	11951	0.04	1256	VV			Unknown	
6		3.213	8503	0.03	941	VB			Unknown	
7		6.089	158685	0.56	11905	BB			Unknown	
8		17.360	27922894	98.51	1113731	BV			Unknown	
9		18.686	59672	0.21	2190	VB			Unknown	

Figure S50. HPLC chromatogram of **1j**. Operative conditions: sample amount: 10 µg; column: Phenomenex Luna® 3 µM C18(2) 100 Å, 4.6x100 mm; eluent system: water/MeOH + 0.05% TFA 30:70; flow rate: 1 mL/min; detector λ: 254 nm; time: 30 min; temperature: 23 °C.

• *5-(3-Cyano-5-(hexyloxy)phenyl)furan-2-carboxylic acid (**1k**)*

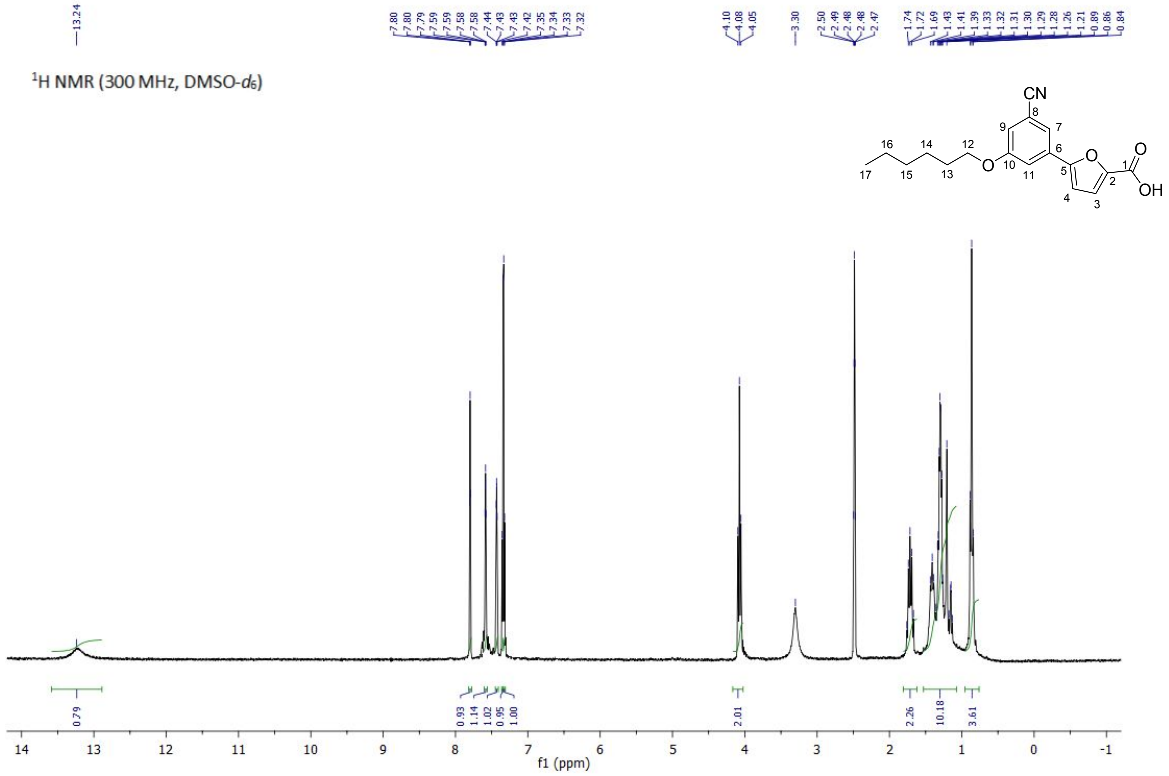

Figure S51. ^{1}H NMR spectrum of **1k**.

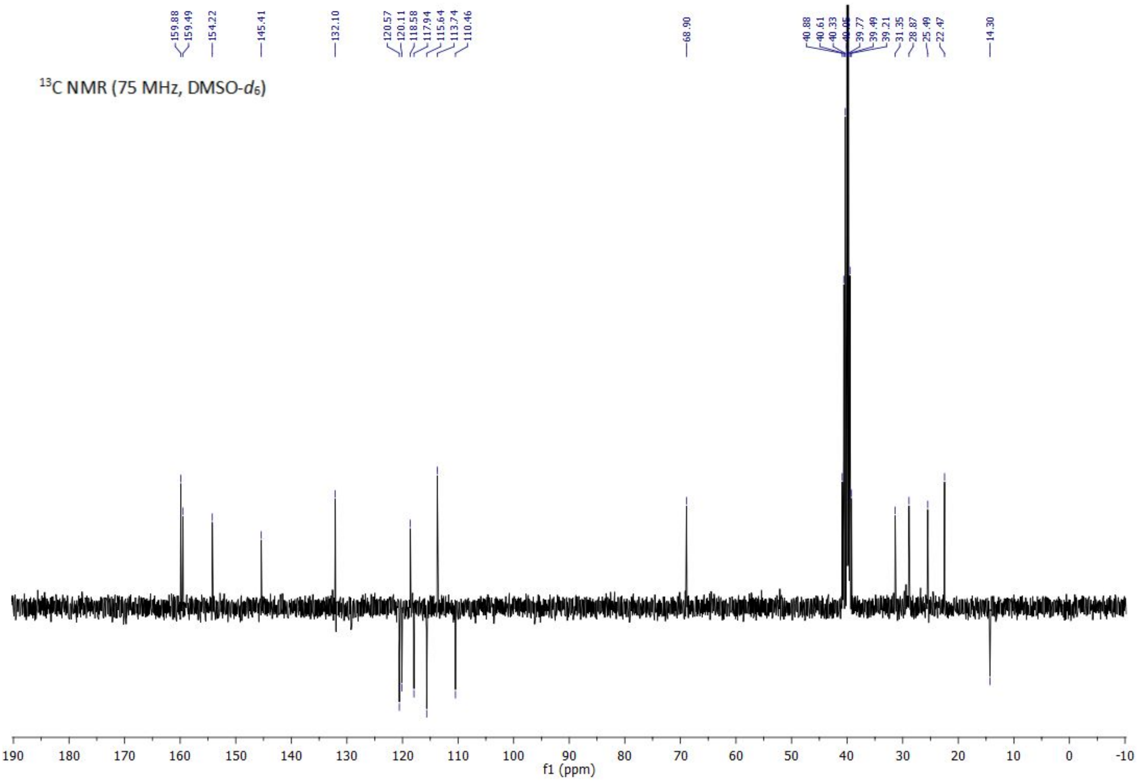

Figure S52. ^{13}C NMR spectrum of **1k**.

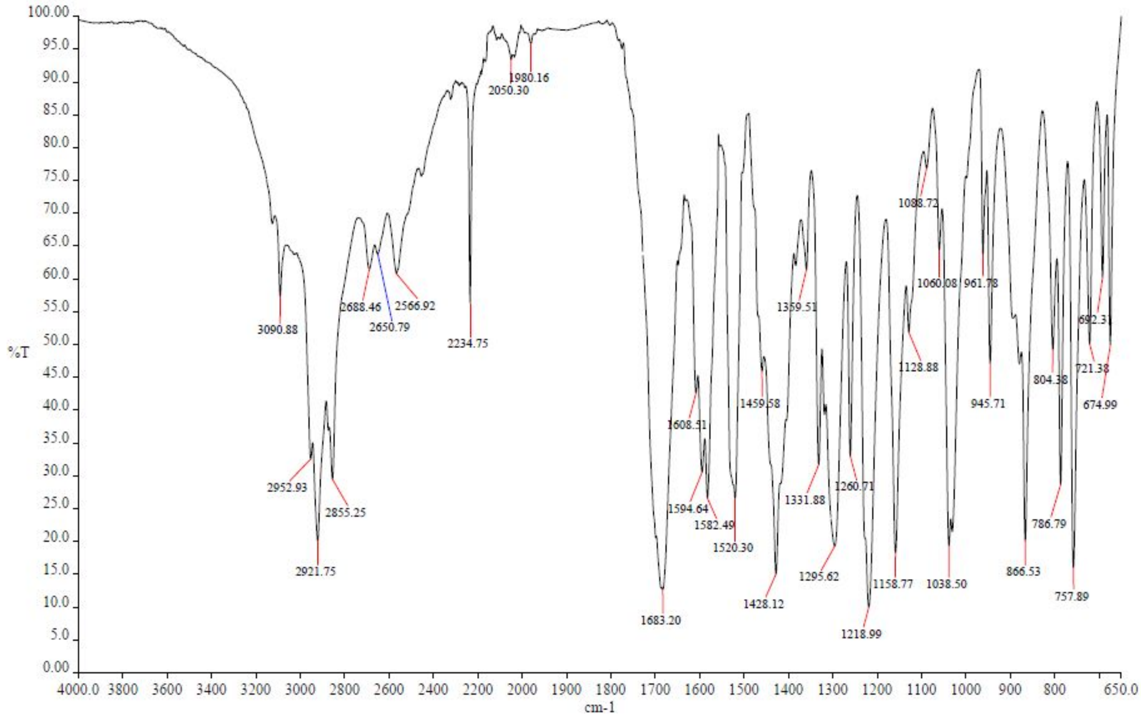

Figure S53. FT-IR spectrum of **1k**.

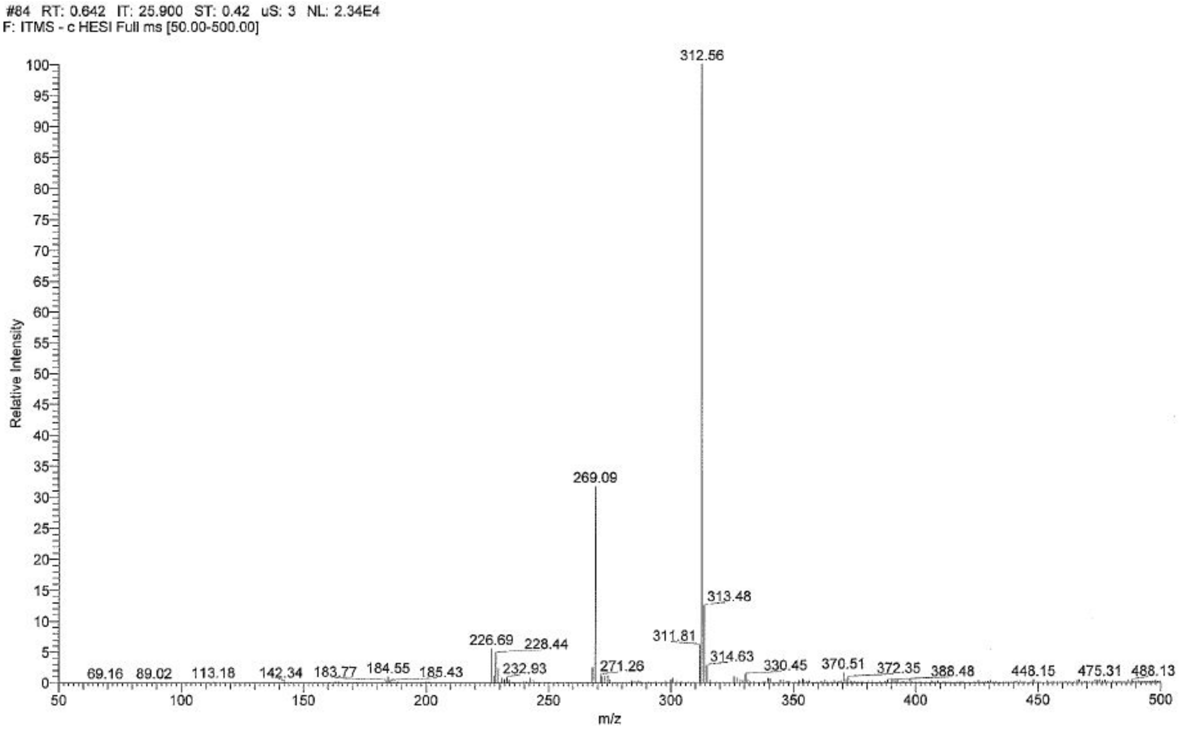

Figure S54. ESI-MS spectrum of **1k**.

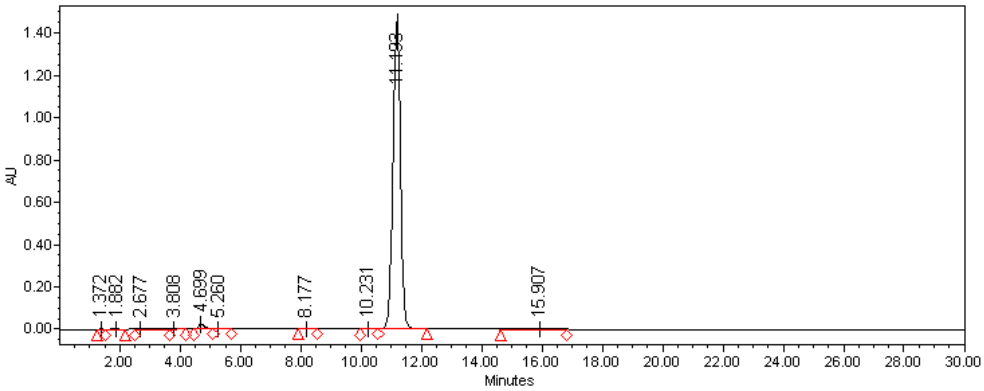

	Name	Retention Time (min)	Area (µV*sec)	% Area	Height (µV)	Int Type	Amount	Units	Peak Type	Peak Codes
1		1.882	47039	0.18	2777	VB			Unknown	
2		2.677	76181	0.29	1323	VV			Unknown	
3		3.808	46139	0.18	2928	VV			Unknown	
4		4.699	347711	1.34	25091	VV			Unknown	
5		5.260	46250	0.18	2125	VV			Unknown	
6		8.177	25292	0.10	1511	BV			Unknown	
7		10.231	54856	0.21	2516	VV			Unknown	
8		11.193	25305786	97.17	1457634	VB			Unknown	
9		15.907	70175	0.27	866	BV			Unknown	
10		17.252	24460	0.09	1008	VB			Unknown	

Figure S55. HPLC chromatogram of **1k**. Operative conditions: sample amount: 10 µg; column: Phenomenex Luna® 3 µM C18(2) 100 Å, 4.6x100 mm; eluent system: water/MeOH + 0.05% TFA 30:70; flow rate: 1 mL/min; detector λ: 254 nm; time: 30 min; temperature: 23 °C.

- *5-(3-Cyano-5-(octyloxy)phenyl)furan-2-carboxylic acid (**1l**)*

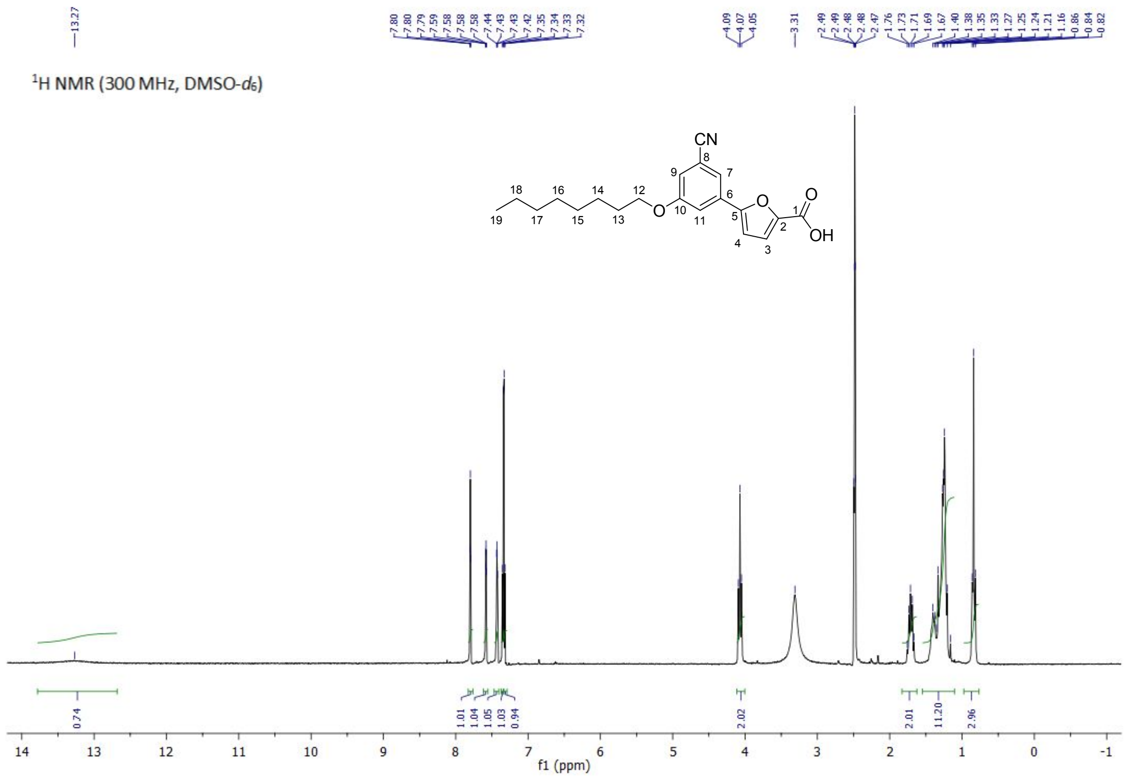

Figure S56. ^{1}H NMR spectrum of **1l**.

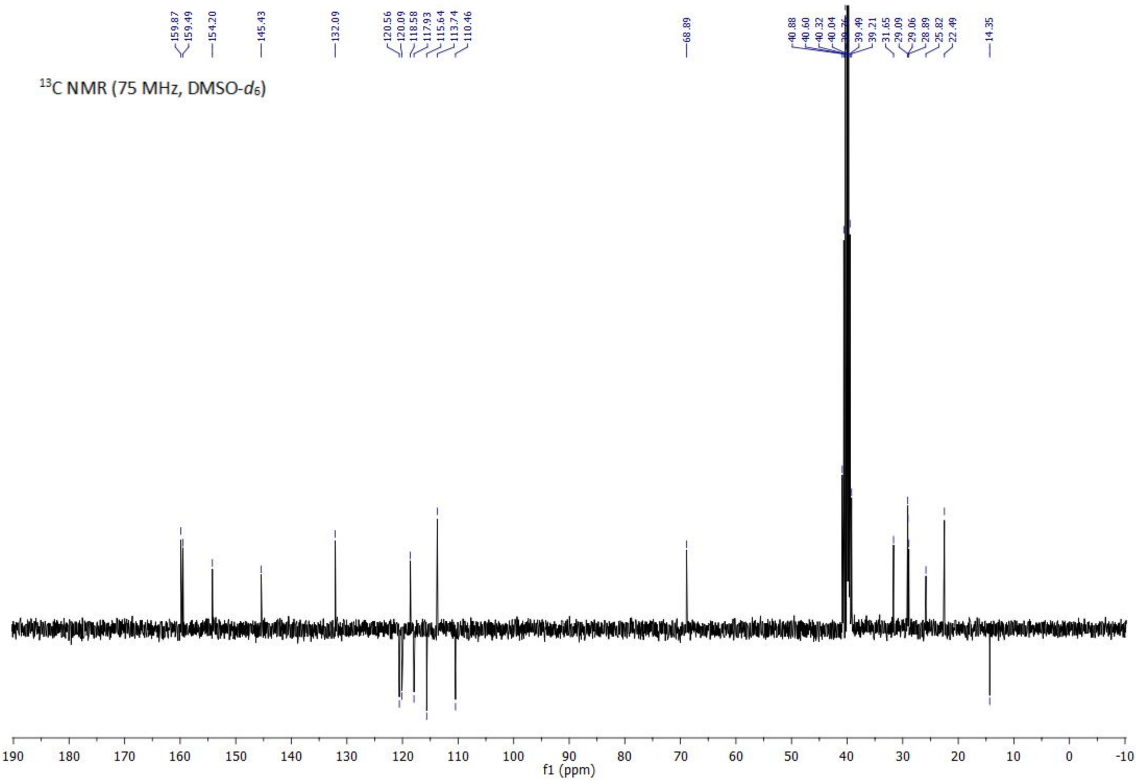

Figure S57. ^{13}C NMR spectrum of **1l**.

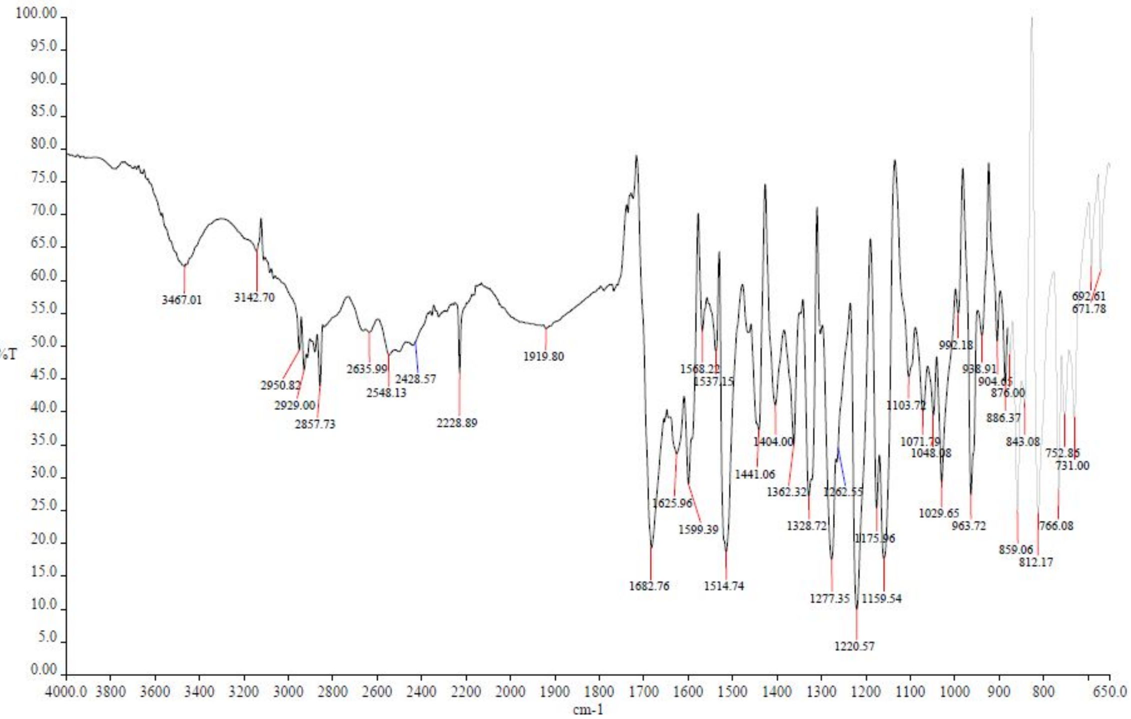

Figure S58. FT-IR spectrum of **1l**.

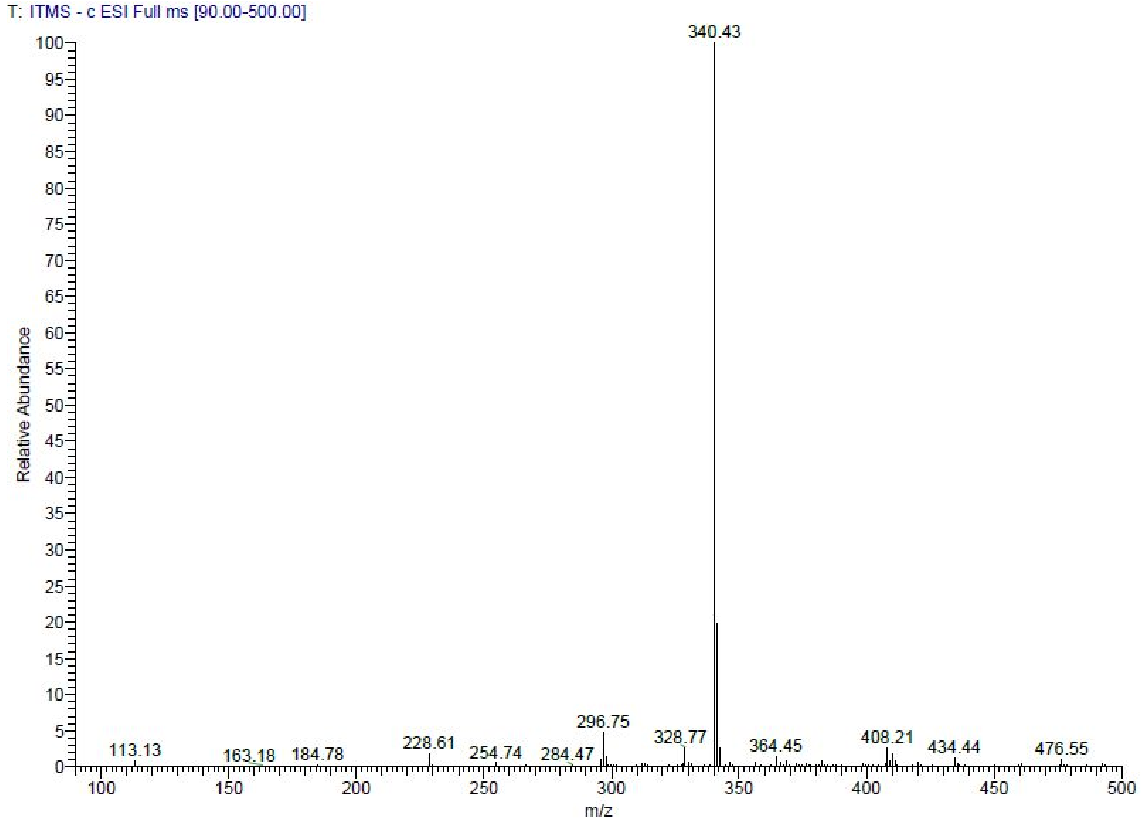

Figure S59. ESI-MS spectrum of **1l**.

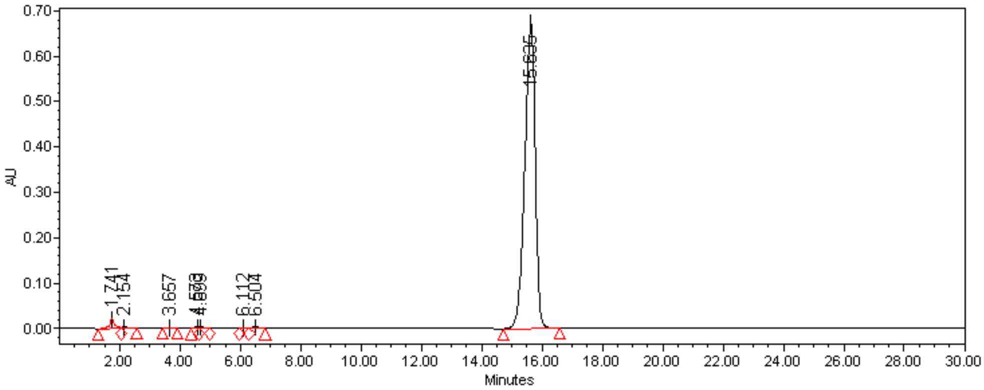

	Name	Retention Time (min)	Area (µV*sec)	% Area	Height (µV)	Int Type	Amount	Units	Peak Type	Peak Codes
1		1.741	291777	1.73	22966	BV			Unknown	
2		2.154	51705	0.31	4233	VB			Unknown	
3		3.657	19261	0.11	2180	BB			Unknown	
4		4.573	40361	0.24	4674	BV			Unknown	
5		4.699	44997	0.27	4171	VV			Unknown	
6		6.112	28795	0.17	2277	VV			Unknown	
7		6.504	55456	0.33	4431	VB			Unknown	
8		15.635	16299363	96.84	671209	BB			Unknown	

Figure S60. HPLC chromatogram of **1l**. Operative conditions: sample amount: 10 µg; column: Phenomenex Luna® 3 µM C18(2) 100 Å, 4.6x100 mm; eluent system: water/MeOH + 0.05% TFA 30:70; flow rate: 1 mL/min; detector λ: 254 nm; time: 30 min; temperature: 23 °C.

- *5-(3-(But-2-en-1-yloxy)-5-cyanophenyl)furan-2-carboxylic acid (**1m**)*

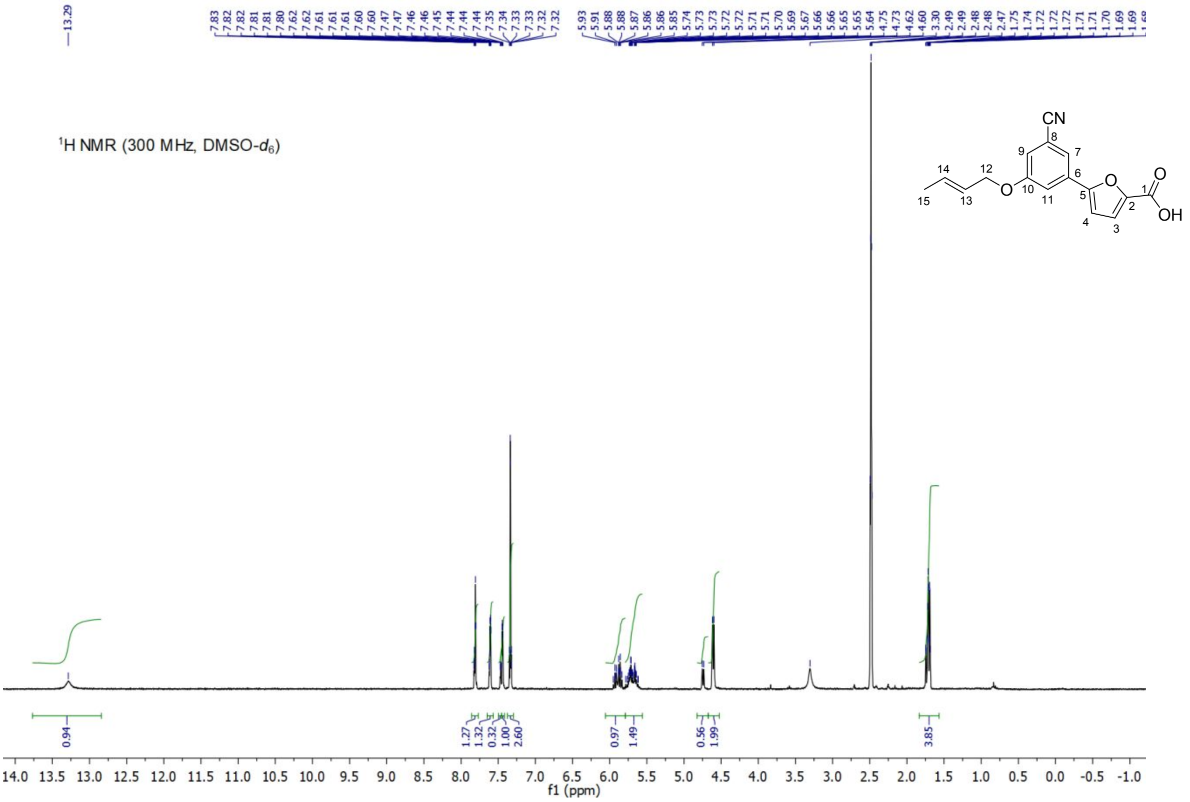

Figure S61. ^{1}H NMR spectrum of **1m**.

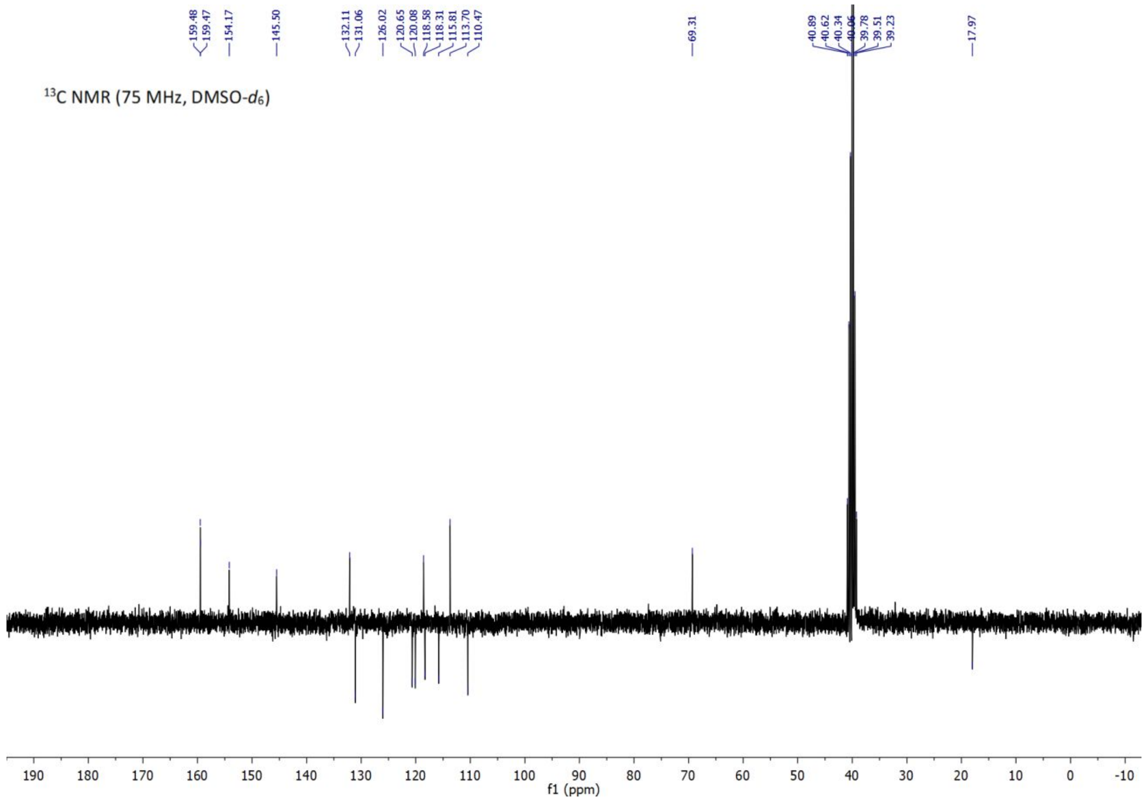

Figure S62. ^{13}C NMR spectrum of **1m**.

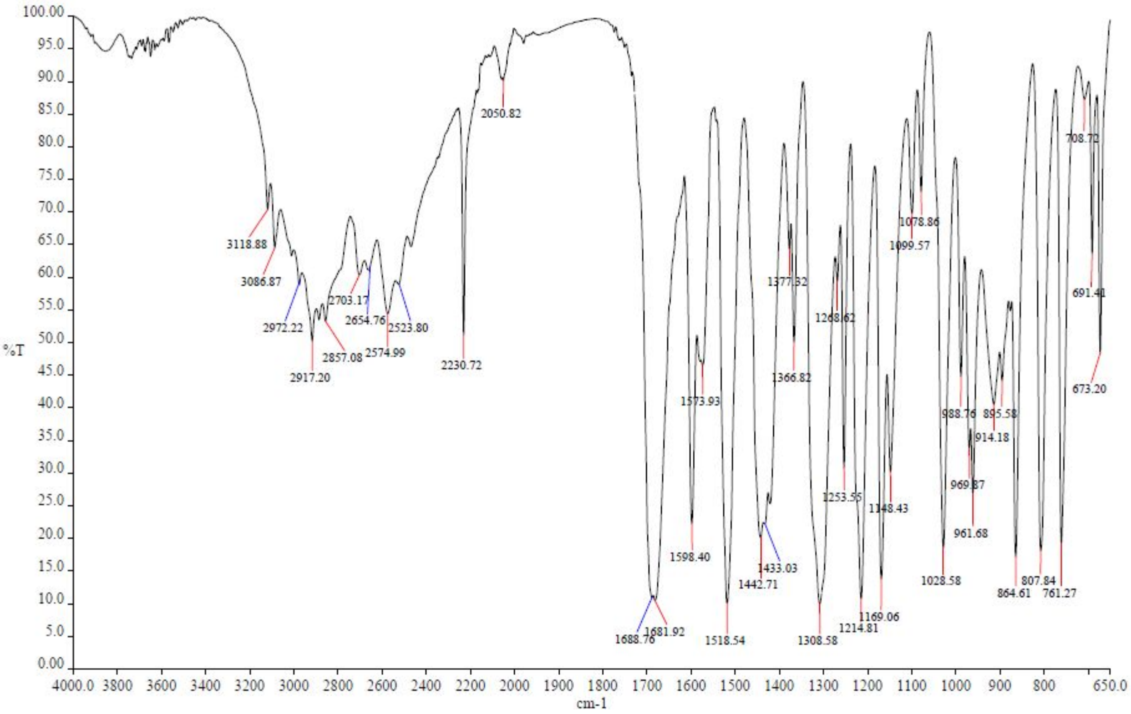

Figure S63. FT-IR spectrum of **1m**.

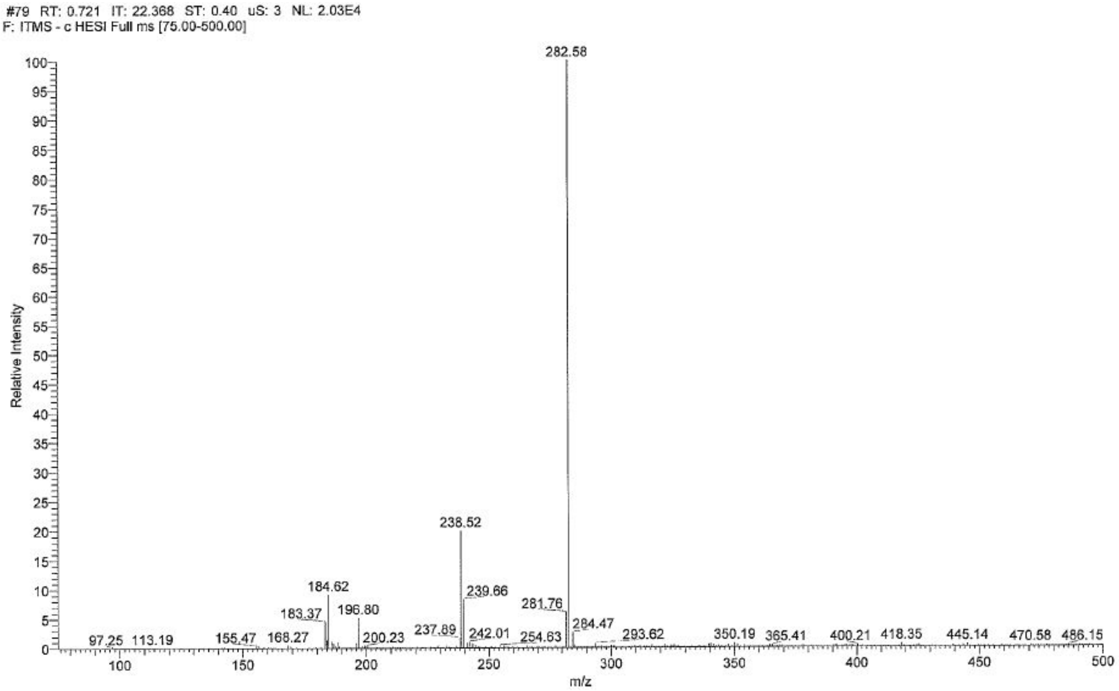

Figure S64. ESI-MS spectrum of **1m**.

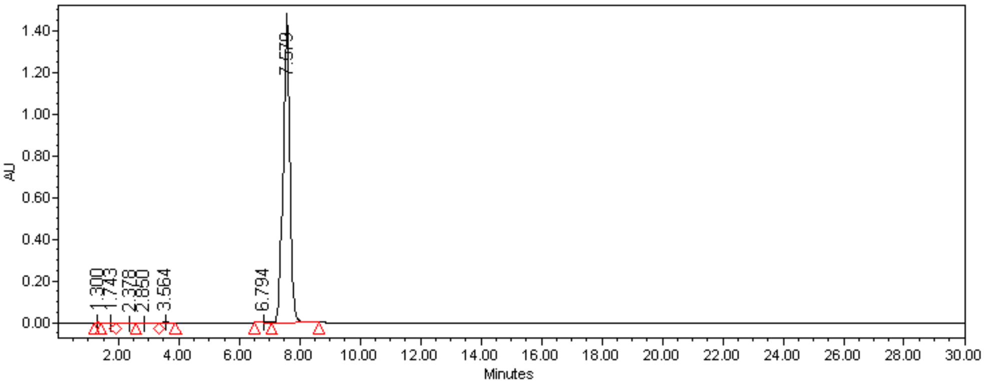

	Name	Retention Time (min)	Area (µV*sec)	% Area	Height (µV)	Int Type	Amount	Units	Peak Type	Peak Codes
1		1.300	16684	0.07	2458	BB			Unknown	
2		1.743	41111	0.18	2696	BV			Unknown	
3		2.378	84565	0.37	2359	VB			Unknown	
4		2.850	58423	0.26	1848	BV			Unknown	I08
5		3.564	50688	0.22	3965	VB			Unknown	
6		6.794	41005	0.18	3510	BB			Unknown	
7		7.579	22464599	98.71	1447366	BB			Unknown	

Figure S65. HPLC chromatogram of **1m**. Operative conditions: sample amount: 10 µg; column: Phenomenex Luna® 3 µM C18(2) 100 Å, 4.6x100 mm; eluent system: water/MeOH + 0.05% TFA 30:70; flow rate: 1 mL/min; detector λ: 254 nm; time: 30 min; temperature: 23 °C.

- *5-(3-Cyano-5-((2E,4E)-hexa-2,4-dien-1-yloxy)phenyl)furan-2-carboxylic acid (**1n**)*

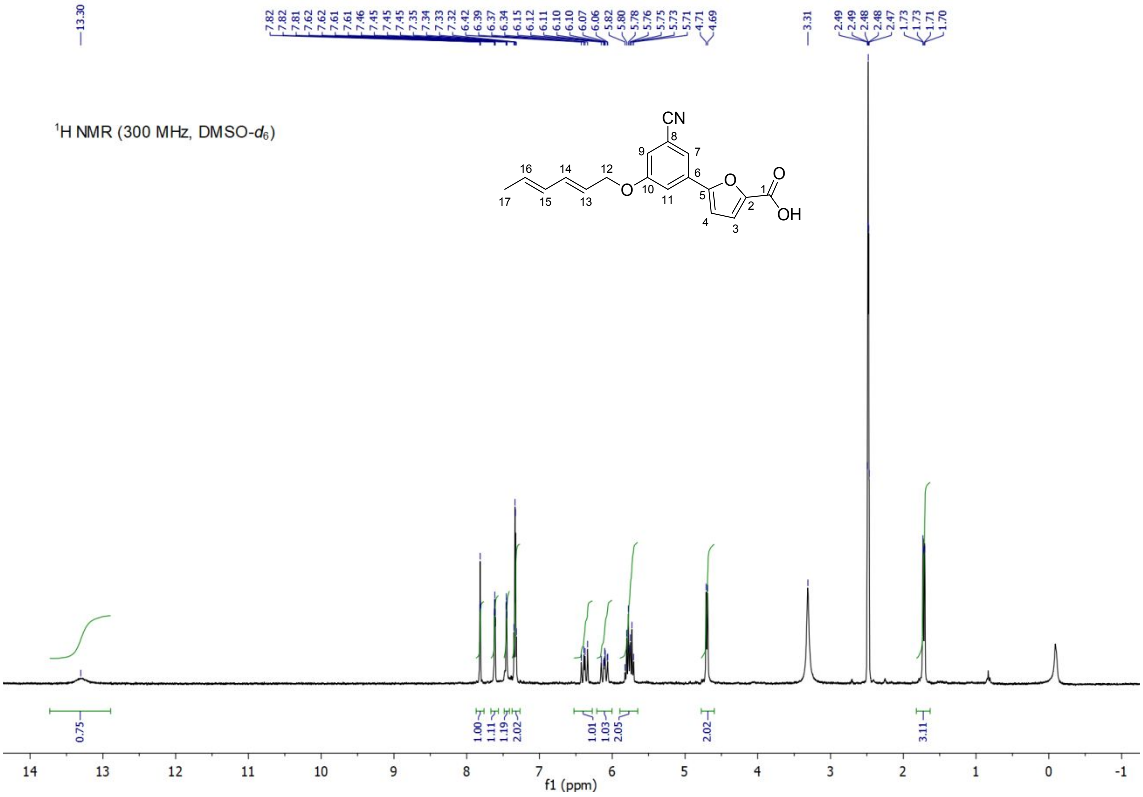

Figure S66. ^{1}H NMR spectrum of **1n**.

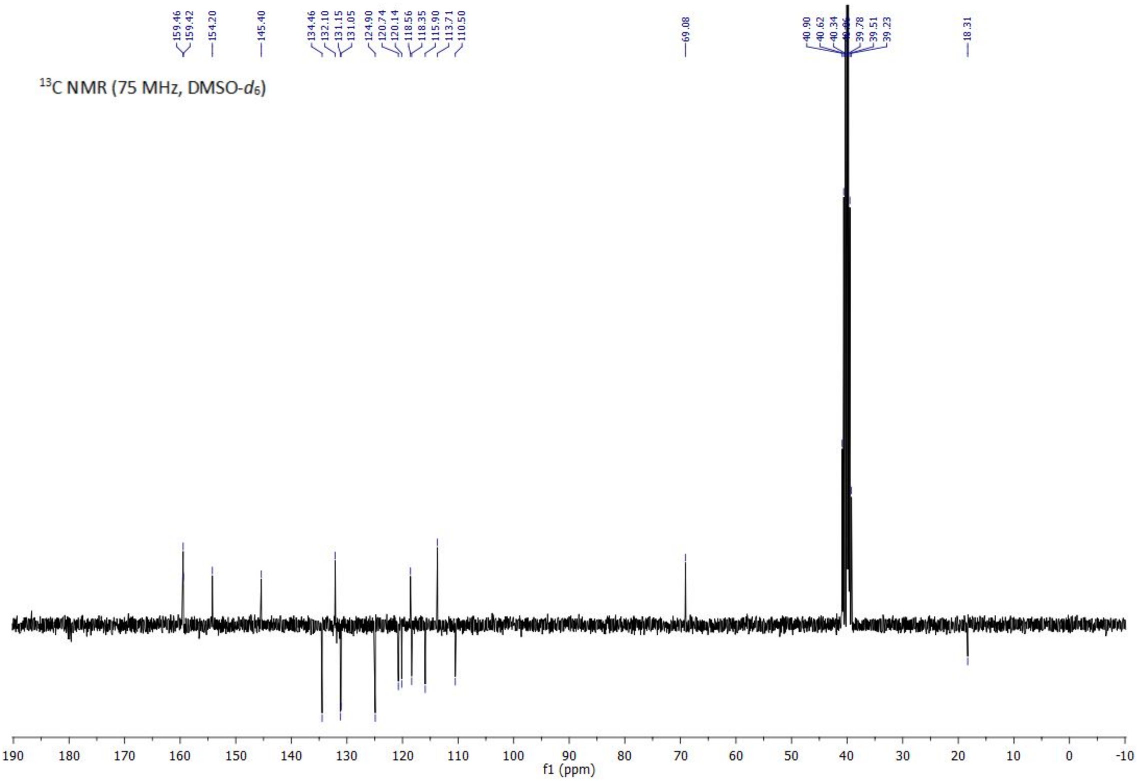

Figure S67. ^{13}C NMR spectrum of **1n**.

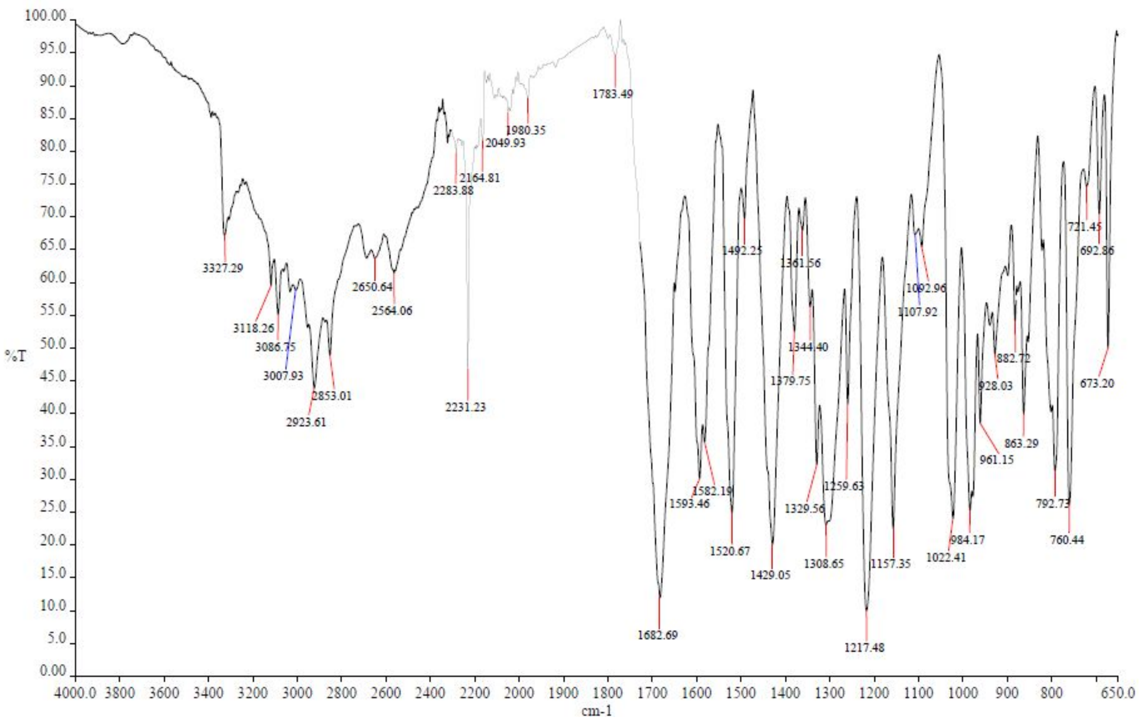

Figure S68. FT-IR spectrum of **1n**.

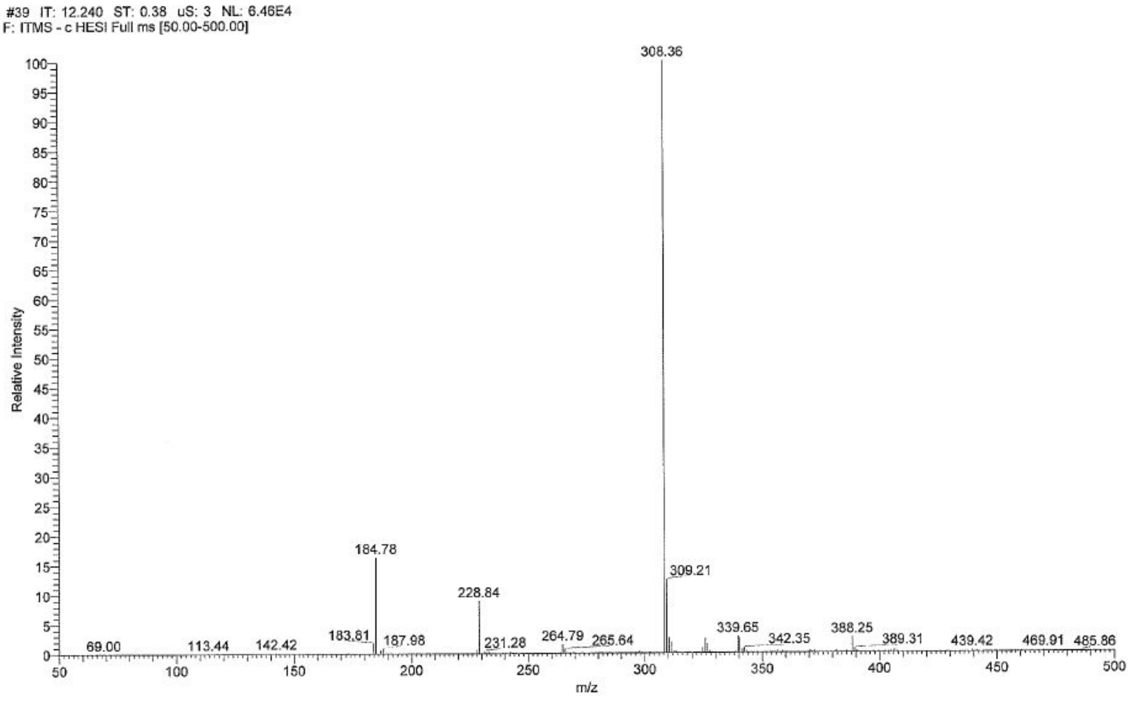

Figure S69. ESI-MS spectrum of **1n**.

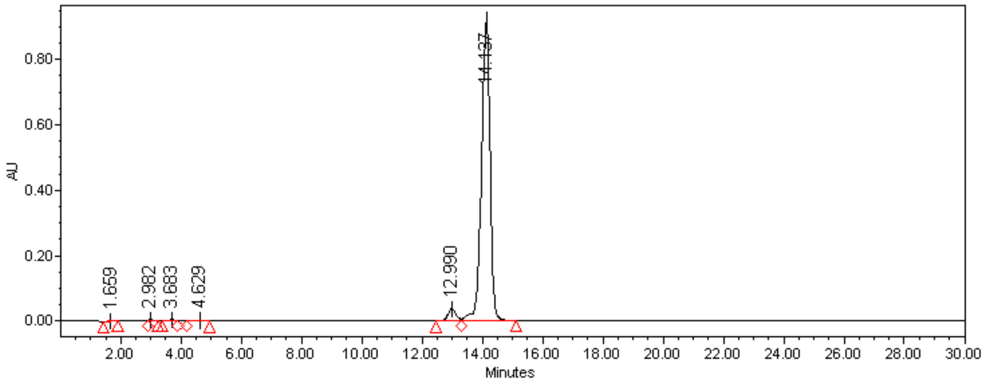

	Name	Retention Time (min)	Area (µV*sec)	% Area	Height (µV)	Int Type	Amount	Units	Peak Type	Peak Codes
1		1.659	49897	0.26	3848	BB			Unknown	
2		2.982	42818	0.22	5830	VB			Unknown	
3		3.683	54472	0.28	4151	BV			Unknown	
4		4.629	48136	0.25	2868	VB			Unknown	
5		12.990	729909	3.73	38370	BV			Unknown	
6		14.137	18618002	95.27	917921	VB			Unknown	

Figure S70. HPLC chromatogram of **1n**. Operative conditions: sample amount: 10 µg; column: Phenomenex Luna® 3 µM C18(2) 100 Å, 4.6x100 mm; eluent system: water/MeOH + 0.05% TFA 30:70; flow rate: 1 mL/min; detector λ: 254 nm; time: 30 min; temperature: 23 °C.

- *5-(3-Cyano-5-((2E,4E,6E)-octa-2,4,6-trien-1-yloxy)phenyl)furan-2-carboxylic acid (**1o**)*

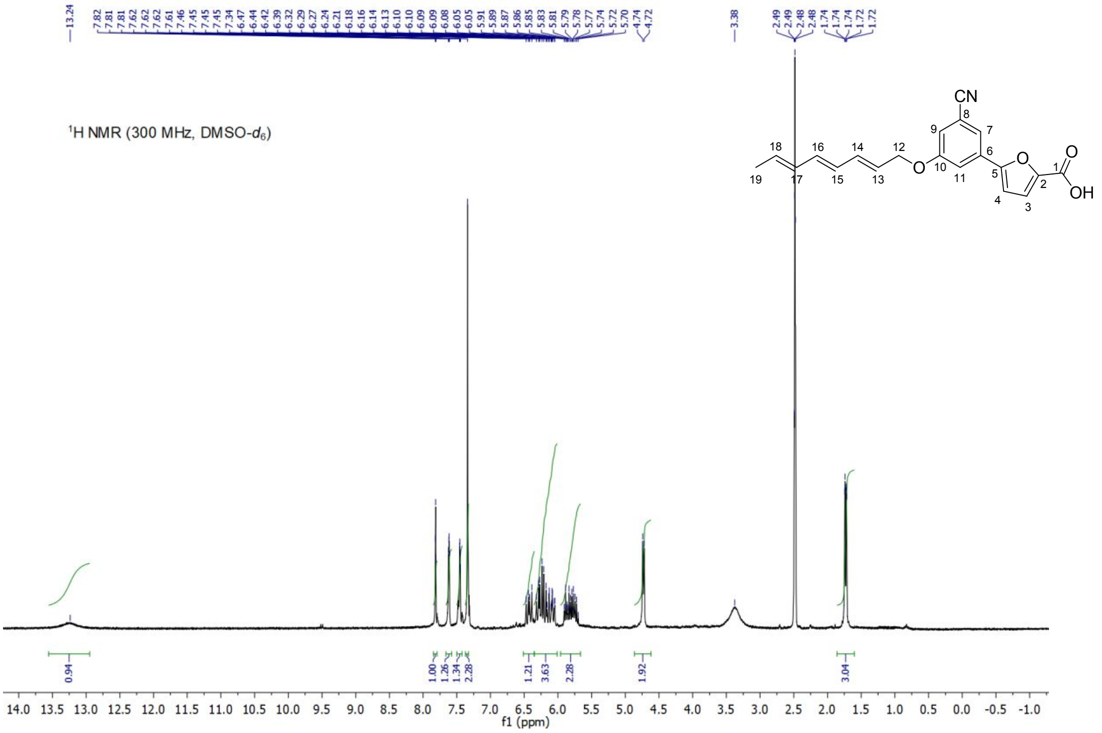

Figure S71. ^{1}H NMR spectrum of **1o**.

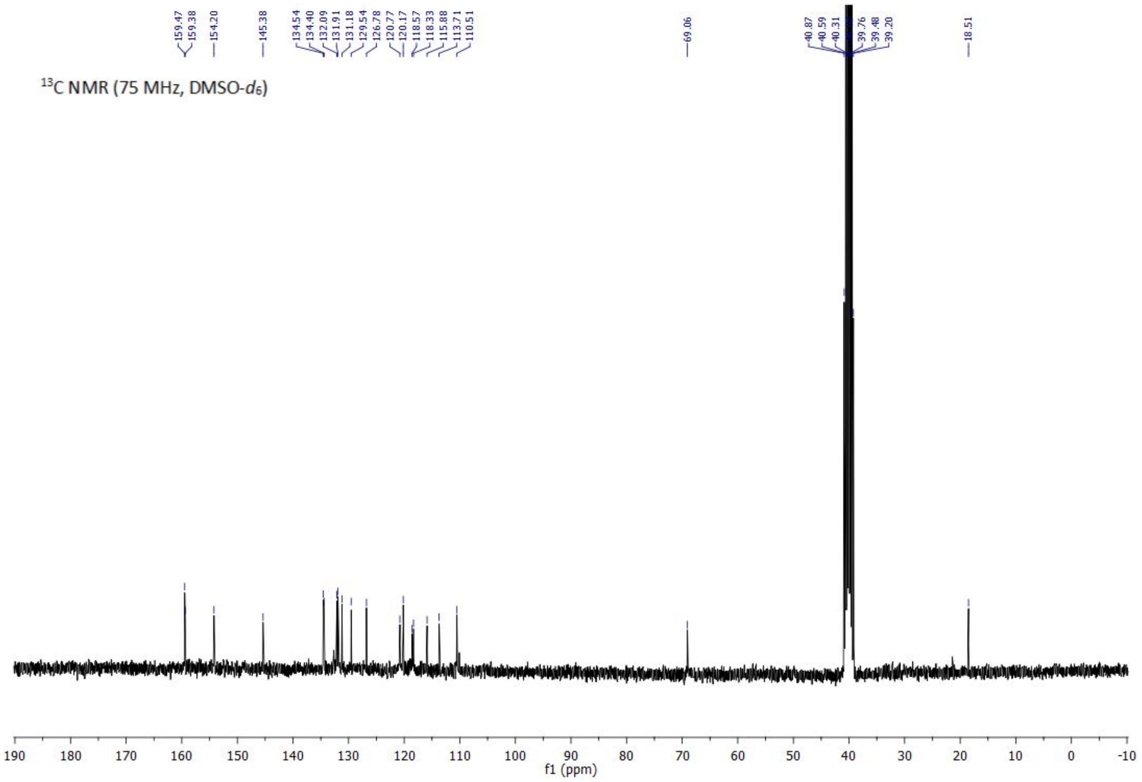

Figure S72. ^{13}C NMR spectrum of **1o**.

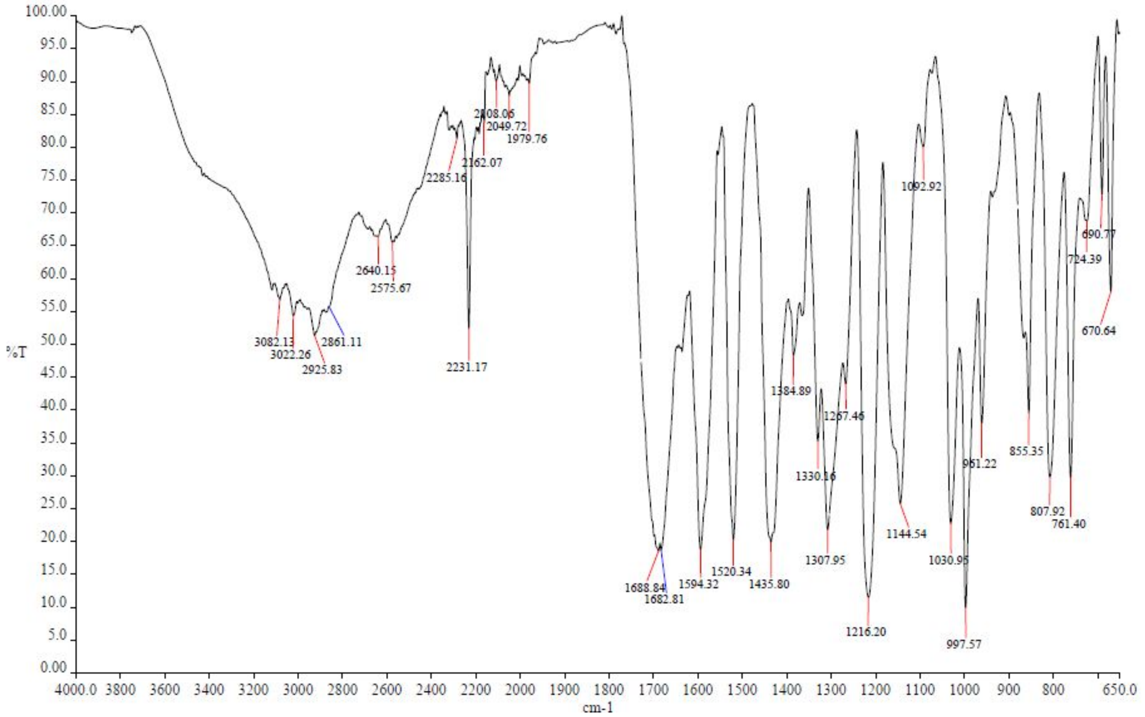

Figure S73. FT-IR spectrum of **1o**.

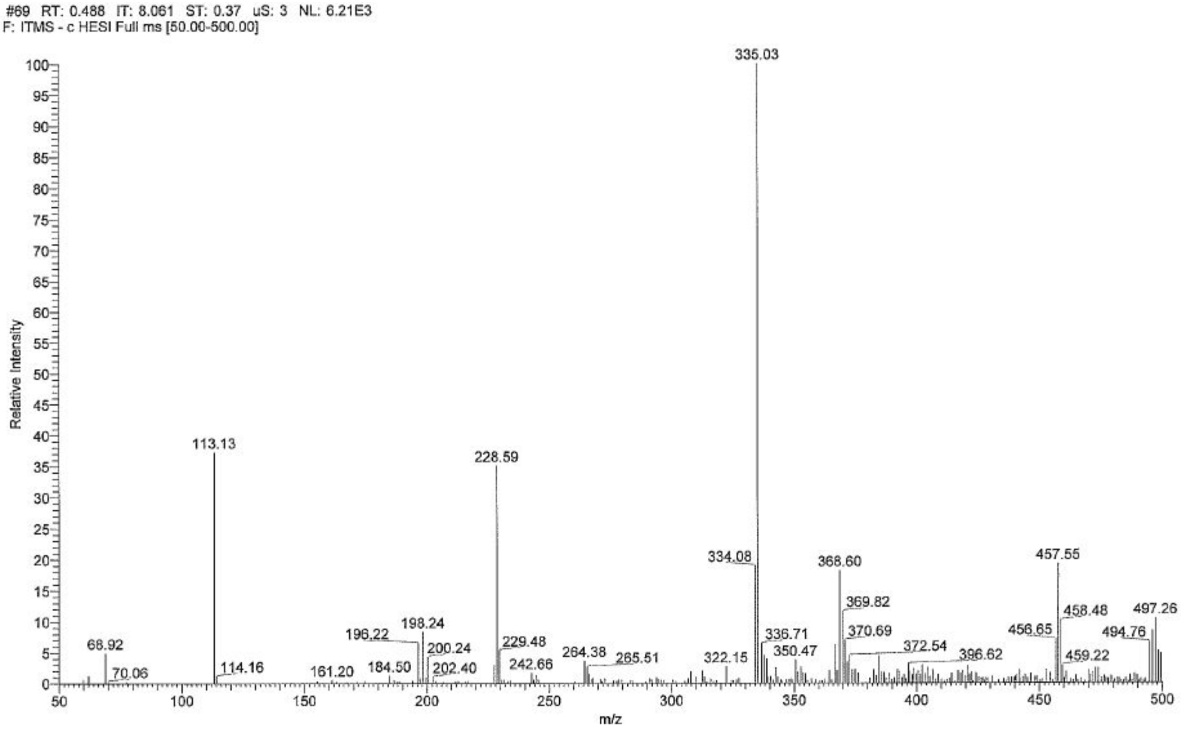

Figure S74. ESI-MS spectrum of **1o**.

- *Ethyl 5-(3-cyano-5-isobutoxyphenyl)furan-2-carboxylate (**2ea**)*

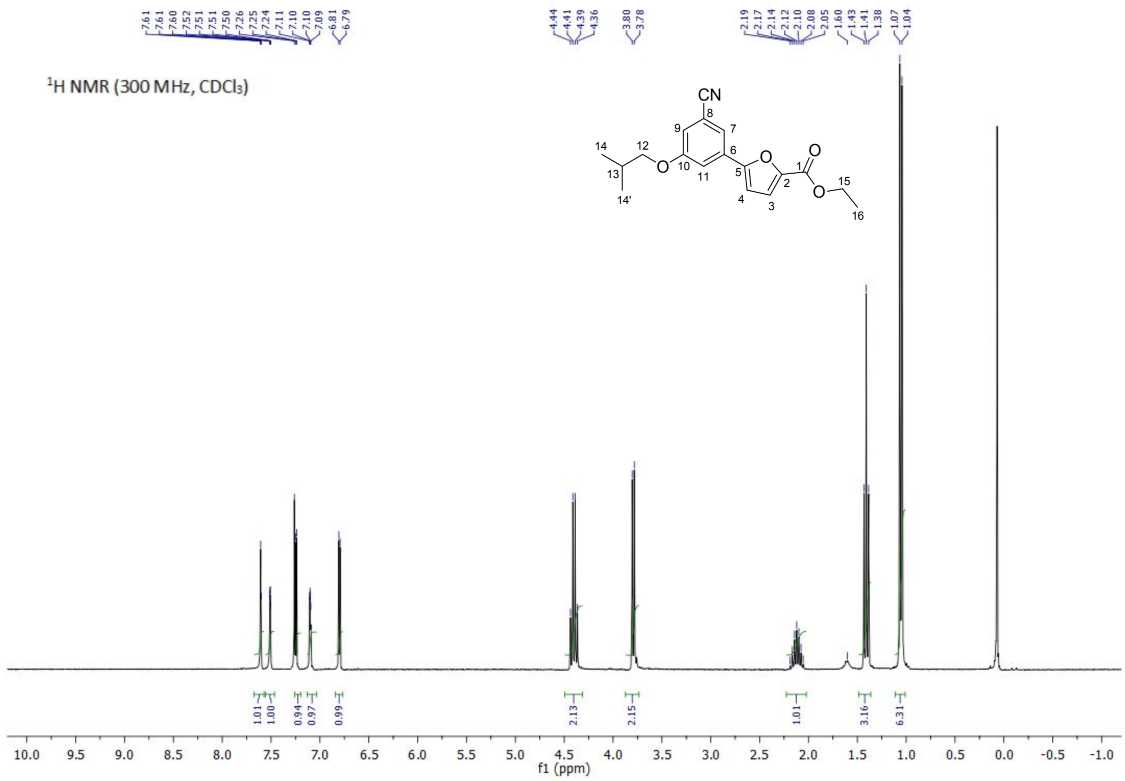

Figure S75. ^{1}H NMR spectrum of **2ea**.

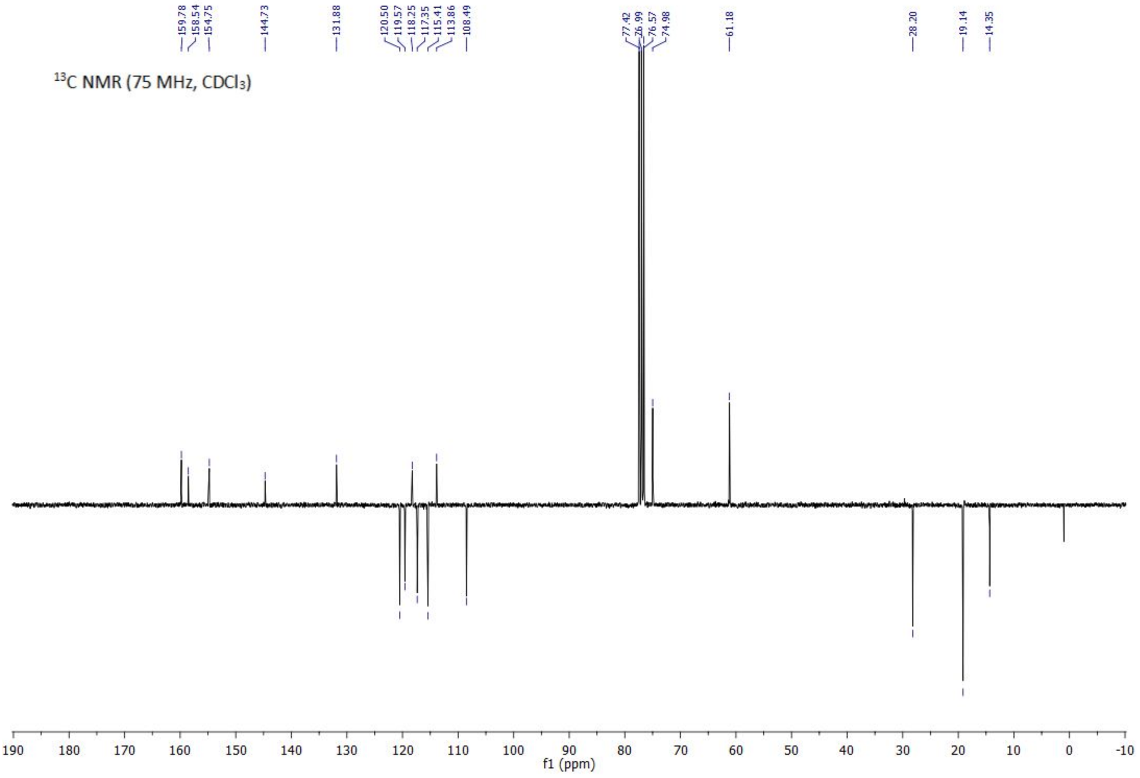

Figure S76. ^{13}C NMR spectrum of **2ea**.

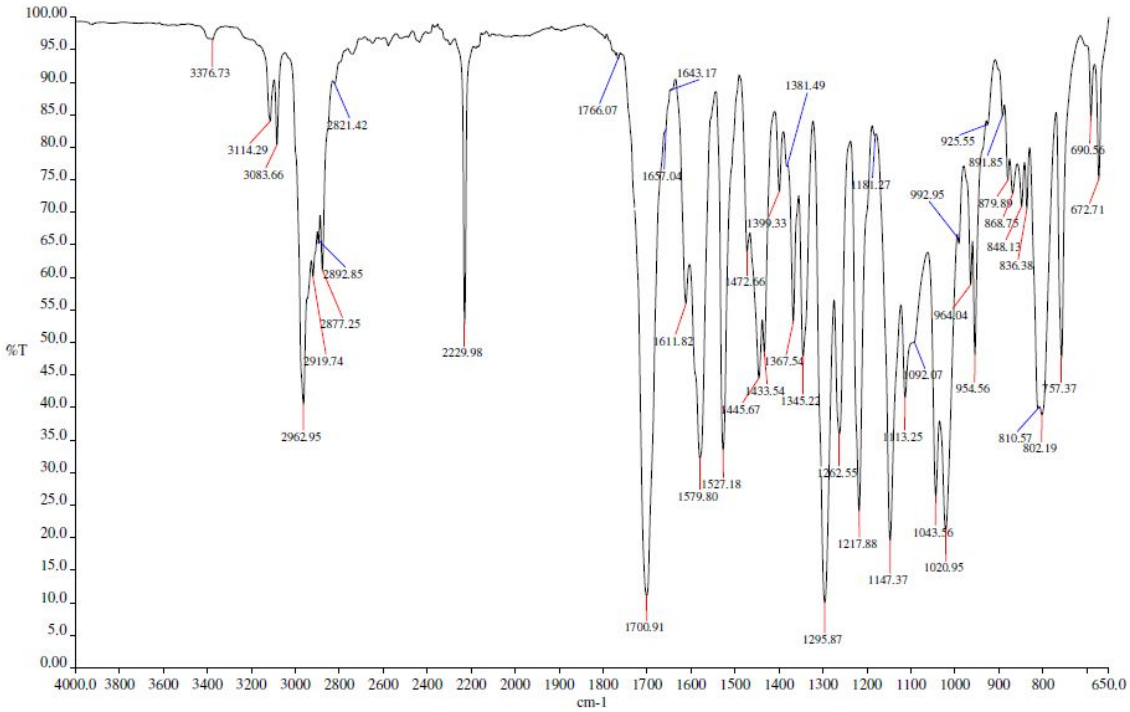

Figure S77. FT-IR spectrum of **2ea**.

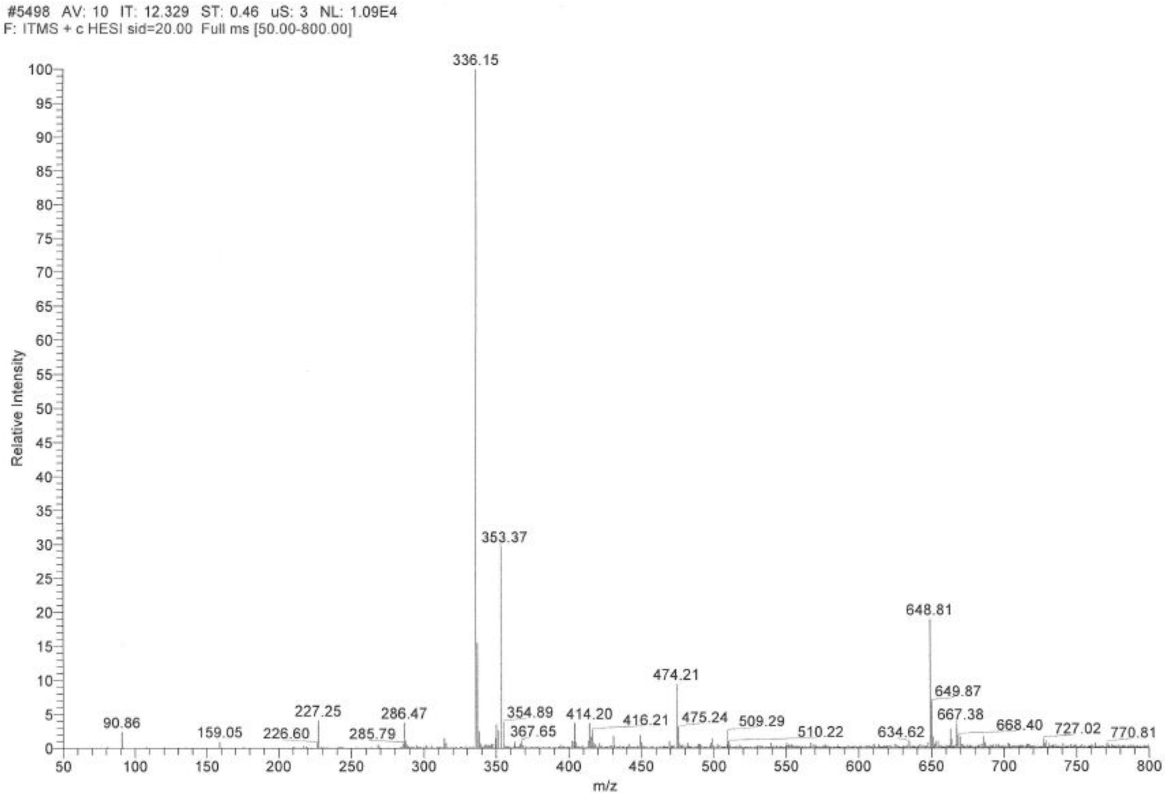

Figure S78. ESI-MS spectrum of **2ea**.

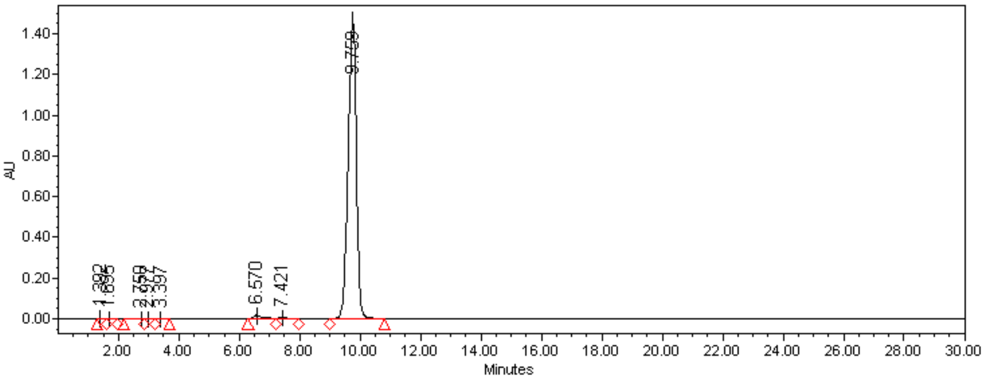

	Name	Retention Time (min)	Area (µV*sec)	% Area	Height (µV)	Int Type	Amount	Units	Peak Type	Peak Codes
1		1.392	45028	0.16	4318	BV			Unknown	
2		1.695	39885	0.14	2368	VV			Unknown	
3		2.750	46412	0.16	1639	BV			Unknown	I08
4		2.977	28975	0.10	1608	VV			Unknown	
5		3.397	27835	0.10	1797	VB			Unknown	
6		6.570	307130	1.08	15786	BV			Unknown	
7		7.421	105128	0.37	6116	VV			Unknown	
8		9.759	27777363	97.88	1466679	VB			Unknown	

Figure S79. HPLC chromatogram of **2ea**. Operative conditions: sample amount: 10 µg; column: Phenomenex Luna® 3 µM C18(2) 100 Å, 4.6x100 mm; eluent system: water/MeOH + 0.05% TFA 20:80; flow rate: 1 mL/min; detector λ: 254 nm; time: 30 min; temperature: 23 °C.

- *Propyl 5-(3-cyano-5-isobutoxyphenyl)furan-2-carboxylate (**2eb**)*

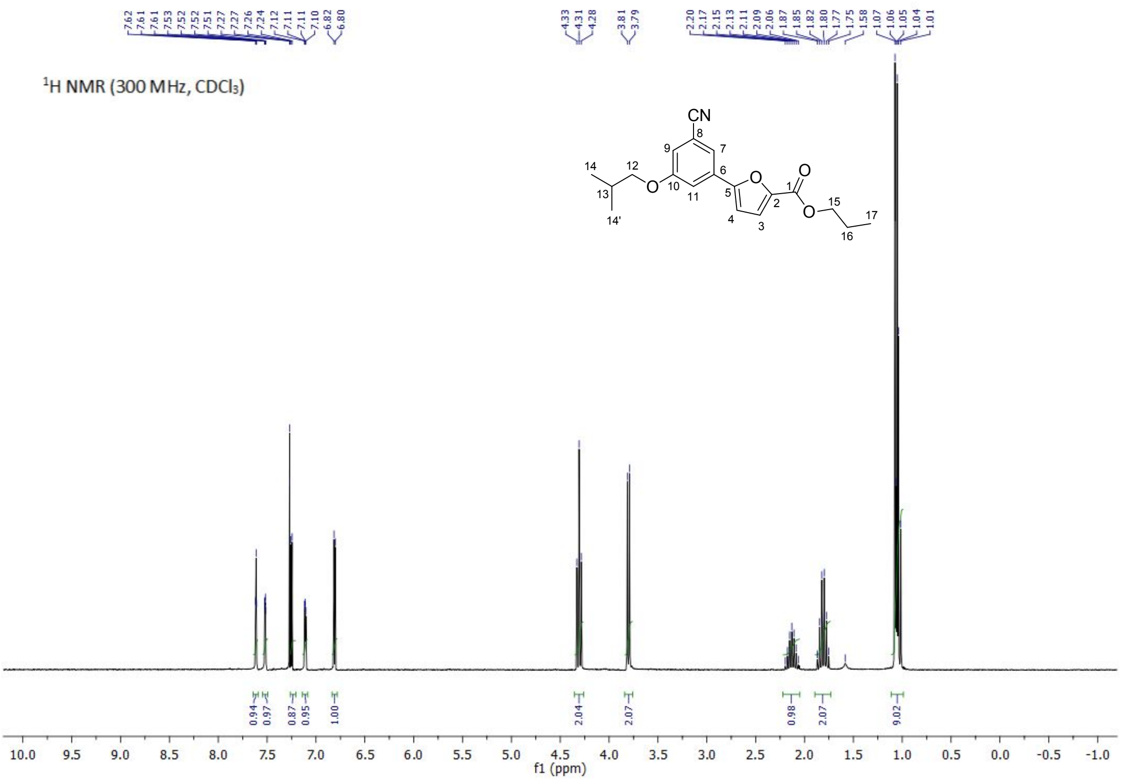

Figure S80. ^{1}H NMR spectrum of **2eb**.

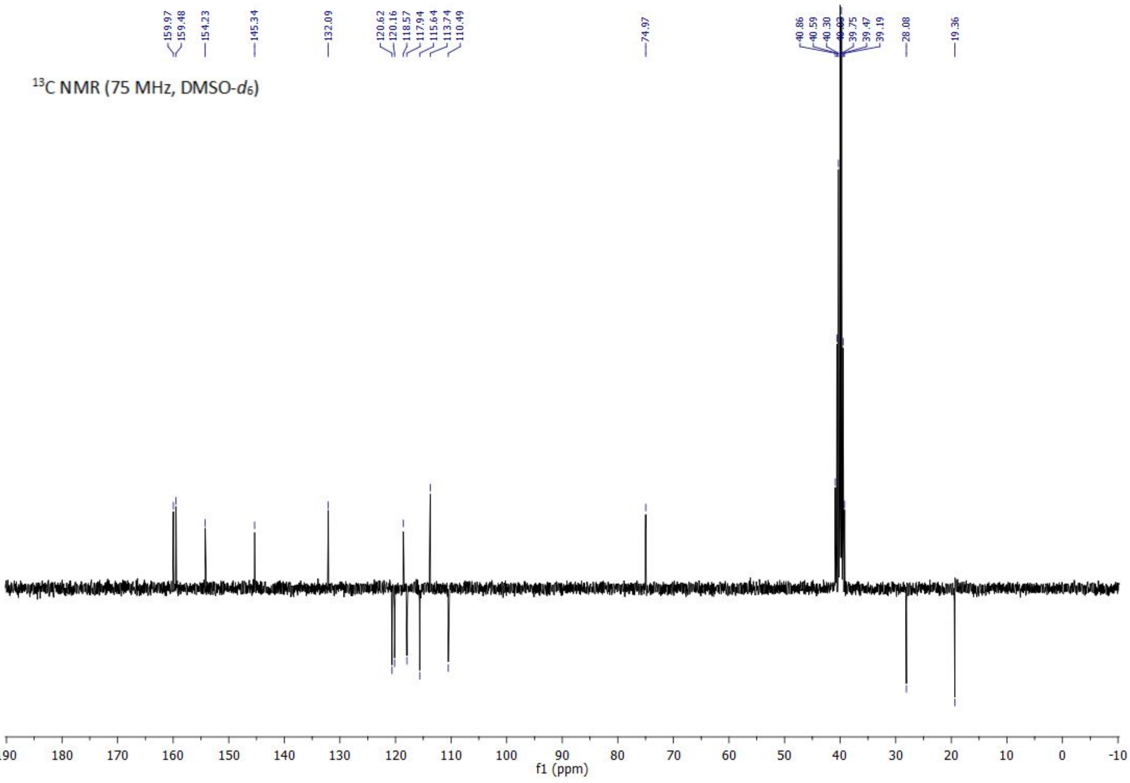

Figure S81. ^{13}C NMR spectrum of **2eb**.

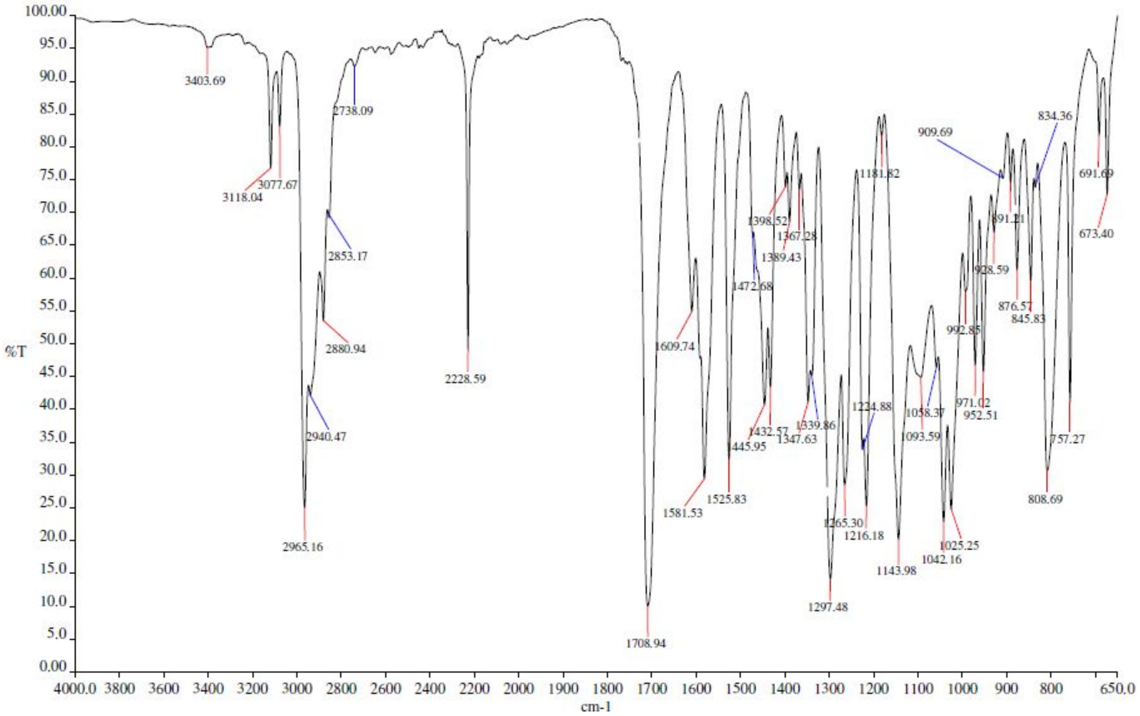

Figure S82. FT-IR spectrum of **2eb**.

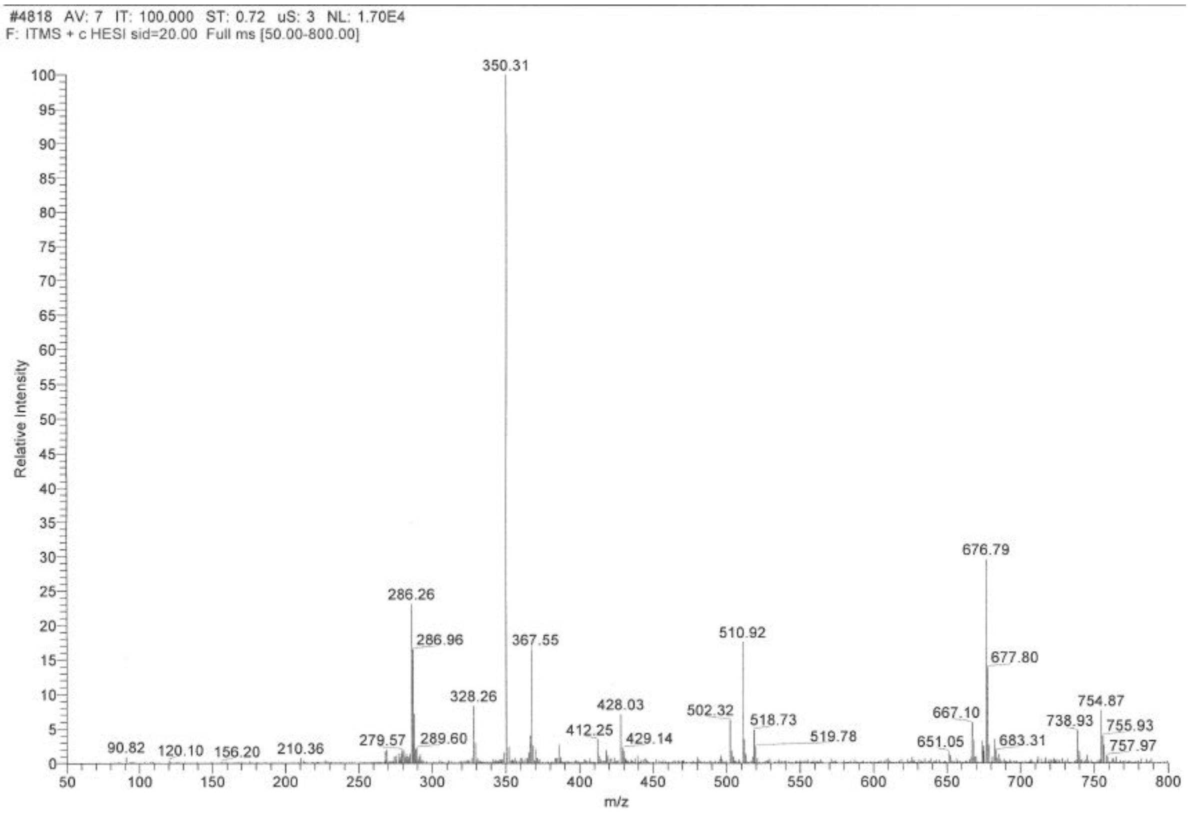

Figure S83. ESI-MS spectrum of **2eb**.

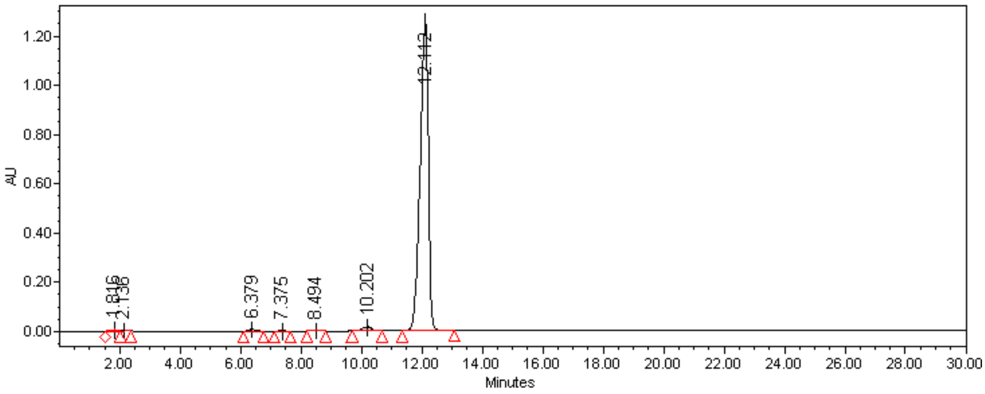

	Name	Retention Time (min)	Area (µV*sec)	% Area	Height (µV)	Int Type	Amount	Units	Peak Type	Peak Codes
1		1.816	69069	0.28	6471	VB			Unknown	
2		2.136	37925	0.15	4847	BB			Unknown	
3		6.379	108213	0.44	8666	BB			Unknown	
4		7.375	18857	0.08	1369	BB			Unknown	
5		8.494	26752	0.11	1655	BB			Unknown	
6		10.202	364059	1.49	16715	BB			Unknown	
7		12.112	23863356	97.45	1259394	BB			Unknown	

Figure S84. HPLC chromatogram of **2eb**. Operative conditions: sample amount: 10 µg; column: Phenomenex Luna® 3 µM C18(2) 100 Å, 4.6x100 mm; eluent system: water/MeOH + 0.05% TFA 20:80; flow rate: 1 mL/min; detector λ: 254 nm; time: 30 min; temperature: 23 °C.

- *Methyl 5-(3-cyano-5-ethoxyphenyl)furan-2-carboxylate (**2a**)*

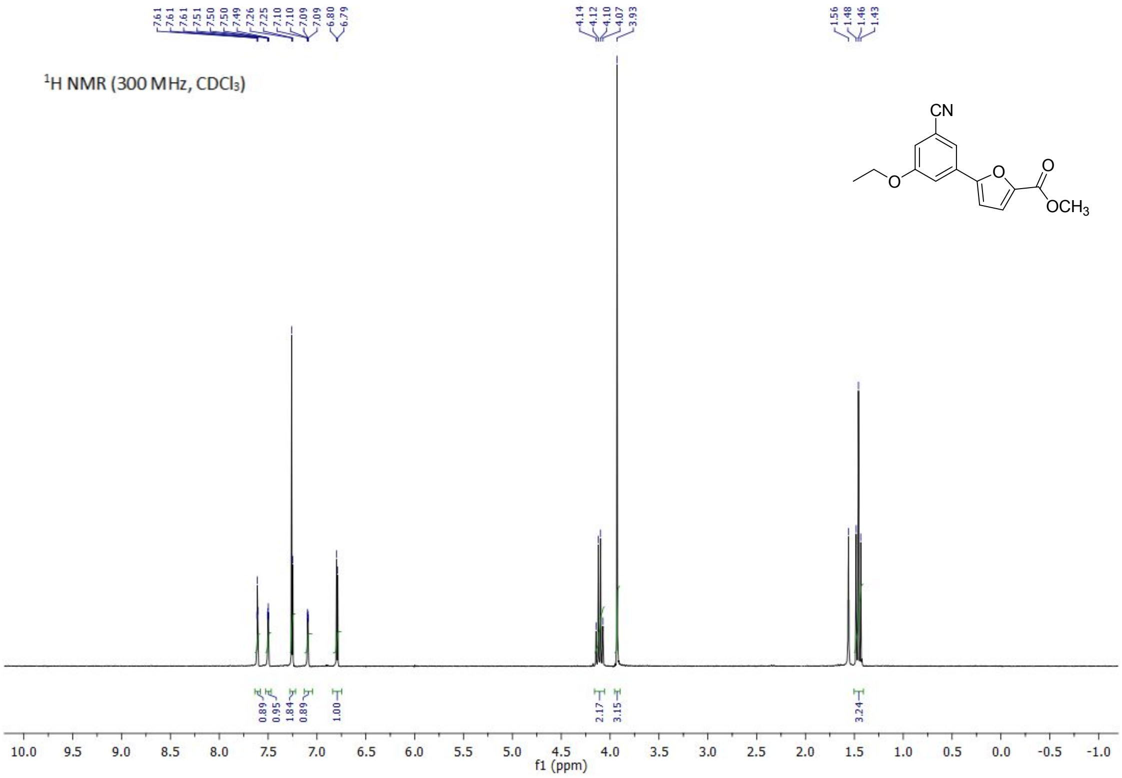

Figure S85. ^{1}H NMR spectrum of **2a**.

- *Methyl 5-(3-cyano-5-isopropoxyphenyl)furan-2-carboxylate (**2b**)*

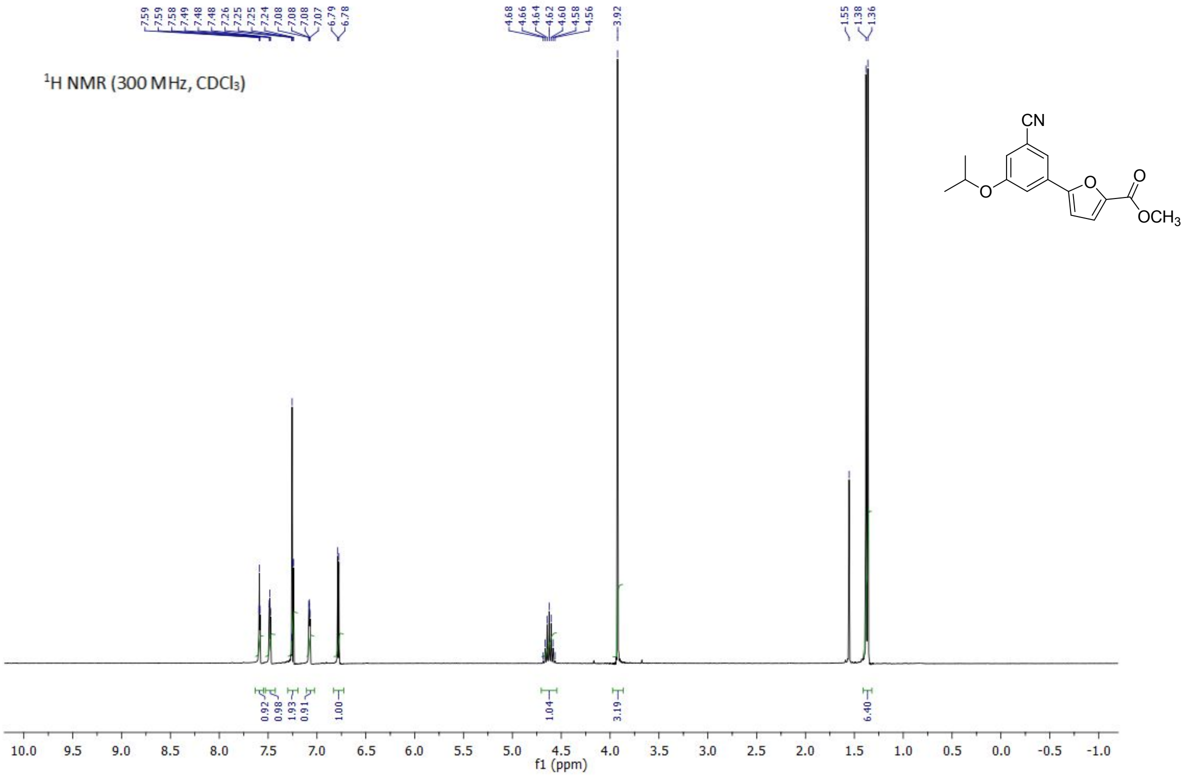

Figure S86. ^{1}H NMR spectrum of **2b**.

- *Methyl 5-(3-cyano-5-propoxyphenyl)furan-2-carboxylate (**2c**)*

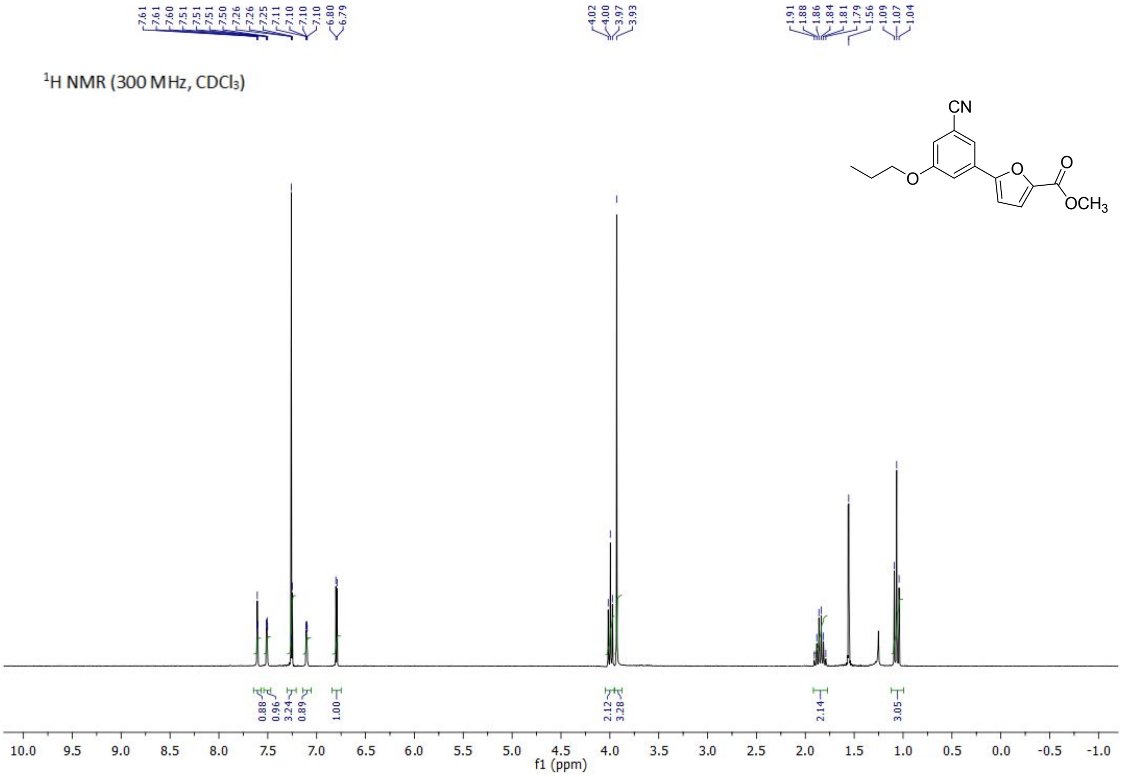

Figure S87. ^{1}H NMR spectrum of **2c**.

- *Methyl 5-(3-butoxy-5-cyanophenyl)furan-2-carboxylate (**2d**)*

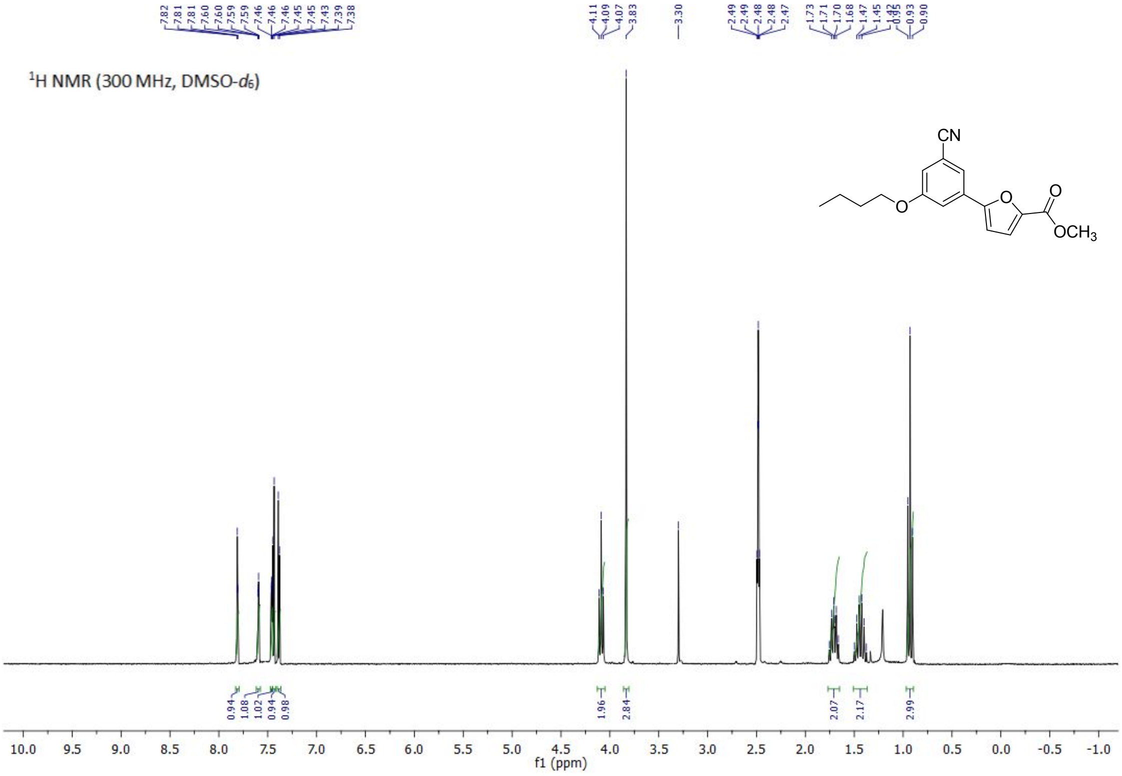

Figure S88. ^{1}H NMR spectrum of **2d**.

- *Methyl 5-(3-cyano-5-isobutoxyphenyl)furan-2-carboxylate (**2e**)*

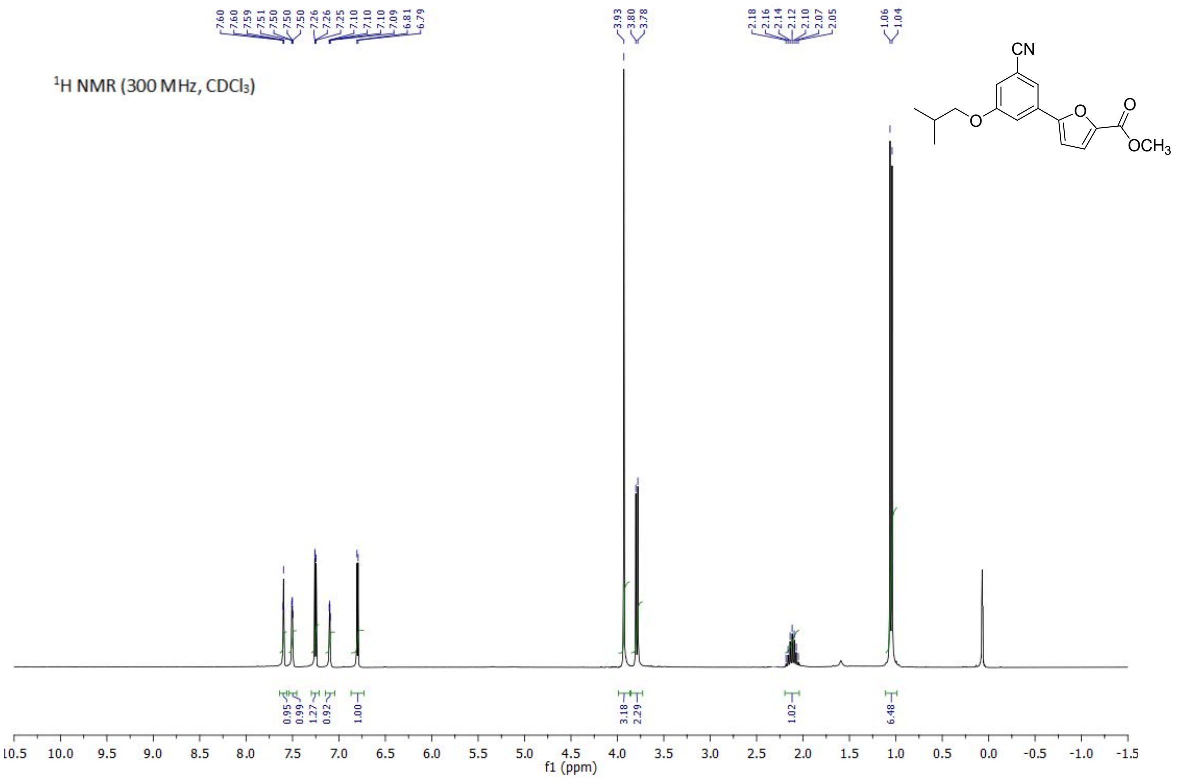

Figure S89. ^{1}H NMR spectrum of **2e**.

- *Methyl 5-(3-cyano-5-(cyclopropylmethoxy)phenyl)furan-2-carboxylate (**2f**)*

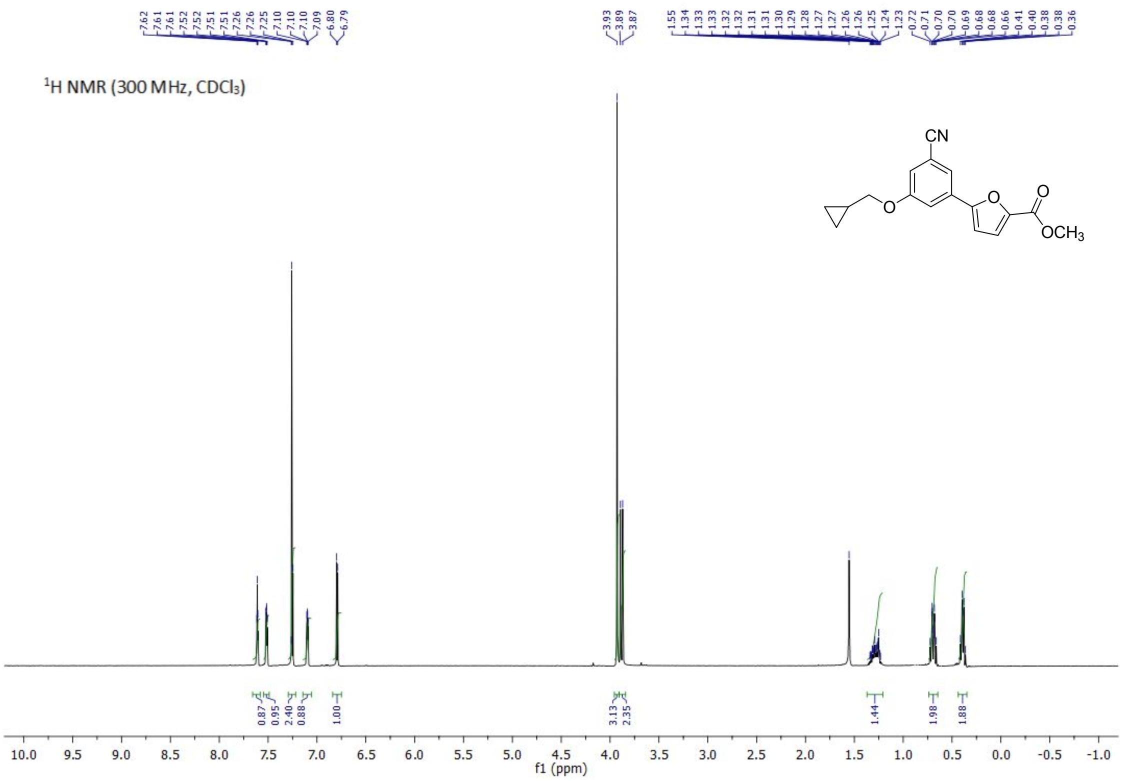

Figure S90. ^{1}H NMR spectrum of **2f**.

- *Methyl 5-(3-cyano-5-(cyclopropylmethoxy)phenyl)furan-2-carboxylate (**2g**)*

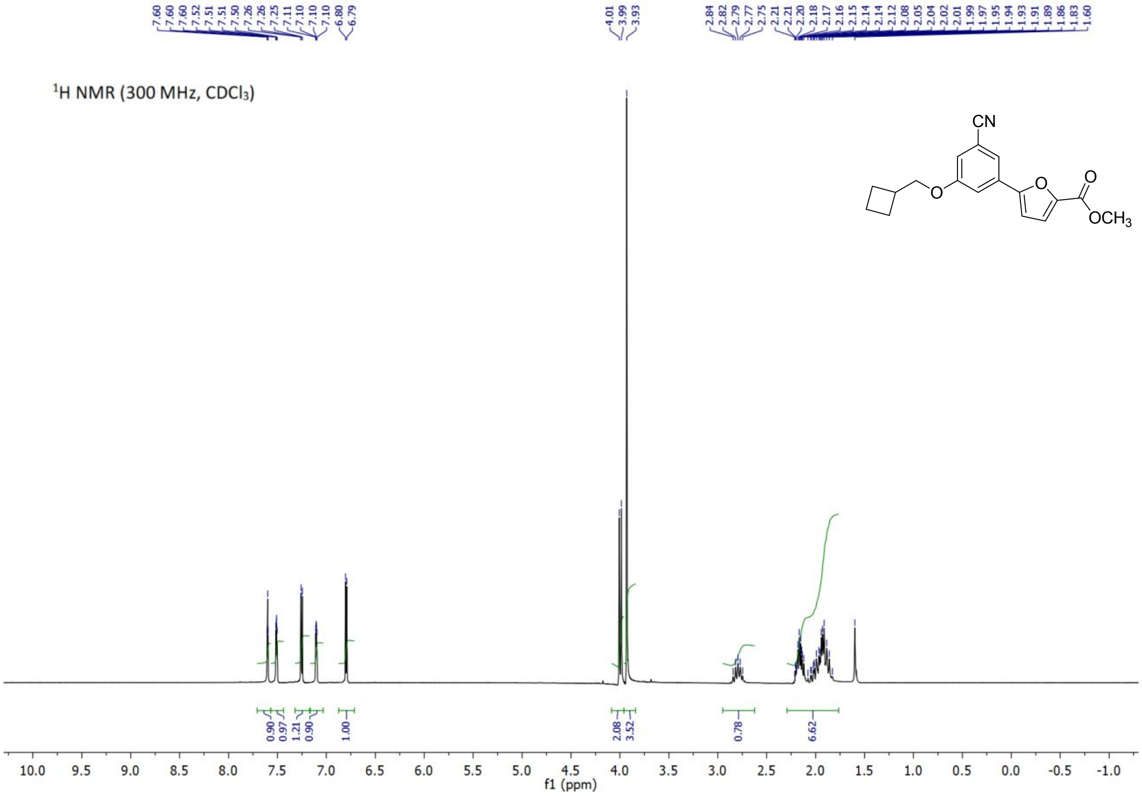

Figure S91. ^{1}H NMR spectrum of **2g**.

- *Methyl 5-(3-cyano-5-(neopentyloxy)phenyl)furan-2-carboxylate (**2h**)*

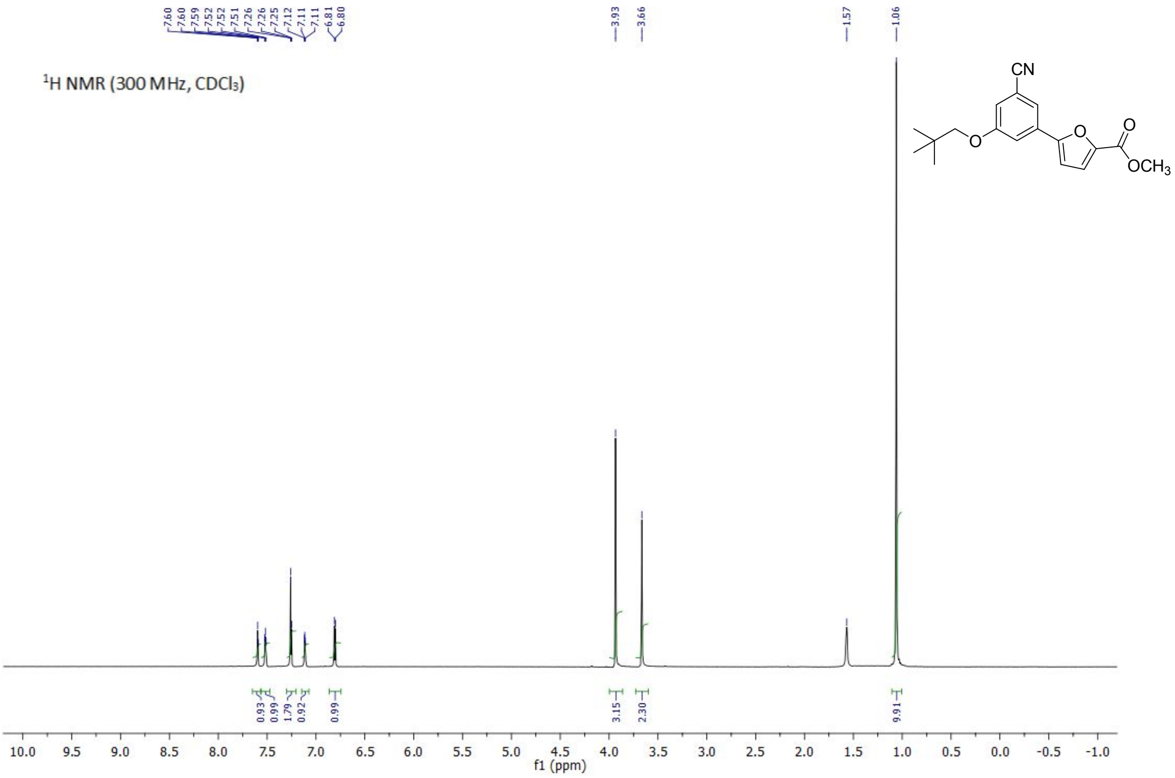

Figure S92. ^{1}H NMR spectrum of **2h**.

- *Methyl 5-(3-cyano-5-(pentyloxy)phenyl)furan-2-carboxylate (**2i**)*

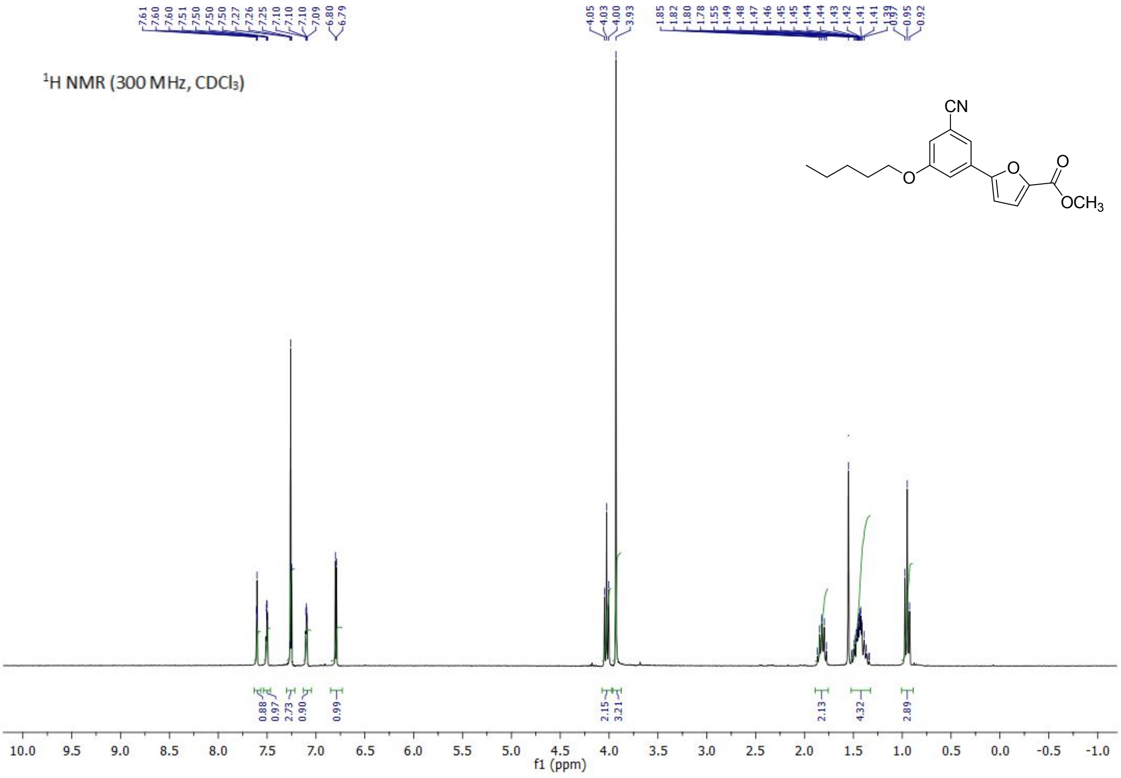

Figure S93. ^{1}H NMR spectrum of **2i**.

- *Methyl 5-(3-cyano-5-(isopentyloxy)phenyl)furan-2-carboxylate (**2j**)*

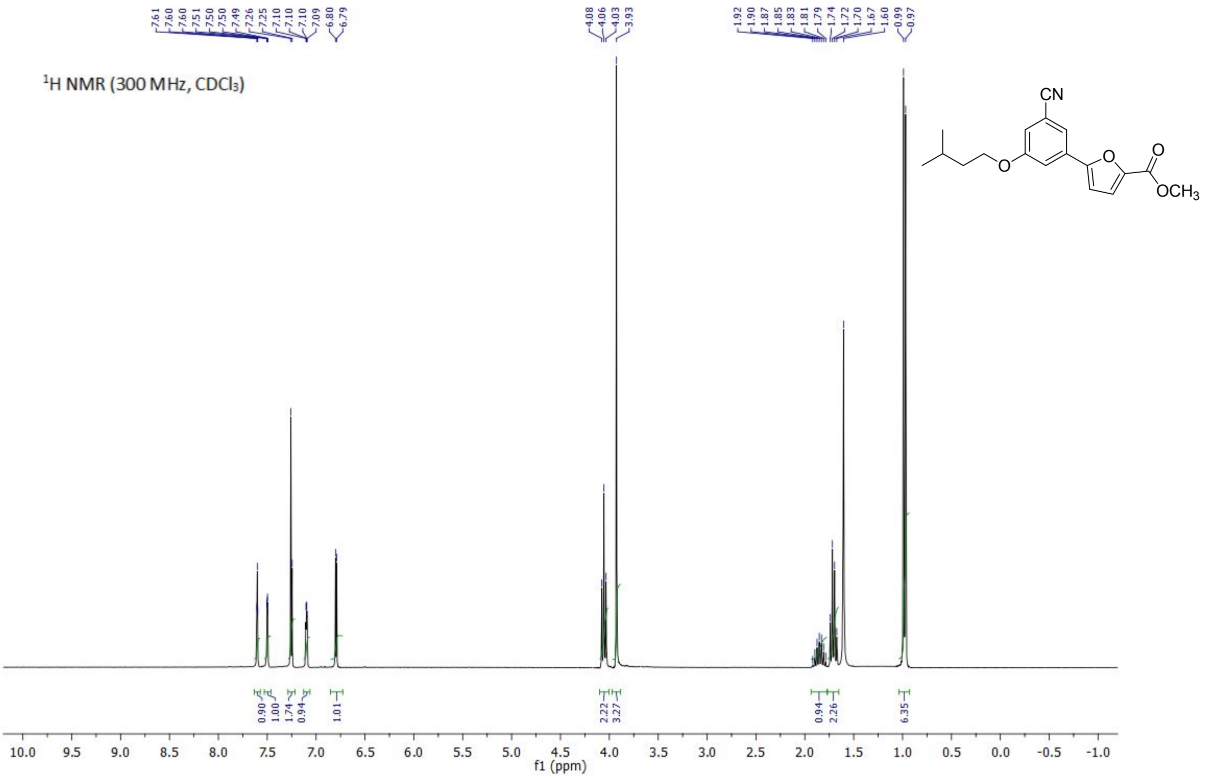

Figure S94. ^{1}H NMR spectrum of **2j**.

- *Methyl 5-(3-cyano-5-(hexyloxy)phenyl)furan-2-carboxylate (**2k**)*

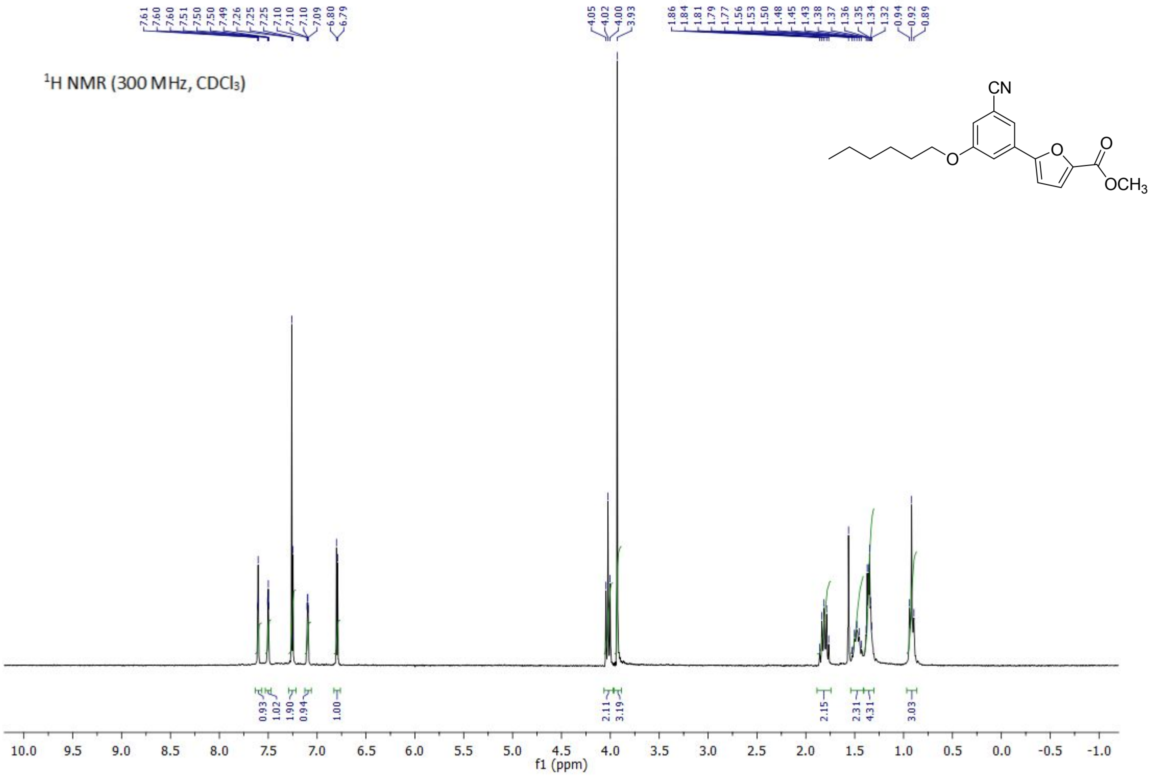

Figure S95. ¹H NMR spectrum of **2k**.

- *Methyl 5-(3-cyano-5-(octyloxy)phenyl)furan-2-carboxylate (**2l**)*

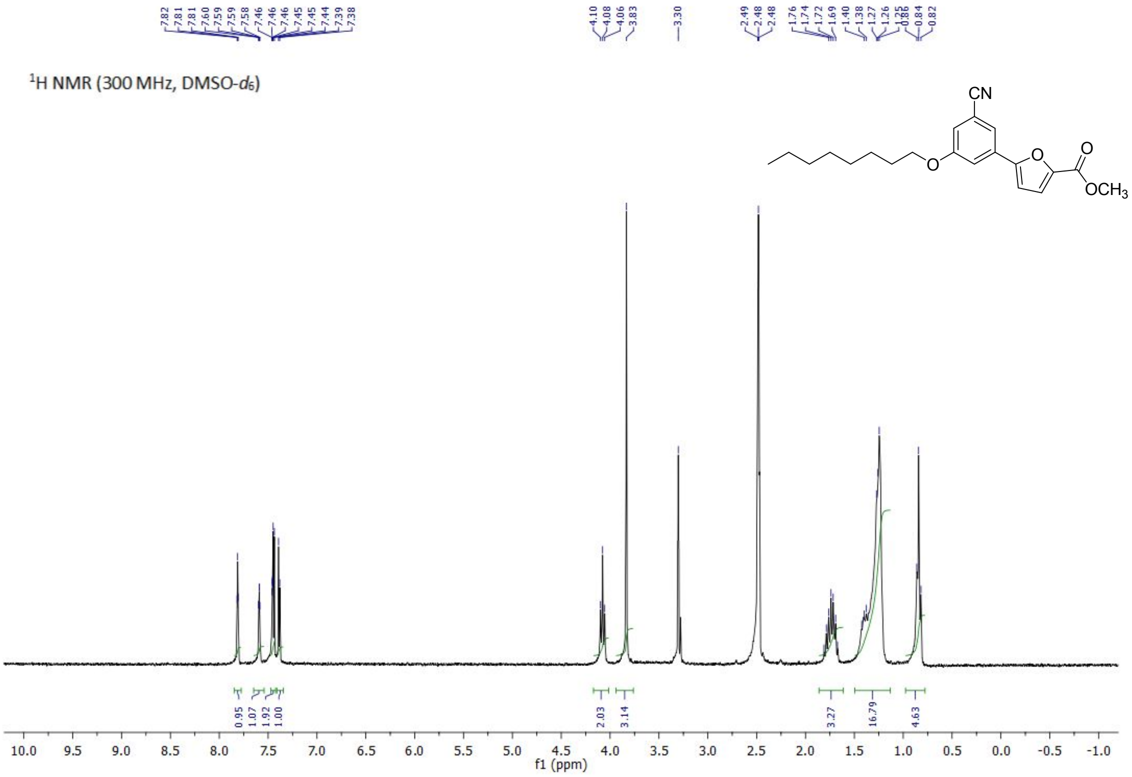

Figure S96. ¹H NMR spectrum of **2l**.

- *Methyl 5-(3-(but-2-en-1-yloxy)-5-cyanophenyl)furan-2-carboxylate* (***2m***)

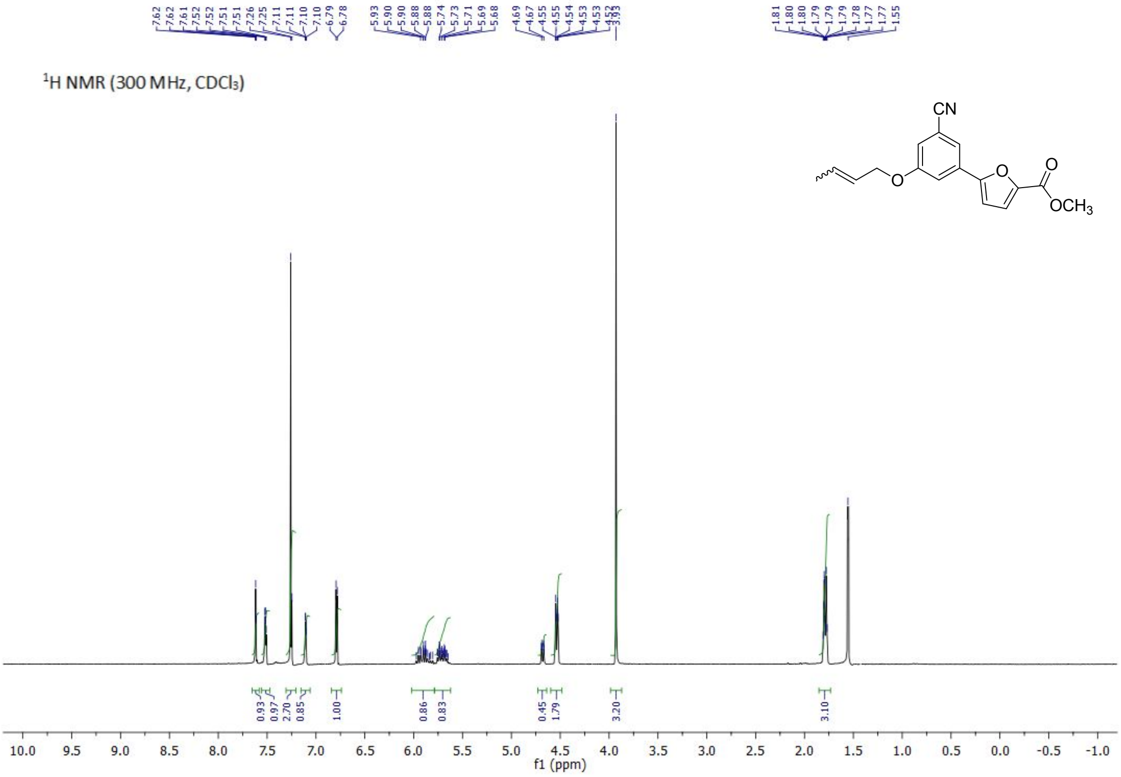

Figure S97. ^{1}H NMR spectrum of **2m**.

- *Methyl 5-(3-cyano-5-((2E,4E)-hexa-2,4-dien-1-yloxy)phenyl)furan-2-carboxylate* (***2n***)

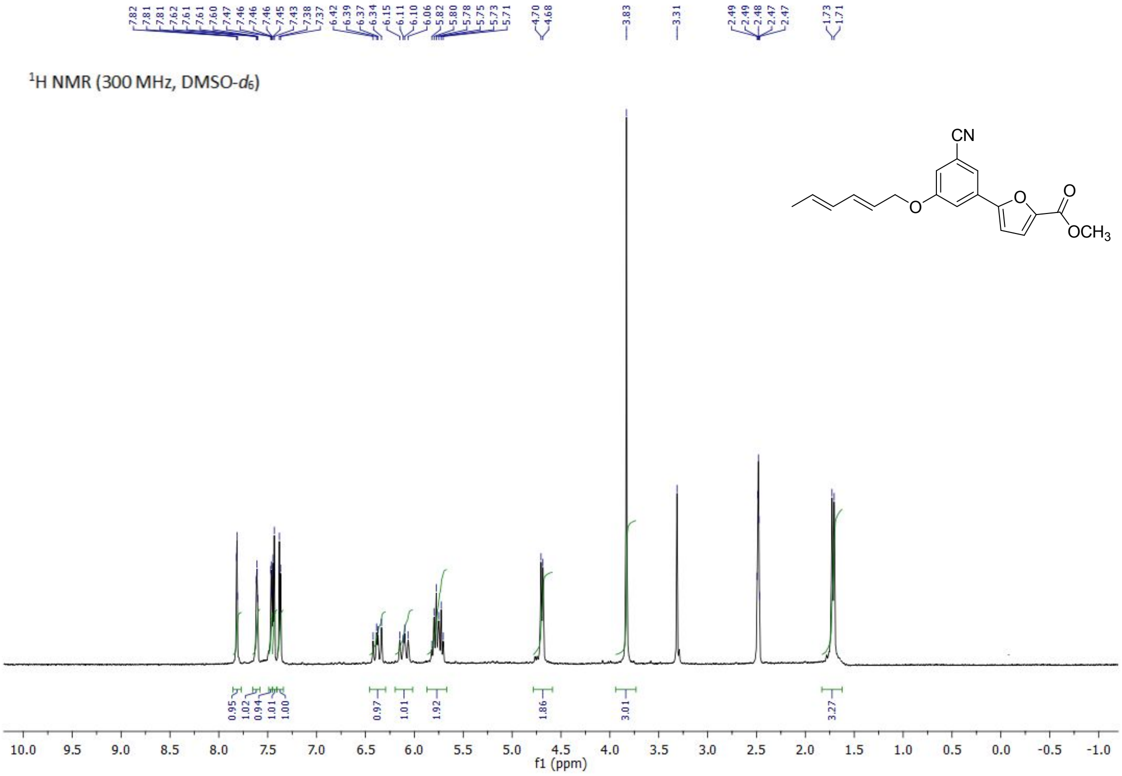

Figure S98. ^{1}H NMR spectrum of **2n**.

- *Methyl 5-(3-cyano-5-((2E,4E,6E)-octa-2,4,6-trien-1-yloxy)phenyl)furan-2-carboxylate (**2o**)*

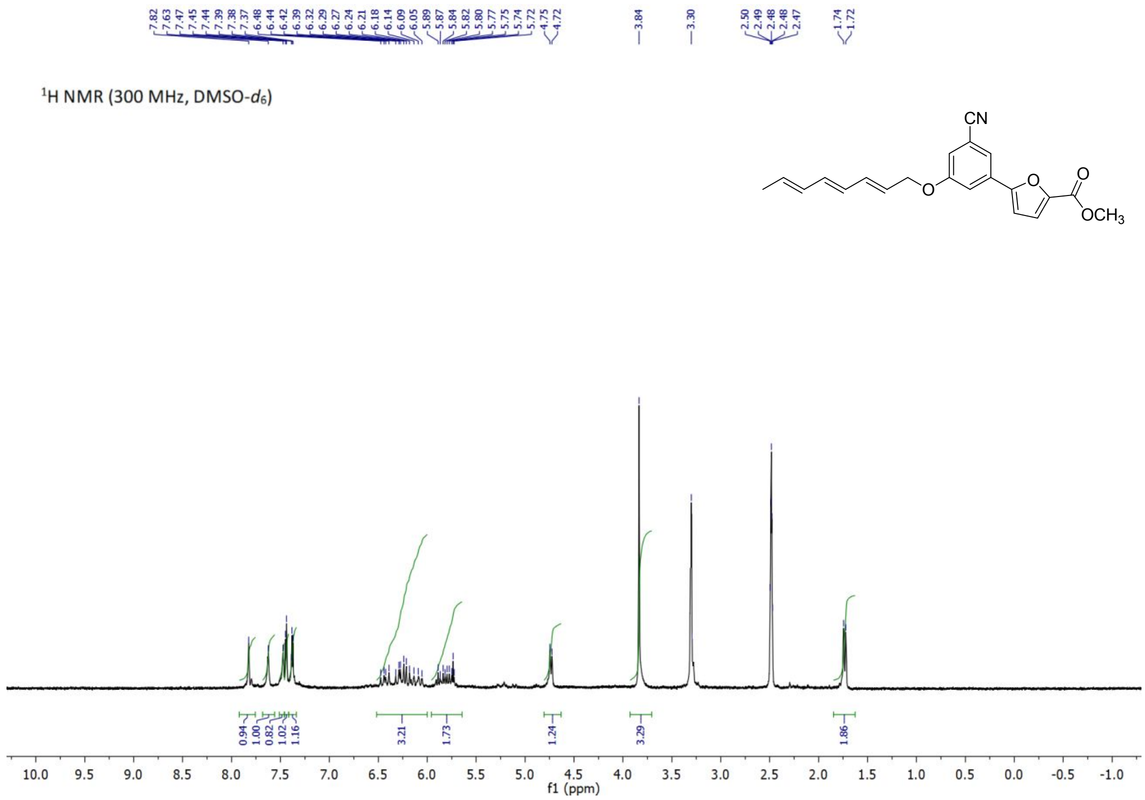

Figure S99. ^{1}H NMR spectrum of **2o**.

- *Methyl 5-(3-cyano-5-hydroxyphenyl)furan-2-carboxylate (**3a**)*

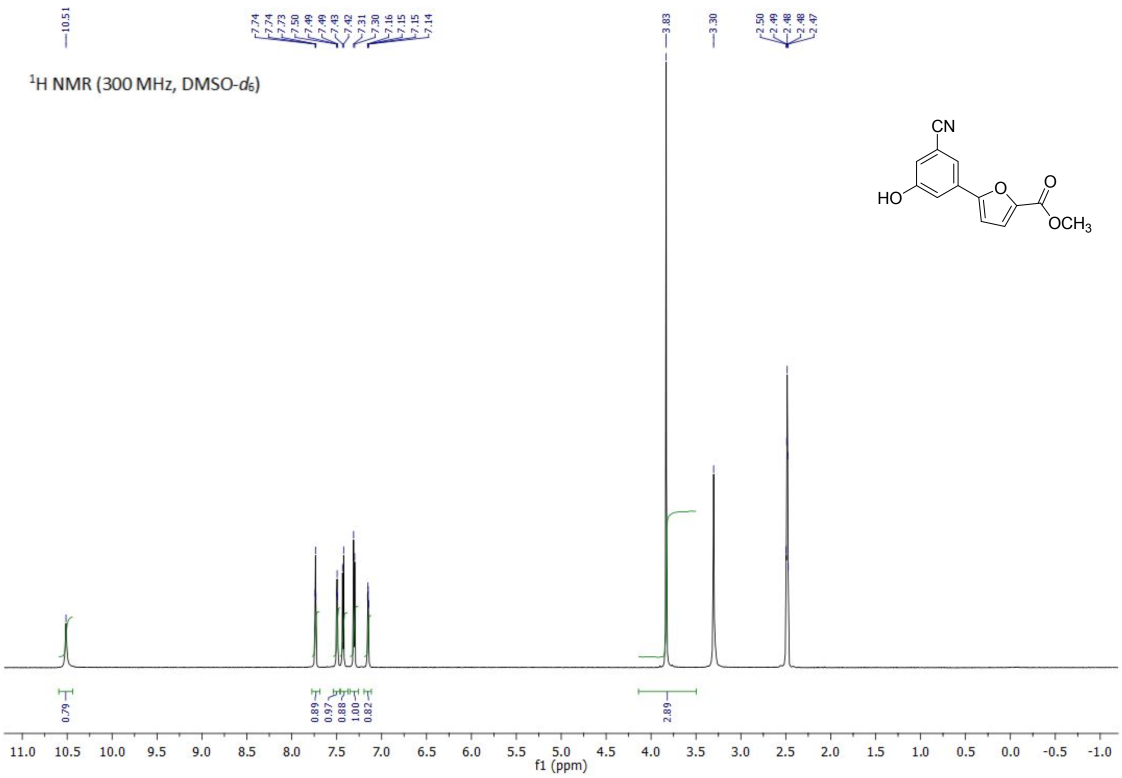

Figure S100. ^{1}H NMR spectrum of **3a**.

- *Ethyl 5-(3-cyano-5-hydroxyphenyl)furan-2-carboxylate (**3b**)*

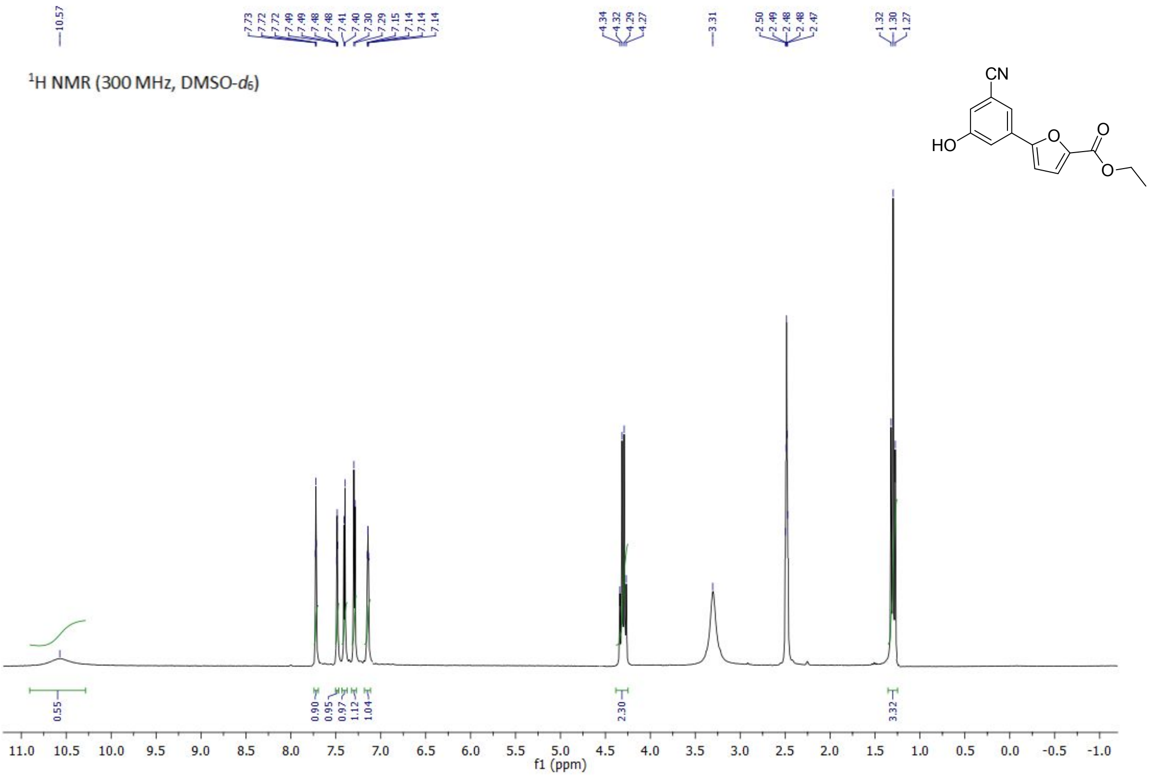

Figure S101. ^{1}H NMR spectrum of **3b**.

- Propyl 5-(3-cyano-5-hydroxyphenyl)furan-2-carboxylate (**3c**)

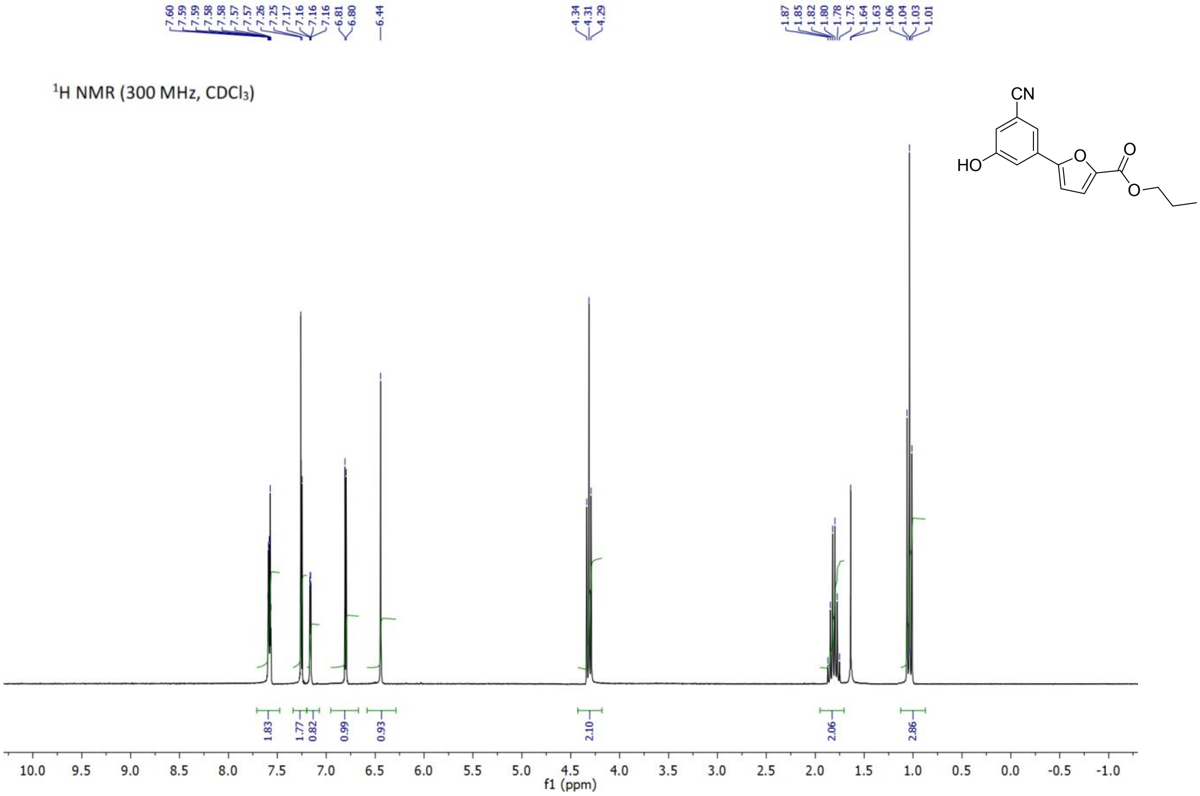

Figure S102. ^{1}H NMR spectrum of **3c**.

- *Ethyl furan-2-carboxylate (**4a**)*

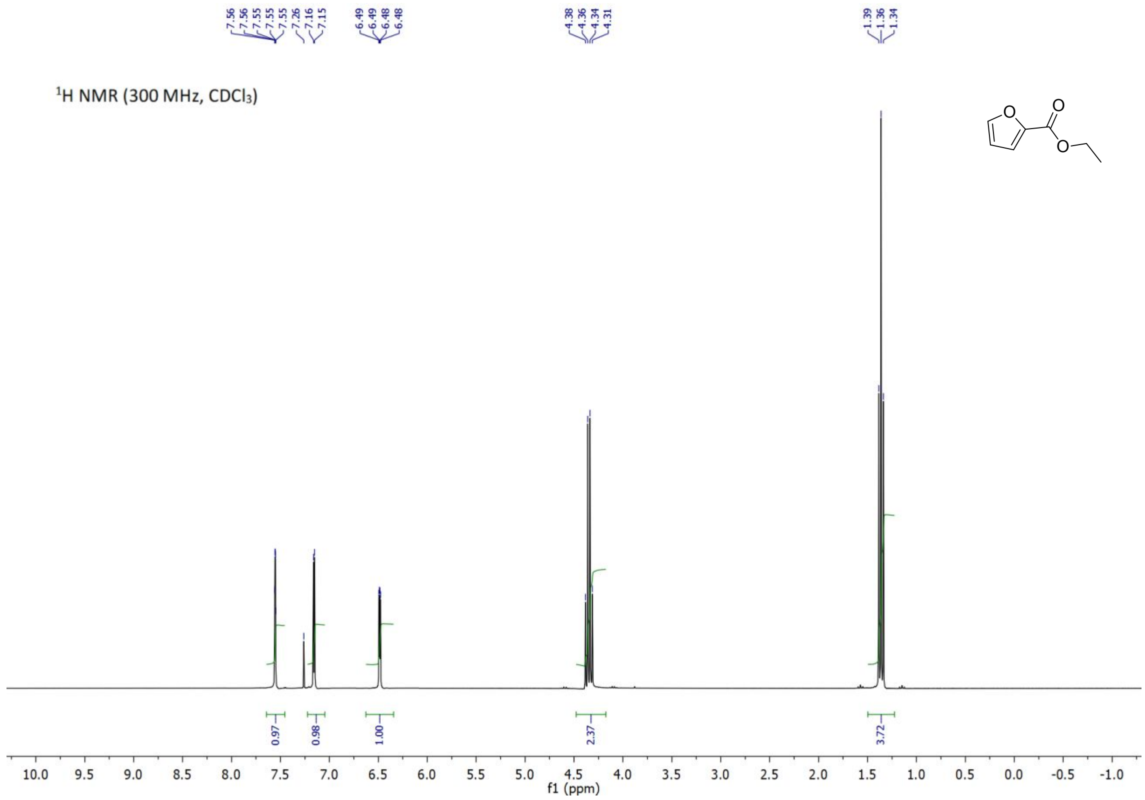

Figure S103. ^{1}H NMR spectrum of **4a**.

- *Propyl furan-2-carboxylate (**4b**)*

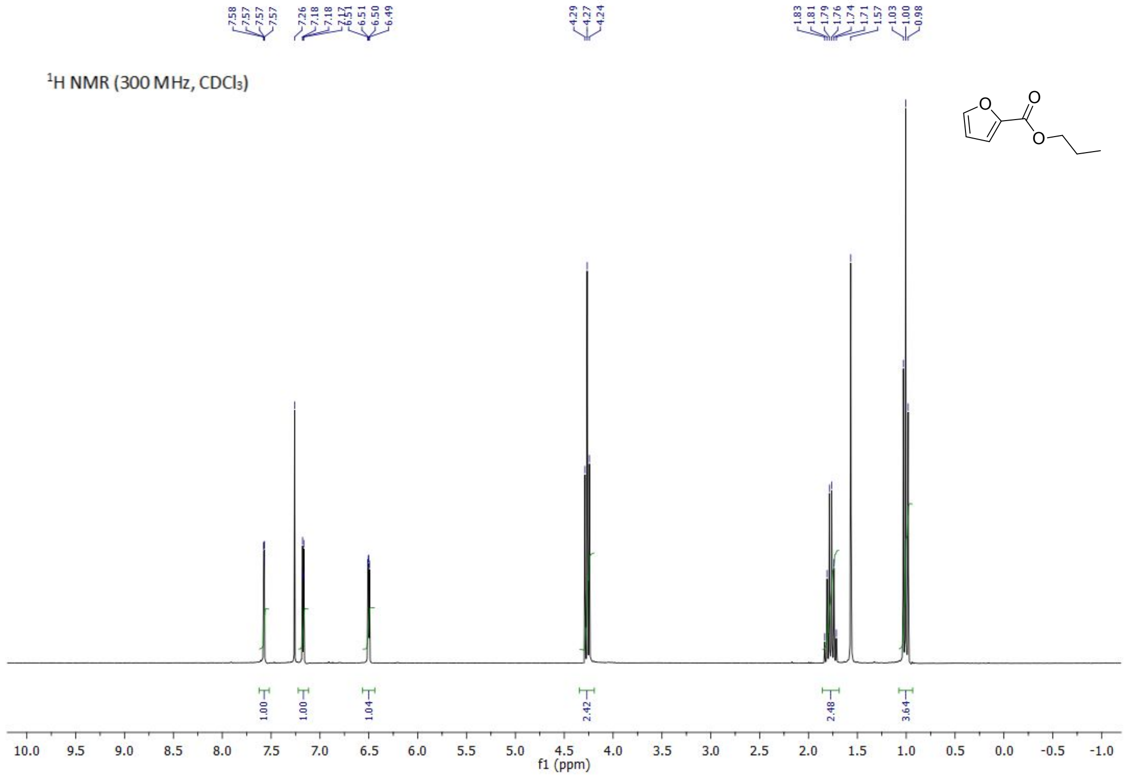

Figure S104. ^{1}H NMR spectrum of **4b**.

- *(2E,4E,6E)-Octa-2,4,6-trien-1-ol*

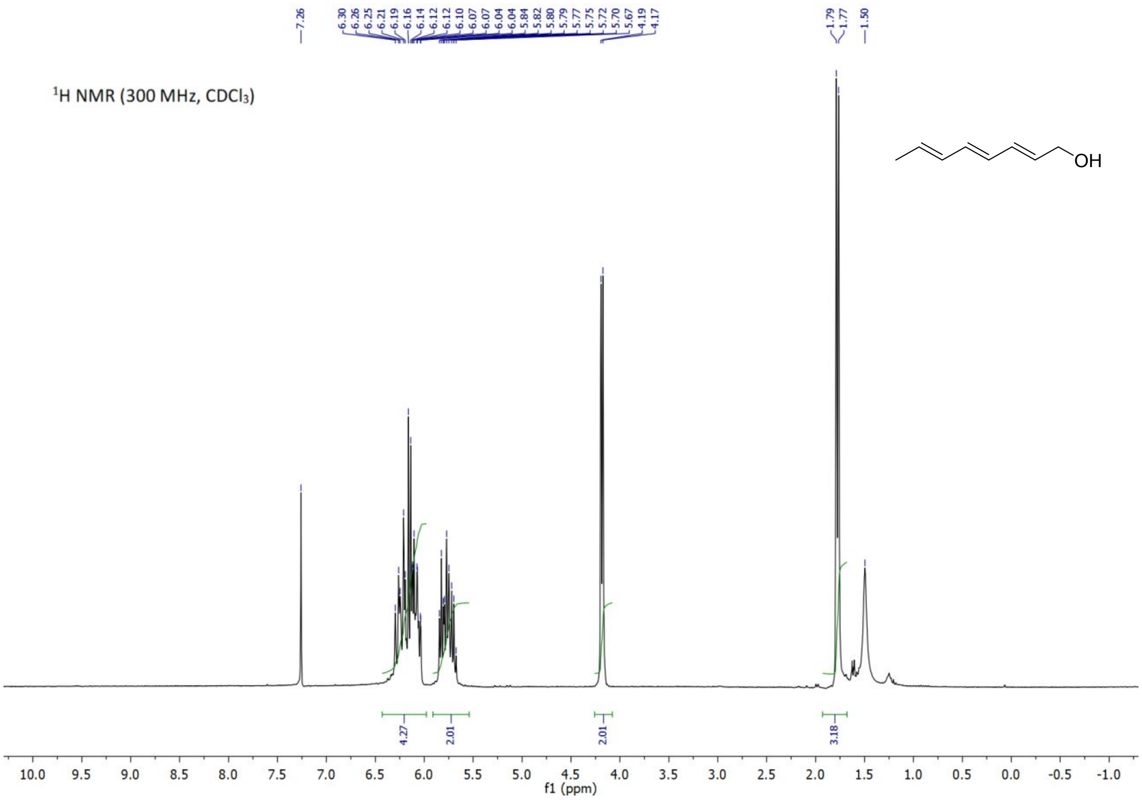

Figure S105. ^{1}H NMR spectrum of (2*E*,4*E*,6*E*)-octa-2,4,6-trien-1-ol.

3. Biological evaluation

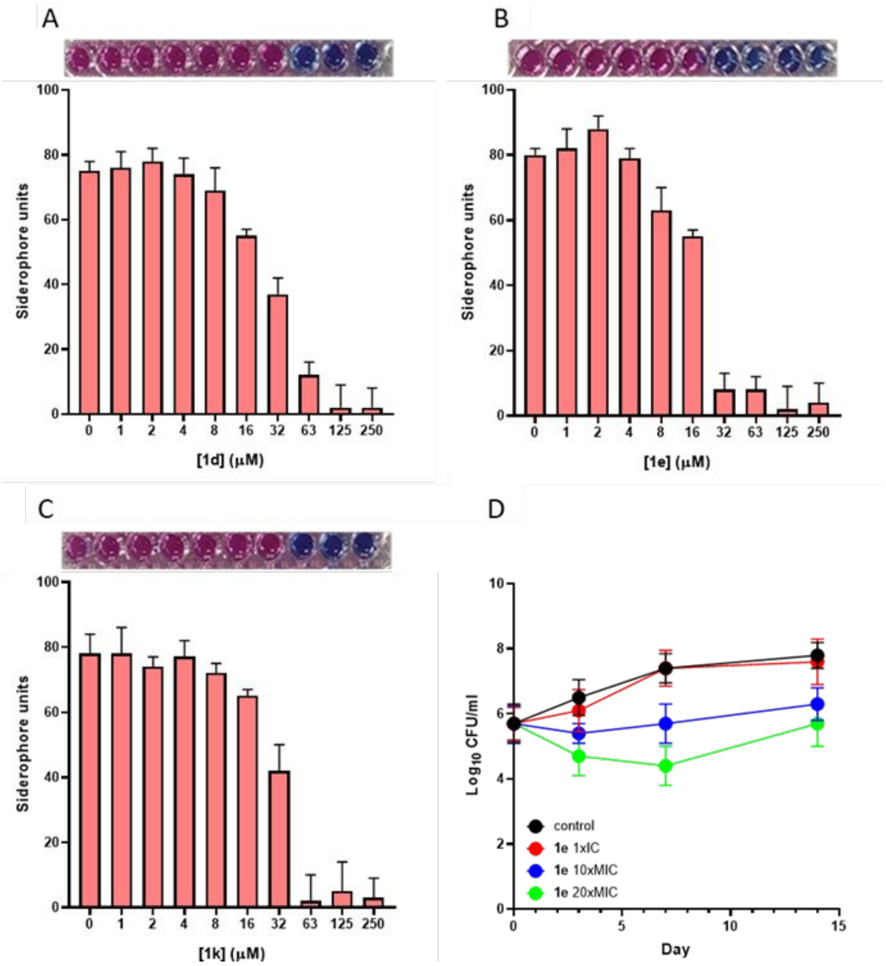

Figure S106. Antimycobacterial activity. Panels **A**-**C** show the REMA MIC determination and Universal CAS assay performed on *M. bovis BCG* cells grown in the presence of different concentrations of **1d** (**A**), **1e** (**B**) and **1k** (**C**). The absorbance at 630 nm of the iron-CAS complex was read, and siderophore activity was determined as previously reported. Bars represent mean and standard deviation of three independent experiments. Panel **D** represents the time killing assay performed for compound **1e**.

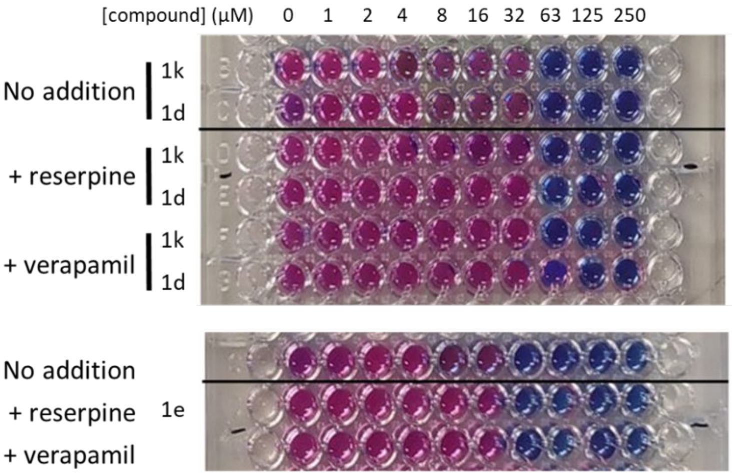

Figure S107. REMA MIC determination of compounds **1d**, **1e** and **1k** on *M. bovis* BCG, performed in the presence of the two efflux pump inhibitors reserpine and verapamil.

A

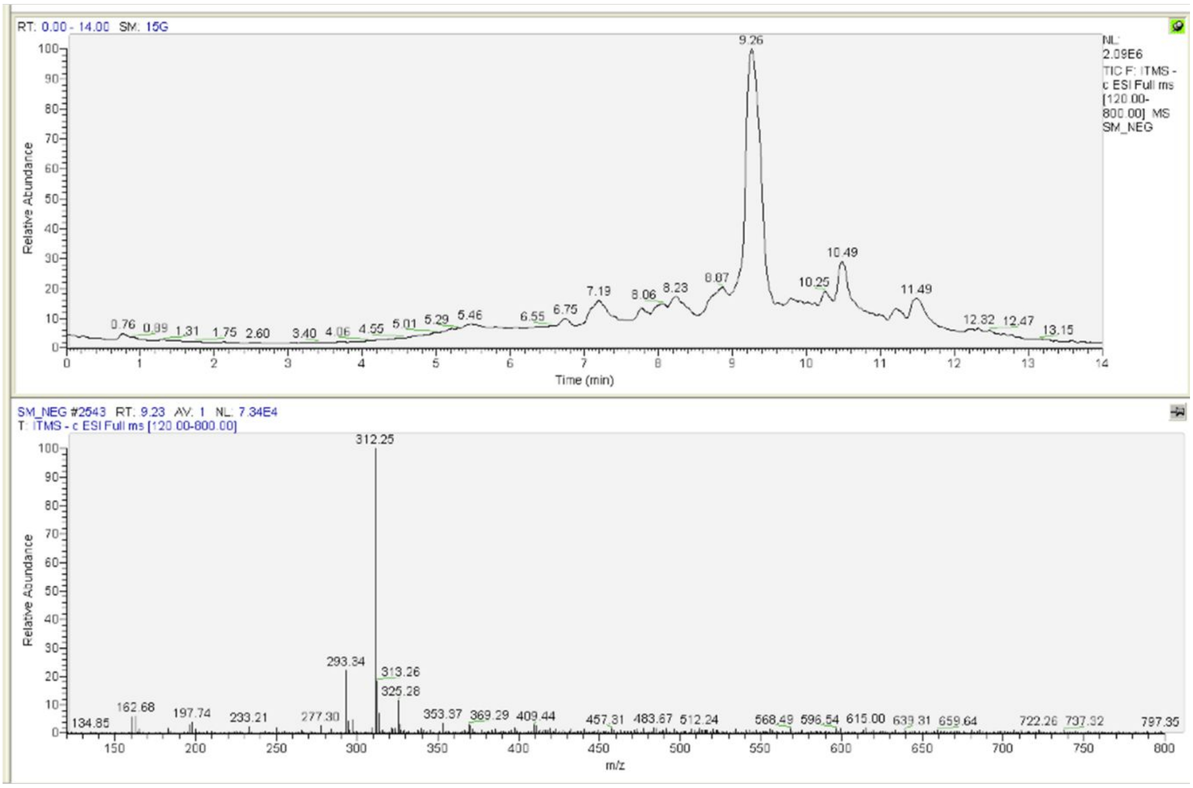

B

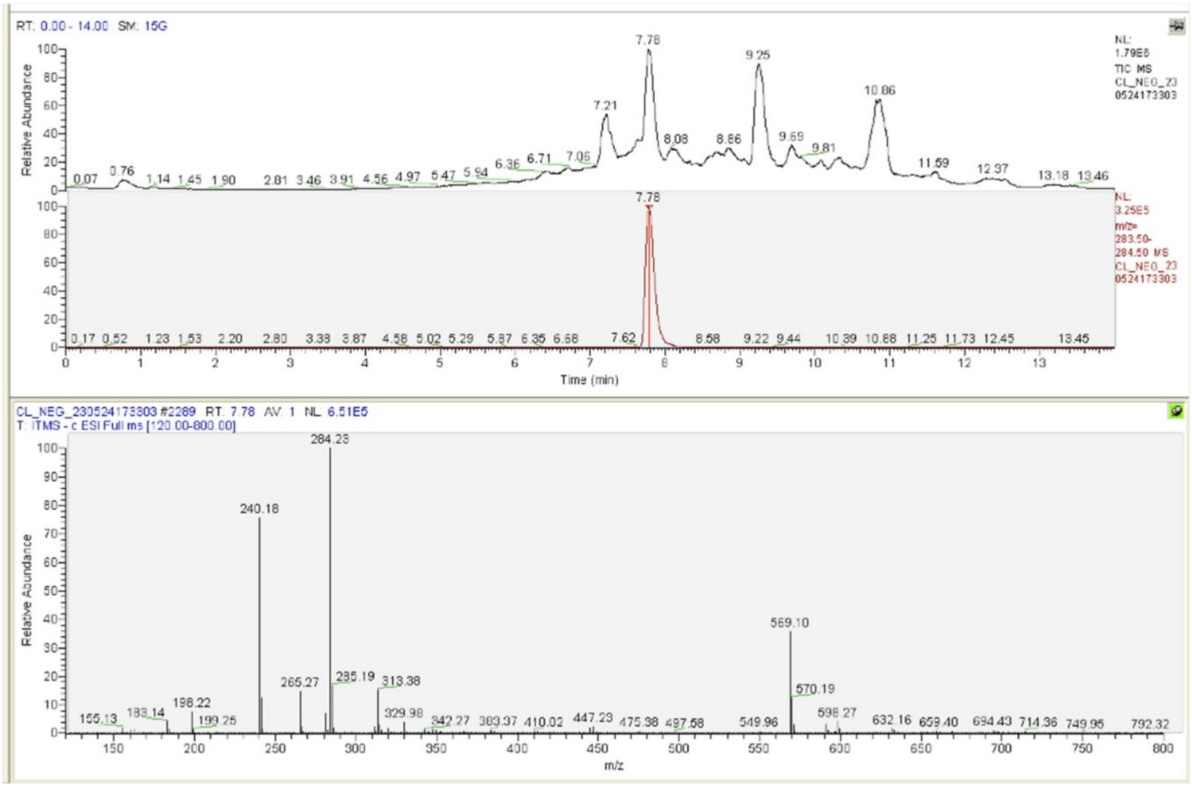

Figure S108. (**A**) UHPLC/LC-MS of compound **2ea** recorded using negative mode for ion detection analysis. (**B**) UHPLC/LC-MS the extracted lysate of *M. bovis* BCG cells grown in the presence of compound **2ea.** The peak at 7.87 min with m/z = 284.12 is in complete agreement with acid substructure obtained after hydrolysis.

4. Molecular modelling studies

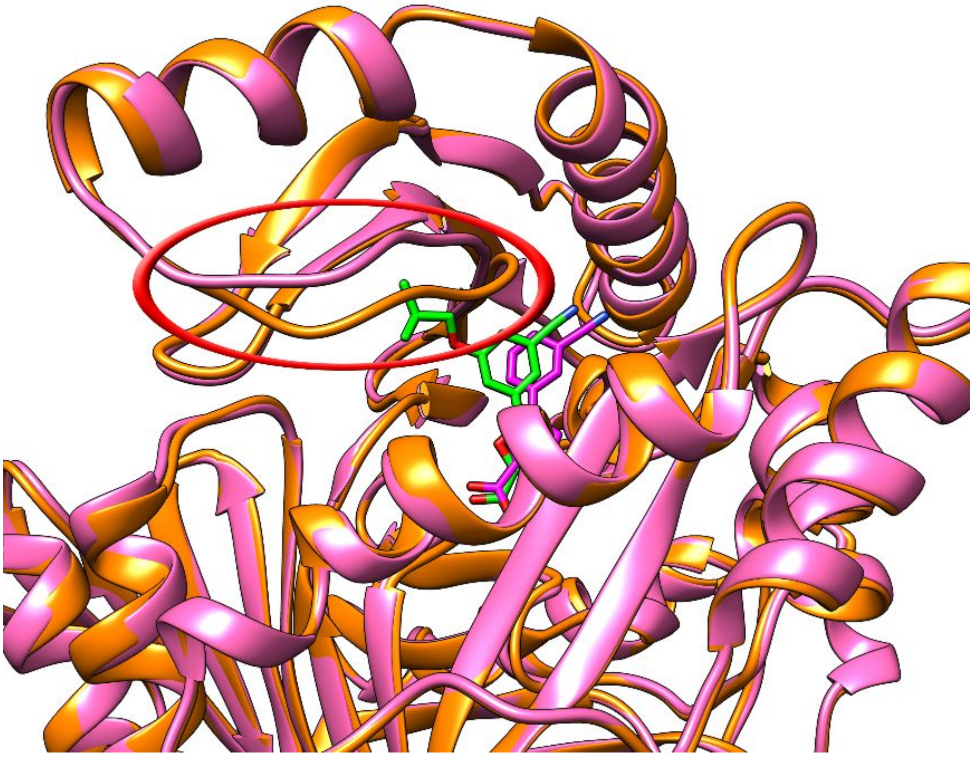

Figure S109. Ribbon representation of the minimized average structure of **I** (orange) and **1e** (pink) in complex with the MbtI binding site. The G270-G277 loop region is highlighted.

5. HPLC chromatograms of polymersomes

A

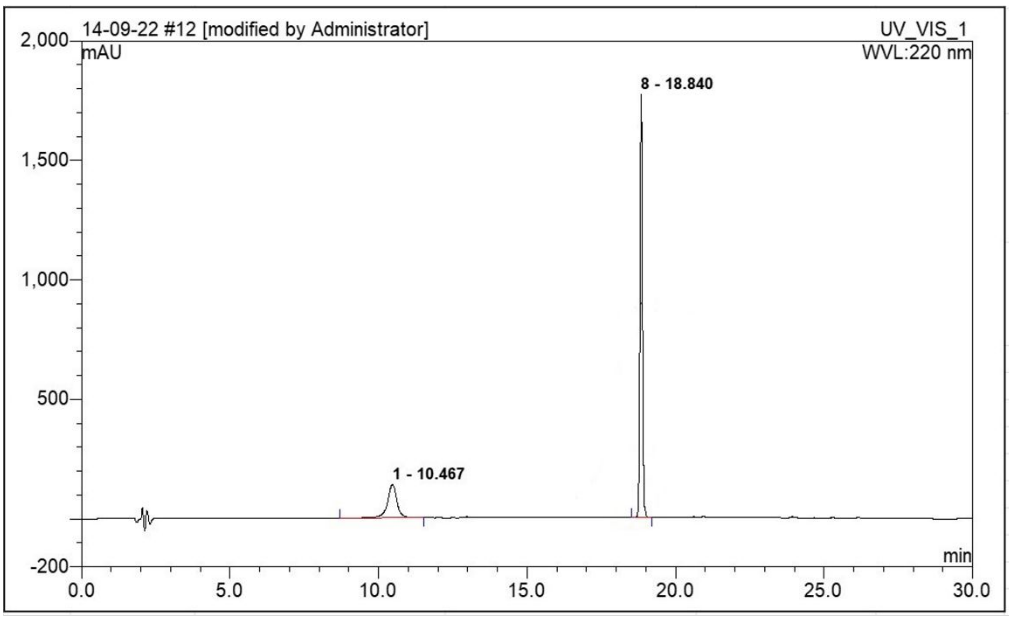

B

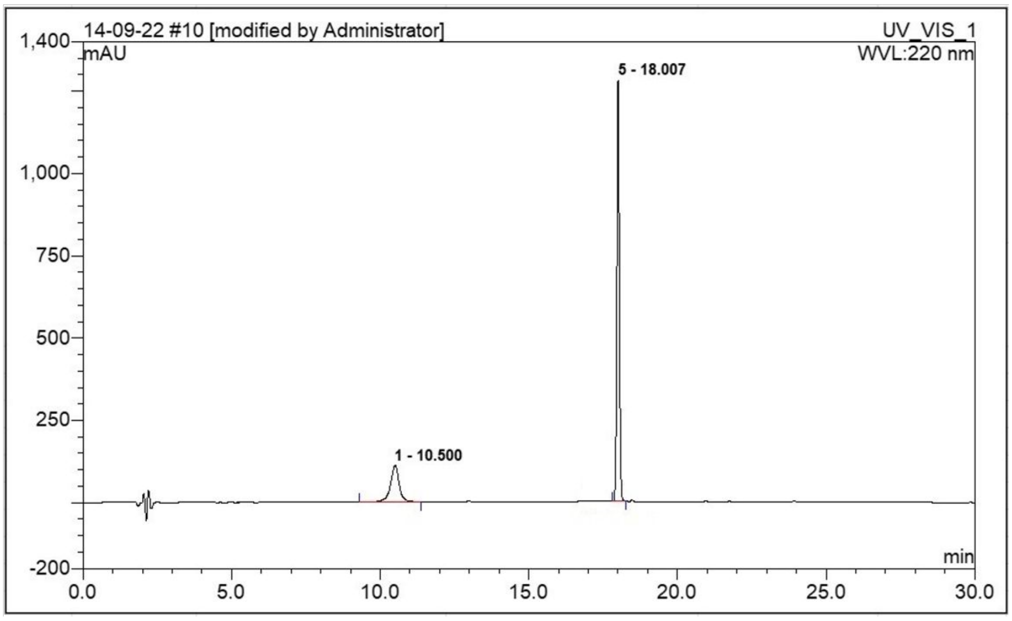

Figure S110. HPLC chromatograms in acidic conditions of (**A**) **2ea**, and (**B**) **2eb**. The peak at 10.467 or 10.500 corresponds to PMPC-PDPA, whereas the second peak (18.840 for **2ea**, 18.007 for **2eb**) corresponds to the loaded drug.

6. DLS analysis of polymersomes

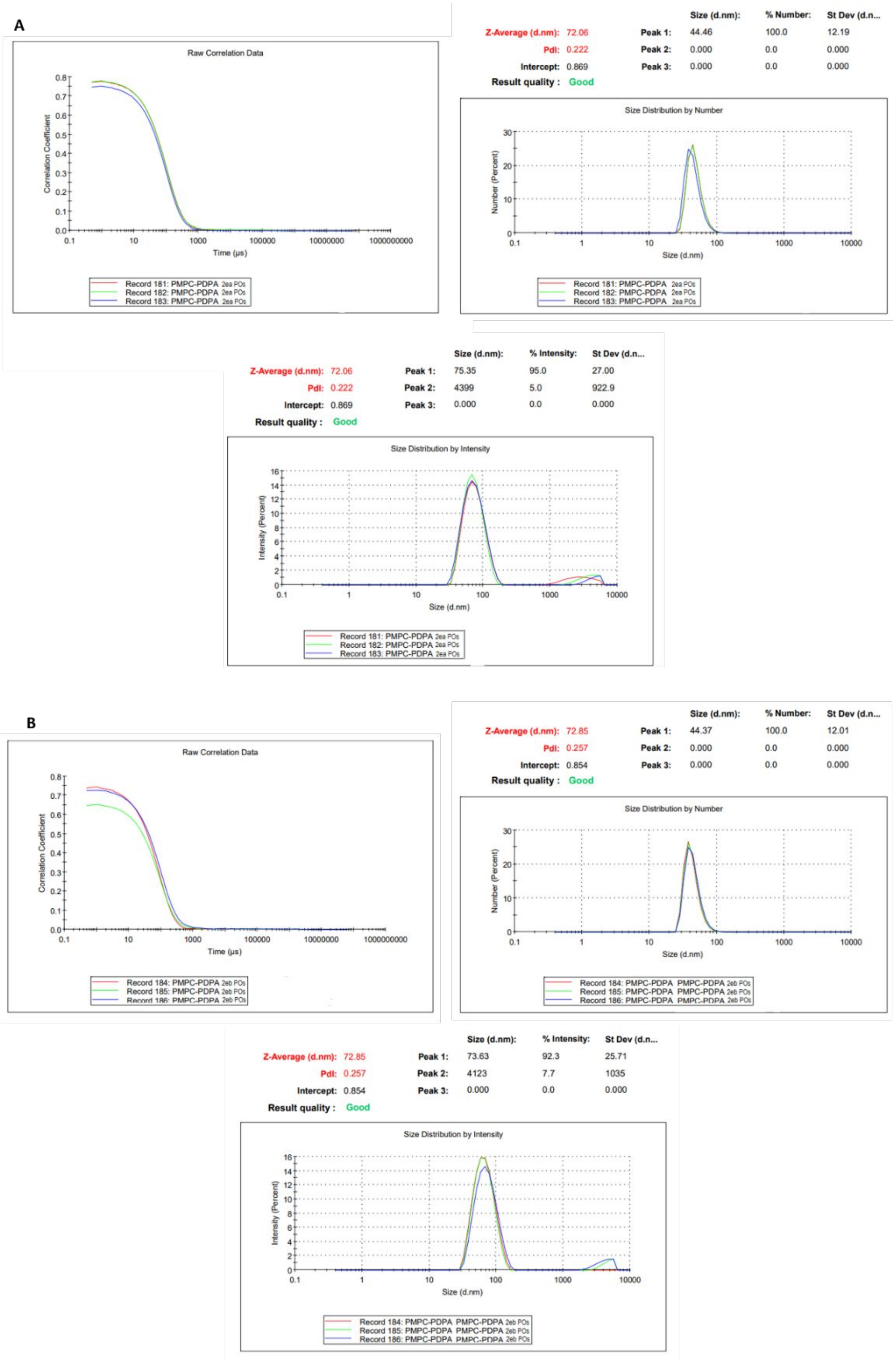

Figure S111. Correlogram, size distribution by number, and size distribution by intensity evaluated by Dynamic Light Scattering (DLS) assays of one batch of (**A**) **2ea-POs**, and (**B**) **2eb-POs**. The DLS values reported in the main text are the average between the DLS analysis of three different batches for each sample.

7. TEM images of polymersomes

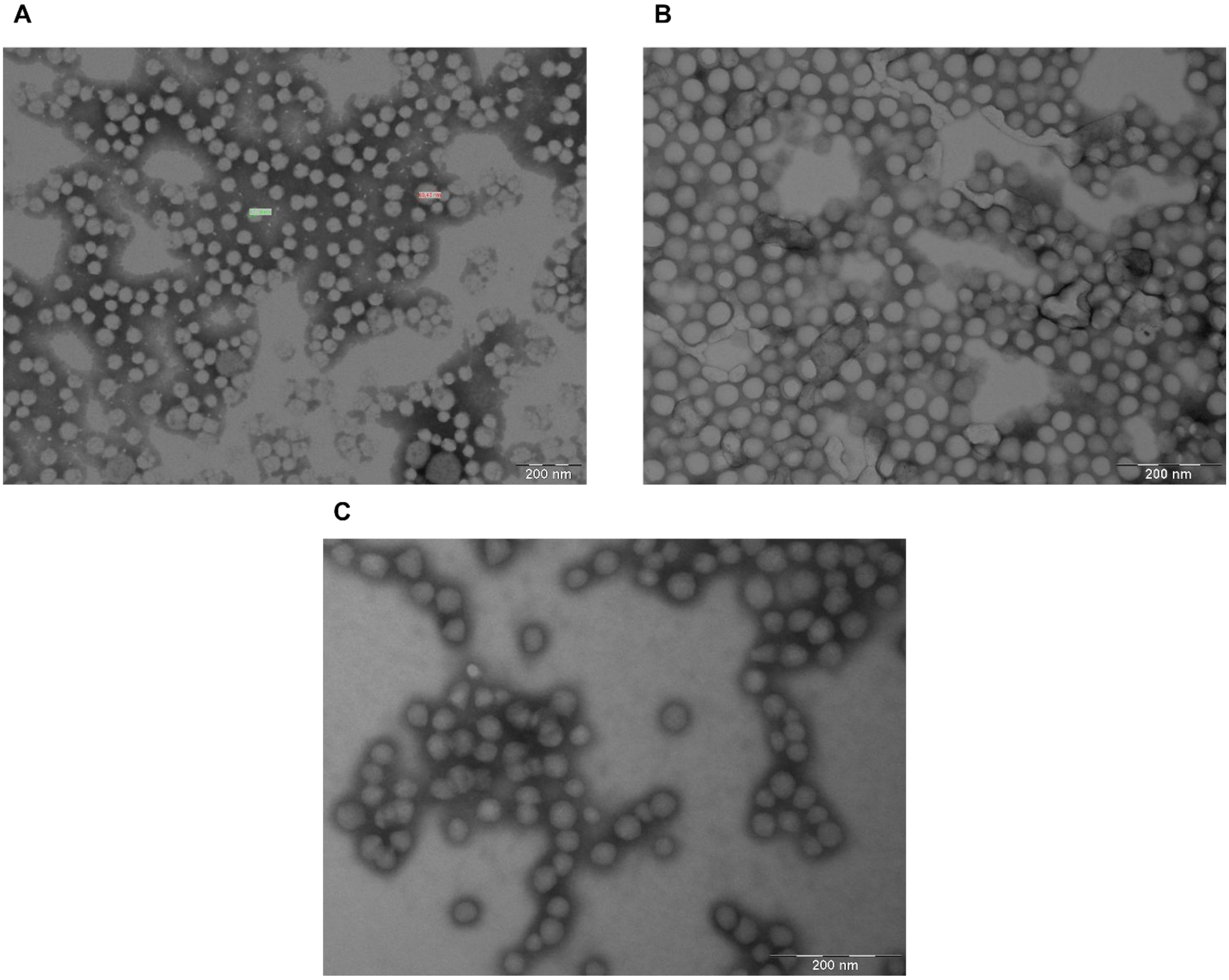

Figure S112. Transmission electron micrograph of PMPC-PDPA polymersomes stained with phosphotungstic acid. (**A**) Empty POs; (**B**) **POs2ea** and (**C**) **POS2eb** after six months from the formulation.

8. Cytotoxicity assays on the free and encapsulated compounds

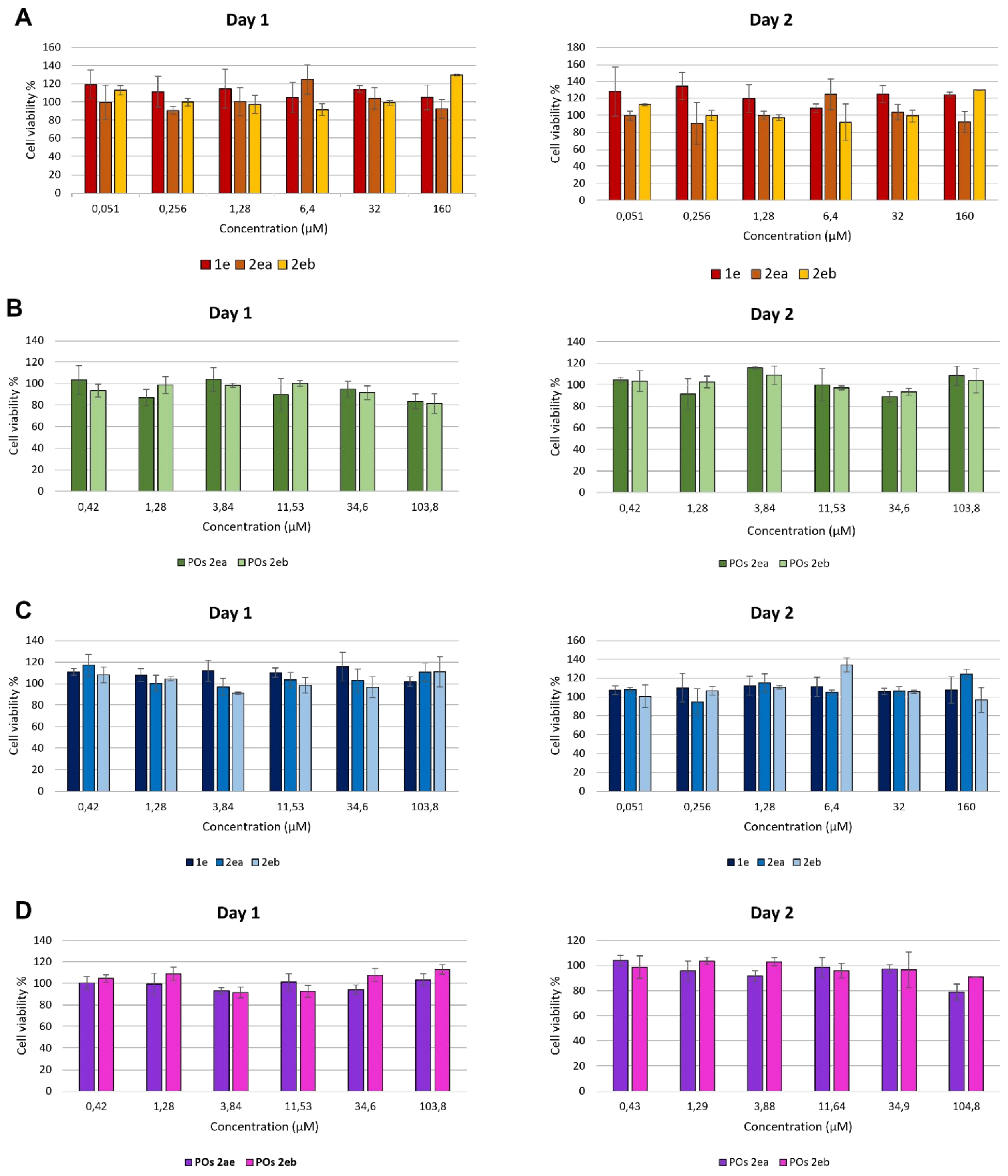

Figure S113. Investigation of the cytotoxicity effect of the free and encapsulated compounds. Spectrofluorometer (plate reader) measure of cell viability after treatment with either free and encapsulated drugs at increasing concentrations at day 1 (left panels) and day 2 (right panels). Black lines represent mean ± SD. Data are from three independent experiments. (**A**) THP-1 cell viability treated with free drugs (**1e**, **2ea**, and **2eb**). (**B**) THP-1 cell viability treated with encapsulated drugs (**POs2ea**, and **POs2eb**). (**C**) HLF cell viability treated with free drugs (**1e**, **2ea**, and **2eb**). (**D**) HLF cell viability treated with encapsulated drugs (**POs2ea** and **POs2eb**).
